# Reduced Nicotine Cigarettes and E-Cigarettes in High-Risk Populations: 3 Randomized Clinical Trials

**DOI:** 10.1001/jamanetworkopen.2024.31731

**Published:** 2024-09-06

**Authors:** Stephen T. Higgins, Stacey C. Sigmon, Jennifer W. Tidey, Sarah H. Heil, Diann E. Gaalema, Dustin C. Lee, Michael J. DeSarno, Elias M. Klemperer, Katherine E. Menson, Patricia A. Cioe, Shirley Plucinski, Rhiannon C. Wiley, Eva Orr

**Affiliations:** 1 University of Vermont Tobacco Center of Regulatory Science, University of Vermont, Burlington; 2 Center for Alcohol and Addiction Studies, Brown University, Providence, Rhode Island; 3 Behavioral Pharmacology Research Unit, Johns Hopkins University School of Medicine, Baltimore, Maryland

## Abstract

**Importance:**

Prohibiting the sale of commonly preferred e-cigarette flavors (eg, fruity and sweet) to discourage use among youths poses a risk of diminishing efforts to decrease smoking in adults.

**Objective:**

To compare reductions in smoking achieved in adults with psychiatric conditions or lower educational level using very low nicotine content (VLNC) cigarettes alone, combined with e-cigarettes limited to tobacco flavor (TF), or combined with e-cigarettes in participant-preferred flavors.

**Design, Setting, and Participants:**

Three randomized clinical trials were conducted for 16 weeks from October 2020 through November 2023 at the University of Vermont, Brown University, and Johns Hopkins University. Participants were adults who smoked daily and were not planning to quit in the next 30 days. These participants were from 3 at-risk populations: those with affective disorders, exemplifying mental illness; those with opioid use disorder, exemplifying substance use disorders; and females of reproductive age with a high-school education or less, exemplifying lower educational level. Participants were randomly assigned to 1 of 4 experimental conditions: (1) normal nicotine content (NNC) cigarettes only; (2) VLNC cigarettes only; (3) VLNC cigarettes plus e-cigarettes with classic TF (hereafter, VLNC + TF); and (4) VLNC cigarettes plus e-cigarettes with preferred flavors (hereafter, VLNC + PF).

**Interventions:**

The NNC cigarettes contained 15.8 mg nicotine/g tobacco, the VLNC cigarettes contained 0.4 mg nicotine/g tobacco, the VLNC + TF had pods containing 5% nicotine by weight and only classic TF, and the VLNC + PF had pods containing 5% nicotine in 8 flavors (including fruity and sweet) from which participants selected 3 flavors.

**Main Outcomes and Measures:**

The primary outcome was mean total cigarettes smoked per day (CPD) during week 16. Tobacco-related biomarkers were assessed, including total 4-(methylnitrosamino)-1-(3-pyridyl)-1-butanol (NNAL), a tobacco-specific carcinogen.

**Results:**

A total of 326 participants (mean [SD] age, 40.09 [10.79] years; 243 females [74.5%]) from 3 randomized clinical trials were included. The VLNC cigarettes decreased total CPD, with least square (LS) means (SEMs) of 22.54 (1.59) in the NNC, 14.32 (1.32) in the VLNC, 11.76 (1.18) in the VLNC + TF, and 7.63 (0.90) in the VLNC + PF conditions. Each VLNC condition differed significantly from NNC, with an adjusted mean difference (AMD) of −8.21 (95% CI, −12.27 to −4.16; *P* < .001) in the VLNC, −10.78 (95% CI, −14.67 to −6.90; *P* < .001) in the VLNC + TF, and −14.91 (95% CI, −18.49 to −11.33; *P* < .001) in the VLNC + PF conditions. Participants in the VLNC + PF condition also decreased smoking below the VLNC and the VLNC + TF conditions (AMDs, −6.70 [95% CI, −9.84 to −3.55; *P* < .001] and −4.13 [95% CI, −7.05 to −1.21; *P* = .02]); the VLNC and VLNC + TF conditions did not differ significantly. Consistent with decreases in CPD, NNAL levels in the VLNC + PF condition were lower than in all other conditions, with AMDs (in pmol/mg creatinine) of −0.94 (95% CI, −1.41 to −0.47; *P* < .001) compared with the NNC condition, −0.47 (95% CI, −0.87 to −0.08; *P* = .03) compared with the VLNC condition, and −0.46 (95% CI, −0.83 to −0.10; *P* = .04) compared with the VLNC + TF condition.

**Conclusions and Relevance:**

These results provide further evidence that a reduced-nicotine standard for cigarettes has the potential to decrease smoking and tobacco-toxicant exposure in high-risk populations and that these effects may be enhanced when adults can access e-cigarettes in commonly preferred flavors.

**Trial Registration:**

ClinicalTrials.gov Identifiers: NCT04092387, NCT04090879, NCT04092101

## Key Points

**Question:**

Do e-cigarettes in flavors selected from a range of appealing options enhance the decreases in cigarette smoking achieved by reducing the nicotine content of combusted cigarettes to minimally addictive levels?

**Findings:**

In 3 randomized clinical trials involving 326 participants, decreases in cigarettes smoked daily, resulting from smoking cigarettes with reduced nicotine content, were significantly larger when adults from at-risk populations had access to e-cigarettes in their preferred flavors.

**Meaning:**

Findings of this study indicate that access to e-cigarettes in commonly preferred flavors has the potential to enhance the effect of a nicotine-reduction policy on cigarette smoking in populations with psychiatric conditions or lower education level who are at greatest risk for smoking and associated harm.

## Teaser

This report of 3 randomized clinical trials investigates smoking reduction in adults with psychiatric conditions or lower educational level who use low-nicotine cigarettes alone, combined with tobacco-flavored e-cigarettes, or combined with e-cigarettes in participant-preferred flavors.

## Introduction

Cigarette smoking is overrepresented among individuals with psychiatric conditions and lower educational level.^1,2,3,4,5,6^ To further reduce smoking, the US Food and Drug Administration (FDA) announced plans to implement a national policy to reduce nicotine content in cigarettes to minimally addictive levels.^7,8,9^ Randomized clinical trials (RCTs) in the general population^10,11^ and disadvantaged populations^12,13^ demonstrate that very low nicotine content (VLNC) cigarettes decrease smoking, toxicant exposure, and dependence. Expert consensus is that success of this policy will be conditional on availability of noncombusted alternative products so that those unable or unwilling to quit using nicotine have alternatives to smoking.^14,15^ However, concerns that e-cigarettes in commonly preferred flavors (eg, fruity and sweet) contribute to e-cigarette use in youths have resulted in the FDA restricting e-cigarette access in the US marketplace to only tobacco and more recently menthol flavors. Such limits have the potential to weaken the impact of a nicotine-reduction policy on adults, especially those with comorbid psychiatric conditions or socioeconomic disadvantage who are likely to have greater difficulty quitting nicotine use.^16,17^ Preliminary studies in the general population suggest that e-cigarettes in commonly preferred flavors enhance VLNC smoking reductions, although the concurrent availability of other alternative tobacco products^18,19^ and small sample size^20^ in those studies precluded firm conclusions. The present RCTs aimed to compare reductions in smoking achieved in adults with psychiatric conditions or lower educational level using VLNC cigarettes alone; combined with e-cigarettes limited to tobacco flavor (only flavor with FDA marketing approval at study initiation); or combined with e-cigarettes in 3 participant-preferred flavors selected from 8 flavors, including fruity and sweet. We hypothesized that VLNC cigarettes in combination with e-cigarettes in preferred flavors would produce the greatest smoking reductions.

## Methods

### Study Design

We report on 3 parallel, 16-week RCTs. Trial protocols (Supplement 1) were designed to be parallel across populations except for the inclusion and exclusion criteria and monitored by an independent data and safety monitoring board. The University of Vermont, Brown University, and Johns Hopkins University School of Medicine Institutional Review Boards approved each RCT. Participants provided written informed consent. We followed the Consolidated Standards of Reporting Trials (CONSORT) guidelines.

Each trial was conducted at 2 of 3 sites (University of Vermont, Brown University, and Johns Hopkins University) between October 2020 and November 2023. Across trials, adults who smoked daily and were not planning to quit in the next 30 days participated, including those with affective disorders and opioid use disorder (OUD) as well as females of reproductive age (21-44 years) with lower educational level (≤high school diploma).^3,4,5^

**Figure 1.**
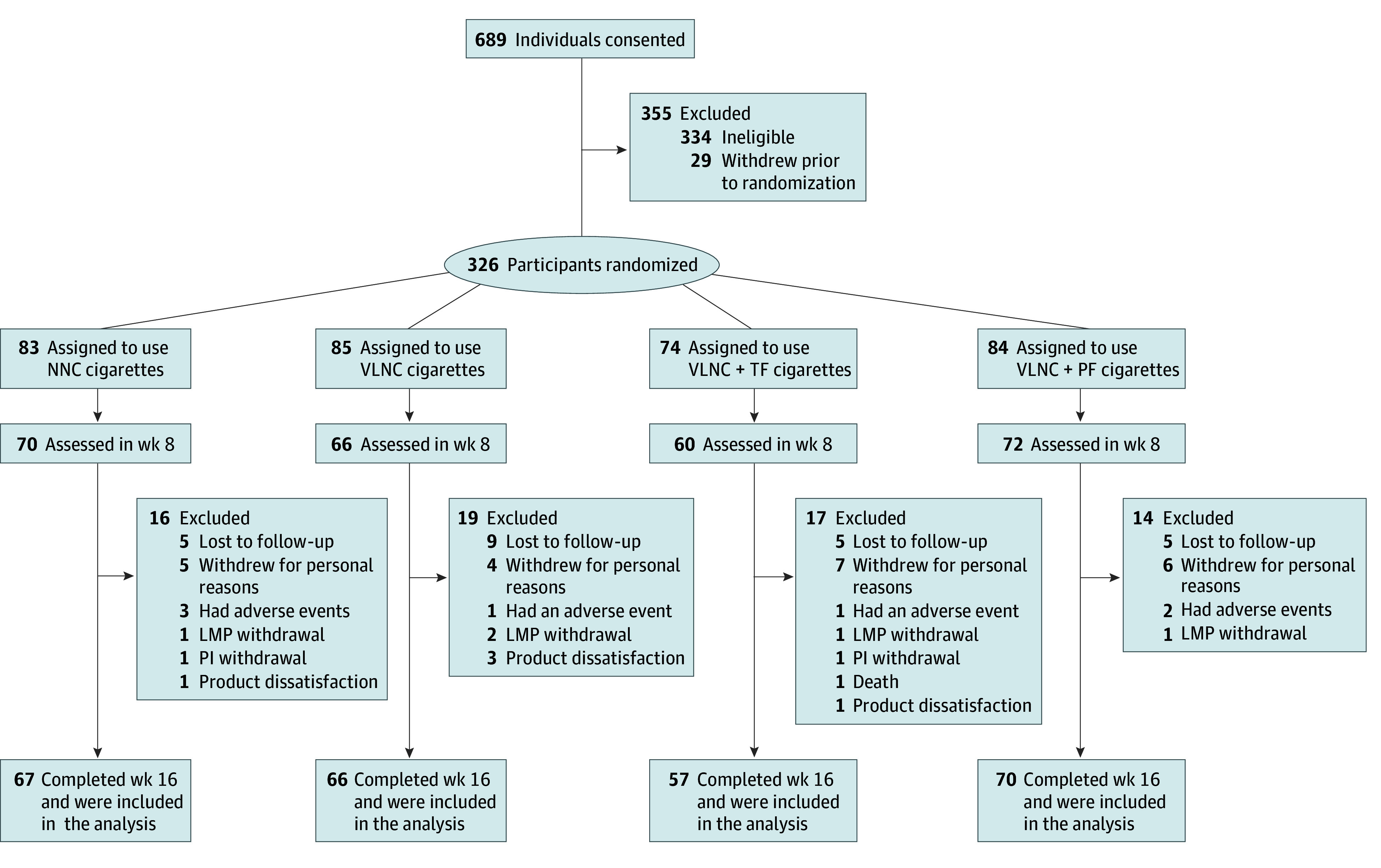
Enrollment, Randomization, Retention, and Reasons for Attrition Across the 4 Experimental Conditions LMP indicates licensed medical professional; NNC, normal nicotine content; PF, e-cigarettes in preferred flavor; PI, principal investigator; TF, e-cigarettes in tobacco flavor; VLNC, very low nicotine content.

Participants were allocated equally across 1 of 4 experimental conditions using block randomization, stratified by study site and menthol cigarette preference: (1) normal nicotine content (NNC) cigarettes only (15.8 mg nicotine/g tobacco); (2) VLNC cigarettes only (0.4 mg nicotine/g tobacco); (3) VLNC cigarettes plus e-cigarettes (JUUL; Juul Labs) with pods containing 5% nicotine by weight and limited to classic tobacco flavor (hereafter, VLNC + TF); and (4) VLNC cigarettes plus e-cigarettes with pods containing 5% nicotine in 8 flavors (classic tobacco, Virginia tobacco, crème brûlée, cucumber, fruit medley, mango, menthol, and mint) from which participants selected 3 preferred flavors (hereafter, VLNC + PF) (Figure 1). Preferred flavors could be changed once during the study. Cigarettes and e-cigarettes were studied under double-blind and open-label conditions, respectively.

Trials were scheduled to begin in January 2020, but the COVID-19 pandemic necessitated postponement until October 2020 and protocol revisions to protect the health of staff and participants. Sample size planned before the pandemic was carried over into the revised protocols (N = 724) but proved unattainable during the pandemic, resulting in a total of 326 participants. Initial sample size estimates were conservative due to a dearth of data on combining VLNC cigarettes with e-cigarettes. The sample size of recruited participants was adequate to generate novel results with important public health implications.

### Study Product

The National Institute on Drug Abuse provided study cigarettes with nicotine content averaged across menthol and nonmenthol cigarettes.^21^ Cigarettes were identical in appearance. Assignment to menthol or nonmenthol cigarettes was based on participant preference. E-cigarettes were purchased from JUUL Labs.

### Participants

Participants were recruited through newspaper and online advertisements and word of mouth. Shared inclusion criteria across trials were age of at least 21 years (US legal age for purchasing cigarettes), 5 or more cigarettes smoked per day (CPD) in the past year, breath carbon monoxide (CO) sample of 8 ppm or higher, no psychiatric conditions with potential to interfere with study completion, sufficient literacy to complete study tasks, no adverse health changes in the past 90 days, no use of e-cigarettes daily or other tobacco products besides commercial cigarettes on 10 or more days in the past 30 days, and access to a computer or telephone for remote assessments (smartphone provided if necessary). Female participants could not be pregnant or nursing and had to be using contraception unless they were surgically sterilized or postmenopausal. Shared exclusion criteria included prior regular use of VLNC cigarettes; plans to quit smoking in the next 30 days, which risked conflating prestudy factors with study effect on quitting; a quit attempt in the past 30 days resulting in more than 3 days of abstinence; use of smoking cessation medications in the past 30 days; exclusive use of roll-your-own cigarettes; positive test result for drugs other than cannabis; binge drinking on 10 or more days in the past 30 days; abnormal vital sign measurements; positive COVID-19 symptoms or test result; recent suicidal ideation or suicide attempt; participation in another research study in the past 30 days; and cohabitation with another participant in the current study.

For trials on psychiatric disorders, inclusion required meeting diagnostic criteria for affective disorders or OUD with stable enrollment in opioid-substitution therapy. Population-specific exclusion criteria included age older than 70 years in the affective disorders and OUD trials and older than 44 years in the reproductive-aged females trial; comorbid substance use disorder in the affective disorders and reproductive-aged females trials; and anticonvulsant use in the OUD and reproductive-aged females trials. Participants were compensated up to $2816. Age, sex, and self-identified race and ethnicity were collected following the National Institutes of Health guidelines. We assessed race and ethnicity as part of characterizing the diversity of the sample.

### Procedures

Participants completed a 2-visit, 1-week baseline assessment. During the first baseline visit, participants received free usual-brand cigarettes for use during the subsequent week to establish baseline CPD. The supply was 150% of self-reported CPD to accommodate increases. Participants used interactive voice response (IVR) daily to report prior-day smoking and other tobacco or nicotine use. Adherence to IVR reporting was compensated at $1.00 per call plus $10.00 bonuses for completing 7 consecutive calls. Participants received the first supply of study cigarettes at the second baseline visit. Those assigned to e-cigarettes participated in an orientation session and received a manual on device operation and storage. Thereafter, participants reported to the clinic weekly for 16 weeks to return unused study product and to receive more supply. Participants received twice the number of cigarettes used during baseline to accommodate smoking increases or missed visits. Those randomly assigned to the e-cigarette conditions received a supply of e-liquid pods sufficient to substitute for baseline smoking (1-pod/pack of cigarettes).

Participants were counseled on using only study products, queried about plans to quit smoking, and offered referrals accordingly. Weekly assessments also included video calls with staff wherein participants completed questionnaires on REDCap (Vanderbilt University) regarding recent substance use, medication use, and mood and anxiety as well as reported any adverse events (AEs); had their vital signs assessed; and completed breath CO, drug toxicology, and pregnancy (biweekly) tests. At the second baseline visit and clinic visits at weeks 8 and 16, participants brought a first-void urine specimen. Urine specimens were analyzed for cotinine (nicotine metabolite), anatabine (tobacco alkaloid), and total 4-(methylnitrosamino)-1-(3-pyridyl)-1-butanol (NNAL, a tobacco-specific N-nitroasmine and carcinogen).^21,22,23,24,25^ Approximately 30 days after the week 16 visit, participants were contacted to assess smoking status. Those reporting abstinence could earn $40 by completing bioverification testing.

### Outcomes

Study outcomes were uniform across populations. The primary outcome was week 16 mean total CPD (using study and nonstudy cigarettes). Secondary outcomes included total CPD across weeks; study and nonstudy CPD; toxicant exposure levels; smoking abstinence outcomes; dependence severity measured using the Fagerström Test for Nicotine Dependence (FTND) total score (ranging from 0 [very low severity] to 7 [high severity]), excluding item 4 on CPD,^26^ and the Brief–Wisconsin Inventory of Smoking Dependence Motives (B-WISDM) Primary Dependence Motives and Secondary Dependence Motives scales (scores ranging from 1 [not true] to 7 [extremely true])^27^; ratings for study and usual-brand cigarettes using the Questionnaire on Smoking Urges–Brief (QSU-Brief) Factor 1 (anticipation of pleasure from smoking) and Factor 2 (anticipation of relief from withdrawal or adverse affect), scored on a 100-point scale ranging from strongly disagree to strongly agree^28^; and total and desire-to-smoke scores using the Minnesota Tobacco Withdrawal Scale (desire to smoke scored on a 5-point scale ranging from 0 [none] to 4 [severe]).^29^ Cigarette Purchase Task^30^ and exploratory outcomes assessed using the Respiratory Health Questionnaire,^31^ Positive and Negative Affect Scales,^32^ Smoking Stages of Change score,^33^ and Questionnaire of Vaping Craving,^34^ will be reported separately.

### Statistical Analysis

Analysis of covariance was used for the week 16 mean total, study, and nonstudy CPD, adjusting for baseline CPD. Additional covariates included age, sex, and population. The CPD and secondary outcomes were analyzed over time using linear mixed models (repeated-measures analysis of variance), with the random effects of participant and repeated factor of time point. Outcomes were log transformed or square root transformed as appropriate, with least square (LS) means back transformed for interpretability. Initial models included all 2-way or 3-way interactions of experimental condition, study population, and time point, which were removed if not significant, with 2-sided α at *P* < .05. Significant effects were followed with pairwise comparisons using Tukey-Kramer correction.

Participants who did not complete the study were excluded in analyses of covariance models but included in linear mixed models by using SAS proc mixed (SAS Institute Inc), which handles missing data by providing estimates using the maximum-likelihood method. Sensitivity analyses were conducted using multiple imputation for analysis of covariance models, all of which produced similar results. Days abstinent were analyzed using 0-inflated negative binomial regression. Supplement 1 provides additional details. All analyses were conducted from December 2023 to July 2024 using SAS statistical software, version 9.4.^35^

## Results

Main effects and interactions involving the 4 experimental conditions (NNC, VLNC, VLNC + TF, and VLNC + PF) are described. The effects involving only the study population or time point are reported in eFigures 1 to 4 in Supplement 2. A total of 326 adults participated (mean [SD] age, 40.09 [10.79] years; 243 females [74.5%], owing to 1 trial including only females, and 83 males [25.5%]) (Table 1). Participants had a mean (SD) CPD of 17.40 (8.87) at study intake. Of the participants, 83 were randomly assigned to NNC, 85 to VLNC, 74 to VLNC + TF, and 84 to VLNC + PF conditions. Most participants completed the study (260 [79.8%]). Only 1 baseline characteristic (FTND total scores) differentiated those who completed from those who withdrew (mean [SD] score, 4.97 [2.23] vs 5.59 [2.07]; *P* = .04) (eTable 1 in Supplement 2).

**Table 1.** Participant Characteristics

Characteristic	Participants by Conditions, No. (%)				
	Overall (n = 326)	NNC cigarettes only (n = 83)	VLNC cigarettes only (n = 85)	VLNC cigarettes + TF (n = 74)	VLNC cigarettes + PF (n = 84)
Population					
Females with lower educational level	80 (24.5)	20 (24.1)	23 (27.1)	16 (21.6)	21 (25.0)
OUD	74 (22.7)	19 (22.9)	19 (22.4)	17 (23.0)	19 (22.6)
Affective disorders	172 (52.8)	44 (53.0)	43 (50.6)	41 (55.4)	44 (52.4)
Age, mean (SD), y	40.09 (10.79)	39.77 (10.98)	39.85 (9.28)	41.51 (12.39)	39.38 (10.59)
Sex					
Female	243 (74.5)	65 (78.3)	69 (81.2)	50 (67.6)	59 (70.2)
Male	83 (25.5)	18 (21.7)	16 (18.8)	24 (32.4)	25 (29.8)
Race and ethnicity^a^					
Black, non-Latino	33 (10.1)	9 (10.8)	6 (7.1)	9 (12.2)	9 (10.7)
Latino	12 (3.7)	4 (4.8)	3 (3.5)	3 (4.1)	2 (2.4)
White, non-Latino	262 (80.4)	66 (79.5)	68 (80.0)	60 (81.1)	68 (81.0)
Non-Latino other or multiracial^b^	19 (5.8)	4 (4.8)	8 (9.4)	2 (2.7)	5 (6.0)
Educational level					
<High school	30 (9.2)	9 (10.8)	4 (4.7)	3 (4.1)	14 (16.7)
High school graduate, equivalency, or some college	201 (61.7)	51 (61.5)	57 (67.1)	46 (62.2)	47 (56.0)
Associate’s degree	43 (13.2)	8 (9.6)	11 (12.9)	13 (17.6)	11 (13.1)
≥College graduate	52 (16.0)	15 (18.1)	13 (15.3)	12 (16.2)	12 (14.3)
Marital status					
Married	51 (15.6)	8 (9.6)	15 (17.7)	17 (23.0)	11 (13.1)
Never married	180 (55.2)	49 (59.0)	47 (55.3)	36 (48.7)	48 (57.1)
Divorced, separated, or widowed	95 (29.1)	26 (31.3)	23 (27.1)	21 (28.4)	25 (29.8)
Primarily smokes mentholated cigarettes	131 (40.2)	32 (38.55)	35 (41.18)	31 (41.89)	33 (39.3)
Cigarettes smoked per day, mean (SD)	17.40 (8.87)	16.42 (8.02)	18.44 (9.47)	17.86 (9.11)	16.90 (8.85)
Urine cotinine level, mean (SD), ng/mL	5043.55 (3680.12)	4499.25 (3120.20)	5235.67 (3251.28)	5156.57 (4150.44)	5293.33 (3799.45)
Breath CO level, mean (SD)	20.46 (13.28)	20.90 (14.02)	19.30 (12.39)	21.03 (13.38)	20.66 (13.44)
Age started smoking regularly, mean (SD)	16.39 (4.26)	16.61 (3.97)	15.92 (3.91)	16.24 (3.84)	16.79 (5.15)
FTND score, mean (SD)	5.09 (2.21)	5.12 (2.24)	5.31 (2.20)	5.26 (2.31)	4.70 (2.07)
Used other tobacco products, past 30 d	23 (7.1)	5 (6.0)	4 (4.7)	5 (6.8)	9 (10.7)
Used e-cigarettes, past 30 d	42 (12.9)	13 (15.7)	11 (12.9)	5 (6.8)	13 (15.5)
E-cigarette flavors, past 30 d					
Fruit medley	23 (7.1)	8 (9.6)	6 (7.1)	3 (4.1)	6 (7.1)
Menthol or mint	12 (3.7)	4 (4.8)	3 (3.5)	2 (2.7)	3 (3.6)
Candy or other sweets	4 (1.2)	1 (1.2)	3 (3.5)	0	0
Other	1 (0.3)	0	0	0	1 (1.2)

Abbreviations: CO, carbon monoxide; FTND, Fagerström Test for Nicotine Dependence; NNC, normal nicotine content (15.8 mg nicotine/g tobacco); OUD, opioid use disorder; PF, e-cigarettes in preferred flavor (participants selected 3 from 8 flavors); TF, e-cigarettes in tobacco flavor; VLNC, very low nicotine content (0.4 mg nicotine/g tobacco).

SI conversion factor: To convert cotinine to nanomoles per liter, multiply by 5.675.

^a^ Participant endorsed race and ethnicity. Race and ethnicity were assessed as part of characterizing the diversity of the sample.

^b^ Non-Latino other included American Indian or Alaska Native and Asian.

### Primary Outcomes

**Figure 2.**
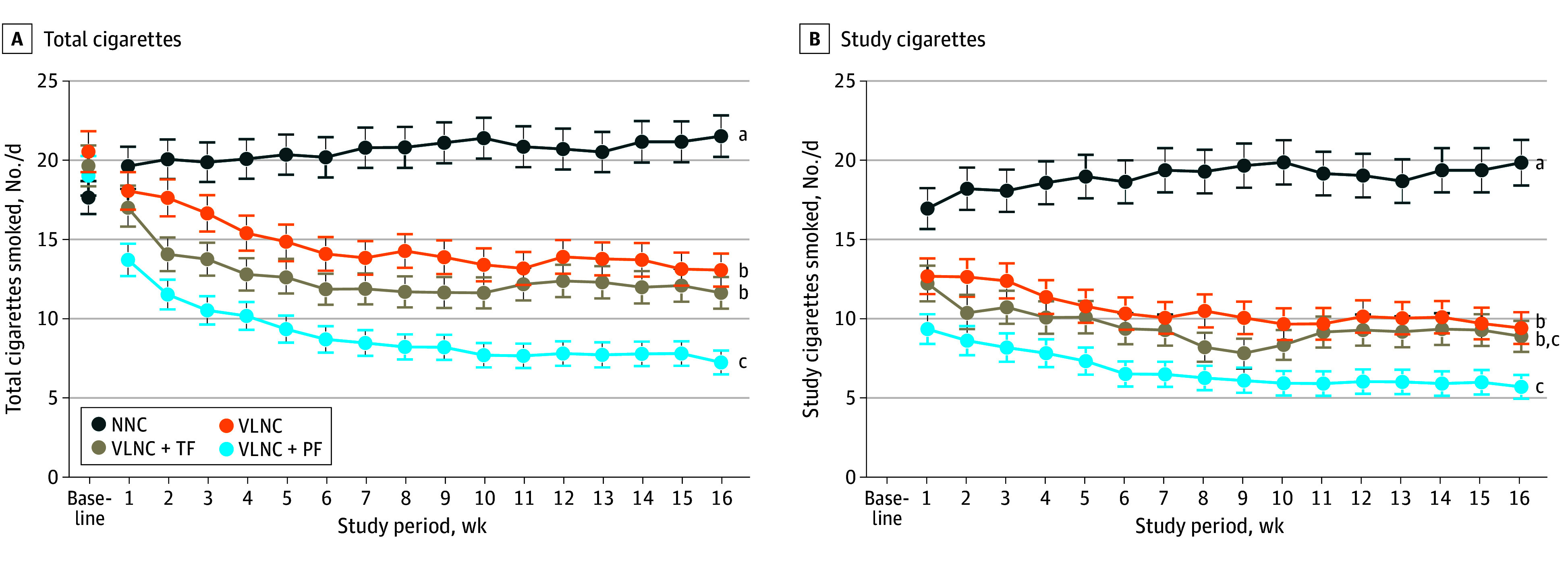
Mean Number of Total Study and Nonstudy Cigarettes Smoked per Day and Study Cigarettes Smoked per Day Collapsed Across Participants and Populations for Each of the 4 Experimental Conditions Data points are least square means from repeated-measures analysis of variance. The four experimental conditions included normal nicotine content (NNC) cigarettes only, very low nicotine content (VLNC) cigarettes only, VLNC cigarettes plus tobacco flavor e-cigarettes (VLNC + TF), and VLNC plus participant-preferred flavor e-cigarettes (VLNC + PF). Error bars indicate SEMs. Data points not sharing a superscript letter (a,b,c) differed significantly in post-hoc testing at week 16 (primary study outcome).

All of the conditions had an effect on week 16 mean total CPD that was consistent across populations (Figure 2A; eFigures 5 and 6 in Supplement 2 provide results by population), with LS means (SEMs) of 22.54 (1.59) in the NNC, 14.32 (1.32) in the VLNC, 11.76 (1.18) in the VLNC + TF, and 7.63 (0.90) in the VLNC + PF conditions. Adjusted mean differences (AMDs) vs NNC were −8.21 (95% CI, −12.27 to −4.16; *P* < .001) in the VLNC, −10.78 (95% CI, −14.67 to −6.90; *P* < .001) in the VLNC + TF, and −14.91 (95% CI, −18.49 to −11.33; *P* < .001) in the VLNC + PF conditions. The CPD rates were decreased more in the VLNC + PF condition vs the VLNC and VLNC + TF conditions (AMDs, −6.70 [95% CI, −9.84 to −3.55; *P* < .001] and −4.13 [95% CI, −7.05 to −1.21; *P* = .02]). The CPD rates between VLNC and VLNC + TF did not differ. When total CPD results were analyzed across study weeks, the magnitude of differences between experimental conditions increased across weeks (Figure 2A). For example, the AMD in the VLNC + PF vs NNC conditions at week 1 was −5.94 (95% CI, −9.08 to −2.79; *P* = .09), while the AMD by week 16 between VLNC + PF and NNC conditions was −14.30 (95% CI, −17.29 to −11.31; *P* < .001).

Imputed results aligned closely with these results when comparing the VLNC (AMDs, −7.79; 95% CI, −11.94 to −3.63; *P* < .001), VLNC + TF (AMDs, −10.23; 95% CI, −14.32 to −6.14; *P* < .001), and VLNC + PF (AMDs, −14.77; 95% CI, −18.50 to −11.04; *P* < .001) conditions with NNC at week 16. Additionally, the results aligned when comparing VLNC + PF with VLNC (AMD, −6.99; 95% CI, −10.13 to −3.85; *P* < .001) and VLNC + TF (AMD, −4.54; 95% CI, −7.59 to −1.49; *P* = .001).

The 3 e-cigarette flavors that participants in the VLNC + PF condition most frequently selected for use during week 16 were mango (47 [24.9%]), fruit medley (37 [19.6%]), and classic menthol (23 [12.2%]), with selections for the remaining flavors ranging from 4.8% to 10.6% (eTable 2 in Supplement 2). Across the 8 flavor options, 53.4% were for fruity and sweet flavors (mango, fruit medley, and crème brûlée). Flavor preferences across the 16-week study were similar to those in week 16 (eTable 2 in Supplement 2).

### Secondary Outcomes

#### Study and Nonstudy CPD

Each of the VLNC conditions significantly decreased week 16 CPD vs NNC across populations (Figure 2B, Table 2); VLNC + PF also had decreased CPD vs VLNC, although not VLNC + TF. In analyses across study weeks, differences between experimental conditions increased significantly across weeks.

**Table 2.** Adjusted Mean Differences for Secondary Outcomes

Outcome	Condition Comparison	AMD (95% CI)	*P* value	Outcome	Condition Comparison	AMD (95% CI)	*P* value
Wk 16 study CPD	VLNC + PF vs NNC	−15.13 (−18.83 to −11.42)	<.001	Days abstinent	VLNC + PF vs NNC	22.61 (14.89 to 30.33)	<.001
	VLNC + TF vs NNC	−12.58 (−16.51 to −8.65)	<.001		VLNC + TF vs NNC	8.31 (3.02 to 13.59)	.004
	VLNC vs NNC	−10.34 (−14.44 to −6.25)	<.001		VLNC vs NNC	2.18 (−0.09 to 4.46)	.18
	VLNC + PF vs VLNC	−4.78 (−7.80 to −1.77)	.004		VLNC + PF vs VLNC	20.43 (12.43 to 28.43)	.002
	VLNC + PF vs VLNC + TF	−2.55 (−5.33 to 0.24)	.23		VLNC + PF vs VLNC + TF	14.30 (4.99 to 23.62)	.047
	VLNC + TF vs VLNC	−2.24 (−5.52 to 1.05)	.47		VLNC + TF vs VLNC	6.12 (0.44 to 11.81)	.60
Wk 16 nonstudy CPD	VLNC + PF vs NNC	0.20 (−0.23 to 0.63)	.76	Wk 16 B–WISDM PDM	VLNC + PF vs NNC	−2.64 (−4.35 to −0.93)	.007
	VLNC + TF vs NNC	0.88 (0.29 to 1.46)	.005		VLNC + TF vs NNC	−1.08 (−2.87 to 0.71)	.59
	VLNC vs NNC	0.86 (0.28 to 1.44)	.005		VLNC vs NNC	−1.20 (−2.95 to 0.55)	.46
	VLNC + PF vs VLNC	−0.66 (−1.26 to −0.06)	.08		VLNC + PF vs VLNC	−1.44 (−3.15 to 0.27)	.30
	VLNC + PF vs VLNC + TF	−0.68 (−1.28 to −0.07)	.08		VLNC + PF vs VLNC + TF	−1.56 (−3.31 to 0.19)	.26
	VLNC + TF vs VLNC	0.02 (−0.70 to 0.73)	.96		VLNC + TF vs VLNC	0.12 (−1.68 to 1.92)	.99
Wk 16 breath CO	VLNC + PF vs NNC	−8.86 (−14.22 to −3.49)	.001	Wk 16 QSU-Brief Factor 1	VLNC + PF vs NNC	−1.77 (−2.51 to −1.02)	<.001
	VLNC + TF vs NNC	−4.43 (−10.41 to 1.56)	.36		VLNC + TF vs NNC	−1.40 (−2.19 to −0.61)	<.001
	VLNC vs NNC	−1.07 (−7.20 to 5.07)	.98		VLNC vs NNC	−0.71 (−1.46 to 0.05)	.07
	VLNC + PF vs VLNC	−7.79 (−13.07 to −2.52)	.006		VLNC + PF vs VLNC	−1.06 (−1.18 to -.0.31)	.002
	VLNC + PF vs VLNC + TF	−4.43 (−9.53 to 0.67)	.23		VLNC + PF vs VLNC + TF	−0.37 (−1.15 to 0.41)	.61
	VLNC + TF vs VLNC	−3.36 (−9.27 to 2.54)	.59		VLNC + TF vs VLNC	−0.69 (−1.48 to 0.10)	.11
Wk 16 NNAL	VLNC + PF vs NNC	−0.94 (−1.41 to −0.47)	<.001	QSU-Brief Factor 1 across wks	VLNC + PF vs NNC	−1.32 (−1.96 to −0.68)	<.001
	VLNC + TF vs NNC	−0.46 (−1.01 to 0.09)	.30		VLNC + TF vs NNC	−0.88 (−1.54 to −0.22)	.004
	VLNC vs NNC	−0.47 (−1.01 to 0.06)	.21		VLNC vs NNC	−0.56 (−1.20 to 0.08)	.11
	VLNC + PF vs VLNC	−0.46 (−0.83 to −0.10)	.03		VLNC + PF vs VLNC	−0.76 (−1.40 to −0.12)	.01
	VLNC + PF vs VLNC + TF	−0.47 (−0.87 to −0.08)	.04		VLNC + PF vs VLNC + TF	−0.44 (−1.10 to 0.22)	.31
	VLNC + TF vs VLNC	0.01 (−0.46 to 0.48)	.99		VLNC + TF vs VLNC	−0.33 (−0.99 to 0.34)	.58
NNAL across wks	VLNC + PF vs NNC	−1.13 (−1.57 to −0.70)	<.001	Wk 16 QSU-Brief Factor 2	VLNC + PF vs NNC	−1.24 (−1.87 to −0.61)	<.001
	VLNC + TF vs NNC	−0.72 (−1.20 to −0.23)	.007		VLNC + TF vs NNC	−0.75 (−1.42 to −0.08)	.02
	VLNC vs NNC	−0.66 (−1.15 to −0.18)	.01		VLNC vs NNC	−0.45 (−1.09 to 0.19)	.26
	VLNC + PF vs VLNC	−0.47 (−0.76 to −0.17)	.002		VLNC + PF vs VLNC	−0.79 (−1.42 to −0.15)	.008
	VLNC + PF vs VLNC + TF	−0.41 (−0.71 to −0.12)	.01		VLNC + PF vs VLNC + TF	−0.49 (−1.15 to 0.18)	.23
	VLNC + TF vs VLNC	−0.05 (−0.42 to 0.31)	.99		VLNC + TF vs VLNC	−0.30 (−0.97 to 0.37)	.65
Wk 16 anatabine	VLNC + PF vs NNC	−6.23 (−10.26 to −2.21)	.003	QSU-Brief Factor 2 across wks	VLNC + PF vs NNC	−0.88 (−1.44 to −0.32)	<.001
	VLNC + TF vs NNC	−2.70 (−7.54 to 2.15)	.65		VLNC + TF vs NNC	−0.39 (−0.98 to 0.19)	.30
	VLNC vs NNC	−3.30 (−7.89 to 1.29)	.42		VLNC vs NNC	−0.31 (−0.87 to 0.26)	.50
	VLNC + PF vs VLNC	−2.93 (−6.40 to 0.53)	.27		VLNC + PF vs VLNC	−0.57 (−1.13 to −0.01)	.045
	VLNC + PF vs VLNC + TF	−3.54 (−7.34 to 0.26)	.18		VLNC + PF vs VLNC + TF	−0.48 (−1.06 to 0.09)	.14
	VLNC + TF vs VLNC	0.60 (−3.79 to 5.00)	.99		VLNC + TF vs VLNC	−0.09 (−0.67 to 0.50)	.98
Anatabine across wks	VLNC + PF vs NNC	−6.25 (−9.56 to −2.95)	<.001	NA	NA	NA	NA
	VLNC + TF vs NNC	−4.07 (−7.76 to −0.38)	.09		NA	NA	NA
	VLNC vs NNC	−5.46 (−8.86 to −2.06)	.001		NA	NA	NA
	VLNC + PF vs VLNC	−0.80 (−2.96 to 1.37)	.87		NA	NA	NA
	VLNC + PF vs VLNC + TF	−2.19 (−4.78 to 0.40)	.27		NA	NA	NA
	VLNC + TF vs VLNC	1.39 (−1.32 to 4.10)	.69		NA	NA	NA

Abbreviations: AMD, adjusted mean difference; B–WISDM PDM, Brief–Wisconsin Inventory of Smoking Dependence Motives Primary Dependence Motives; CPD, cigarettes smoked per day; NA, not applicable; NNAL, urine 4-(methylnitrosamino)-1-(3-pyridyl)-1-butanol; NNC, normal nicotine content (15.8 mg/g tobacco); PF, e-cigarettes in preferred flavor (participants selected 3 from 8 flavors); QSU-Brief, Questionnaire on Smoking Urges–Brief; TF, e-cigarettes in tobacco flavor; VLNC, very low nicotine content (0.4 mg/g tobacco).

Use of nonstudy cigarettes in week 16 was increased significantly above NNC levels in the VLNC and VLNC + TR conditions across populations, although not in the VLNC + PF condition (eFigure 7 in Supplement 2; Table 2). In analyses across weeks, only CPD in the VLNC condition were increased above NNC levels.

#### Biomarkers of Exposure

**Figure 3.**
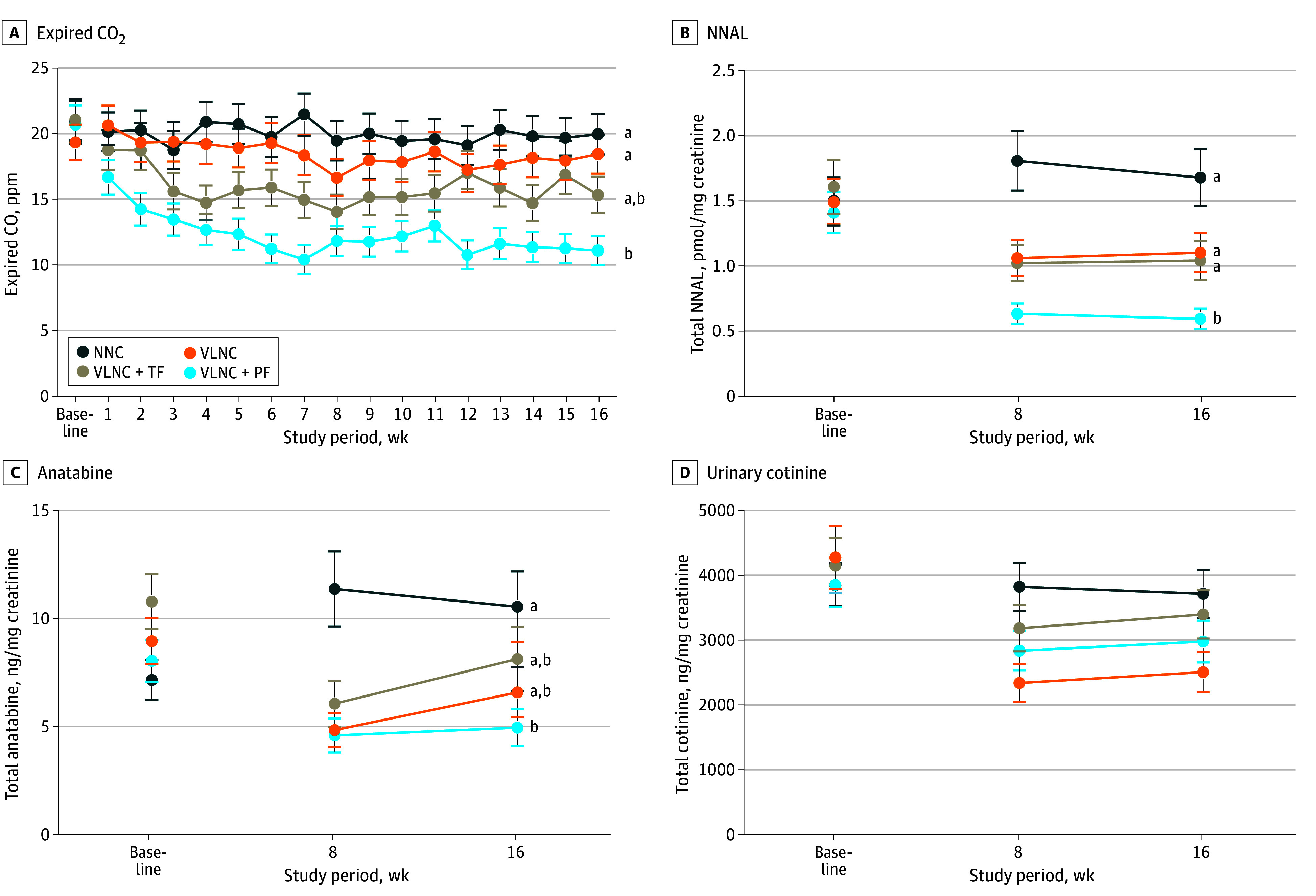
Mean Expired Breath Carbon Monoxide (CO), Urine 4-(Methylnitrosamino)-1-(3-Pyridyl)-1-Butanol (NNAL), Urine Anatabine, and Urine Cotinine Levels Collapsed Across Participants and Populations for Each of the 4 Experimental Conditions Data points are least square means from repeated-measures analysis of variance. The 4 experimental conditions included normal nicotine content (NNC) cigarettes only, very low nicotine content (VLNC) cigarettes only, VLNC cigarettes plus tobacco flavor e-cigarettes (VLNC + TF), and VLNC plus participant-preferred flavor e-cigarettes (VLNC + PF). Error bars indicate SEMs. Data points not sharing a superscript letter (a,b) differed significantly in post-hoc testing at week 16. There were no significant differences in urinary cotinine level among the conditions.

Experimental condition had effects during week 16 and across study weeks on breath CO, urine NNAL, and urine anatabine levels that were consistent across populations (Figure 3A-C; Table 2). There were no effects of experimental condition on urine cotinine (Figure 3D). Specifically, week 16 breath CO levels were lower in the VLNC + PF vs NNC and VLNC conditions. In analyses across study weeks, the magnitude of these differences between study conditions increased. The week 16 urine NNAL levels in the VLNC + PF condition were lower than the NNC (AMD, −0.94; 95% CI, −1.41 to −0.47; *P* < .001), VLNC (AMD, −0.47; 95% CI, −0.87 to −0.08; *P* = .03), and VLNC + TF (AMD, −0.46; 95% CI, −0.83 to −0.10; *P* = .04) levels; in analyses across weeks 8 and 16, NNAL levels in the VLNC and VLNC + TF conditions were also lower than in the NNC condition. Week 16 urine anatabine levels were lower in the VLNC + PF vs NNC; when analyzed across weeks 8 and 16, VLNC levels were also lower than NNC levels.

### Abstinence Outcomes

The VLNC + PF condition had a significant effect on the mean number of days without smoking during the 16-week study across populations (Table 2; eFigure 8 in Supplement 2), with significantly more abstinent days in the VLNC + PF vs the NNC, VLNC, and VLNC + TF conditions. Mean days abstinent in the VLNC + TF condition was also greater than in NNC, although not in VLNC; VLNC did not differ significantly from NNC. There were no significant differences by condition on post-trial 24-hour abstinence test results or 30-day smoking cessation rates.

### Dependence Severity

Across populations, the VLNC + PF condition had a significant effect on week 16 B-WISDM Primary Dependence Motives scores, with lower scores in VLNC + PF vs NNC (Table 2; eFigure 9A in Supplement 2). The experimental conditions had no significant effects on these scores across weeks or on Secondary Dependence Motives scores during week 16 or across weeks. There were also no effects on FTND total scores during week 16 or across weeks.

### Craving and Withdrawal

Across populations, the VLNC + PF and VLNC + TF conditions had significant effects on week 16 QSU-Brief Factor 1 and Factor 2 craving for study cigarettes (eFigure 9B and C in Supplement 2). Factor 1 LS mean craving was lower in the VLNC + PF and VLNC + TF vs NNC conditions. Craving in the VLNC + PF condition was also below that in the VLNC condition; the VLNC + TF did not differ significantly from the VLNC condition. Factor 2 craving was lower in the VLNC + PF and VLNC + TF vs NNC conditions. Craving in the VLNC + PF condition was also below that in the VLNC condition but not in the VLNC + TF condition.

In analyses across weeks, Factor 1 craving in the VLNC + PF and VLNC + TF conditions was significantly lower than NNC craving; VLNC + PF craving was also lower than that in the VLNC condition, while VLNC + TF craving was not. Effects on Factor 2 craving across populations was limited to the VLNC + PF level being lower than the NNC and VLNC levels.

None of the experimental conditions had a significant effect on the QSU-Brief craving for usual-brand cigarettes or the Minnesota Nicotine Withdrawal Scale total or desire-to-smoke scores at week 16 or across weeks.

### Adverse Effects

Most participants reported at least 1 AE (284 of 326 [87.1%]) (eTables 3-4 in Supplement 2). There were no significant differences between experimental conditions in incidence, total number, or number of serious or severe AEs (eTables 3-6 in Supplement 2).

## Discussion

Results from these 3 RCTs support the primary hypothesis that access to e-cigarettes in preferred flavors, including the most often selected fruity and sweet flavors, enhances the reductions in total CPD achieved by using VLNC cigarettes alone. The evidence also demonstrates that this product combination protected against the small increases in use of nonstudy cigarettes in the VLNC and VLNC + TF conditions, while also enhancing the effects of VLNC cigarettes on other clinically important outcomes such as NNAL exposure, a carcinogen biomarker of tobacco use,^36^ days without smoking, dependence severity, and craving. Additionally, no significant differences in AEs were noted between those assigned to flavored e-cigarettes vs the other conditions There is well-established evidence that smoking rate and dependence severity are associated with success in quitting smoking, that toxicant exposure from combusted tobacco is the primary cause of the adverse health effects of smoking, and craving is a common factor associated with smoking relapse.^37,38,39,40^ Observing these outcomes across 3 at-risk populations is important as they are among the most difficult populations to reach with tobacco control and regulatory interventions.^1,2,3,4,5,6^ The present results are consistent with recent results from an RCT in adults who smoke from the general population where access to a variety of noncombusted alternatives to smoking enhanced the effects of VLNC cigarettes on a comparable battery of outcomes.^19^

Any population health benefits that adults may accrue from having access to preferred e-cigarette flavors, as suggested by these results, must be balanced against potential adverse health effects if such access also increases e-cigarette use in youths. Ideally, a well-regulated marketplace with strong enforcement would allow adult access while preventing youth access.^15^ Whether that balance is achievable in the US marketplace remains unclear, but new evidence such as that reported here and elsewhere^19,41,42,43^ provides a reason to continue exploring that possibility and suggests that doing so can enhance the overall effect of a nicotine product standard on cigarette smoking should the FDA move forward with that policy.^7^

### Limitations

Study limitations include the effect of the COVID-19 pandemic on participant recruitment, which yielded a smaller than planned sample. There was also a 20% dropout rate, which is within the level deemed to be low risk for bias in tobacco clinical trials, when direction of results is robust to imputation methods as was the case in the present study,^44^ but nevertheless may have introduced bias. The smaller sample size likely limited the ability to discern some effects of VLNC cigarettes alone, such as decreases in dependence severity.^13,45^ However, even in the absence of e-cigarettes, VLNC cigarettes significantly reduced the smoking rates compared with NNC cigarettes across these 3 high-risk populations, as reported previously.^13^ The use of paid volunteers who were not selected to be representative of the US population may limit generalizability. While the magnitude of effects of access to preferred e-cigarettes increased over time, study duration was limited to 16 weeks, leaving unanswered the effects of longer-term exposure, such as risk for chronic dual use. Moreover, while there is sound evidence that e-cigarettes are safer than combusted cigarettes, they are not without risk and long-term effects are unavailable.^46^ While the VLNC + PF condition produced the greatest number of days without smoking during the 16-week study, there were no significant differences between conditions in the post-trial 24-hour abstinence test or smoking cessation rates at 30-day post-trial follow-up. Perhaps that is not surprising considering that the RCTs limited inclusion to those who were not planning to quit smoking, but it underscores a need for future trials on this topic involving those planning to quit smoking.

## Conclusions

Results of 3 RCTs provide compelling evidence that the effects on smoking of reducing nicotine content of cigarettes to minimally addictive levels could be enhanced if adults have access to e-cigarettes in commonly preferred flavors. This finding applies to populations at high risk for smoking, addiction, and smoking-related adverse health outcomes.

## Notes

Supplement 1.
Trial Protocol

Supplement 2. **eFigure 1.** Number of Total Cigarettes Smoked per Day According to Study Population **eFigure 2.** Biomarkers of Exposure According to Study Population **eFigure 3.** Dependence Severity According to Study Population **eFigure 4.** Craving and Withdrawal **eFigure 5.** Number of Total Cigarettes Smoked per Day Over Time According to Condition, Across Populations, and According to Population **eFigure 6.** Number of Total Cigarettes Smoked per Day at Week 16 According to Condition, Across Populations, and According to Study Population **eFigure 7.** Number of Non-Study Cigarettes Smoked per Day According to Condition **eFigure 8.** Cigarette Abstinence According to Condition **eFigure 9.** Nicotine Dependence Severity and Cigarette Craving According to Condition **eTable 1.** Participant Characteristics **eTable 2.** e-Cigarette Flavors Selected (VLNC+PF Condition) **eTable 3.** Total Number of Adverse Events **eTable 4.** Number of Participants With Any Adverse Event **eTable 5.** Count of Serious and Severe Adverse Events **eTable 6.** Description of Serious and Severe Adverse Events

Supplement 3.
Data Sharing Statement
