# Supplementary material for: Reduced Nicotine Cigarettes and E-Cigarettes in High-Risk Populations: 3 Randomized Clinical Trials
Source: JAMA Netw Open. 2024 Sep 6;7(9):e2431731. doi: 10.1001/jamanetworkopen.2024.31731 (PMC11380105; doi:10.1001/jamanetworkopen.2024.31731)
Supplement: Supplement 1. — Trial Protocol [file jamanetwopen-e2431731-s001.pdf]

eSupplement 1

eSupplement 1 was prepared by the authors to provide interested readers additional protocol and statistical-analysis details on the three randomized, controlled trials reported in Higgins ST, Sigmon SC, Tidey JW, et al. *Reduced Nicotine Cigarettes and E-Cigarettes in High-Risk Populations: 3 Randomized Clinical Trials*

Protocols for each of the three trials are detailed separately below followed by the Statistical Analysis Plan for those trials at the end of each protocol.

The protocol information provided below is what local Institutional Review Boards at each research site approved.

Summary of Protocol Modifications:

10-2-20 Two modifications were made across the three trials. (1) We stipulated that individuals must have access to the technology necessary to complete the initial study eligibility assessment. (2) Deleted mention of a debit card and an e-cigarette sampling session and associated questionnaire which were carried over from the original in-person protocol.

10-12-20 Made three protocol modifications across the three trials. (1) Moved to salivary rather that urine drug toxicology testing to allow observed specimen collection. Urine specimens were retained as a back-up should someone be able to provide adequate saliva for valid testing. (2) Stipulated that should a participant fail to provide a first void urine specimen, arrangements to get that specimen on another day will be made rather than substituting a spot urine specimen. (3) Added an e-cigarette flavor rating questionnaire.

11-30-20 Modified curbside assessments to include a lab option for inclement weather.

2-2-21 Modified protocols to allow increases in weekly supply of e-liquid pods if a participant consistently uses more that their baseline determined amount.

3-17-21 Substituted breath CO for a urine specimen to confirm abstinence in the assessments completed 30 days after trial completion.

5-11-21 Increased maximal participant compensation to more equitably compensate participants for the considerable time commitement the trials require.

6-27-22 (1) Provided greater detail to the protocols regarding study policy on contraception use. (2) Updated the Data Safety Monitoring Board roster.

1-23-23 Increased maximal participant compensation to include bonuses for every two consecutive study visits completed on schedule.

Reduced Nicotine Cigarettes and E-Cigarettes in High-Risk Populations: 3 Randomized Clinical Trials

Stephen T. Higgins, PhD[1]*, Stacey C. Sigmon, PhD[1], Jennifer W. Tidey, PhD[2], Sarah H. Heil, PhD[1], Diann E. Gaalema, PhD[1], Dustin C. Lee, PhD[3], Michael J. DeSarno, MS[1], Elias M. Klemperer, PhD[1], Katherine E. Menson, DO[1], Patricia A. Cioe, PhD[2], Shirley Plucinski, MPH[1], Rhiannon C. Wiley, MA[1], Eva Orr, MPH[1]

[1] UVM Tobacco Center of Regulatory Science, University of Vermont, Burlington, VT
[2] Center for Alcohol and Addiction Studies, Brown University, Providence, RI
[3] Behavioral Pharmacology Research Unit, Johns Hopkins University School of Medicine, Baltimore, MD

eSupplement 1

Table of Contents

Content	Page Number

eProtocol: Smokers with Affective Disorders 4

eProtocol: Smokers with Opioid Use Disorder 47

eProtocol: Smokers with Socioeconomic Disadvantage (women of reproductive age) .. 95

STUDY PROTOCOL: SMOKERS WITH AFFECTIVE DISORDERS

Table of Contents

1. OBJECTIVE 7
2. SIGNIFICANCE 8
   2.1 Affective Disorders (AD) and Smoking 8
   2.2 Relevance of the Project to the Integrative Theme and Goals of the TCORS 9
   2.3 Relevance to the Scientific Domains and Priorities of the FDA CTP 9
   2.4 How Study Outcomes Will Improve Scientific Knowledge Related to the Manufacture, Distribution, and Marketing of Tobacco Products 9
3. RATIONALE 9
   3.1 Effects of Nicotine Reduction in Smokers With AD 9
   3.2 E-Cig Use 11
   3.3 E-Cig Effects on Smoking 11
   3.4 Mechanisms of E-Cig Effects 11
   3.5 Importance of E-Cig Flavors 12
   3.6 Products to be Tested 12
   3.7 Summary 12
4. PROJECT STUDY METHODS 13
5. STUDY SCREENING PROCEDURES 13
   5.1 Participants 13
   5.2 Recruitment 13
   5.3 Informed Consent Process 15
   5.4 Screening Measures 15
   5.5 Suicidality/Mental Health Monitoring 17
   5.6 Inclusion/Exclusion Criteria 17
   5.7 Eligibility Determination 19
6. STUDY BASELINE PROCEDURES 20
   6.1 Visit Scheduling Requirements for Baseline Period 21
   6.2 Measures/Assessments 21
   6.3 E-Cigarette Training Sessions (Baseline 2) 23
   6.4 Interactive Voice Response System 23
7. STUDY EXPERIMENTAL PROCEDURES 24
   7.1 Experimental Period 24
   7.2 Experimental Visits Weeks 1, 3, 5, 7, 9, 11, 13, and 15 Procedures 25
      7.2.A. Measures/Assessments 25
   7.3 Experimental Visits Weeks 2, 4, 6, 8, 10, 12, 14, and 16 Procedures 25
      7.3.A. Measures/Assessments 25

7.4 Interactive Voice Response System 26
7.5 Variable Incentive Program 27
7.6 Product and Procedures Compliance Review Sessions 27
7.7 Quit Attempts During the Study Protocol 28
7.7.A. If a participant is currently abstaining from smoking with the intention to quit 28
7.7.B. If a participant is planning to quit smoking, but has not initiated the quit attempt 28
7.8 Abstinence Assessment Session 29
7.8.A. Participants Who Meet Criteria for Abstinence 29
7.8.A.1. Measures/Assessments 29
7.8.B. Participants Who Do Not meet Criteria for Abstinence 29
7.8.B.1. Measures/Assessments 30
7.9 Participant Compensation 30
7.10 End of Study 30
7.11 30-day Follow Up Phone Call 31
8. STUDY RANDOMIZATION 31
8.1 Randomization Process 31
8.2 Study Product Administration 31
8.3 Guidelines for Reporting Other Nicotine Product Use 32
8.4 Study Product Accountability 32
9. STUDY 3 STATISTICAL METHODS AND SAMPLE SIZE 32
9.1 Statistical Methods 32
9.2 Sample Size 33
10. POTENTIAL RISKS OF PARTICIPATION 34
10.1 Risks of Participation 34
10.2 Avoiding Risk to Fetus 35
10.3 Expected Benefits of Participation 36
11. PROTECTION AGAINST RISK 36
11.1 Data Collection Protections 36
11.2 Data Storage 37
12. ADVERSE EVENTS 37
13. WITHDRAWAL OR MONITORING OF PARTICIPANTS 37
14. DATA SAFETY MONITORING BOARD 38
15. INVESTIGATIONAL TOBACCO PRODUCT 39
16. CERTIFICATE OF CONFIDENTIALITY 39
17. OUTCOME VARIABLES 40
18. REFERENCES 41

Abbreviations

- 3HC: 3-hydroxycotinine
- BAL: Breath alcohol levels
- BDI: Beck's Depression Inventory
- BMI: Body Mass Index
- BP: Blood pressure
- BPM: Beats per minute
- BRIEF-A: Behavioral Rating Inventory of Executive Function
- CES: Cigarette Evaluation Scale
- CO: Carbon monoxide
- COT: Cotinine
- CPD: Cigarettes per day
- CPT: Cigarette Purchase Task
- CPT: Continuous Performance Task
- DAST: Drug Abuse Screening Test
- DDT: Delay Discounting Task
- D-KEFS: Delis-Kaplan Executive Function System
- EDC: Electronic Data Capture
- EQ-5D: Euro-Qol
- FSPTCA: Family Smoking Prevention and Tobacco Control Act
- FTND: Fagerström Test for Nicotine Dependence
- GAD: Generalized Anxiety Disorder
- HR: Heart rate
- IVR: Interactive Voice Response
- MDD: Major Depressive Disorder
- MINI: Mini International Neuropsychiatric Interview
- MNWS: Minnesota Nicotine Withdrawal Scale
- NMR: Nicotine metabolite ratio
- NNAL: 4-(methylnitrosamino)-1-(3-pyridyl)-1-butanol
- NNC: Normal nicotine content
- NNN: *N′*-nitrosonornicotine
- OASIS: Overall Anxiety Severity and Impairment Scale
- OUD: Opioid Use Disorder
- PANAS: Positive and Negative Affect Schedule
- PHQ: Patient Health Questionnaire
- PSS: Perceived Stress Scale
- QSU: Questionnaire of Smoking Urges
- RNC: Reduced nicotine content
- TLFB: Timeline Follow Back
- VLNC: Very low nicotine content
- WISDM: Wisconsin Index of Smoking Dependence Motives

PROTOCOL

1. OBJECTIVE

While the prevalence of smoking in the US general population has declined over the past 50 years, there has been little to no decline among people with mental health conditions. Affective disorders (ADs) are the most common mental health conditions in the US, and over 40% of people with ADs are current smokers.[1] A national policy of reducing the nicotine content of cigarettes has the potential to reduce tobacco use, dependence, and related adverse health outcomes.[2-4] Controlled trials in psychiatrically stable smokers have shown that extended use of very low nicotine content cigarettes (VLNCCs) results in reductions in cigarettes per day (CPD), cigarette dependence, and tobacco toxicant exposure, with few adverse consequences.[5-8] Furthermore, our research during UVM TCORS Phase 1 funding indicates that smokers with ADs respond to reductions in cigarette nicotine content with reductions in cigarette demand.[9-10] However, tobacco market conditions are likely to exert considerable influence over the extent to which that potential is realized. In particular, we hypothesize that the following conditions are likely to exert considerable influence over the potential effectiveness of a national nicotine reduction policy for cigarettes, particularly in smokers with ADs and other vulnerabilities to tobacco addiction: (1) whether alternative sources of non-combusted nicotine are readily available, and (2) whether these alternatives are available under conditions that optimize their appeal.

The goal of the proposed trial is to experimentally model whether increasing the availability and appeal of an alternative, non-combusted source of nicotine (e-cigarettes) enhances the effects of reduced-nicotine cigarettes in smokers with ADs. Daily smokers with current ADs, recruited at University of Vermont and Brown University, will be randomized to one of the following four conditions: (1) normal nicotine content cigarettes (NNCCs) alone, serving as the control condition, (2) VLNCCs alone, (3) VLNCCs + nicotine e-cigarettes in only tobacco flavors (TF e-cigs), or (4) VLNCCs + nicotine e-cigarettes in preferred flavors (PF e-cigs). Participants will be asked to use only their assigned study products for 16 weeks. Outcome measures include total CPD, cigarette demand assessed by behavioral economics-based purchase tasks, craving, withdrawal, psychiatric symptoms, breath carbon monoxide (CO) and biomarkers of tobacco toxicant exposure. In Week 17, participants will receive incentives to abstain from cigarettes during a three-hour laboratory test session and we will assess the effects of study conditions on cigarette demand, craving and withdrawal.

This research will address the following specific aims: Aim 1 (Primary): Compare the effects of (1) NNCCs alone, (2) VLNCCs alone, (3) VLNCCs + TF e-cigs and = (4) VLNCCs + PF e-cigs on total CPD in smokers with ADs. We hypothesize that at Week 16, total CPD will be reduced in a linear, graded manner (condition 4 > 3 > 2 > 1), with the largest reduction in the VLNCC + PF e-cig condition. Aim 2 (Secondary): Compare the effects of the four study conditions on cigarette demand, psychiatric symptoms, and biomarkers of smoke and tobacco toxicant exposure (CO, NNAL, PAHs) in smokers with ADs. We hypothesize that at Week 16, cigarette demand, CO, and toxicant biomarkers will have decreased in a linear, graded manner, with the largest reduction in the VLNCC + PF e-cig condition. We will characterize effects on psychiatric symptoms but are not testing specific hypotheses on these symptoms. Aim 3 (Exploratory): To explore the effects of the four study conditions on cigarette demand, craving, and withdrawal in smokers with ADs during the abstinence assessment period.

The integrative theme of this TCORS is vulnerable populations. The proposed research is highly relevant to the CTP's scientific domains of Addiction and Behavior because it will address whether reducing the nicotine content of cigarettes reduces cigarette use, dependence, and product appeal, and whether these effects are enhanced by the availability of an appealing alternative source of non-combusted nicotine. It will address the Health Effects domain by assessing the effects of these conditions on tobacco toxicant exposure and a respiratory biomarker. The proposed study is significant and innovative because it will model how

availability and appeal of e-cigs may moderate the effectiveness of a national reduced- nicotine policy for cigarettes in this understudied population. Finally, it is programmatic as it will build upon and extend the work that our team accomplished on VLNCCs in those with ADs during phase 1 of UVM TCORS funding.

2. SIGNIFICANCE

2.1. Affective Disorders (AD) and Smoking

Each year, cigarette smoking kills almost half a million Americans and costs the US almost $300 billion in medical costs and lost productivity.[11] While smoking rates in the general US population have declined over the past 50 years, there has been little to no decline among people with mental health conditions (MHCs), indicating that current tobacco control policies and treatments are not benefiting these smokers.[1,12] Affective disorders (ADs), which include major depressive disorder and anxiety disorders, are the most common MHCs in the US; in 2015, 7% of US adults reported past-year major depressive disorder and 18% reported a past-year anxiety disorder.[13] Over 40% of people with ADs are current smokers.[1] Stated conversely, 22% of nicotine-dependent smokers report having a mood disorder and 23% report an anxiety disorder.[14] Smokers with ADs make as many cessation attempts as those without MHCs, but are more likely to relapse when they try to quit.[15-18] Hence, ADs are associated with disproportionately high rates of tobacco-related disease and death.[19, 20]

Similar transdiagnostic factors appear to underlie the low cessation rates in people with depression and anxiety disorders.[21] Negative mood is a prominent feature of major depression and anxiety disorders[22]. Although long-term cessation is associated with improvements in mood,[23] smoking abstinence produces transient increases in negative mood that are reversed by smoking.[24] Smokers with ADs report more severe effects of abstinence on negative mood than those without MHCs,[25] and have stronger expectancies that smoking will reduce negative mood.[26-28] Anhedonia (reduced capacity to experience pleasure from rewarding stimuli) may also contribute to smoking persistence in people with ADs. Anhedonia is a key symptom of depression and anxiety disorders and is associated with increased responsiveness to pharmacological reinforcers that potently release mesolimbic dopamine (DA).[29] Consistent with this hypothesis, smokers with a history of major depression have greater smoking-induced DA release than those without depression,[30] and overvalue cigarette reinforcement.[31,32] Furthermore, nicotine can enhance the reinforcing effects of environmental stimuli.[33,34] This may contribute to low cessation rates in smokers with ADs, as smokers with depression, but not those without MHCs, report greater enjoyment of activities in their natural environments while smoking.[31]

A national nicotine reduction policy for cigarettes may be an effective regulatory approach to reducing cigarette dependence in smokers with ADs. The 2009 Family Smoking Prevention and Tobacco Control Act gave the Food and Drug Administration (FDA) the authority to regulate tobacco products as appropriate to protect public health, including limiting the nicotine content of cigarettes.[35] An FDA-mandated reduction in the nicotine content of cigarettes to a minimally-addictive level could reduce tobacco reinforcement and dependence.[3,36] This approach could be particularly beneficial to subpopulations of smokers who have less success with currently-available cessation treatments, such as people with ADs. Our work during the UVM TCORS phase 1 funding period, indicates that smokers with ADs, like smokers without MHCs, respond to reductions in the nicotine content of cigarettes with reductions in cigarette demand and other measures of addiction potential. However, tobacco market conditions are likely to exert considerable influence over the extent to which the effects of reduced-nicotine cigarettes are realized in the natural environment. This may be particularly true of smokers with ADs, who are more sensitive to the effects of nicotine withdrawal than those without MHCs.[37] Use of electronic cigarettes (e-cigs) is increasing sharply in the US, and it is important to consider the potential moderating effects of e-cig use on a reduced-nicotine policy for cigarettes. By providing an alternative source of nicotine, we believe that the availability of nicotinized e-cigs, and manipulations that increase their appeal (flavors), will enhance the ability of a national cigarette nicotine-reduction policy to decrease cigarette smoking, dependence and toxin exposure among

smokers with ADs.

2.2. Relevance of the project to the integrative theme and goals of the TCORS

The integrative theme of the UVM TCORS is vulnerable populations, and its goals are to model the potential effects of tobacco product standards on product use in vulnerable populations, with the goal of reducing the risks of product use, dependence, and product-related adverse health outcomes. For the FDA to effectively execute its tobacco regulatory responsibilities, it must have sound scientific evidence on how product standards impact tobacco use in populations with high rates of tobacco dependence. This project will provide the FDA with evidence on the effects of a reduced-nicotine standard for cigarettes, alone and combined with another FDA-regulated product (e-cigs), on measures of cigarette use, demand, dependence, tobacco toxicant exposure and psychiatric symptoms in this vulnerable population.

2.3. Relevance to the scientific domains and priorities of the FDA CTP

The proposed research is highly relevant to the CTP's scientific domains of Addiction and Behavior because it will address whether reducing the nicotine content of cigarettes reduces cigarette use, dependence, and product appeal, and whether these effects are enhanced by the availability of appealing alternative sources of non-combusted nicotine. It will address the **Health Effects** domain by assessing the effects of these conditions on tobacco toxicant exposure and a respiratory biomarker.

2.4. How study outcomes will improve scientific knowledge related to the manufacture, distribution and marketing of tobacco products

Study outcomes will directly inform scientific knowledge concerning the manufacture of tobacco products by demonstrating whether a reduction in the maximum nicotine content of cigarettes sold in the US to ≤ 0.4 mg nicotine/g tobacco would reduce smoking in this vulnerable population. The outcomes will also indicate whether continuing to allow the sale of e-cigs in characterizing flavors improves the efficacy of a reduced-nicotine standard for cigarettes on smoking reduction in this population.

3. RATIONALE

3.1. Effects of Nicotine Reduction in Smokers with AD

Controlled trials of research cigarettes differing in nicotine content in general population samples have demonstrated that those who are switched to VLNCCs reduce their daily cigarette use, nicotine exposure, cigarette dependence, and tobacco toxicant exposure, with few adverse consequences.[5-8] Given the low smoking cessation rates in smokers with ADs, it is important to consider whether these smokers experience benefits or negative consequences of nicotine reduction. Prior to the work accomplished by this TCORS during Phase 1 funding, few studies had examined effects of VLNCCs in smokers with ADs. Several laboratory studies in smokers with elevated depression or anxiety symptoms found that both VLNCCs and normal-nicotine cigarettes reduce craving and withdrawal and do not exacerbate depression or anxiety (reviewed by Gaalema et al.[38]). We recently conducted a secondary analysis of a large randomized clinical trial that examined the effects of cigarettes varying in nicotine content over a 6-week period in non-treatment-seeking smokers, in which we examined whether those with higher vs. lower depressive symptoms at baseline differed with regard to their responses to cigarettes varying in nicotine content.[39] We found that after 6 weeks of use, those who had used VLNCCs had lower smoking rates, nicotine dependence and craving than those

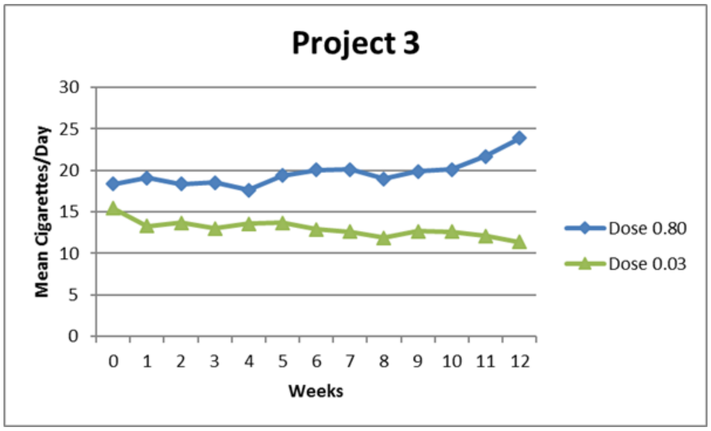

who had used NNCCs, regardless of baseline depressive symptom score. Furthermore, those with elevated depressive symptoms at baseline who had been assigned to VLNCCs had lower depressive symptoms at Week 6 than those who had used NNC cigarettes. Importantly, biochemically-confirmed VLNCC compliance also did not differ by group. These findings provide support for the idea that a reduced-nicotine standard for cigarettes may reduce smoking, without worsening depressive symptoms, in smokers with elevated depressive symptoms. However, that study examined VLNCC effects in a non-clinical sample with elevated depressive symptoms, not those with diagnosed depression or anxiety disorders.

To our knowledge, we are the first to report the effects of reduced-nicotine cigarettes on measures of cigarette reinforcement in smokers with ADs. In Study 1, we examined the acute subjective and behavioral effects of cigarettes varying in nicotine content (15.8, 5.2, 2.4 and 0.4 mg nicotine/g tobacco) in smokers with Ads.[9,10] In concurrent choice testing with the cigarettes available at an equal response effort, participants chose the higher over the lower nicotine content cigarettes across each of the six dose pairs consistent with reduced nicotine content cigarettes having lower addiction potential (below, left). The only difference between populations was at the 0.4 versus 2.4 mg/g dose pair, where smokers with affective disorders chose the higher dose more often ($p < .001$), while disadvantaged women ($p = .06$) and those with opioid dependence ($p = .91$) did not exhibit a significant preference between those two doses (below, right). All doses decreased craving and withdrawal symptoms with no evidence of compensatory smoking. While the higher nicotine cigarettes were rated as more satisfying, increasing the response requirement to obtain these cigarettes reversed that preference. This observation has considerable tobacco regulatory implications. For example, allowing VLNCCs to be sold in common retail outlets while restricting sale of higher content cigarettes to more regulated stores would be predicted to shift preference towards the former. This same concept may also extend to regulatory efforts to shift preference from harmful combusted to less harmful non-combusted tobacco products. Overall, our results indicate that reducing the nicotine content of cigarettes reduces the relative reinforcing effects of smoking, and hence addiction potential, in smokers with ADs and other vulnerabilities to tobacco addiction. The 0.4 mg/g most robustly differed from the 15.8 mg/g dose in this population, supporting prior hypotheses about reducing nicotine content below 0.7 mg/g[3].

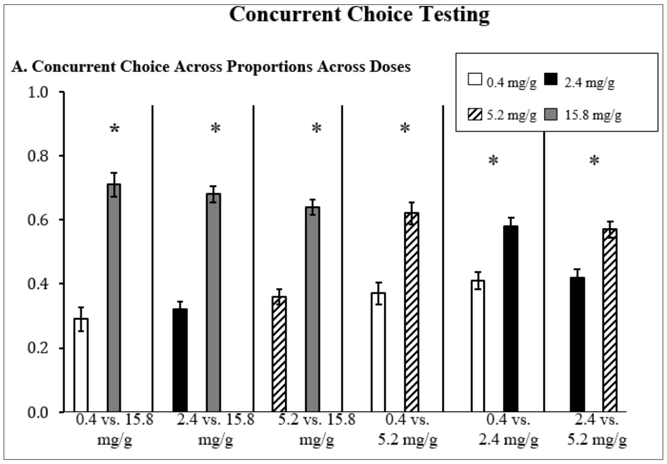

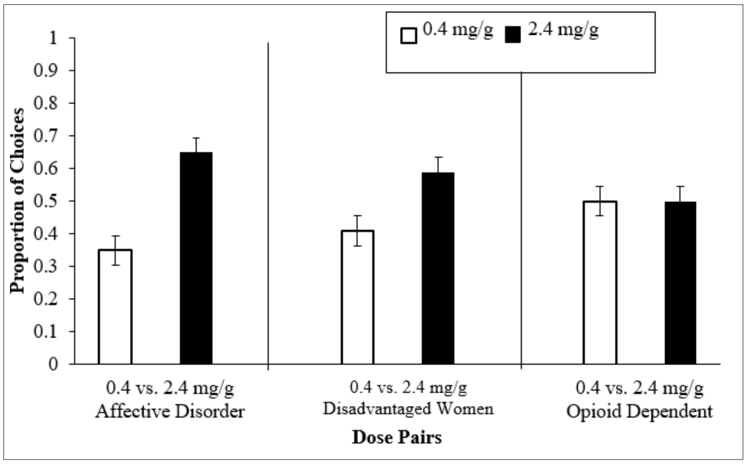

In Study 2, we are examining effects of cigarettes varying in nicotine content in smokers with ADs over a 12-week period. Preliminary results from 35 participants indicate that VLNCCs reduce smoking rates by >40% relative to NNCCs in Weeks 9-12 (see figure at right). However, while smokers with ADs reduce their smoking rates, few quit completely, similar to results seen in general population samples.[7] These findings set the stage for examining whether providing an alternative, non-combusted source of nicotine (e-cigs) enhances the efficacy of reduced-nicotine

cigarettes in this population.

3.2. E-Cig Use

E-cigs consist of a cartridge containing an e-liquid solution of nicotine, propylene glycol (PG), vegetable glycerin (VG), flavoring, and other additives, which is heated with an atomizer that vaporizes the solution. First generation e-cigs resemble cigarettes (“cigalikes”) and are disposable or rechargeable, second generation products (such as the product that will be used in this study) often resemble pens and have refillable e-liquid reservoirs, and third generation devices have larger batteries, adjustable power delivery and replacement heating coils and wicks.[40] Nicotine levels from e-cigs can be comparable to those from cigarettes, depending on e-cig characteristics, e-liquid nicotine content and user topography.[41-44] Potential risks of e-cig use include exposure to low levels of carcinogens, toxicants, metals in the vapor, and cytotoxic effects of flavors.[45,46] However, toxin and carcinogen levels from e-cigs appear to be far lower than those from cigarettes. Hecht et al.[47] reported that former smokers who had switched to e-cigs had 59-99% lower levels of 6 tobacco toxicant and carcinogen metabolites than ongoing smokers, comparable to reductions seen in smokers who had switched to nicotine lozenges.[8] Another study found that NNAL, a metabolite of the tobacco carcinogen NNK, was reduced by 64% in smokers who had switched to e-cigs for two weeks, as was chest tightness.[48]

E-cig use has increased sharply in the US, particularly among people with MHCs. A 2012 national probability survey in >10,000 US adults found that people with MHCs (including ADs) were twice as likely to have ever used or to currently use e-cigs than those without MHCs.[49] Survey data from 2015 indicate a doubling of use since the Cummins et al. survey, and people with MHCs were still twice as likely to use e-cigs as those without MHCs (24.4% ever use, 11.4% current use).[50] Of those with depression and anxiety disorders, 24-30% reported ever use and 11-13% reported current use.[50] Two surveys also reported that likelihood of e-cig use was associated with psychiatric symptom severity or number of MHCs endorsed, suggesting a systematic relationship.[50,51] Smokers in general report that their reasons for e-cig use are to quit or reduce smoking, to reduce cigarette craving and nicotine withdrawal, because e-cigs bother other people less than cigarettes, and because they can use e-cigs in places where smoking is forbidden.[51-55] Smokers with MHCs report similar reasons for use.[56-59]

3.3. E-cig effects on smoking

To date, clinical trials indicate effects of e-cigs on smoking reduction, but little effect on quitting.[60-62] Modest effects on cigarette abstinence in these trials may have been due to use of first generation e-cigs that provided variable nicotine delivery and were subject to battery failure.[63] A trial using second generation e-cigs found cigarette abstinence rates of 34% after 8 weeks and 21% 6 months later, with an overall 60% reduction in CPD.[64] Few studies have examined the effects of e-cigs in smokers with ADs. Among hospitalized smokers with serious mental illness enrolled in a cessation trial, e-cig use increased during the trial but was not associated with smoking abstinence.[65] Likewise, a survey in veterans found a strong association between MHCs and e-cig use, but no association with quitting.[56] A secondary analysis of the Bullen et al. ASCEND trial[60] found that mental health status did not moderate the effect of e-cigs on quitting.[66] A pilot study in 21 smokers with psychotic disorders found that e-cig use decreased CPD.[59] Both of the latter studies reported high rates of e-cig acceptability among smokers with MHCs.[59,66]

We recently completed a pilot study in 18 smokers who were asked to switch to a second-generation e-cig with 18 mg/ml nicotine e-liquid for 6 weeks.[67] All of those enrolled completed the study. Participants significantly reduced their CPD, breath CO levels, Fagerström Test for Cigarette Dependence scores, and increased their readiness to quit, all with large effect sizes (Cohen’s *d*’s ranging from 0.88 to 1.3). At a follow-up visit 4 weeks later, changes from baseline had been maintained with large effects sizes on all measures (Cohen’s *d*’s ranging from 0.8 – 1.1). This work demonstrates our experience and success with testing an e-cig similar to that which will be used in this study over a multi-week period.

3.4. Mechanisms of e-cig effects

E-cigs reduce cigarette craving and nicotine withdrawal symptoms.[68] Although early studies

found that e-cigs were less effective than cigarettes at reducing cigarette craving and withdrawal,[60,69,70] second-generation e-cigs more effectively reduce craving and withdrawal under natural *ad lib* use conditions.[41,64,71] E-cig effects on craving and withdrawal symptoms are determined by the extent to which e-cig are used by smokers; in turn, determinants of e-cig use include product appeal and reinforcing effects.[72,73]

3.5. Importance of e-cig flavors

More than two-thirds of adult e-cig users in 2013-2014 used a flavored e-cig.[74,75] Flavors have been shown to substantially enhance the appeal and relative reinforcing effects of e-cigs[76,77] and have been cited as a key feature of e-cigs affecting use among adults.[75,78,79] Experimental studies show that flavors increase demand for e-cigs among cigarette smokers,[80,81] particularly smokers who are not current e-cig users.[81] Studies of e-cig users also highlight that flavors play an important role in their experience of the product[82-84] and in reducing cigarette consumption and craving.[83,85] Although a recent review on e-cigs and mental illness reported one study of flavors, which found no differential appeal in young adult smokers with mental illness in the VA,[57] flavored (menthol) cigarette use is preferentially used among individuals with severe psychological distress[86] and is associated with greater prevalence of both depression and anxiety in US young adults.[87] Therefore, it warrants investigation whether providing e-cigs in preferred flavors increases e-cig use and the ability of e-cigs to substitute for cigarettes in smokers with ADs.

3.6 Products to be tested

<u>Cigarettes to be assessed</u>

The cigarettes to be used in this study were made under an NIH contract with production being overseen by the Research Triangle Institute (referred to as “Spectrum cigarettes”). NIH currently has approximately 10 million of these cigarettes (of varying types) for research purposes. The cigarettes selected for the study span the range of yields likely to produce the hypothesized effects, as described above. Spectrum cigarettes are not currently commercially available, although they are similar in many ways to marketed cigarettes (e.g., similar manufacturing, filter, paper, etc.).

<u>E-cigarettes to be assessed</u>

Both the JUUL and the Vuse Solo will be used and assessed in this study. While JUUL will be offered to all participants, participants that are unwilling to use JUUL will be offered the Vuse Solo.

JUUL is a commercially available closed system containing two components. One component contains a lithium-ion battery (200 mAh), nichrome coil heater, silica wick, and stainless steel vapor path. The other component is the prefilled e-liquid container that also serves as the mouthpiece. Each commercially available cartridge holds approximately 0.7 mL of e-liquid containing approximately 40 mg of nicotine or 5% nicotine by weight (NBW). A lower dose containing approximately 23 mg of nicotine per cartridge or 3% NBW is also marketed but will not be used in this study. All containers contain glycerol, propylene glycol, natural oils, extracts and flavors, nicotine, benzoic acid. We will not alter the e-liquid in any way. The research staff will distribute the e-liquid containers as purchased from the manufacturer. The JUUL apparatus and 5% NBW e-liquids that will be used are legally purchasable and have been as of August 8, 2016. We will not alter them in any way.

Vuse Solo is a commercially available closed system containing two components. The power/heating device includes a 270 mAh battery, silica wick, microchips, and sensor. The other component is the prefilled e-liquid container. Each commercially available cartridge holds approximately 1 mL of liquid containing 48 mg of nicotine or 4.8% NBW. All containers contain vegetable glycerin, propylene glycol, reverse-osmosis water, glycerin, flavorings, and nicotine. The research staff will distribute the e-liquid containers as purchased from the manufacturer. The Vuse apparatus and e-liquid cartridges that will be used are legally purchasable and have been as of August 8, 2016. We will not alter them in any way.

3.7. Summary

Although smoking rates have declined in the overall US population, there has been little to no decline among people with ADs. A nicotine reduction strategy for combustible tobacco combined with e-cigarette availability may have complementary effects on smoking reductions and consequent tobacco-related health effects in this vulnerable population. This research is highly significant because it will model how the availability of e-cigs, a non-combusted nicotine product that is rapidly increasing in use among US smokers, impacts the effectiveness of a reduced-nicotine policy for cigarettes in this vulnerable population. It is responsive to the goals of the FDA in that the study conditions are designed to model real-world scenarios of possible harm reduction policies in a population that is vulnerable to smoking persistence.

4. Project Study Methods

This study will use a four-condition, parallel-groups research design. After a baseline period in which daily smoking rate and other baseline assessments are completed, participants will be randomly assigned to one of the following four conditions for a 16-week experimental period: (1) normal nicotine content cigarettes (NNCCs, 15.8 mg/g) alone, which serves as the control condition; (2) very low nicotine content cigarettes (VLNCCs, 0.4 mg/g) alone; (3) VLNCCs + tobacco-flavored nicotinized e-cigs (TF e-cig, 4.8 - 5.0% nicotine by weight, NBW, if they choose to use the Vuse or JULL devise, respectively); or (4) VLNCCs + preferred-flavor nicotinized e-cigarette (PF e-cig).

5. Study Screening Procedures

5.1. Participants

Participants will be men and women, ages 21 – 70 years, with a current diagnosis of an affective disorder, defined as a major depressive disorder, dysthymic disorder, generalized anxiety disorder, post-traumatic stress disorder, obsessive-compulsive disorder, or panic disorder with or without agoraphobia based on the Mini Neuropsychiatric Interview (MINI) which uses DSM-5 criteria, OR Lifetime diagnosis of one of the above based on MINI with a self-report of currently receiving treatment for one of these disorders prescribed psychoactive medication, behavioral therapy, etc.). Additionally, they must be sufficiently literate to complete the research tasks, be in good physical health without serious illness or change in health in past three months, and have the technological capabilities to complete weekly face-to-face video assessments and the compatibility to use ico Smartphone Smokerlyzers for assessing breath carbon monoxide (CO) levels. Study inclusion and exclusion criteria are detailed below.

5.2. Recruitment

Using an intent-to-treat analysis approach, we require 53 randomized per condition (212 total) to test our primary aims. We estimate 10% attrition between enrollment and randomization based on UVM TCORS Phase 1 research, and will therefore enroll up to 59 per condition (236 total). In addition, we will pilot test with up to 20 participants (10/site) for a total of 256 participants (90 at Brown, 166 at UVM). Potential participants will respond to community advertisements (local newspapers, community bulletin boards, lab Facebook page, Facebook ads, lab website, center website, behavioral health centers, Craigslist, city buses, etc.) that contain a study description, link to an online survey and the name and phone number of the Research Assistant. Participants can choose to complete the pre-screening questionnaire online or by phone. At UVM, individuals recruited from online sources will be directed to a UVM-hosted recruitment website where they will have the opportunity to select which research studies interest them. They will then be redirected to a brief online screener to assess eligibility. The Patient Health Questionnaire-4[88] will be used to screen for probable mood or anxiety disorder. This 4-item instrument, which comprises the first 2 items of the Patient Health Questionnaire 9-item(PHQ-9) scale[89] and the first 2 items of the Generalized Anxiety Disorder 7-item (GAD-7) scale[90] measures the two core DSM-IV criteria for major depressive disorder and generalized anxiety disorder, respectively. The PHQ–4 begins with the stem question: “Over the last 2 weeks, how often have you been bothered

by the following problems?" and each item is scored from 0 ("not at all"), 1 ("several days"), 2 ("more than half the days"), or 3 ("nearly every day"). Therefore, the total score on this composite measure ranges from 0 to 12. A total score of 3 for the 2 anxiety items or the 2 depression items, plus questions querying past treatment for depression or anxiety, will be used to identify probable cases. If deemed eligible, those who complete the online questionnaire will be called by the Research Assistant to further discuss the study. The RA will read a script briefly explaining the study. Participants will be informed that this is not a smoking cessation program, and that smoking cessation services are available in the community independent of their decision to participate in this study. If interested, they will be invited to participate in the first portion of the screening interview. Research assistants will inform eligible participants that the screening will occur over video chat, and will assist the participant with setting up an appropriately secure video platform.Those who call into the laboratory will be read a script briefly explaining the study. After verbal informed consent is received, the participants will be asked questions over the phone to determine initial eligibility.

During this first portion of the screening, the participant will complete questionnaires through REDCap online while the research assistant is present over video chat or phone to deliver instructions and to answer any questions. The participant will then answer interviewer-administered questionnaires over video chat. Participants who did not yet set up their video platforms will do so with the research assistant before beginning any questionnaires. Participants will be instructed to have picture identification (e.g. driver's license) available to show the staff. If participants anticipate not having acceptable ID, staff should consult with the project coordinator or study PI. Initial study eligibility will be determined after data are collected from this visit. Participants who meet initial study eligibility will be scheduled for the second portion of the screening.

Before the second portion of the screening occurs, eligible participants will receive the equipment necessary to use for collecting physiological measurements. Participants will be asked to pick up this equipment via curbside pickup at our clinic (UVM University Health Center, UHC), which will consist of participants calling staff once they arrive at UHC and staff coming out to give participants a bag/box containing the following equipment: a Smokerlyzer; an audio jack adapter for the Smokerlyzer if necessary; a blood pressure cuff; an oximeter; a thermometer; a urinary cotinine dipstick; urine cups with attached temperature test strips; a pregnancy test strip (if applicable) and urine toxicology test strips or a saliva toxicology test. Participants (and staff) will be asked to use cloth face coverings when exchanging product. If participants arrive via car, staff will drop this bag on the hood of the participant's car while the participant remains in the car Participants may be invited to come inside to pick up this equipment if the participant is asked to wait for this exchange. All participants must pass a COVID19 screening before entering the building. If there is any waiting that needs to occur inside the building, the participant will wait inside one of our five highly ventilated smoking chambers. If there happens to be no space in the smoking chambers, the participant will be told that they cannot come up to the clinic until space is available. After each use, the all of the surfaces in the smoking chambers will be cleaned with 70% or greater of alcohol solution by staff wearing a mask and gloves, as well as all of the door handles. If the participant uses the bathroom while they are in the clinic, the bathroom surfaces and handles will be wiped down after use by staff while masks and gloves are worn. Participants who are using the smoking chambers at any point in the study to wait for product or equipment exchange will remain in the chambers until a staff member comes to knock on the door to let them out. In this way, we can avoid people coming into close contact with each other in the larger room that contains the smoking chambers. A minimum of 6 feet of distance will be maintained for all staff and participants at all times. For participants who come to the clinic, a commercial courier will deliver this equipment to them before the second portion of the screening.

If at any point the Smokerlyzers are not available for distribution, we will conduct the CO test curbside before or after participants are invited inside and the courier service will not be available. Research Assistants will bring down the CO monitor when they bring the rest of the equipment to the participant. While maintaining 10 feet of distance and wearing gloves, staff will explain how the CO monitor works. Once the participant is ready, staff will press the button to

obtain the measurement and will set the CO monitor down and will back away 10 feet. The participant will then come to pick up the device and will blow into the monitor. After the participant completes the test, they will set down the monitor and back up 10 feet and staff will retrieve the monitor. After every use, staff will wipe down the CO monitor with disinfectant wipes and hydrogen peroxide wipes. When using the monitor, a D-piece (a portable valve filter) must be placed into the monitor and then the single use plastic mouthpiece is placed into the D-piece. The monitor has built in SteriTouch technology to ensure optimum infection control, and the D-pieces filter out 99.9% of airborne bacteria and greater than 97% of viruses for excellent infection control. Each participant will be assigned their own D-piece to use throughout the study, and no D-piece will ever be shared among participants. Participants will gently exhale into the D-piece for the breath carbon monoxide reading. Participants will be instructed only to exhale through the device, not to inhale..D-piece technology also includes a one-way valve that prevents air from being drawn back from the monitor. D-pieces will also be wiped down after each use with disinfectant wipes and hydrogen peroxide wipes and stored in a container at the lab.

Once participants have received the necessary equipment to complete the physiological portion of the screening, the research assistant will initiate a video call with the participant. During this call, the participant will be instructed on how to use the equipment and then will be asked to use the equipment to obtain the following physiological readings: blood pressure, heart rate, oxygen saturation, temperature, and breath CO levels. Participants will also be asked to collect a urine or saliva sample during the visit. If a saliva sample is collected, the participant you will provide the saliva sample over video chat while the staff observes. If a urine sample is collected, staff will ask participant to bring this urine sample to the video screen after collection to perform a urine toxicology test and a pregnancy test (if applicable). These urine cups will have temperature strips affixed to ensure that the sample is valid. Participants who have a carbon monoxide level of less than or equal to 8 will also be asked to use the urinary cotinine dipstick to determine whether they are positive for cotinine. The participant will obtain the physiological readings and perform the tests and then will hold the results of the test up to the camera so that the research assistant can interpret and record the readings on REDCap, Potential participants will also be instructed to have handy a pack of their usual brand cigarettes, all prescription medications they are currently taking and identification (example, driver’s license) during this second portion of screening visit. If participants anticipate not having acceptable ID site staff should consult with the project coordinator or study PI.

A participant must complete his/her two-part screening session within 30 days of completing the pre-screening questionnaire. If the participant is not able to complete the two-part screening visit in that timeframe, he/she will need to complete the pre-screening questionnaire again.

5.3. Informed Consent Process:

Before beginning the informed consent process, potential participants will need to produce identification as described above. The interviewer will confirm the age and identity of the participant. If the participant is not between the ages of 21 and 70, he/she will be dismissed without payment. During the first portion of the screening session, study information will be presented and documentation of the participant’s informed consent via electronic signature on REDCap will be required prior to participating in the screening session. In order to ensure adequate informed consent, participants will be asked to read the first several lines aloud (to determine literacy) and will then be given ample time to read the consent document. If the interviewer suspects the participant is not literate, he or she will have them continue reading further to confirm. Inability to read and comprehend written study materials will result in ineligibility and the interviewer will inform the participant that they are not eligible. Only after the participant and the researcher are fully satisfied that the participant understands the purpose of the study, the confidentiality of the data, the procedures, the risks/benefits and his/her rights as a research participant will the consent form be signed and the participant undergo screening procedures.

5.4. Screening Measures

Those who consent will be screened for eligibility using the following measures:

The following physiological measures will be collected and entered directly into REDCap by the interviewer:

1) Expired breath carbon monoxide (CO) levels will be assessed using an ico Smokerlyzer Smartphone Monitor (Covita -for remote collection) or a Bedfont CO monitor (for curbside collection), a reliable and valid measure of recent smoking.
    a. Urinary cotinine test strips will be used to asses cotinine levels if a participant's carbon monoxide reading is less than or equal to 8 ppm.
2) A urine or saliva toxicological screen will be performed to assess the presence of illicit drugs including up to the following drugs: marijuana, cocaine, opiates, oxycodone, benzodiazepines, barbiturates, amphetamines, methadone, buprenorphine, methamphetamines, MDMA and PCP. Participants who fail the drug screen for drugs other than marijuana or their prescribed opioid medication may reschedule the interview but will need to be re-consented to ensure they have received adequate informed consent. They will be excluded if they are positive for drugs (other than marijuana or prescribed medications as determined by PI on a case-by-case basis) the second time.
3) Urine Pregnancy Test (HCG detection) will be performed for all participants.
4) Blood pressure and heart rate will be measured using an automated blood pressure monitor and a finger pulse oximeter to help the licensed medical professional determine final participant eligibility. Participants will be told if their blood pressure is in an abnormal range and advised to see a doctor by research staff. The research staff will also submit a medical event form for the LMP to review along with a Blood Pressure and Heart Rate Symptom Checklist form to ascertain details of the symptomatology for the LMP to review. In severe cases, the LMP may also choose to call the participant to follow-up and/or withdraw the participant from the study if necessary. All of these procedures are documented in our Blood Pressure/Heart Rate Collection: Standard Operating Procedure form which we can submit to the IRB if the Committee deems necessary.
5) Body temperature, respiratory rate and oxygen saturation will be added as physiological measures based on the CDC recommendations and those of Dr. David Kaminsky. https://www.cdc.gov/tobacco/basic_information/e-cigarettes/severe-lung-disease/healthcare-providers/index.html

The following screening assessment will be administered on paper as an interview:

1) The Mini International Neuropsychiatric Interview (MINI) 7.0[70]

The following screening assessments will be administered as an interview and then will be entered directly into REDCap by the interviewer:

1) The MINI Plus Modules
2) The MINI suicide subscale[94] to evaluate suicide risk.
3) MINI Follow-up Questionnaire (if applicable)
4) Tobacco Use History and Exposure Questionnaire, which measures variables such as smoking amount, cigarette brand, age of initiation of smoking, number of quit attempts, duration of quit attempts and duration of smoking.
5) Smoking Cessation Therapy Use Questionnaire
6) Time Since Last Cigarette Questionnaire
7) Medical History Questionnaire to assess current diagnoses, symptoms and past health problems.
    a. Medications will be recorded directly onto the Concomitant Medications form in REDCap.
8) Drug Abuse Screening Test (DAST-10), which assesses quantity and frequency of alcohol and drug use (12 month and 1 month version)

The following screening assessments will be completed by the participant directly in REDCap, except where noted otherwise:

1) Demographic History Questionnaire, which will assess age, gender, ethnicity, race, education, income, marital status, and employment history.
2) Alcohol Use Questionnaire---based on the Alcohol Use Disorders Identification Test[95] (12 month and 1 month version)
3) Drug Use Questionnaire---based on the Drug Abuse Screening Test[96] (12 month and 1 month version)
4) Fagerström Test for Nicotine Dependence (FTND)[97];
5) Wisconsin Inventory of Smoking Dependence Motives-Brief Scale (WISDM)[97] will be administered to assess nicotine dependence severity.
6) Penn State Electronic Cigarette Dependence Index[99];
7) Smoking Stages of Change Algorithm[100];
8) Identifying Information Form will include the participant's REDCap Subject Identifier, name, address (including the county of residence), email address, phone number, age, date of birth, and social security number (if applicable).
    a. This form will be entered into the 'Identifying Information Access Database'.
        - Each site will have a separate 'Identifying Information Access Database'.
        - Identifying information will not be shared with other sites. Each site is responsible for maintaining confidentiality of this information.
        - Identifying information will be kept in a locked file cabinet (source document) and in a password protected Access Database (electronic version) separate from all other study data.
9) Beck Depression Inventory (BDI-II)[91], to assess depressive symptoms.
   (This form has been updated on 1.29.20 so as to use the BDI-II. The BDI-II will be used instead of the BDI so that the data collected will be directly comparable to other projects using the BDI-II).
10) Overall Anxiety Severity and Impairment Scale[92](OASIS); to assess frequency and severity of anxiety symptoms.
11) COVID19 Symptom Questionnaire
12) Respiratory Symptom Questionnaire will be administered to assess respiratory health

In the event that the REDCap website is not functioning, the assessments will be administered aloud and participant answers will be recorded securely. The interviewer will enter the data into REDCap when it resumes functioning properly. This information should be recorded in the 'End of Visit Evaluation Form'.

5.5. Suicidality/Mental Health Monitoring

Participants who endorse suicidal intention in the past month or a suicide attempt in the past 6 months as indicated on the BDI (score > 1 on question 9) or MINI suicide subscale (endorse question 4 and/or 5 on the MINI suicide subscale or question 6 on the MINI suicide subscale with suicide attempt in the past 6 months) or answer "yes" to question A3g on the MINI Neuropsychiatric interview and symptoms have occurred in the past two weeks will be assessed by a clinician for eligibility and possible intervention. The research staff member will contact a licensed clinician for evaluation. In the event that no clinician is available, staff will put the participant in contact with the National Suicide Prevention Lifeline at 1-800-273- 8255. They will also contact the Study Coordinator and Site PI to inform them of the situation as soon as possible. Additionally, they will contact the Project Coordinator to inform her of the situation. The participant will be paid $25 (+$25 bonus if applicable) and provided with local mental health resources. Post enrollment, any report of suicidal ideation or attempt by a participant will be grounds for immediate withdrawal from the study.

5.6 Inclusion/Exclusion Criteria

Inclusion Criteria:

1) Men and women ages 21-70,
2) Past-year: MDD, dysthymic disorder, generalized anxiety disorder, post-traumatic stress disorder, obsessive-compulsive disorder, or panic disorder with or without agoraphobia,

based on MINI structured interview, OR Lifetime diagnosis of one of the above based on MINI with a self-report of currently receiving treatment (prescribed psychoactive medication, behavioral therapy, etc.),

3) Report smoking ≥ 5 cigarettes per day for the past year,
4) Provide an intake breath CO sample >8 ppm, (if ≤ 8 ppm, then urinary-cotinine strip must be positive)
5) Be without current substance abuse/dependence other than nicotine,
6) Be sufficiently literate to complete the research-related tasks,
7) Be in good physical health without serious illness or change in health in the past three months as determined by the licensed medical professional at each site,
8) Have appropriate equipment to complete face-to-face video assessments and use ico Smartphone Smokerlyzer Monitors. For those who do not have a Smartphone, staff will explore potential alternate plans (e.g., project-provided inexpensive Android phone)

Exclusion Criteria:

1) Exclusive use of roll-your-own cigarettes,
2) Planning to quit smoking in the next 30 days,
3) A quit attempt in the past 30 days resulting in greater than 3 days of abstinence,
4) Significant use of other tobacco or nicotine products within the past month (more than 9 days in the past 30).
5) Positive toxicology screen for any of the following drugs: cocaine, opiates, methadone, oxycodone, buprenorphine, benzodiazepines, barbiturates, amphetamines, methamphetamines, MDMA and PCP
    a. Marijuana will be tested for but will not be an exclusionary criterion. Participants will be discouraged from smoking marijuana during the study.
    b. Participants with valid prescriptions for opiates, benzodiazepines, barbiturates, or amphetamines will not necessarily be excluded.
    c. Participants failing the toxicology screen will be allowed to re-screen once. These participants will need to be re-consented before being rescreened to ensure they have received adequate informed consent.
6) Self-report of binge drinking alcohol (more than 9 days in the past 30 days, 4/5 drinks in a 2 hour period in females/males),
7) Systolic blood pressure < 90 or ≥ 160 mmHg
    a. Participants failing for blood pressure will be allowed to re-screen once.
8) Diastolic blood pressure < 50 or ≥ 100 mmHg
    a. Participants failing for blood pressure will be allowed to re-screen once.
9) Breath CO > 80 ppm,
10) Heart rate is greater than or equal to 115 bpm or less than 45 bpm
    a. Participants failing for heart rate will be allowed to re-screen once.
11) Currently seeking treatment for smoking cessation,
12) Being pregnant, trying to become pregnant, or nursing, or not report using a form of approved birth control if applicable determined by the Project Medical Director
13) Have used nicotine replacement, bupropion or other pharmacotherapies as cessation aids in the past month (bupropion will be allowed for treatment of depression),
14) Unstable psychiatric conditions (psychiatric medication changes in the past 4 weeks),
15) Symptoms of psychosis, dementia or mania,
16) Suicidal ideation in the past month (score > 1 on the BDI question 9 or endorse question 4 and/or 5 on the MINI suicide subscale),
17) Reporting a plan or attempt to commit suicide, which is assessed on question A3g of the MINI Neuropsychiatric Interview Major Depressive Episode Module. Thoughts of suicide without an intent or plan is not an exclusion criteria,
18) Suicide attempt in the past 6 months (endorse question 6 on the MINI suicide subscale with suicide attempt in the past 6 months),

19) Participation in another research study in the past 30 days,
20) Co- habitation with any research participant who has or is participating in the current study,
21) Daily use of e-cigarettes in the past month (defined as 6 – 7 days per week)._
22) Oxygen saturation of < 90%
23) Reporting positive symptoms for COVID19

Individuals under age 21 are excluded because they cannot legally buy cigarettes. Those with unstable medical, psychiatric, or medication conditions (as determined by the licensed medical professional) are excluded as these symptoms could affect a participant's ability to complete the study. Examples include but are not limited to the following: angina, stroke, heart attack which occurred since phone screening, blood clots in the arms or legs for which the individual is undergoing active medical treatment, cancer requiring active chemotherapy or radiation therapy, severe shortness of breath caused by conditions such as uncontrolled asthma, COPD, or arrhythmia, active untreated infection such as pneumonia, active untreated endocrine disorder such as hyperthyroidism. We will exclude those currently seeking smoking treatment and those who plan to quit in the next 30 days, as participation in this study may not lead to reductions in smoking. We will exclude pregnant or nursing women and women of reproductive potential who are unwilling to use acceptable forms of birth control throughout the study if applicable determined by the Project Medical Director. We will also exclude anyone with current or recent alcohol or drug abuse problems as these factors could independently affect smoking behavior during the study. Individuals with baseline CO readings greater than 80 ppm, those with heart rate or blood pressure readings that are out of range (systolic: 90-159 mmHg; diastolic: 50-99 mmHg; HR: 45-114 bpm) and anyone who has attempted suicide in the past six months will be excluded from the study for safety concerns. Individuals who smoke 'roll your own' cigarettes exclusively will be excluded from the study because we will be unable to standardize their baseline smoking behavior. Individuals who have reported daily use of e-cigarettes in the past 30 days will be excluded as they may not be compliant with experimenter-provided e-cigarettes. Individuals who have recently participated in a research study will be excluded as participation may have changed their smoking patterns, which may preclude a stable smoking baseline. Because participants are required to complete portions of the protocol independently, they will need to be able to independently read and comprehend the study materials.

5.7. Eligibility Determination:

The research assistant will review the entire screening assessment battery for initial eligibility determination, confirming the participant meets the above described inclusion/exclusion criteria. All eligibility criteria that are not related to physiological measurements will be assessed during the first portion of the screening visit, and all criteria related to physiological measurements will be determined during the second portion of the screening. The final eligibility of the participant will be determined by a licensed medical professional (MD,DO, NP, PA, Master's prepared RN or CRN) at each site after reviewing the Medical History Questionnaire, BDI, Mini Neuropsychiatric Interview, and the MINI suicide subscale. The licensed medical professional may meet with a participant if available and think it necessary for eligibility determination. He/she will sign off on eligibility prior to the first baseline visit. If the licensed medical professional determines the participant is not medically eligible to participate in the study, has current symptomatology that would interfere with interpretation of the data, or is unlikely to complete the study he/she will inform the research assistants who will contact the participant prior to the first baseline visit. The licensed medical professional will not need to review the medical history forms of participants who are ineligible for other, non-medical reasons.

If a participant fails the urine or saliva toxicology screen due to a prescription medication he/she is taking, then he/she will not be automatically excluded. The interviewer will make note of this when he/she submits the forms to the licensed medical professional for final eligibility determination.

Once all the screening procedures have been completed, researchers will pay participants $25 (+$25 bonus if applicable) for their time as long as they pass the drug tests and meet the

minimum requirements for carbon monoxide or urinary cotinine levels. Participants will be paid after the completion of the study visit. If participants are deemed ineligible at any point in the screening, the participant will be paid after determined ineligible. Marijuana will be tested for but will not be an exclusionary criterion. If a participant does not pass the drug test but has a current, valid prescription that would explain the failed test he/she will not be automatically excluded and will still receive the visit payment. Participants who meet all other eligibility criteria, sans the medical criteria, will be scheduled for the first baseline visit.

At the end of the screening session, the researcher will complete the End of Visit Evaluation Form. This will allow the researcher to make note of any problems encountered during the visit and to assess the truthfulness of the participant in regards to self-report of tobacco use.

6. Study Baseline Procedures

This study will use a one-week, two-session baseline period to collect baseline individual difference measures and monitor daily usual-brand smoking behavior. At Baseline 1 or within 1 business day of the Baseline 1 visit, participants will be provided their usual brand cigarettes to smoke, equivalent to 150% of their daily smoking rate. Participants will be encouraged to come to the lab to pick up their usual brand cigarettes in a curbside exchange after they complete the questionnaires and physio for the BL1 visit. Those who cannot come to the lab will receive product via a commercial courier. A time line follow back (TLFB) will be used during the period between Baseline 1 and Baseline 2 to assess the daily cigarette use for the first 7 days the participant has product. The participant must have received their UB cigarettes from the lab before the 7-day assessment period starts, and Baseline 2 must occur at least 7 days after the participant receives their usual brand cigarettes from the lab. If the baseline period extends past seven days and if the participant has run out of product, participants will need to purchase their own usual brand cigarettes. Use of a two session baseline period will ensure stability of daily smoking reports, reduce reactivity to the daily cigarette monitoring, and reduce participant burden. During the two baseline sessions, participants will complete subjective questionnaires. Each visit will last approximately two to four hours. At the end of each baseline session, the researcher will complete the End of Visit Evaluation Form,. This will allow the researcher to make note of any problems encountered during the visit and to assess the truthfulness of the participant in regards to self-report of tobacco use. Participants will also be supplied with saliva test equipment and urine collection equipment during the Baseline 1 product exchange so that they can collect saliva and first void urine samples during the Baseline 2 visit.

For the Baseline 1 visit and all subsequent visits, the participant will be sent a REDCap link within 15 minutes of the start of the scheduled visit to complete all of the non-interviewer administered questionnaires. The participant will complete these questionnaires on their own but can have the research assistant present on a video call if they desire. Before beginning the physiological assessment portion of the visit over video call, the research assistant must review the participant's questionnaire responses for that visit. Participants will be compensated after the completion of the study visit and when the participant has received their new product.

At Baseline 2 and all subsequent visits, after the participant has answered the questionnaires and has completed the physiological portion of the visit over video call, participants will be asked to come to the lab for exchange of product and biological samples. Participants will bring in their used and unused product from the previous visit as well as a first-void urine sample for assessing tobacco- related toxin exposure and a salivary sample for assessing nicotine metabolism rate on applicable visits (BL2, Week 8 and Week 16) using equipment that was provided at the previous visit. Participants will be instructed to call the RA at the office when they get to the clinic to ensure that there is enough space in the smoking chambers to house all participants while abiding by safety guidelines as detailed on page 14 of the protocol. All participants must pass a COVID19 screening before entering the building. When invited into the lab, the participant will be shown to a smoking chamber and will instructed to place their bag of product outside of the chamber. The participant will wait here while the RA processes and returns product through the randomization database. Then the RA will dispense new product and bring the bag back to the participant. When

the RA deems it safe for the participant to exit the chamber, the RA will instruct the participant that they can leave. The RA will instruct the participant to observe social distancing measures during this exchange, providing clarification if necessary. If a participant forgets their first-void urine sample at the Baseline 2 visit, staff will ask participant to come back to the clinic with their first void urine sample before exchange of product occurs. If participant is unable to return to the clinic with their first void sample, staff can arrange to meet the participant off campus to pick up their urine sample and to give participant their study product. Distancing and safety measures as described above must be observed. For participants who cannot make it to UHC, special arrangements will be made to enable use of the randomization database and product return/distribution procedures to the extent possible. Each week, during - or scheduled as nearly as possible to – a virtual visit, a complete accounting of the participant's product inventory will be taken and processed remotely through the randomization database. The participant will separate product based on its type (e-cigarette or combustible inventory) and status (used/unused), and the RA will process return characteristics through the database accordingly. The RA will clarify barcode characteristics with the participant when legibility is compromised. The participant will be instructed to keep unused product in their possession, but to exchange any used product with the courier who will deliver newly dispensed replacement products within 48 hours.

Product that will be given to participants for the Baseline 2 visit cannot be given/sent to participants until 7 days have passed following completion of the Baseline 1 visit. We will need to calculate baseline smoking rate during this 7-day period and so participants cannot have access to any blinded study product or e-cigarettes before this 7-day period has ended.

6.1. Visit scheduling requirements for baseline period:

Participants will be required to schedule the Baseline 1 visit within 30 days of the completion of their screening visit. If a participant still wants to be in the study after 30 days, he/she will need to be re-screened. The participant will need to be re-consented but will maintain the original REDCap Subject Identifier. The ideal target window separating Baseline 1 and Baseline 2 is between 7 and 12 days. The minimum is 7 days and the maximum is 21 days. If the participant does not complete the visit within 21 days, then he/she will not be rescheduled and will be discontinued from the study.

6.2. Measures/Assessments

The following physiological measures will be collected and recorded directly into REDCap by the interviewer:

1) CO
2) Blood Pressure
3) Heart Rate
4) Body temperature
5) Oxygen saturation
6) Respiratory rate
7) Urine or Saliva Toxicology
8) Urine Pregnancy test (if applicable, to be performed every 2 weeks)

The following assessments will be administered as an interview at Baseline 1 and entered directly into REDCap by the interviewer at the end of the visit:

1) Concomitant Medications Form
2) Health Changes Questionnaire, which will assess any weekly health changes,
3) Time Since Last Cigarette Questionnaire

The following assessments will be administered at Baseline 1 and completed by the participant directly in REDCap:

1) BDI

2) OASIS
3) Respiratory Symptom Questionnaire
4) COVID19 Symptom Questionnaire
5) Wisconsin Inventory of Smoking Dependence Motives-Brief Scale (WISDM) will be administered to assess nicotine dependence severity.
6) Perceived Health Risks Rating[101], a measure of the perceived addictive potential and other health risks associated with cigarettes;
7) Perceived Stress Scale (PSS)[101], assessing the degree to which life situations are perceived as stressful;
8) Positive and Negative Affect Scales (PANAS)[102], a measure of changes in positive and negative mood;
9) Respiratory Health Questionnaire, a UVM measure of cough, shortness of breath and other respiratory symptoms;
10) Minnesota Nicotine Withdrawal Scale (MNWS)[103], a measure of nicotine withdrawal;
11) Questionnaire of Smoking Urges-brief scale - Usual Cigarette (QSU)[104], which measures the urge to smoke;
12) Vaping Craving Questionnaire[105], which measures the urge to vape;
13) Cigarette Evaluation Scale – Usual Cigarette (CES)[106], which measures responses to cigarettes (e.g., reward, satisfaction);
14) Vaping Evaluation Scale (VES), which measures responses to taping (e.g. reward, satisfaction)
15) Cigarette Purchase Task – Usual Brand Version (CPT)[107], a self-report analogue of a progressive-ratio schedule that measures the relative reinforcing efficacy of cigarettes by querying how many of that day's cigarette they would consume in a day at varying prices. This task will indicate whether prolonged VLNC cigarette use reduces cigarette demand and increases sensitivity to increases in cigarette costs;
16) Snaith-Hamilton Pleasure Scale (SHAPS)[110].

All participants will also be asked to select their top three flavors of e-cigarette liquid from a list read to them by the RA. This question will be asked in preparation for giving flavored pods to participants who are randomized into the flavored e-cigarette condition. This information will be recorded directly into REDCap. Participants will be asked to rate these top three flavors based on either previous experience with these flavors or to indicate how much they believe that they will like or dislike the flavors.

Physiological measures collected at Baseline 2 will be entered directly into REDCap by the interviewer:

1) CO
2) Blood Pressure
3) Heart Rate
4) Body temperature
5) Oxygen saturation
6) Respiratory rate
7) Urine or Saliva Toxicology
8) Urine Pregnancy (if applicable; to be performed every two weeks)
9)

The following assessments will be administered as an interview at Baseline 2 and then entered directly into REDCap by the interviewer:

1) Concomitant Medications Form
2) Health Changes Questionnaire
3) Time Since Last Cigarette Questionnaire

The following assessments will be administered at Baseline 2 and completed by the participant directly in REDCap:

1) BDI
2) OASIS
3) COVID19 Symptom Questionnaire
4) Respiratory Symptom Questionnaire
5) MNWS
6) WISDM
7) PANAS
8) QSU (usual brand)
9) Vaping Craving Questionnaire
10) CES (usual brand)
11) Vaping Evaluation Scale
12) CPT (usual brand)
13) Snaith-Hamilton Pleasure Scale (SHAPS)
14) E-Cigarette flavor rating questionnaire
    a. Participants will rate only the flavors that they received from staff for this visit

In the event that the REDCap website is not functioning, the assessments will be administered aloud and participant answers will be recorded securely. The interviewer will enter the data into REDCap when it resumes functioning properly. This information should be recorded in the 'End of Visit Evaluation Form'.

1) for each picture as they appear on the screen.

6.3 E-cigarette Training Session (Baseline 2):

Participants assigned to an e-cig condition will be told that they will be provided with a JUUL. If a participant indicates an unwillingness to use the JUUL device, the research assistants will offer the participant the Vuse Solo as alternative device. If the participants does not wish to use either device, he or she would be ineligible for the study.

Participants in the e-cigarette conditions will be given e-cigarette pods BEFORE the training session occurs. These pods will be either picked up at the lab (preferred) or delivered to the participant 1 to 2 days before their Baseline 2 visit occurs. Participants randomized to the e-cigarette conditions will be notified before their Baseline 2 visit (but after the 7-day period following Baseline 1) and informed of their e-cigarette condition. Participants randomized to the flavored condition will be given pods of up to three flavors of their choice. Staff will calculate how many total pods participants will be given at this time based on their smoking rate, and participants will be able to choose the proportion of each flavor that they would like to receive.

The e-cigarette training session will occur over video chat after the physiological measurements have been collected. The first 30 minutes of the training session will consist of the participant being taught how to use, charge, and replace pods/cartridges for their e-cigarette of choice. Participants will first try their JUUL using the tobacco flavor, and then decide which device they would like to use for the duration of the study. If desired, participants are able to choose a preferred device to use without testing both e-cigarettes. At this point, participants who are in the tobacco-only flavor condition will conclude their training session.

For all visits following Baseline 2, participants in the preferred flavor condition are permitted to take up to three flavors home per week, but can choose to take less than three flavors if desired. Participants will take home their chosen pods or cartridges of up to three flavors until next visit. Participants will be able to change their flavors at only one point in the study if they desire. Participants will only be allowed to take home three flavors at one time. Participants will be given an E-cigarette instructional manual that reviews the e-cigarette training done at this visit. Participants will be encouraged to call with any device issues.

6.4. Interactive Voice Response System:

At the end of the first baseline visit, participants will be trained to use the Interactive Voice Response (IVR) System, which will contact participants each day throughout the study and ask about their smoking behavior as well as withdrawal symptoms the week before and after Baseline 2. We will also review the IVR adherence incentive program, which consists of $1 per call plus a $10 bonus for seven consecutive calls.

The IVR system is operated by TeleSage (https://telesage.com/about/). To be enrolled in the IVR system, research staff will enter the participant's initials, telephone number, subject identifier, and visit dates into the IVR TCORS website. Identifying information (initials and telephone numbers) will not be extracted as part of the data by the bioinformatics group. Please refer to TeleSage's privacy statement and HIPAA compliance form for additional information.

Baseline 2 biological specimens:

1) Urine sample for smoking biomarker assessment**:**
   Participants will be asked to provide a urine sample (first void of the day) at the second baseline session and to post-randomization weeks 8 and 16 for biomarker assessment. Biomarker analysis will provide nicotine and carcinogen exposure outcome measures and verify compliance with VLNC cigarettes. Samples will be stored at -80C. Urine samples will be analyzed for total nicotine (cotinine plus its glucuronide conjugate, a useful measure of daily nicotine exposure), the tobacco-specific nitrosamine 4-methylnitrosamineo-1-(3-pyridyl)-1-butanol (NNAL), and metabolites of 4 polycyclic aromatic hydrocarbons (PAHs), which are biomarkers of tobacco smoke carcinogens and decrease upon tobacco cessation or reduction. Anatabine is a minor alkaloid that is reduced in users of VLNC cigarettes and e-cigs. Therefore, anatabine levels in samples from those assigned to the VLNCC, VLNCC +TF e-cig and VLNCC+PF e-cig conditions should be lower than levels from those in the NNCC condition. These analyses will be performed by the Murphy lab at the University of Minnesota.
2) Saliva Samples for smoking biomarker assessment:
   We will collect a saliva sample at the second baseline session and post-randomization weeks 8 and 16 for analysis of nicotine metabolite ratio (NMR; ratio of 3-hydroxycotininine [3 HC] to cotinine [COT]), a phenotypic marker of nicotine metabolic rate. Analyses of these samples will be performed by the Tyndale lab.

Biomarker shipping and storage:

Biomarkers will be shipped quarterly to the University of Vermont Laboratory for Clinical Biochemistry Research (Tracy Lab). The Tracy Lab will serve as a central repository for all biomarker specimens and will be responsible for distributing specimens to the appropriate labs on a quarterly basis. Urine samples will be analyzed and stored at the University of Minnesota Murphy Lab. The saliva samples will be analyzed and stored at the University of Toronto Tyndale Lab.

7. Study Experimental Procedures

7.1 Experimental Period:

Participants will be seen weekly throughout the 16-week experimental period. Weeks 4, 8, 12, 16 and the abstinence visit will take approximately 2-4 hours each. All other sessions will last approximately 2 hours If a participant has a positive urine or saliva toxicology test or is visibly intoxicated as determined by slurred speech, swaying, or stumbling, the session will be rescheduled until a negative test result is obtained and intoxication is not present. As a part of each experimental visit, participants will be asked to come to UHC for a product exchange. All participants must pass a COVID19 screening before entering the building. Participants will be instructed to contact the RA at the office when they get to the clinic to ensure that there is enough space in the smoking chambers to house all participants while abiding by safety guidelines as detailed on page 14 of the protocol. All participants must pass a COVID19 screening before entering the building. When invited into the lab, the participant will be shown to a smoking

chamber and will instructed to place their bag of product outside of the chamber. The participant will wait here while the RA processes and returns product through the randomization database. Then the RA will dispense new product and bring the bag back to the participant's smoking chamber and leave it on the ground in front of the chamber. When the RA deems it safe for the participant to exit the chamber, the RA will instruct the participant that they can leave. The RA will instruct the participant to observe social distancing measures during this exchange, providing clarification if necessary. At the end of each experimental session, the researcher will complete the End of Visit Evaluation Form, which will be filed in the participant's binder. This will allow the researcher to make note of any problems encountered during the visit and to assess the truthfulness of the participant in regards to self-report of tobacco use and compliance to study procedures.

Visit scheduling requirements for experimental period:

The ideal scheduling window between each visit is 7 days based on the date of the Baseline 2 Visit. For additional scheduling requirements, refer to the '*Scheduling Visits SOP*'. If a participant misses a visit and is unable to reschedule during the window (± 3 days), that visit will not be 'made-up' in the future. All measures that were not completed will be considered missing data and will not be collected during future visits. If a visit mistakenly occurs outside of the designated window, this is a protocol deviation. A 'Non-Medical Event Form' will need to be completed. Additionally, each visit should occur at approximately the same time of day ± 2 hours.

If a participant is not able to attend his/her Week 16 visit, then it should be rescheduled even if it is outside of the scheduling window. This will be documented as a protocol deviation.

7.2 Experimental Visits Weeks 1, 3, 5, 7, 9, 11, 13, and 15 Procedures

7.2.A. Measures/Assessments

Physiological Measures Collected and entered directly into REDCap by the interviewer:

1) CO
2) Blood Pressure
3) Heart Rate
4) Body temperature
5) Oxygen saturation
6) Respiratory rate
7) Urine or Saliva Toxicology
8) Urine Pregnancy test (if applicable, to be performed every 2 weeks)

The following assessments will be administered as an interview and will be entered directly into REDCap by the interviewer:

1) <u>Concomitant Medications</u>
2) <u>Medical Event Form, if applicable</u>
3) <u>Health Changes Questionnaire</u>
4) <u>Time Since Last Cigarette Questionnaire</u>

The following assessments will be completed by the participant directly in REDCap:

1) <u>BDI</u>
2) <u>OASIS</u>
3) <u>COVID19 Symptom Questionnaire</u>
4) <u>Respiratory Symptom Questionnaire</u>
5) <u>MNWS</u>
6) <u>Snaith-Hamilton Pleasure Scale (SHAPS)</u>

In the event that the REDCap website is not functioning, the assessments will be administered aloud and participant answers will be recorded securely. The interviewer will enter the data into REDCap when it resumes functioning properly. This information should be recorded in the 'End of Visit Evaluation Form'.

7.3 Experimental Visits Weeks 2, 4, 6, 8, 10, 12, 14, and 16 Procedures:

7.3.A Measures/Assessments

Physiological measures collected and entered directly into REDCap by interviewer:

1) CO
2) Blood Pressure
3) Heart Rate
4) Body temperature
5) Oxygen saturation
6) Respiratory rate
7) Urine or Saliva Toxicology
8) Urine Pregnancy test (if applicable)

The following assessments will be administered as an interview and will be entered into REDCap by the interviewer at the end of the visit:

1) Concomitant Medications
2) Medical Event Form, if applicable
3) Health Changes Questionnaire
4) Time Since Last Cigarette Questionnaire

The following assessments will be completed by the participant directly in REDCap:

1) BDI
2) OASIS
3) COVID19 Symptom Questionnaire
4) Respiratory Symptom Questionnaire
5) MNWS
6) QSU (usual brand)
7) QSU (study cigarette)
8) Vaping Craving Questionnaire
9) CES (usual brand)
10) CES (study cigarette)
11) Vaping Evaluation Scale
12) PANAS
13) Cigarette Purchase Task - Usual Brand Cigarette Version (weeks 4, 8, 12 and 16 only)
14) Cigarette Purchase Task – Study Cigarette Version (weeks 4, 8, 12 and 16 only)
15) Cross-price Elasticity Task[109]- e-cigarettes and combustible cigarettes (weeks 4, 8, 12 and 16 only) (for e-cigarette experimental conditions only)
16) Penn State Electronic Cigarette Dependence Index (weeks 8 and 16 only)
17) Respiratory Health Questionnaire (weeks 8 and 16 only)
18) FTND (weeks 8 and 16 only)
19) Perceived Health Risks Questionnaire (weeks 8 and 16 only)
20) Smoking Stages of Change Algorithm and Contemplation Ladder (weeks 8 and 16 only)
21) WISDM – Brief Scale
22) Snaith-Hamilton Pleasure Scale (SHAPS)
23) Drug Use Questionnaire – 1 month version (weeks 8 and 16 only)
24) Perceived Stress Scale (weeks 8 and 16 only)
25) Alcohol Use Questionnaire – 1 month version (weeks 8 and 16 only)
26) E-cigarette flavor rating questionnaire (weeks 4, 8, 12 and 16 only)

In the event that the REDCap website is not functioning, the assessments will be administered aloud and participant answers will be recorded securely. The interviewer will enter the data into REDCap when it resumes functioning properly. This information should be recorded in the 'End of Visit Evaluation Form'.

Biological Samples to be collected:

1) First void urine sample (Weeks 8 and 16 only)
2) Saliva sample (Weeks 8 and 16 only)

7.4 Interactive Voice Response System:

Participants will continue to use the IVR system on a daily basis throughout the experimental period to record the number of study cigarettes smoked per day, measurement of e-cig use and use of non-study cigarettes or other tobacco products. Measurement of e-cig use will be collected by asking two questions: how many daily e-cigarette episodes occurred, where one episode consists of around 10-15 puffs or up to approximately 10 minutes, and what proportion of pods and/or cartridges were used per day. Participants will also be asked to log how many flavors of e- cigs they used per day. During the first week after Baseline 1, the IVR system will collect information about mood and withdrawal symptoms.

7.5 Variable Incentive Program:

An incentive program has been developed with the goal of improving attendance at scheduled assessment sessions, compliance with using only study-provided tobacco products, and encouraging honest self-reports regarding all nicotine/tobacco use.

Briefly, participants will receive a total of seven tickets for each weekly visit they attend after randomization (Visits 03-18, weeks 1-16). In total, participants could earn 112 valid tickets across the 16 visits. Participants will be instructed that these tickets correspond to attendance (one ticket), honest reporting (one ticket), compliance in bringing back used and unused pods/cartridges (two tickets) and adherence to using only the assigned study product (three tickets). Participants who do not bring back all of their unused study product and used packaging will be told that they may not be eligible to earn the two compliance tickets.

Participants will be further instructed that all of the tickets that they receive “could” be eligible for entry into a monthly drawing for prizes, but that only tickets that are “validated” will be eligible for prizes.

Since it is prohibitively expensive to test urine samples each week for each participant and because it is currently not feasible to detect with reasonable precision non-compliance based on biomarkers in the two higher nicotine group, we plan to only validate the attendance tickets. Hence, each participant who attends their regularly scheduled weekly session will have a total of seven validated tickets entered into the monthly drawing.

To convey the message that we may be validating honest reporting and use of only study-provided products, a bogus pipeline of sorts, we will tell the participants that a composite assessment of the measures that we collect MAY be used to validate the amount of nicotine and tobacco products that they are using. So there is some minor deception involved, but technically we could conduct urine toxicology testing for both purposes. Hence, if the urine toxicology testing is presented as something that MAY be done for validation purposes, we feel that any deception is relatively minor. For scientific/economic reasons we are just electing to restrict validation to attendance.

Nevertheless, we will debrief all participants upon the completion of the trial. We will inform them that the incentive program was based exclusively on attendance due to the relatively high cost of urine toxicology testing and other practical problems with shipping the urines for prompt testing.

Drawings will be conducted on the 1st of each month. Validation will be performed by staff who have no participant interaction and are not blind to condition. Any ticket drawn will be eligible for an incentive as the only true contingency is for attendance. There will be no mention of the basis for earning incentives (i.e., whether the ticket was for attendance, honesty, adherence). Participants will simply be informed that he or she earned an incentive from the drawing.

Each drawing will be independent (without replacement); consequently, some participants will not win a prize and others may win more than one during the study if more than one of their tickets is drawn. After confirming winners, the remaining tickets from each month will be discarded (i.e., tickets will only be entered into one drawing). The monthly prize amounts are

detailed below.

We estimate based on the 2 ½ years we think it will take to complete this study, that participants will win an average of approximately $65 in prizes or an additional $5.50 per week per participant.

Grand Prize (1): $500 cash Second Prize (1): $200 cash Third Prize (5): $10 cash

7.6 Product and Procedures Compliance Review Sessions:

At each visit, Baseline 2 through Week 16, participants will be counseled about their use of the study cigarettes and assigned e-cigarette (if applicable). Participants will be asked about any concerns or obstacles associated with use of the study cigarettes and assigned e-cigarette (if applicable). The importance of honest self-reporting will be stressed. Participants will be told that they will not be penalized for use of other nicotine or tobacco products and that it is crucial for them to report any use of these products. If difficulties are encountered, participants will be asked why they think they are experiencing difficulties (e.g., taste, withdrawal symptoms) and to problem-solve how to deal with these difficulties in order to meet the protocol requirements. Additionally, participants will be counseled about their IVR completion, visit attendance, task engagement and product accountability. Refer to the '*Product and Procedures Compliance Review Sessions SOP*' for more information.

7.7 Quit Attempts During the Study Protocol:

At each weekly session, we will ask each participant if s/he is currently abstaining from smoking with the intention of quitting and whether s/he is planning to quit smoking prior to his/her next scheduled visit. If a participant is currently abstaining from smoking with the intention to quit, we will encourage the participant to continue abstaining, schedule them for weekly visits, and provide them with NCI's Clearing the Air manual and local smoking cessation resources. We will give them the option of taking study product(s) home but not require that they take them, and if they do take the product(s) home we will suggest that they put the product(s) away at home so as to remove these cues from view. We will ask the participant to contact staff if they lapse and would like to receive study product(s) prior to his/her next visit. If a participant is planning to quit but has not initiated a quit attempt, we will ask if s/he has identified a quit date and if so what the date is, provide them with the Clearing the Air manual and local smoking cessation resources, provide them with the study product(s), and recommend that they put the product(s) away out of view on the quit date.

For those in a condition including e-cigarettes, we will defer to the participant's interests in continuing to use e-cigarettes as part of their quit attempt. Those who indicate that they will continue to use them will be given their same weekly supplies base on their baseline smoking rate. Those who indicate that they are planning to abstain from both combusted and non-combusted tobacco, we will honor that request. As we state above about combusted cigarettes, if participant changes his or her mind about resuming e-cigarette use, they can contact us and obtain their weekly supply.

7.7.A. If a participant is currently abstaining from smoking with the intention to quit:

- Encourage participant to continue abstaining from smoking
- Schedule the participant for normal weekly visits, but no puff topography
- Provide the participant with the '*Clearing the Air*' manual and local smoking cessation resources
- Give the participant the option to receive study product rather than require him/her to take the product
- If the participant choses to receive the study product have him/her sign a form acknowledging that cigarette availability could be detrimental to the quit attempt. Recommend that he/she put the product "away" at home as to avoid unwanted cues to smoke.
- If the participant chooses not to receive the study product, have him/her contact the lab if

he/she lapses and would like to pick up or be mailed the study product prior to his/her next visit.

7.7.B. If a participant is planning to quit smoking, but has not initiated the quit attempt:

- Ask if he/she has identified a target quit date and, if so, what that target date is
- Provide the participant with the '*Clearing the Air*' manual and local smoking cessation resources
- Provide the participant with the study product as usual. Recommend that on the target date he/she put the product "away" at home as to avoid unwanted cues to smoke.

7.8 Abstinence Assessment Session:

After the week 16 visit, participants will be required to attend one additional visit the following day. During this visit, participants will have been encouraged to abstain from smoking until their next scheduled visit (approximately 24 hours later). The abstinence assessment session should be scheduled no less than 18 hours and no more than 30 hours after the Week 16 visit. Abstinence will be verified by an expired breath carbon monoxide level of less than or equal to 6 parts per million (ppm). This session will allow us to determine whether the experimental cigarettes and e-cigarette use (for the e- cigarette conditions) have reduced the effects of abstinence on these measures relative to the control conditions. If the participant does NOT meet abstinence criteria, he/she will only receive $20 for the visit. Those who do meet abstinence criterion will do concurrent choice session detailed below.

7.8.A Participants Who Meet Criteria for Abstinence

7.8.A.1 Measures/Assessments

Physiological measures collected and entered directly into REDCap by the interviewer at the end of the visit:

1) CO
2) Blood Pressure
3) Heart Rate
4) Body temperature
5) Oxygen saturation
6) Respiratory rate
7) Urine or Saliva Toxicology

The following assessments will be administered as an interview and will be entered directly into REDCap by the interviewer:

1) Concomitant Medications
2) Medical Event Form, if applicable
3) Health Changes Questionnaire
4) Time Since Last Cigarette Questionnaire

The following assessments will be completed by the participant directly in REDCap:

1) BDI
2) OASIS
3) COVID19 Symptom Questionnaire
4) Respiratory Symptom Questionnaire
5) MNWS
6) PANAS
7) QSU-brief - Usual Cigarette
8) QSU-brief - Study Cigarette
9) Vaping Craving Questionnaire
10) Cigarette Purchase Task - Usual Brand Cigarette Version
11) Cigarette Purchase Task - Study Cigarette Version
12) E-cigarette Purchase Task- E-cigarette Version

13) Snaith-Hamilton Pleasure Scale (SHAPS)

In the event that the REDCap website is not functioning, the assessments will be administered aloud and participant answers will be recorded securely. The interviewer will enter the data into REDCap when it resumes functioning properly. This information should be recorded in the 'End of Visit Evaluation Form'.

7.8.B. Participants Who Do Not Meet Criteria for Abstinence

7.8.B.1 Measures/Assessments

Participants who do NOT meet abstinence criteria will be required to complete the following assessments:

1) CO
2) Blood Pressure
3) Heart Rate
4) Body temperature
5) Oxygen saturation
6) Respiratory rate
7) Urine or Saliva Toxicology

The following assessments will be administered as an interview and entered directly into REDCap by the interviewer:

1) Concomitant Medications
2) Health Changes Questionnaire
3) Medical Event Form, if applicable
4) TLFB

The following assessments will be completed by the participant directly in REDCap:

1) BDI
2) OASIS
3) COVID19 Symptom Questionnaire
4) Respiratory Symptom Questionnaire

7.9 Participant Compensation:

Participants will receive $25 plus a $25 bonus for completing each screening visit on time as scheduled. Payment for the first screening session will be made upon its completion. Payment for the second screening session will be made regardless of enrollment as long as the participant passes the drug test and meets the minimum requirements for carbon monoxide or urinary cotinine levels. Participants who do not pass the drug test or who are visibly intoxicated as determined by slurred speech, swaying, or stumbling will not be able to complete the visit and will be asked to take another test several days after the first positive. If they are negative for the second test, they will be eligible to participate, and if they are positive the for the second text they will be excluded. Participants will receive $100 for each study visit from Baseline 1 to Week 16. Participants will also have a chance to earn an additional $20 bonus for every study visit that is completed on time as scheduled starting at Week 1 and ending at Week 15.

Participants can receive up to $120.00 for the abstinence session ($20 if participant does not achieve abstinence, $120 if participant reaches abstinence), $40 for biochemical verification of abstinence at 30 day follow up visit, and up to $306 for completing daily IVR reports of study cigarette and other nicotine and tobacco use. There will also be a $150 bonus distributed at Week 16 for completing the study. Participants who do not complete the entire study will receive compensation for the sessions that they do complete. Total compensation for completing Study 3, including study visit payments, daily IVR calls and end of study bonus is $2816. As mentioned above, participants will have a chance to earn additional incentives for compliance, honesty and attendance through urine testing. Participants will be given a debit card at the beginning of the study (during the second portion of the screening visit) and compensation for

each visit will be automatically transferred to the card after they complete that visit. If debit cards are unavailable, participants will be paid via an alternate method (i.e. cash or check).

7.10 End of Study:

After a participant has completed all study procedures and has been paid for participation the research assistant will read the following script and give the participant the *Clearing the Air*

Manual.

"If you've reduced your smoking during this study, we encourage you to continue these reductions or even consider quitting. We would like to provide you with some resources should you decide to try to abstain from smoking (give "Clearing the Air" and hotline information). Please also feel free to consult with your physician and use any medications he/she deems appropriate. We will call you in approximately 30 days to ask about your smoking since leaving the study. There is no right answer and we know how difficult quitting can be. Please just answer honestly. The call will take less than 5 minutes. Thanks again for your participation."

The following assessments will be administered using REDCap:

1) End of Study Questionnaire

7.11 30 Day Follow up Phone Call:

Participants will receive a follow-up phone call between 25 and 35 days after the abstinence assessment session to assess their smoking patterns. The phone questionnaire will last less than five minutes. The questionnaire will ask if the participant is still smoking, how much and whether he/she has attempted to quit smoking since the end of the study. Participants will receive 5 variable incentive program lottery tickets for completing the call as compensation.

Those who report abstinence will be invited to complete biochemical verification and be compensated $40 for doing so. Abstinence will be achieved by a carbon monoxide reading of 6 parts per million (ppm) or under. A urine sample may also be collected to be sent to the lab for analysis. Additionally, any Medical Event Forms that remain open from the last session will be discussed. If the participant became pregnant during the study, this would have been recorded as a medical event. During this phone call, the research assistant will confirm her due date.

This event will remain open until delivery. At that time the licensed medical professional will contact the participant to ask a few questions about the baby's health and will update the Medical Event Form.

Once a participant has completed all study procedures and all open events have been closed, the PI or Project Manager will review the participant's record and sign a form indicating study completion for that participant.

8.0 Study Randomization

8.1 Randomization Process

The lead statistician will create a randomization schedule for each of the two sites, amounting to 150% of expected enrollment at each site. The excess randomization codes will be used in the event that a site will have to enroll extra participants due to unexpectedly slow enrollment at another site. The nicotine doses will be identified by letter code and only Administrative Core personnel with no participant contact will have the link between the statistician's letter code and dose assignments. There will be no blinding of e-cigarette conditions. The Administrative Core will maintain the randomization schedule and the link between the alphabetic code and treatment assignment securely. A second sealed copy will be secured in a separate building to protect against loss related to fire or other unforeseen events.

The University of Vermont will be responsible for removing all identifying information from cigarettes received from the Research Triangle Institute (RTI), labeling each carton with a blind code, assigning product using this blind code based on the randomization schedule being provided by the UVM Biostatistics Core, and shipping cigarettes and e-cigarettes to each site as needed based on recruitment. Each site will be responsible for tracking product received and distributed to participants, collecting unused product from participants, and returning unused cigarettes and e-cigarettes to UVM. The participants, investigators and study staff will not have knowledge of which product is given to a participant or whether different participants received the same or different product.

8.2 Study Product Administration

During the experimental period, participants will be provided with a 14-day supply of research cigarettes equivalent to 150% of their daily smoking rate. Those in the e-cigarette conditions will

also be provided with a 14-day supply of e-cigarettes equivalent to their daily smoking rate. This rate will be calculated at Baseline 2 and will be an average daily smoking rate based on the IVR data that reports on the usage for the first seven days following the day of the first baseline visit. This will ensure adequate availability of cigarettes in the numerous locations participants may typically keep a supply (home, work, vehicle, etc.) as well as avoid expending the entire supply if they miss a scheduled visit. Participants will be instructed to use the research cigarettes for 12 weeks, at which point they are to discontinue product use.

If there is prior knowledge a participant will be missing a visit (i.e. planned vacation, laboratory closure, etc.), then the participant will be provided with an adequate supply of cigarettes to make up for the missed visit(s). The participant will be given a 21-day supply if one visit is going to be missed and a 28-day supply if two visits are going to be missed.

8.3 Guidelines for Reporting other Nicotine Product Use

Participants will be asked to refrain from use of other non-study cigarettes during the study period. If participants have to use another nicotine product, they will be told to use a non-combustible product (gum, patch, etc.). Additionally, they will be told there is not a penalty for use of non-study products, and that it is crucial for them to report any use of non-study tobacco products. Throughout the baseline and experimental periods, an Interactive Voice Response (IVR) system will be used on a daily basis to record the number of study cigarettes and non-study cigarettes used the previous day. During the baseline and first experimental week, participants will also answer daily IVR questions about their mood. Participants will be seen weekly for assessments. Brief standardized review sessions focusing on compliance with the study cigarettes and other study procedures will be provided at each visit.

8.4 Product Accountability:

Participants will be required to keep track of all the products provided to them. Therefore, they will be instructed to return all unused products and empty cigarette packs e-liquid pods/cartridges to the laboratory each week. Research staff will complete the ‘Product Accountability Log’ as they process participants’ product. Any discrepancies in the product dispensed versus product returned will be discussed and recorded in the log. Research staff will weigh all opened e-cigarette pods/ cartridges that the participant returns at all visits to determine how much e-liquid was used since the participant was last seen. Empty cigarette packs and e-liquid pods/cartridges will not be saved. Unused cigarette packs and e-liquid pods/cartridges will be re-distributed to the participants during Weeks 1-15. During Week 16, remaining unused cigarettes and e-cigarette pods/cartridges returned by the participants will be collected by the research staff.

Participants who report running out of cigarettes or e-liquid pods/cartridges prior to a scheduled weekly visit will be allowed to come in for an unscheduled visit to obtain more research cigarettes. To determine whether a rate change for cigarettes is necessary, we will look at the past two CO levels as compared to the Baseline 2 CO. If the CO trend is consistent with the self-report of smoking all of the allotted cigarettes then a rate increase will be granted. The participant will then receive cigarettes at a rate of 175% of their daily smoking rate. The maximum increase is 200% of their daily smoking rate. To determine whether a rate change for e-cigarettes is necessary, we will monitor the amount of e-cigarette use the participant is reporting and showing through product return along with any unscheduled visits. The investigator may grant an e-cigarette rate increase in increments of 25%. The maximum increase for e-cigarettes will be 200% of their baseline weekly e-cigarette dispensation rate.

If participants lose more than two packs of cigarettes and/or pods/cartridges and require an unscheduled visit to the laboratory to supplement their supply, they will be told the next time they lose more than two packs they will have to wait until their next scheduled appointment to receive more cigarettes.

9. Study 3 Statistical Methods and Sample Size

9.1 Statistical Methods

Continuous outcomes will be summarized by mean, standard deviation, median and range. Categorical outcomes will be summarized by frequencies and percentages. Skewed continuous outcomes will be log- or square-root transformed as appropriate. Variables measured at each baseline visit will be averaged and the average will be used as the baseline measurement. As we expect conditions to be balanced on important baseline characteristics due to randomization, our primary analysis for all endpoints will not be adjusted for potential confounders. However, a secondary analysis will be completed for all outcomes adjusting for demographic characteristics (e.g., age), that we have found to be important in prior studies. Potential moderators (e.g., SSRI vs. non-SSRI antidepressant, depression vs. anxiety disorders, BMI above or below 30) will be explored by adding that term and the moderator-by-condition term to the model. We will examine age group and gender as potential moderators in a similar fashion.

Participants will be randomized in equal probability to one of the four conditions, with randomization stratified by site and menthol cigarette status. All analyses will follow the intent-to-treat principle (i.e. subjects will be analyzed according to condition assignment, regardless of compliance). The Primary Aim will examine the effects of condition on total CPD (study product and non-study product). CPD will be analyzed by week (mean over all days in a seven-day period) using a mixed model to account for repeated measures from the same individual.
Models will include baseline CPD as a covariate. Using a mixed model also allows us to include the effect of study site as a random effect. Additional analyses conducted using data collected at the end of the study will use orthogonal comparisons to test for a linear trend in the decrease in CPD, such that VLNCC + PF e-cig > VLNCC + TO e-cig > VLNCC > NNCC, with the largest reduction in the VLNCC + PF e-cig condition. As we expect that differences among conditions for some of the outcomes may not follow a linear pattern, we will use related planned comparisons to test for threshold effects, specifically contrasting the NNCC condition to all three VLNCC conditions, and NNCC to the two VLNC + e-cigarette conditions.. Analysis of cigarette demand, smoke exposure and tobacco carcinogens (Aim 2) as well as additional outcomes, including subjective effects, will be analyzed in a similar manner. Because Exploratory Aim 3 is based on abstinence-induced effects and will be examined using data collected at a single visit at Week 17, analysis will be based on an analysis of co-variance model. In addition to the effect of condition, we will include important covariates noted above. Exploratory analyses will also be conducted combining data collected from three of the vulnerable populations (disadvantaged women of childbearing age, opioid dependent individuals, individuals with AD) to explore potential differences in effects of study condition across these populations. This will be done with the addition of the effects of population and population-by-condition terms to the models described above. Study staff will make every effort to minimize missing data, and results of our ongoing trial suggest that this will be minimal. We will examine the missing data pattern, and if it is missing at random, will use all data available, without imputation.

permutation tests.[121]

9.2 Sample Size

Sample size was determined using NQuery Advisor based on hypothesis tests related to Aim 1, specifically to detect a significant difference between the study conditions (NNCC, VLNCC, VLNCC + TO e-cigs, VLNCC + PF e-cigs) on total cigarettes per day (CPD). The primary statistical approach will be repeated measures ANOVAs but required sample sizes were calculated focusing on expected outcomes at Week 16. This sample size estimate is intentionally conservative and calculated based on one outcome at one time point; however, given the repeated measures nature of our data, we will have correlated observations within subjects. Thus, with the given sample sizes we will achieve the stated power to detect differences of even lesser magnitude than stated or planned. Our sample size determination is based on preliminary results from our current trial and results from a large randomized clinical trial of

Table 2. Observed effect sizes

Outcome	Observed between-group ES (15.2 vs. 0.4 mg/g)
CPD	0.81
Craving	0.20
FTND	0.42
Breath CO	0.33

cigarettes varying in nicotine content.[7] Note that the between-group effect size is defined as the difference of study condition means divided by the common standard deviation. A sample size of 53 participants per condition will provide 80% power to detect an effect size of 0.60 for all pair-wise comparisons, with a two-sided type 1 error rate of 0.05. This is smaller than that found in our on-going study to date for CPD and smaller than the effect sizes reported by Hatsukami et al.[8] for all measures except breath CO. In addition, this sample size provides greater thn 95% power to detect a linear dose-response effect across the four experimental conditions. Becase we have relied only on outcomes at Week 16, our proposed sample sizes are somewat conservative, but we believe this is appropriate for this study given that the effects of VLNC cigarettes in this population, particularly in combination with e-cigarettes, are completely unknown. The sample size above assumes a 15% loss to follow-up, consistent with our experience in the current study. We will increase our overall sample size to 232 in order to allow pilot testing in a group of 20 participants.

10. Potential Risks of Participation

10.1 Risks of Participation

1) Survey Questionnaires. This interview will include questions about your medical and psychiatric histories, drug and alcohol use and history, breath tests for cigarette and alcohol use, urine or salivary tests of illicit drug use and pregnancy, and questionnaires about your mood. Answering these personal questions could make you uncomfortable. If you report thoughts of killing yourself or other indicators of suicidality, a study clinician will come to talk to you. You may also request to see a study clinician if you are in discomfort and would like help and/or referrals for mental health resources.
2) Breach of Confidentiality: The risk of the interview is loss of privacy if other people find out the results.
3) Undue Influence: Undue influence is a possible risk due to monetary compensation for participating in these studies. The likelihood of this risk is low because the compensation is commensurate with the amount of time and effort required for these studies.
4) Drug Testing: A breach of confidentiality could occur and other people could learn of the participant's drug use.
5) Obtaining Blood Pressure and Heart Rate. The blood pressure cuff may cause minimal discomfort. In obtaining blood pressure we may find a participant to have abnormal blood pressure and/or heart rate. If participant's blood pressure is abnormal, we will inform the participant of this, and participant may be advised to see a doctor, and may also be contacted by our study doctor. Also, smoking and nicotine can affect the cardiovascular system, which may result in changes in blood pressure and/or heart rate.
6) Smoking Cigarettes: All cigarettes are detrimental to a person's health and can lead to significant medical problems including:
   a. Cardiovascular Diseases: Coronary heart disease, heart attack, stroke, peripheral vascular disease, reduced blood circulation, abdominal aortic aneurysm
   b. Respiratory Diseases: Emphysema, bronchitis, and chronic airway obstruction Cancers: Cancer of the lung, bladder, cervix, esophagus, kidney, larynx, mouth, pancreas, throat, and stomach; leukemia
   c. Metabolic Diseases: Type 2 Diabetes
   d. Other Health Risks Associated with Smoking: Including but not limited to infertility, lower bone density in postmenopausal women, and hip fracture in women
   e. Death
7) Smoking Study Cigarettes. All cigarettes are harmful to a person's health and can lead to cardiovascular (heart) disease, respiratory (lung) disease, cancer and other health problems. In addition to the above medical problems, you may experience some minor negative health effects such as headaches. You may also experience smoking withdrawal symptoms, which are listed below. In addition, due to the altered nicotine

levels, there could be a change in your use of cigarettes including the manner in which you inhale the smoke. Smoking the study cigarette does not necessarily provide any less risk than your usual brand of cigarette and could pose increased health risks.

8) Using Study E-cigarettes. E-cigarettes are devices that heat nicotine to produce an aerosol. The health effects of e-cigarettes are still unclear, but appear to be less than that for tobacco cigarettes. Most e-cigarette users have lower nicotine levels than when they smoked regular cigarettes. Some e-cigarette users, especially those who use both e-cigarettes and regular tobacco cigarettes as well as youth and young adults, can have increased nicotine levels. In some rare cases, these use patterns have been associated with seizures. Whether this would occur with the concurrent use of very low nicotine cigarettes is unclear. E-cigarettes users very often maintain addiction to nicotine, but this addiction appears to be somewhat less than that from tobacco cigarettes. Abruptly quitting e-cigarettes could cause withdrawal symptoms similar to those from quitting tobacco cigarettes (see below) but slightly less severe. The most common side effects include dry mouth, irritation of the throat and mouth, and mild cough. The JUUL and Vuse e-cigarettes we will be providing have not been well-studied but appear to be of similar risk to other e-cigarettes. You may have heard that e-cigarettes, or "vapes," can explode and seriously injure people. Although they appear rare, these explosions are dangerous. The exact causes of these incidents are not yet clear, but some evidence suggests that battery-related issues may lead to vape explosions. In order to prevent e-cigarette related injuries, keep your e-cig away from other metal objects, never charge your e-cig with a phone or tablet charger, don't charge your e-cig overnight or leave it charging unattended, and stop using the e-cig if the batteries get damaged or wet. Always keep e-cig liquid out of kids' and pets' reach and sight after use. If we learn about additional risks of e-cigarettes during the study, we will inform you of these risks.

Mood and Psychiatric Symptom Changes. You may experience smoking withdrawal symptoms during this study. These symptoms can include anger, anxiousness, craving for a cigarette, depressed mood, difficulty concentrating, frustration, increased appetite, impatience/impulsivity, irritability, restlessness, sleep problems, and weight gain. These feelings can be uncomfortable and can last a couple of weeks, but usually are of minimal risk. In addition, if you have a past history of anxiety, depression, or alcoholism, it is possible withdrawal could cause substantial increases in depression and anxiety symptoms, but this appears to be rare. At each visit, we will ask you how you feel. If you or we think that being in this study is putting your mental health at risk, we may have you meet with an on-site clinician and/or stop participating in the study. Further, if you report thoughts of killing yourself or other indicators of suicidality, a study clinician will come to talk to you. You may also request to see a study clinician if you are in discomfort and would like help and/or referrals for mental health resources.

9) Returning to Regular Smoking: It is possible that if participants return to smoking their usual brand of cigarette at the end of the study they may experience mild and transient nausea, dizziness, and lightheadedness.

10) Risk to Fetus. To avoid risks to a fetus, it is important that you are not pregnant during this study. Avoiding sexual activity is the only certain method to prevent pregnancy. However, if you choose to be sexually active, you should be using approved forms of birth control if applicable determined by the Project Medical Director, including but not limited to prescribed birth control pills, patch, ring, injections, implants or intrauterine device (IUD) or an appropriate "double barrier" method. If you choose to be sexually active during this study, pregnancy could still result even with the use of these birth control methods.

10.2 Avoiding Risks to Fetus:

If participants choose to be sexually active, they should use an appropriate "double barrier" method of birth control (such as female use of a diaphragm, or contraceptive sponge, in addition

to male use of a condom) or the female should be using prescribed “birth control” pills, patch, ring, injections, implants or intrauterine device (IUD) if applicable determined by the Project Medical Director. If a participant endorses a “double barrier” method, our medical professional will speak to the participant to confirm which methods will be used during the duration of the study. Participants will be tested for pregnancy every two weeks beginning at screening through the last study visit. If a participant becomes pregnant during the study, she will be withdrawn from the study. Approximately 30 days after being withdrawn or having a positive pregnancy test at the last study visit, the research staff will call the participant to confirm her due date. The licensed medical professional will follow-up with the participant after delivery to ask questions about the baby’s health.

10.3 Expected benefits of participation:

There are no benefits from participating in the study. The information obtained from this study may ultimately help the Food and Drug Administration decide how best to regulate nicotine and tobacco products with the goal of improving public health.

11. Protection Against Risk

11.1 Data Collection Protections

Research data without identifiers will be maintained in a locked file cabinet and on password-protected computers in the research staff workplace, with only code numbers identifying subjects. Study consent forms and the linkage between the participants’ names and codes will be stored in a locked file cabinet inside a locked office. Interviews with participants will be conducted in private rooms. Urine or saliva samples for drug and pregnancy tests and tobacco exposure biomarkers will be obtained in a private bathroom within the laboratory suite.

Subjective measures will be administered electronically. The biostatistics and data-management team will provide consistent data-management practices for all data in the Center. Using REDCap, which is housed on the University of Vermont Medical Center’s HIPAA-compliant computing system, will maximize validity and reliability of data. REDCap is a secure, web-based system that accommodates local and remote data collection by each project team, and allows for data entry work-flow monitoring and data quality control monitoring by biometry staff. The RedCap database for this project will be hosted on the UVMCOM servers. In addition, data will be collected from participants on a daily basis using an interactive voice recognition system (IVR) developed and hosted by TeleSage Inc (www.telesage.com, Chapel Hill, NC). TeleSage is a company with expertise on gathering patient-centered outcomes tracking data for mental health clinical and research institutions. TeleSage has developed and hosted behavioral health-related research software systems using IVR and Web-based technologies and is leader in behavioral health outcomes tracking technologies.For data integrity, data entry windows will follow the structure of paper forms as much as possible to allow for ease of entry, and will use predefined choices to minimize errors when possible. Data quality monitoring will be facilitated with periodic down loads and analysis using a variety of common statistical program format such as SAS, Stata, R, and SPSS. Quality control procedures will be conducted for all data collected, including analysis of missing data and logic checks for out of range and other anomalous values. This secure electronic data gathering and transmission plan, overseen by the experienced biostatistical team, will minimize opportunities for breaches of confidentiality. Biological samples for nicotine and carcinogen biomarker analysis will be marked with participant ID, stored in the locked laboratory suite, and sent to a laboratory for analysis on a quarterly basis.

All information collected as part of this study will be accessible only to research staff. No information will be shared with participants’ clinicians unless the participant requests this in writing. All investigators and staff have undergone (and any new staff will undergo) human subjects’ ethics training as required by UVM and are fully conversant with relevant ethical principals around confidentiality. Assessments, consenting and study procedures will be closely supervised by the PI.

The sponsors (NIDA/FDA) as well as the Institutional Review Board and regulatory authorities could be granted direct access to original medical and research records for verification of clinical trial procedures and/or data. If this is required, it will be done under conditions that will protect privacy to the fullest extent possible consistent with laws relating to public disclosure of information and the law-enforcement responsibilities of the agency.

11.2 Data Storage:

Data will be stored locally at each site. Long-term storage of all study data, for at least 7 years after study completion, will be at the University of Vermont.

12. Adverse Events

The research assistant will ask about adverse events at each session, using a form that assesses the nature, severity, duration, action taken, and outcome of study-related adverse events. AEs will be captured from the time of first study cigarette. Participants will be given contact cards to inform us of events that occur between study contacts. Any AE that remains open will be reviewed and closed at an interview conducted 30 days after the study completion date (completers) or when the study should have ended had the participant completed the study (dropouts and those withdrawn by investigator).

All procedures will be monitored to ensure that they conform to the approved protocol. In addition, monitoring will be done of all unforeseen circumstances that might arise and affect safety; of all reports of serious adverse events as defined in 38 CFR 46 (death, new or prolonged hospitalization, persistent or significant disability/incapacity); of other significant adverse events (adverse events that lead to drop out by the participant or termination by the investigator); of unexpected adverse events resulting from the study, and of expected adverse events.

Any SAE will be brought to the attention of the site PIs as soon as possible and not longer than 24 hours. Any AE or SAE that is both unexpected and related to study participation will be reported to the IRB within 7 days of the event. The local IRB will make a determination as to whether additional reporting requirements are needed. IRB actions will be reported to the funding agency by the PIs no less than annually and more frequently as recommended by the local IRB. Any SAEs will be summarized in the yearly Progress Reports to the funding agency, including a review of frequency and severity. All SAEs will be followed through ongoing consultation with the physician caring for the patient until they resolve, result in death, or stabilize and are not expected to improve. The study staff will be in close contact with participants and health care providers throughout the study to monitor for potential unanticipated problems. Any unanticipated problems will be discussed at the weekly research staff meetings and reported as required to the local IRB.

13. Withdrawal or Monitoring of Participants

For the participant's protection, participants will be withdrawn immediately from the study if any of the following occur:

1) <u>Cardiovascular disease (CVD) event</u>: Typically includes MI (heart attack), PTCA (angioplasty/stenting), bypass surgery, stroke, peripheral vascular disease (arterial blockages in arms or legs leading to procedure or surgery). Less common CVD problems would be new cardiac arrhythmias (e.g., new atrial fibrillation) or new valvular disease (e.g., mitral or aortic regurgitation).
2) <u>DVT/PE</u> (deep vein thrombosis/pulmonary embolism, i.e., blood clots in the venous system).
3) <u>Suicide Attempt</u>: A participant will be withdrawn if he/she attempts suicide at any time during participation in the study.
4) <u>Psychiatric Hospitalization</u>: A participant will be withdrawn if he/she is hospitalized for psychiatric reasons at any time during participation in the study.

5) Pregnancy: If participant indicates she is pregnant or has a positive pregnancy test at any time during the study, she will be withdrawn from the study, and this event will remain open until delivery. At that time the licensed medical professional will contact the participant to ask a few questions about the baby's health and will update the open 'Medical Event Form'. A positive pregnancy test at Week 16 will trigger a 'Medical Event Form' to be completed but will not result in withdrawal since she is no longer receiving study product.
6) Expired breath carbon monoxide increase: A participant will be withdrawn from the study if the average of two consecutive CO readings during the same visit is 100 ppm or greater.
7) Marked increase in smoking: A participant will be withdrawn from the study if he/she meets **BOTH** of the following criteria for two consecutive weeks
    a. Cigarette per day increase: The average CPD increases by more than 100% from the average CPD during baseline.
    b. Expired breath carbon monoxide increase: If the average of two consecutive CO measurements in the same visit is
        i. CO is greater than 50 ppm if CO at Baseline 1 is <20 ppm.
        ii. CO is greater than 60 ppm if CO at Baseline 1 is 20 – 34 ppm.
        iii. CO is greater than 70 ppm if CO at Baseline 1 is 35 – 49 ppm.
        iv. CO is greater than 80 ppm if CO at Baseline 1 is 50 – 64 ppm.
        v. CO is greater than 90 ppm if CO at Baseline 1 is 65 – 80 ppm.
    c. Note: If the second consecutive visit is the last study visit, then the participant would not be withdrawn from the study.
8) If a participant is discharged from or discontinues his or her methadone or buprenorphine treatment, they will be discontinued from the study.

The following will be monitored and can lead to the participant being withdrawn by the PI or Licensed Medical Professional:

1) Cigarettes per day increase: Continued participation will be evaluated by the site PI if the average number of cigarettes per day (CPD) increases by more than 100% from the average CPD during baseline as determined by CPD at Baseline 2.
2) Blood pressure (BP) or heart rate (HR) changes: If any of the following occur post-enrollment: 1) BP is at or above 160/100 or below 90/50, or 2) HR is at or above 115 bpm or below 45 bpm a manual blood pressure and heart rate measurement will be taken after 10 minutes have passed. If the manual reading is still out of range, a 'Blood Pressure and Heart Rate Symptom Checklist' and 'Medical Event Form' will be completed, and the participant will be monitored by the medical professional.
3) Expired breath Carbon Monoxide increase: If the average of two consecutive CO measurements meets the criteria below then the 'Medical Event Form' will be completed and the participant will be monitored by the medical professional.
    a. CO is greater than 50 ppm if CO at Baseline 1 is <20 ppm.
    b. CO is greater than 60 ppm if CO at Baseline 1 is 20 – 34 ppm.
    c. CO is greater than 70 ppm if CO at Baseline 1 is 35 – 49 ppm.
    d. CO is greater than 80 ppm if CO at Baseline 1 is 50 – 64 ppm.
    e. CO is greater than 90 ppm if CO at Baseline 1 is 65 – 80 ppm.
4) Any hospitalization or debilitation in which participation in the study could be detrimental to the recovery process. This will be self-reported by the participant and will be reviewed by the site PI and medical professional to determine whether continued participation in the study is appropriate.
5) If a participant is behaving in an inappropriate or threatening manner, admits to lying about eligibility criteria, is participating in other smoking research studies that could affect the primary outcome measures, etc., then the PI can withdraw him/her from the study at the PI's discretion.
6) If a participant fails to attend regularly scheduled research assessment visits or comply with the research procedures or schedule, then the PI can withdraw him/her from the study at the PI's discretion.

7) Increase in psychiatric symptoms: Exacerbation in symptoms noted during the study (i.e., change in BDI category from mild to moderate or moderate to severe) will trigger review by the study's licensed medical professional. The PI will withdraw the participant upon the licensed medical professional's recommendation.

14. Data Safety Monitoring Board

A Data and Safety Monitoring Board (DSMB) has been established to monitor safety outcomes and will be comprised of four members. The DSMB will be chaired by Kevin Delucchi, PhD., Professor in Residence of Biostatistics in Psychiatry at the University of California San Francisco and Director of the Quantitative Core of the San Francisco Treatment Research Center; Eden Evins, MD, MPH., Cox Family Professor of Psychiatry at Harvard Medical School and Director of Center for Addiction Medicine at Massachusetts General Hospital; Ari Kirshenbaum, PhD, Professor of Psychology at Saint Michael's College who he teaches courses in psychopharmacology and neuroscience, and currently has grants from NIH and NSF for work in human behavioral pharmacology and his grant-funded work focuses on cognitive and behavioral responses to nicotine and cannabinoids; and Elisabeth Johnson, Ph.D., who has over twenty years of clinical experience in women's health and pediatrics, including caring for women with substance use disorders.

Conflict of Interest

None of the board members will be otherwise affiliated with the center and each member will complete a conflict of interest disclosure form prior to each meeting. Ad hoc specialists may be invited to participate as non-voting members at any time if additional expertise is desired.

Monitoring Activities and Frequency of Meetings

The DSMB will set their own agenda and decisions about monitoring; e.g. how frequently to monitor, what threshold requires changes to protocol or stopping the study, and whether to view raw or analyzed data. The DSMB will be given FDA and EMEA guidelines for DSMBs and recent reviews on DSMBs. A brief report will be generated from each meeting for the study record and forwarded to each of the study site's Institutional Review Boards (IRB) and NIDA's Program Officer with the progress report. The DSMB will be available to convene outside of the regular meetings, if necessary. If concerns should arise regarding a particular subject, or any troublesome trends in the experiences of participants, they will make appropriate recommendations for changes in protocol, as needed. The project investigators will continue to examine safety data, blind to study condition, in case they wish to make study modifications. Before modifications are made, they will inform the DSMB and request their comments.

Communication Plan to IRB, NIDA, and FDA (if applicable)

All IRBs, the FDA and the NIDA's Program Officer will be informed of any significant action taken as a result of the Data and Monitoring Board's findings. Study Participants will be informed of any changes in risk.

Protection of Confidentiality

For DSMB meetings only de-identified data, including blinded study site and condition type, will be provided to the board. All data and discussion during the meeting will be confidential.

15. Investigational Tobacco Product

The University of Vermont Center on Tobacco Regulatory Science will complete an Investigational Tobacco Product (ITP) application with the FDA to cover the experimental cigarettes being used in this study. This application encompasses both Project 3 sites.

16. Certificate of Confidentiality

To help protect the participant's privacy, Dr. Stephen Higgins, PhD, will obtain a Certificate of Confidentiality from the national Institute on Drug Abuse. With this certificate, the researchers cannot be forced to disclose information that may identify the participants, even by a court subpoena, in any federal, state, or local civil, criminal, administrative, legislative, or other proceedings. The researchers will use the Certificate to resist any demands for information that would identify the participants, except as explained below. The Certificate cannot be used to resist a demand for information from personnel of the United States Government that is used for auditing or evaluation of federally funded projects or for information that must be disclosed in order to meet the requirements of the Federal Food and Drug Administration (FDA).

The Certificate of Confidentiality does not prevent the participant or a member of their family from voluntarily releasing information about themselves and their involvement in the research. If an insurer, employer or other person obtains the participant's written consent to receive research information, then the researcher may not use the Certificate to withhold that information.

The Certificate of Confidentiality does not prevent the researchers from disclosing voluntarily, without consent, information that would identify the individual as a participant of the research project in instances such as evidence of child abuse or a participant's threatened violence to self or others.

17. Outcome Variables

Primary Endpoints for Study 3:

1) Cigarette Smoked per Day (CPD)
2) Nicotine Dependence Severity

Secondary Endpoints for Study 3:

1) Measures of adherence: non-study cigarette use, drop-out rate
2) Measures of psychiatric symptoms: BDI, OASIS
3) Measures of discomfort/dysfunction: MNWS, QSU
4) Measures of other health-related behaviors: breath alcohol, urine or salivary drug screen, TLFB-drug use, Alcohol Use Questionnaire, Drug Use Questionnaire, weight
5) Measures of nicotine/tobacco dependence: FTND, WISDM
6) Measures of tobacco exposure: CO, total nicotine equivalents, NNN, NNAL, minor alkaloids
7) Measures of intention to quit: Stages of Change, Contemplation Ladder
8) Measures of compensatory smoking: puff topography, filter analysis
9) Measures of other tobacco use: TLFB-other tobacco
10) Measures of cigarette characteristics: CES, Cigarette Purchase Task
11) Measures of cardiovascular function: heart rate, blood pressure, urine 11-dehydroTXB2
12) Measures of perceived risk: Perceived Health Risk Questionnaire
13) Safety outcome variables: Adverse Events (AEs), Serious Adverse Events (SAEs)

References

1. Smith PH, Mazure CM, McKee SA. Smoking and mental illness in the U.S. population. Tob Control. 2014;23(e2):e147-153.
2. Benowitz NL, Henningfield JE. Establishing a nicotine threshold for addiction. The implications for tobacco regulation. N Engl J Med. 1994;331(2):123-125.
3. Benowitz NL, Henningfield JE. Reducing the nicotine content to make cigarettes less addictive. Tob Control. 2013;22 Suppl 1:i14-17.
4. Zeller M, Hatsukami D, Strategic Dialogue on Tobacco Harm Reduction G. The Strategic Dialogue on Tobacco Harm Reduction: a vision and blueprint for action in the US. Tob Control. 2009;18(4):324-332.
5. Benowitz NL, Hall SM, Stewart S, Wilson M, Dempsey D, Jacob P, 3rd. Nicotine and carcinogen exposure with smoking of progressively reduced nicotine content cigarette. Cancer Epidemiol Biomarkers Prev. 2007;16(11):2479-2485.
6. Benowitz NL, Dains KM, Hall SM, Stewart S, Wilson M, Dempsey D, Jacob P, 3rd. Smoking behavior and exposure to tobacco toxicants during 6 months of smoking progressively reduced nicotine content cigarettes. Cancer Epidemiol Biomarkers Prev. 2012;21(5):761-769.
7. Donny EC, Denlinger RL, Tidey JW, Koopmeiners JS, Benowitz NL, Vandrey RG, al'Absi M, Carmella SG, Cinciripini PM, Dermody SS, Drobes DJ, Hecht SS, Jensen J, Lane T, Le CT, McClernon FJ, Montoya ID, Murphy SE, Robinson JD, Stitzer ML, Strasser AA, Tindle H, Hatsukami DK. Randomized trial of reduced-nicotine standards for cigarettes. N Engl J Med. 2015 Oct;373(14):1340-9.
8. Hatsukami DK, Kotlyar M, Hertsgaard LA, Zhang Y, Carmella SG, Jensen JA, Allen SS, Shields PG, Murphy SE, Stepanov I, Hecht SS. Reduced nicotine content cigarettes: effects on toxicant exposure, dependence and cessation. Addiction. 2010;105(2):343- 355.
9. Higgins ST, Heil SH, Sigmon SC, Tidey JW, Gaalema DE, Stitzer ML, Durand H, Bunn JY, Priest JS, Arger CA, Miller ME, Bergeria CL, Davis DR, Streck JM, Zvorsky I, Redner R, Vandrey R, Pacek LR. Response to varying the nicotine content of cigarettes in vulnerable populations: an initial experimental examination of acute effects. Psychopharmacology (Berl). 2017;234(1):89-98.
10. Higgins ST, Heil SH, Sigmon SC, Tidey JW, Gaalema DE, Stitzer ML, Durand H, Bunn JY, Priest JS, Arger CA, Miller ME, Bergeria CL, Davis DR, Streck JM, Reed DD, Skelly JM, Tursi L. Addiction potential of reduced nicotine content cigarettes in populations with psychiatric disorders and other vulnerabilities to tobacco addiction. JAMA Psychiatry. 2017;in press.
11. US Department of Health and Human Services. The Health Consequences of Smoking—50 Years of Progress: A Report of the Surgeon General. Atlanta: U.S. Department of Health and Human Services, Centers for Disease Control and Prevention, National Center for Chronic Disease Prevention and Health Promotion, Office on Smoking and Health, 2014.
12. Cook BL, Wayne GF, Kafali EN, Liu Z, Shu C, Flores M. Trends in smoking among adults with mental illness and association between mental health treatment and smoking cessation. JAMA. 2014; 311(2):172-82
13. Center for Behavioral Health Statistics and Quality. Behavioral health trends in the United States: Results from the 2014 National Survey on Drug Use and Health (HHS Publication No. SMA 15-4927, NSDUH Series H-50). 2015. Retrieved from http://www.samhsa.gov/ data/
14. Grant BF, Hasin DS, Chou SP, Stinson FS, Dawson DA. Nicotine dependence and psychiatric disorders in the United States: results from the national epidemiologic survey on alcohol and related conditions. Arch Gen Psychiatry. 2004;61(11):1107-1115.
15. Prochaska JJ, Rossi JS, Redding CA, Rosen AB, Tsoh JY, Humfleet GL, Eisendrath SJ, Meisner MR, Hall SM. Depressed smokers and stage of change: implications for treatment interventions. Drug Alc Depend. 2004;76(2):143-151.
16. Young-Wolff KC, Fromont SC, Delucchi K, Hall SE, Hall SM, Prochaska JJ. PTSD symptomatology and readiness to quit smoking among women with serious mental illness. Addict Behav. 2014;39(8):1231-4.
17. Cooper J, Borland R, McKee SA, Yong HH, Dugué PA. Depression motivates quit attempts but

predicts relapse: differential findings for gender from the International Tobacco Control Study. Addiction. 2016;111(8):1438-47.

18. Hitsman B, Papandonatos GD, McChargue DE, DeMott A, Herrera MJ, Spring B, Borrelli B, Niaura R. Past major depression and smoking cessation outcome: a systematic review and meta-analysis update. Addiction. 2013;108(2):294-306.
19. Callaghan RC, Veldhuizen S, Jeysingh T, Orlan C, Graham C, Kakouris G, Remington G, Gatley J. Patterns of tobacco-related mortality among individuals diagnosed with schizophrenia, bipolar disorder, or depression. J Psychiatr Res. 2014;48(1):102-110.
20. Goodwin RD, Lavoie KL, Lemeshow AR, Jenkins E, Brown ES, Fedoronko DA. Depression, anxiety, and COPD: the unexamined role of nicotine dependence. Nicotine Tob Res. 2012;14(2):176-183.
21. Leventhal AM, Zvolensky MJ. Anxiety, depression, and cigarette smoking: a transdiagnostic vulnerability framework to understanding emotion-smoking comorbidity. Psychol Bull. 2015;141(1):176- 212.
22. Watson D. Differentiating the mood and anxiety disorders: a quadripartite model. Annu Rev Clin Psychol. 2009;5:221-247.
23. Taylor G, McNeill A, Girling A, Farley A, Lindson-Hawley N, Aveyard P. Change in mental health after smoking cessation: systematic review and meta-analysis. BMJ. 2014;348:g1151. Erratum in: BMJ. 2014;348:g2216.
24. Shiffman S, Patten C, Gwaltney C, Paty J, Gnys M, Kassel J, Hickcox M, Waters A, Balabanis M. Natural history of nicotine withdrawal. Addiction. 2006;101(12):1822-1832.
25. Weinberger AH, Desai RA, McKee SA. Nicotine withdrawal in U.S. smokers with current mood, anxiety, alcohol use, and substance use disorders. Drug Alcohol Depend. 2010;108(1-2):7-12.
26. Garey L Bakhshaie J, Brandt CP, Langdon KJ, Kauffman BY, Schmidt NB, Leventhal AM, Zvolensky MJ. Interplay of dysphoria and anxiety sensitivity in relation to emotion regulatory cognitions of smoking among treatment-seeking smokers. Am J Addict. 2016;25(4):267-274.
27. Pang RD, Khoddam R, Guillot CR, Leventhal AM. Depression and anxiety symptoms moderate the relation between negative reinforcement smoking outcome expectancies and nicotine dependence. J Stud Alcohol Drugs. 2014;75(5):775-80.
28. Weinberger AH, George TP, McKee SA. Differences in smoking expectancies in smokers with and without a history of major depression. Addict Behav. 2011;36(4):434- 437.
29. Wise RA. Dopamine and reward: the anhedonia hypothesis 30 years on. Neurotox Res. 2008;14(2- 3):169-83.
30. Brody AL, Olmstead RE, Abrams AL, Costello MR, Khan A, Kozman D, Saxena S, Farahi J, London ED, Mandelkern MA.. Effect of a history of major depressive disorder on smoking-induced dopamine release. Biol Psychiatry. 2009;66(9):898-901.
31. Audrain-McGovern J, Wileyto EP, Ashare R, Cuevas J, Strasser AA. Reward and affective regulation in depression-prone smokers. Biol Psychiatry. 2014;76(9):689-697.
32. Spring B, Pingitore R, McChargue DE. Reward value of cigarette smoking for comparably heavy smoking schizophrenic, depressed and nonpatient smokers. Am J Psychiatry. 2003;160(2):316-322.
33. Chaudhri N, Caggiula AR, Donny EC, Palmatier MI, Liu X, Sved AF. Complex interactions between nicotine and nonpharmacological stimuli reveal multiple roles for nicotine in reinforcement. Psychopharmacology (Berl). 2006;184(3-4):353-366.
34. Donny EC, Chaudhri N, Caggiula AR, Evans-Martin FF, Booth S, Gharib MA, Clements LA, Sved AF. Operant responding for a visual reinforcer in rats is enhanced by noncontingent nicotine: implications for nicotine self-administration and reinforcement. Psychopharmacology (Berl). 2003;169(1):68-76.
35. US Congress. Family Smoking Prevention and Tobacco Control Act. In: Congress US (ed) H.R. 1256 U. S. Government Printing Office, Washington, D.C., 2009.
36. Donny EC, Hatsukami DK, Benowitz NL, Sved AF, Tidey JW, Cassidy RN. Reduced nicotine product standards for combustible tobacco: building an empirical basis for effective regulation. Prev Med. 2014;68:17-22.
37. Weinberger AH, Mazure CM, Morlett A, McKee SA. Two decades of smoking cessation

treatment research on smokers with depression: 1990-2010. Nicotine Tob Res. 2013;15(6):1014-1031.
38. Gaalema DE, Miller ME, Tidey JW. Predicted impact of nicotine reduction on smokers with affective disorders. Tob Regul Sci. 2015;1(2):154-165.
39. Tidey JW, Pacek L, Koopmeiners JS, Vandrey R, Nardone N, Drobes DJ, Benowitz NL, Dermody SS, Lemieux A, Denlinger RL, Cassidy R, al'Absi M, Hatsukami DK, Donny EC. Effects of 6-week use of reduced-nicotine content cigarettes in smokers with and without elevated depressive symptoms. 2017;19(1):59-67.
40. Dawkins L, Kimber C, Puwanesarasa Y, Soar K. First- versus second-generation electronic cigarettes: predictors of choice and effects on urge to smoke and withdrawal symptoms. Addiction. 2015;110(4):669-77.
41. Farsalinos KE, Spyrou A, Tsimopoulou K, Stefopoulos C, Romagna G, Voudris V. Nicotine absorption from electronic cigarette use: comparison between first and new- generation devices. Sci Rep. 2014;4:4133.
42. Goniewicz ML, Kuma T, Gawron M, Knysak J, Kosmider L. Nicotine levels in electronic cigarettes. Nicotine Tob Res. 2013;15(1):158-166.
43. Talih S, Balhas Z, Eissenberg T, Salman R, Karaoghlanian N, El Hellani A, Baalbaki R, Saliba N, Shihadeh A. Effects of user puff topography, device voltage, and liquid nicotine concentration on electronic cigarette nicotine yield: measurements and model predictions. Nicotine Tob Res. 2015;1(2):150-7.
44. Trtchouniam A, William, M, Talbot P. Conventional and electronic cigarettes (e- Cigarettes) have different smoking characteristics. Nicotine Tob Res. 2010;12(9):905- 912.
45. Farsalinos KE, Polosa R. Safety evaluation and risk assessment of electronic cigarettes as tobacco cigarette substitutes: a systematic review. Ther Adv Drug Saf. 2014;5(2):67- 86.
46. Rom O, Pecorelli A, Valacchi G, Reznick AZ. Are E-cigarettes a safe and good alternative to cigarette smoking? Ann N Y Acad Sci. 2015;1340:65-74.
47. Hecht SS, Carmella SG, Kotandeniya D, Pillsbury ME, Chen M, Ransom BW, Vogel RI, Thompson E, Murphy SE, Hatsukami DK. Evaluation of toxicant and carcinogen metabolites in the urine of e-cigarette users versus cigarette smokers. Nicotine Tob Res. 2015;17(6):704-709.
48. Goniewicz ML, Gawron M, Smith DM, Peng M, Jacob P 3rd, Benowitz NL. Exposure to nicotine and selected toxicants in cigarette smokers who switched to electronic cigarettes: A longitudinal within- subjects observational study. Nicotine Tob Res. 2017;9(2):160-167.
49. Cummins SE, Zhu S-H, Tedeschi GJ, Camst AC, Myers MG. Use of e-cigarettes by individuals with mental health conditions. Tob Control. 2014;23 Suppl 3:iii48-53.
50. Spears CA, Jones DM, Weaver SR, Pechacek TF, Eriksen MP. Use of electronic nicotine delivery systems among adults with mental health conditions. Int J Environ Res Public Health. 2017;14(1), pii: E10.
51. Pulvers K, Hayes RB, Scheuermann TS, Romero DR, Emami AS, Resnicow K, Olendzki E, Person SD, Ahluwalia JS. Tobacco use, quitting behavior, and health characteristics among current electronic cigarette users in a national tri-ethnic adult stable smoker sample. Nicotine Tob Res. 2015;17(9):1085- 1095
52. Biener L, Hargraves JL. A longitudinal study of electronic cigarette use among a population-based sample of adult smokers: association with smoking cessation and motivation to quit. Nicotine Tob Res. 2015;17(2):127-33.
53. Dunbar MS, Shadel WG, Tucker JS, Edelen MO. Use of and reasons for using multiple other tobacco products in daily and nondaily smokers: Associations with cigarette consumption and nicotine dependence. Drug Alc Depend. 2016;168:156-163.
54. Etter JF, Bullen C. Electronic cigarettes: users profile, utilization, satisfaction and perceived efficacy. Addiction. 2011;106(11):2017-2028.
55. Tackett AP, Lechner WV, Meier E, Grant DM, Driskill LM, Tahirkheli NN, Wagener TL. Biochemically verified smoking cessation and vaping beliefs among vape store customers. Addiction. 2015;110(5):868-874.
56. Hefner K, Rosenheck R, Merrel J, Coffman M, Valentine G, Sofuoglu M. E-cigarette use in Veterans seeking mental health and/or substance use services. J Dual Diagn. 2016;12(2):109-

17.
57. Hefner K, Valentine G, Sofuoglu M. Electronic cigarettes and mental illness: Reviewing the evidence for help and harm among those with psychiatric and substance use disorders. Am J Addict. 2017 Feb 2. doi: 10.1111/ajad.12504. [Epub ahead of print]
58. Miller ME, Tidey JW, Rohsenow DJ, Higgins ST. Electronic cigarette expectancies in smokers with psychological distress. Tob Regul Sci. 2017;3(1):108-114.
59. Pratt SI, Sargent J, Daniels L, Santos MM, Brunette M. Appeal of electronic cigarettes in smokers with serious mental illness. Addict Behav. 2016;59:30-34.
60. Bullen C, Howe C, Laugesen M, McRobbie H, Parag V, Williman J, Walker N. Electronic cigarettes for smoking cessation: a randomised controlled trial. Lancet. 2013;382(9905):1629-37.
61. Caponnetto P, Auditore R, Russo C, Cappello GC, Polosa R. Impact of an electronic cigarette on smoking reduction and cessation in schizophrenic smokers: a prospective 12-month pilot study. Int J Environ Res Public Health. 2013;10(2):446-461.
62. Tseng TY, Ostroff JS, Campo A, Gerard M, Kirchner T, Rotrosen J, Shelley D. A randomized trial comparing the effect of nicotine versus placebo electronic cigarettes on smoking reduction among young adult smokers. Nicotine Tob Res. 2016;18(10):1937- 1943.
63. Walton KM, Abrams DB, Bailey WC, Clark D, Connolly GN, Djordjevic MV, Eissenberg TE, Fiore MC, Goniewicz ML, Haverkos L, Hecht SS, Henningfield JE, Hughes JR, Oncken CA, Postow L, Rose JE, Wanke KL, Yang L, Hatsukami DK. NIH Electronic Cigarette Workshop: Developing a Research Agenda. Nicotine Tob Res. 2015;17(2):259-69.
64. Adriaens K, Van Gucht D, Declerck P, Baeyens F. Effectiveness of the electronic cigarette: An eight- week Flemish study with six-month follow-up on smoking reduction, craving and experienced benefits and complaints. Int J Environ Res Public Health. 2014;11(11):11220-11248.
65. Prochaska JJ, Grana RA. E-cigarette use among smokers with serious mental illness. PLoS One. 2014;9(11):e113013.
66. O'Brien B, Knight-West O, Walker N, Parag V, Bullen C. E-cigarettes versus NRT for smoking reduction or cessation in people with mental illness: secondary analysis of data from the ASCEND trial. Tob Induc Dis. 2015;13(1):5.
67. Rohsenow DJ, Tidey JW, Martin RA, Colby S, Eissenberg T. Electronic cigarettes effects on smoking, CO and motivation: A pilot study. Presented at the combined meeting of the Society for Research on Nicotine and Tobacco and the Society for Research on Nicotine and Tobacco-Europe, Florence, Italy. 2017.
68. Glasser AM, Collins L, Pearson JL, Abudayyeh H, Niaura RS, Abrams DB, Villanti AC. Overview of Electronic Nicotine Delivery Systems: A Systematic Review. Am J Prev Med. 2017;52(2):e33-e66.
69. Dawkins L, Turner J, Hasna S, Soar K. The electronic-cigarette: Effects on desire to smoke, withdrawal symptoms and cognition. Addict Behav. 2012;37(8):970-973.
70. Vansickel AR, Cobb CO, Weaver MF, Eissenberg TE. A clinical laboratory model for evaluating the acute effects of electronic "cigarettes": nicotine delivery profile and cardiovascular and subjective effects. Cancer Epidemiol Biomarkers Prev. 2010;19(8):1945-53.
71. Lechner WV, Meier E, Wiener JL, Grant DM, Gilmore J, Judah MR, Mills AC, Wagener TL. The comparative efficacy of first- versus second-generation electronic cigarettes in reducing symptoms of nicotine withdrawal. Addiction. 2015;10(5):862-867.
72. Vansickel AR, Eissenberg T. Electronic cigarettes: effective nicotine delivery after acute administration. Nicotine Tob Res. 2013;15(1):267-270.
73. Vansickel AR, Weaver MF, Eissenberg T. Clinical laboratory assessment of the abuse liability of an electronic cigarette. Addiction. 2012;107(8):1493-500.
74. Bonhomme MG, Holder-Hayes E, Ambrose BK, Tworek C, Feirman SP, King BA, Apelberg BJ. Flavoured non-cigarette tobacco product use among US adults: 2013- 2014. Tob Control. 2016;25(Suppl 2):ii4-ii13.
75. Villanti AC, Johnson AL, Ambrose BK, Cummings KM, Stanton CA, Rose SW, Feirman SP, Tworek C, Glasser AM, Pearson JL, Cohn AM, Conway KP, Niaura RS, Bansal- Travers M,

Hyland A. Flavored tobacco product use in youth and adults: Findings from the first wave of the PATH Study (2013-2014). Am J Prev Med. 2017 Mar 13. doi: 10.1016/j.amepre.2017.01.026. [Epub ahead of print]

76. Audrain-McGovern J, Strasser AA, Wileyto EP. The impact of flavoring on the rewarding and reinforcing value of e-cigarettes with nicotine among young adult smokers. Drug Alcohol Depend 2016;166:263-7.

77. Goldenson NI, Kirkpatrick MG, Barrington-Trimis JL, Pang RD, McBeth JF, Pentz MA, Samet JM, Leventhal AM. Effects of sweet flavorings and nicotine on the appeal and sensory properties of e- cigarettes among young adult vapers: Application of a novel methodology. Drug Alcohol Depend. 2016;168:176-180.

78. Berg CJ. Preferred flavors and reasons for e-cigarette use and discontinued use among never, current, and former smokers. Int J Public Health. 2016 Mar;61(2):225-36.

79. Kistler CE, Crutchfield TM, Sutfin EL, Ranney LM, Berman ML, Zarkin GA, Goldstein AO. Consumers' Preferences for Electronic Nicotine Delivery System Product Features: A Structured Content Analysis. Int J Environ Res Public Health. 2017;14(6). doi: 10.3390/ijerph14060613

80. Czoli CD, Goniewicz M, Islam T, Kotnowski K, Hammond D. Consumer preferences for electronic cigarettes: results from a discrete choice experiment. Tob Control. 2016;25(e1):e30-6.

81. Pesko MF, Kenkel DS, Wang H, Hughes JM. The effect of potential electronic nicotine delivery system regulations on nicotine product selection. Addiction. 2016;111(4):734- 44.

82. Etter JF. Electronic cigarettes: a survey of users. BMC Public Health. 2010;10:231. doi: 10.1186/1471- 2458-10-231.

83. Farsalinos KE, Romagna G, Tsiapras D, Kyrzopoulos S, Spyrou A, Voudris V. Impact of flavour variability on electronic cigarette use experience: an internet survey. Int J Environ Res Public Health 2013;10(12):7272-82.

84. Yingst JM, Veldheer S, Hrabovsky S, Nichols TT, Wilson SJ, Foulds J. Factors associated with electronic cigarette users' device preferences and transition from first generation to advanced generation devices. Nicotine Tob Res. 2015;17(10):1242-6.

85. Litt MD, Duffy V, Oncken C. Cigarette smoking and electronic cigarette vaping patterns as a function of e-cigarette flavourings. Tob Control. 2016; 25(Suppl 2):ii67-ii72.

86. Hickman NJ 3rd, Delucchi KL, Prochaska JJ. Menthol use among smokers with psychological distress: findings from the 2008 and 2009 National Survey on Drug Use and Health. Tob Control. 2014;23(1):7- 13.

87. Cohn AM, Johnson AL, Hair E, Rath JM, Villanti AC. Menthol tobacco use is correlated with mental health symptoms in a national sample of young adults: implications for future health risks and policy recommendations. Tob Induc Dis. 2016;14:1. doi:10.1186/s12971-015-0066-3.

88. Kroenke, K., Spitzer, R. L., Williams, J. B. W., Löwe, B. (2009). An ultra-brief screening scale for anxiety and depression: the PHQ-4 Psychosomatics, 50, 613-621.

89. Kroenke K, Spitzer RL, Williams JB. The PHQ-9: validity of a brief depression severity measure. J Gen Intern Med. 2001;16(9):606-13.

90. Spitzer RL, Kroenke K, Williams JB, Löwe B. A brief measure for assessing generalized anxiety disorder: the GAD-7. Ach Intern Med. 2006 May 22;166(10):1092-7.

91. Beck AT, Steer RA, Brown GK. Manual for Beck Depression Inventory – II 1996; San Antonio, TX: Psychological Corporation.

92. Norman SB, Cissell SH, Means-Christensen AJ, Stein MB. Development and validation of an Overall Anxiety Severity And Impairment Scale (OASIS). Depress Anxiety. 2006;23(4):245-249.

93. Sheehan DV, Lecrubier Y, Sheehan KH, et al. The Mini-International Neuropsychiatric Interview (M.I.N.I.): the development and validation of a structured diagnostic psychiatric interview for DSM-IV and ICD-10. J Clin Psychiatry. 1998;59 Suppl 20:22-33.

94. Sheehan DV, Lecrubier Y, Sheehan K, Harnett, Janavs J, Weiller E, et al. (1997). The validity of the Mini International Neuropsychiatric Interview (MINI) according to the SCID- P and its reliability. *Eur Psychiat 12*, 232–241.

95. Babor TF, de la Fuente JR, Saunders J, Grant M. AUDIT: The Alcohol Use Disorders Identification Test. Guidelines for use in primary health care. Geneva : World Health Organization, 1992.
96. Skinner HA. The Drug Abuse Screening Test. Addict Behav. 1982; 7(4):363-371.
97. Piper ME, McCarthy DE, Bolt DM, Smith SS, Lerman C, Benowitz N, et al. Assessing dimensions of nicotine dependence: An evaluation of the Nicotine Dependence Syndrome Scale (NDSS) and the Winsdomsin Inventory of Smoking Dependence Motives (WISDM) Nicotine & Tobacco Research. 2008;10:1009-1020.
98. Heatherton TF, Kozlowski LT, Frecker RC, Fagerström KO. The Fagerström Test for Nicotine Dependence: a revision of the Fagerström Tolerance Questionnaire. Br J Addict. 1991;86(9):1119-1127.
100. Foulds J, Veldheer S, Yingst J, Wilson SJ, Nichols TT, Eissenberg T. Development of a questionnaire for assessing dependence on electronic cigarettes among a large sample of ex-smoking E-cigarette users. Nicotine Tob Res. 2015; 17(2): 186-92.
101. DiClemente CC, Prochaska JO, Fairhurst SK, Velicer WF, Velasquez MM, Rossi JS. The process of smoking cessation: an analysis of precontemplation, contemplation, and preparation stages of change. J Consult Clin Psychol. 1991;59(2):295-304.
102. Cohen S, Kamarck T, Mermelstein R. A global measure of perceived stress. J Health Soc Behav. 1983;24(4):385-396.
103. Hatsukami DK, Kotlyar M, Hertsgaard LA, et al. Reduced nicotine content cigarettes: effects on toxicant exposure, dependence and cessation. Addiction. 2010;105(2):343- 355.
104. Watson D, Clark LA, Tellegen A. Development and validation of brief measures of positive and negative affect: the PANAS scales. J Pers Soc Psychol. 1988;54(6):1063-
105. Hughes JR, Hatsukami D. Signs and symptoms of tobacco withdrawal. Arch Gen Psychiatry. 1986;43(3):289-294.
106. Cox LS, Tiffany ST, Christen AG. Evaluation of the brief questionnaire of smoking urges (QSU-brief) in laboratory and clinical settings. Nicotine Tob Res. 2001;3(1):7-16.
107. Dowd AN, Motschman CA, Tiffany ST. Development and Validation of the Questionnaire of Vaping Craving. Nicotine Tob Res. 2019;12(1):63-70.
108. Cappelleri JC, Bushmakin AG, Baker CL, Merikle E, Olufade AO, Gilbert DG. Confirmatory factor analyses and reliability of the modified cigarette evaluation questionnaire. Addict Behav. 2007;32(5):912-923.
109. Jacobs EA, Bickel WK. Modeling drug consumption in the clinic using simulation procedures: demand for heroin and cigarettes in opioid-dependent outpatients. Exp Clin Psychopharmacol. 1999;7(4):412-426.
110. Cassidy RN, Tidey JW, Colby SM, Long V, Higgins ST. Initial development of an e- cigarette purchase task: a mixed methods study. Tob Regul Sci. 2017; 3(2): 139-150.
111. Grace RC, Kivel BM, Laugesen M. Estimating cross-price elasticity of e-cigarettes using a simulated demand procedure. Nicotine Tob Res. 2015 May;17(5):592-598.
112. Snaith RP, Hamilton M, Morley S, Humayan A, Hargreaves D, Trigwell P. A scale for the assessment of hedonic tone the Snaith–Hamilton Pleasure Scale. British Journal of Psychiatry. 1995;167:99–103.

STUDY PROTOCOL: SMOKERS WITH OPIOID USE DISORDER

Table of Contents

1. OBJECTIVE ... 50
2. SIGNIFICANCE ... 51
   2.1 Opioid Use Disorders (OUD) and Smoking ... 51
   2.2 Reducing the Addiction Potential of Tobacco Products ... 51
   2.3 Relevance of the Project to the Integrative Theme and Goals of the TCORS ... 52
   2.4 Relevance to the Scientific Domains and Priorities of the FDA CTP ... 52
   2.5 How study outcomes will improve scientific knowledge related to the manufacture, distribution, and marketing of tobacco products ... 52
3. RATIONALE ... 52
   3.1 Effects of Nicotine Reduction in Smokers With OUD ... 52
   3.2 Increasing Appeal of Alternative Non-Combusted Nicotine Sources ... 54
   3.3 E-Cig Use ... 54
   3.4 E-Cig Effects on Smoking ... 55
   3.5 Importance of E-Cig Flavors ... 55
   3.6 Products to be Tested ... 55
   3.7 Summary ... 56
4. PROJECT STUDY METHODS ... 56
5. STUDY SCREENING PROCEDURES ... 56
   5.1 Participants ... 56
   5.2 Recruitment ... 57
   5.3 Informed Consent Process ... 59
   5.4 Screening Measures ... 59
   5.5 Suicidality/Mental Health Monitoring ... 61
   5.6 Inclusion/Exclusion Criteria ... 61
   5.7 Eligibility Determination ... 63
6. STUDY BASELINE PROCEDURES ... 63
   6.1 Visit Scheduling Requirements for Baseline Period ... 65
   6.2 Measures/Assessments ... 65
   6.3 E-Cigarette Training Sessions ... 67
   6.4 Interactive Voice Response System ... 67
7. STUDY EXPERIMENTAL PROCEDURES ... 68
   7.1 Experimental Period ... 68
   7.2 Experimental Visits Weeks 1, 3, 5, 7, 9, 11, 13, and 15 Procedures ... 69
      7.2.A. Measures/Assessments ... 69
   7.3 Experimental Visits Weeks 2, 4, 6, 8, 10, 12, 14, and 16 Procedures ... 69

7.3.A. Measures/Assessments 69
7.4 Interactive Voice Response System 70
7.5 Variable Incentive Program 70
7.6 Product and Procedures Compliance Review Sessions 71
7.7 Quit Attempts During the Study Protocol 72
7.7.A. If a participant is currently abstaining from smoking with the intention to quit 72
7.7.B. If a participant is planning to quit smoking, but has not initiated the quit attempt 72
7.8 Abstinence Assessment Session 72
7.8.A. Participants Who Meet Criteria for Abstinence 73
7.8.A.1. Measures/Assessments 73
7.8.B. Participants Who Do Not meet Criteria for Abstinence 73
7.8.B.1. Measures/Assessments 73
7.9 Participant Compensation 74
7.10 End of Study 74
7.11 30-day Follow Up Phone Call 75
8. STUDY RANDOMIZATION 75
8.1 Randomization Process 75
8.2 Study Product Administration 76
8.3 Guidelines for Reporting Other Nicotine Product Use 76
8.4 Study Product Accountability 76
9. STUDY 3 STATISTICAL METHODS AND SAMPLE SIZE 76
9.1 Statistical Methods 77
9.2 Sample Size 77
10. POTENTIAL RISKS OF PARTICIPATION 78
10.1 Risks of Participation 78
10.2 Avoiding Risk to Fetus 80
10.3 Expected Benefits of Participation 80
11. PROTECTION AGAINST RISK 80
11.1 Data Collection Protections 80
11.2 Data Storage 81
12. ADVERSE EVENTS 81
13. WITHDRAWAL OR MONITORING OF PARTICIPANTS 81
14. DATA SAFETY MONITORING BOARD 83
15. INVESTIGATIONAL TOBACCO PRODUCT 83
16. CERTIFICATE OF CONFIDENTIALITY 83
17. OUTCOME VARIABLES 84
18. REFERENCES 85

Abbreviations

- 3HC: 3-hydroxycotinine
- BAL: Breath alcohol levels
- BDI: Beck's Depression Inventory
- BMI: Body Mass Index
- BP: Blood pressure
- BPM: Beats per minute
- BRIEF-A: Behavioral Rating Inventory of Executive Function
- CES: Cigarette Evaluation Scale
- CO: Carbon monoxide
- COT: Cotinine
- CPD: Cigarettes per day
- CPT: Cigarette Purchase Task
- CPT: Continuous Performance Task
- DAST: Drug Abuse Screening Test
- DDT: Delay Discounting Task
- D-KEFS: Delis-Kaplan Executive Function System
- EDC: Electronic Data Capture
- EQ-5D: Euro-Qol
- FSPTCA: Family Smoking Prevention and Tobacco Control Act
- FTND: Fagerström Test for Nicotine Dependence
- GAD: Generalized Anxiety Disorder
- HR: Heart rate
- IVR: Interactive Voice Response
- MDD: Major Depressive Disorder
- MINI: Mini International Neuropsychiatric Interview
- MNWS: Minnesota Nicotine Withdrawal Scale
- NMR: Nicotine metabolite ratio
- NNAL: 4-(methylnitrosamino)-1-(3-pyridyl)-1-butanol
- NNC: Normal nicotine content
- NNN: *N′*-nitrosonornicotine
- OASIS: Overall Anxiety Severity and Impairment Scale
- OUD: Opioid Use Disorder
- PANAS: Positive and Negative Affect Schedule
- PHQ: Patient Health Questionnaire
- PSS: Perceived Stress Scale
- QSU: Questionnaire of Smoking Urges
- RNC: Reduced nicotine content
- SST: Stop Signal Task
- TLFB: Timeline Follow Back
- TPQ: Time Perspectives Questionnaire
- VLNC: Very low nicotine content
- WASI-II: Wechsler Abbreviated Scale of Intelligence-II
- WISDM: Wisconsin Index of Smoking Dependence Motives

Protocol

1. OBJECTIVE

Prevalence of smoking among individuals with opioid use disorder (OUD) is six-fold that of the general US adult population. The mortality rate of opioid-dependent smokers is four times that of opioid-dependent nonsmokers, and their response to smoking cessation interventions is notoriously poor. A national policy of reducing the nicotine content of cigarettes has the potential to be an effective method of reducing tobacco use prevalence, dependence, and related adverse health outcomes. Controlled trials in the general smoker population have demonstrated that switching smokers to very low nicotine content cigarettes (VLNCCs) results in reductions in cigarettes per day (CPD), dependence and tobacco toxicant exposure, with few adverse consequences. Furthermore, our work during the current funding period indicates that smokers with OUD also respond to reductions in cigarette nicotine content with reductions in cigarette demand and other measures of addiction potential, although to a lesser extent than the other vulnerable smoker populations we have been examining (i.e., economically disadvantaged women, individuals with affective disorders). We believe that the impact of reduced nicotine standards on use of combusted cigarettes in this highly tobacco addicted population will be moderated considerably by other tobacco market conditions including (1) availability of alternative sources of non-combusted nicotine, and (2) whether these alternatives are available under conditions that optimize their appeal. We hypothesize the same for other vulnerable populations as well, but achieving significant reductions in use of combusted cigarettes in smokers with OUD seems especially unlikely in the absence of readily available and appealing alternative sources of non-combusted nicotine.

The goal of the proposed trial is to experimentally model whether increased availability and appeal of an alternative, non-combusted source of nicotine (e-cigarettes) will enhance the effectiveness of a reduced nicotine standard for cigarettes in smokers with OUD. Daily smokers receiving methadone or buprenorphine treatment will be recruited at University of Vermont and Johns Hopkins University and randomized to one of the following conditions: (1) normal nicotine content cigarettes (NNCC) alone, serving as the control condition, (2) VLNCCs alone, (3) VLNCCs + tobacco-flavored nicotinized e-cigarettes (TF e-cigs), or (4) VLNCCs + nicotinized e- cigarette with preferred flavoring (PF e-cigs). Participants will be asked to use only their assigned products for 16 weeks. Outcome measures include total CPD, product demand using behavioral-economic purchase tasks, craving, withdrawal, and biomarkers of tobacco toxicant exposure. In Week 17, participants will abstain from combusted cigarette use and we will assess effects on cigarette craving, withdrawal and demand.

This research will address the following specific aims:

Aim 1 (Primary): To compare the effects of: (1) NNCCs alone, (2) VLNCCs alone, (3) VLNCCs + TF e-cigs and (4) VLNCCs + PF e-cigs on total CPD in smokers with OUD. We hypothesize that at Week 16, total CPD will be reduced in a linear manner, with the largest reduction in the VLNCCs + PF e-cig condition.

Aim 2 (Secondary): To compare the effects of study conditions on measures of cigarette demand, smoke exposure (breath CO) and tobacco carcinogen biomarkers (NNAL, PAHs) in smokers with OUD. We hypothesize that at Week 16, these measures will have decreased in a linear manner, with the largest reduction in the VLNCCs + PF e-cig condition.

Aim 3 (Exploratory): To explore the effects of the four study conditions on cigarette demand,

craving, and withdrawal in smokers with OUD during the abstinence assessment period.

The integrative theme of this TCORS is vulnerable populations. The proposed research is relevant to FDA CTP's scientific domains of Addiction and Behavior by addressing whether reducing the nicotine content of cigarettes reduces cigarette use, dependence, and product appeal, and whether these effects are enhanced by availability of appealing alternative sources of non-combusted nicotine. It addresses the domain of Health Effects by assessing tobacco toxicant exposure and other biomarkers. The proposed study is significant and innovative by modeling the potential moderating effects of e-cigarette availability and appeal on a national reduced-nicotine policy for cigarettes in this understudied population. Finally, it is programmatic as it builds directly upon and extends the work our team accomplished during the Phase 1 funding period.

2. SIGNIFICANCE

2.1. Opioid Use Disorder (OUD) and smoking

Cigarette smoking is the leading preventable cause of US morbidity and mortality.[1] While smoking has steadily declined in the general population, it remains entrenched among vulnerable populations. Individuals with substance use disorders, mental illness or socioeconomic disadvantage are disproportionately represented among current cigarette smokers and bear a disproportionate burden of smoking attributable disease and premature death.[2-5] The focus of this proposal is on the vulnerable population of smokers with concurrent substance abuse. Recent data from Wave 1 of the Population Assessment of Tobacco and Health study show that among current adult cigarette smokers, 80.7% report past-year alcohol or drug use.[6] Current smokers are more than twice as likely as non-smokers to report past-year misuse of painkillers/sedatives and more than four times as likely to be past-year users of other drugs, including heroin. Conversely, individuals with substance use disorders (SUDs) are substantially more likely to die of tobacco-related disorders (e.g., lung and larynx cancers, respiratory disease) than the general population.[7-9] They are also more likely to die of problems related to their tobacco use than other drug use.[10]

As an exemplar of the larger population of smokers with SUDs, we have been focusing on those with concomitant opioid use disorder (OUD). The disproportionate burden of smoking and smoking-related consequences is especially evident in these individuals. **Prevalence of smoking among adults with OUD is six-fold that of the general US adult population (84-94% vs. 15%, respectively).**[11-17] Smokers with OUD also have greater severity of nicotine dependence. In recent analyses by our group of data from the National Survey on Drug Use and Health (2006- 2014), after accounting for sociodemographic and smoking characteristics, mean Nicotine Dependence Syndrome Scale scores were significantly higher for opioid-dependent smokers vs. smokers not dependent on opioids ($p<0.05$).[18] Finally, smoking is associated with significant morbidity and mortality in this population, with the **mortality rate of opioid-dependent smokers four times that of opioid-dependent nonsmokers**.[7,9-10]

The majority of OUD patients report knowledge of the adverse health consequences of smoking as well as a desire to quit smoking.[12,16-17,19-23] Despite this, their responses to standard smoking cessation treatments, including standard pharmacotherapies, are notoriously poor, with quit rates one-fourth that of non-substance abusers.[16, 24-29] Taken together, the high smoking prevalence, poor treatment response, and public health costs associated with smoking among opioid-dependent individuals are of significant concern given the scope of the current opioid epidemic, with 5% of Americans - over 16 million people - reporting recent opioid abuse.[30-31]

2.2. Reducing the addiction potential of tobacco products

One potential way to reduce smoking prevalence is to implement public policy mandating a reduction in cigarette nicotine content below the threshold necessary to establish and sustain nicotine dependence.[32] The 2009 Family Smoking Prevention and Tobacco Control Act gave the FDA the authority to regulate tobacco products to protect public health, including limiting the nicotine content of cigarettes. Nicotine is the constituent in tobacco that drives repeated use, dependence, and eventually the chronic diseases and premature death that result from chronic smoke exposure. Thus, reductions in nicotine content may reduce smoking prevalence and related

disease by disrupting initiation of smoking by new users and increasing cessation rates among current smokers.[33-34] This approach could be particularly beneficial to subpopulations of smokers who have less success with currently available cessation treatments, such as people with OUD.[35] Our work during the current funding period, reviewed below, indicates that smokers with OUD respond to reductions in the nicotine content of cigarettes with reductions in cigarette demand and other measures of addiction potential, although to a lesser extent than the other vulnerable smoker populations we have been examining (i.e., economically disadvantaged women, individuals with affective disorders (AD)). We anticipate that a *combination* of tobacco market conditions will be necessary to produce a maximal effect on combusted smoking in this population. In particular, we hypothesize that (1) making alternative sources of non-combusted nicotine (i.e., electronic cigarettes; e-cigs) available and (2) doing so under conditions that optimize their appeal (i.e., preferred flavoring) may enhance the ability of a national policy to reduce nicotine standards to decrease cigarette smoking, dependence, and related adverse health outcomes among smokers with OUD.

2.3. Relevance of the project to the integrative theme and goals of the TCORS

The integrative theme of our UVM TCORS is **vulnerable populations**, and our goals are to model the potential effects of tobacco product standards on product use in vulnerable populations, with the goal of reducing the risks of product use, dependence, and related adverse health outcomes. For the FDA to effectively execute its tobacco regulatory responsibilities, it must have sound scientific evidence on how product standards impact tobacco use in populations with high rates of tobacco dependence. Our goal is to provide the FDA with that evidence. This project is relevant to that goal because it will examine the effects of a reduced-nicotine standard for cigarettes, alone and combined with another FDA-regulated product (e-cigs) on measures of cigarette use, demand, dependence and tobacco toxicant exposure in this vulnerable population.

2.4. Relevance to the scientific domains and priorities of the FDA CTP

The proposed research is highly relevant to the CTP's scientific domains of **Addiction** and **Behavior** because it will address whether reducing the nicotine content of cigarettes reduces cigarette use, dependence, and product appeal, and whether these effects are enhanced by the availability of appealing alternative sources of non-combusted nicotine. It will address the **Health Effects** domain by assessing the effects of these conditions on tobacco toxicant exposure and a respiratory biomarker.

2.5. How study outcomes will improve scientific knowledge related to the manufacture, distribution and marketing of tobacco products

Outcomes will directly inform scientific knowledge concerning the manufacture of tobacco products by demonstrating whether a reduction in the maximum nicotine content of cigarettes to ≤0.4 mg nicotine/g tobacco would reduce smoking in this vulnerable population. The outcomes will also indicate whether continuing to allow the sale of e-cigs in characterizing flavors improves the efficacy of a reduced-nicotine standard for cigarettes on smoking reduction in this population.

3. RATIONALE

3.1 Effects of nicotine reduction in smokers with OUD

There is growing evidence to support the public health benefit of a nicotine reduction policy. Studies in the general smoker population have demonstrated that use of reduced nicotine content cigarettes decreases smoking rates, dependence and toxin exposure levels.[33,36-46] However, these studies were conducted with psychiatrically and socioeconomically stable, healthy smokers. While a national nicotine reduction policy may hold significant potential to reduce smoking prevalence and related adverse health outcomes, it is important to first understand whether vulnerable populations of smokers may respond differently than the general population.[43,47] This includes the possibility that these subgroups respond to reduced nicotine content cigarettes (RNCCs) with compensatory smoking, which could lead to increased carcinogen and other toxin exposure, failure to use RNCCs altogether, or transition to use of other products (e.g., cigars) which would undermine the merit of

this regulatory strategy.[48,49]

Research conducted by our UVM TCORS provides encouraging evidence that the beneficial effects of very low nicotine content cigarettes (VLNCCs) may extend to populations that are highly vulnerable to tobacco addiction and its associated adverse health consequences. During the current funding period, we have been conducting two multi-site studies examining acute and extended exposure to RNCCs on the addiction potential of smoking. The initial acute exposure study consisted of three parallel, within-subject laboratory studies in economically disadvantaged women of reproductive age (n=56), individuals with OUD (n=60), and individuals with AD (n=53).[50] Participants sampled NIDA-provided research cigarettes varying in nicotine content (15.8, 2.4, 1.3, 0.4mg/g) under double-blind conditions, with the highest dose representing nicotine content typical of commercial cigarettes. Following brief abstinence, participants completed 14 sessions comparing the relative reinforcing effects of the 6 possible dose pairs in concurrent choice procedures. We also assessed demand for each cigarette dose using the Cigarette Purchase Task (CPT), a behavioral economic task that models hypothetical demand for cigarettes under varying prices. Lastly, we compared cigarettes on smoking topography and subjective effects.

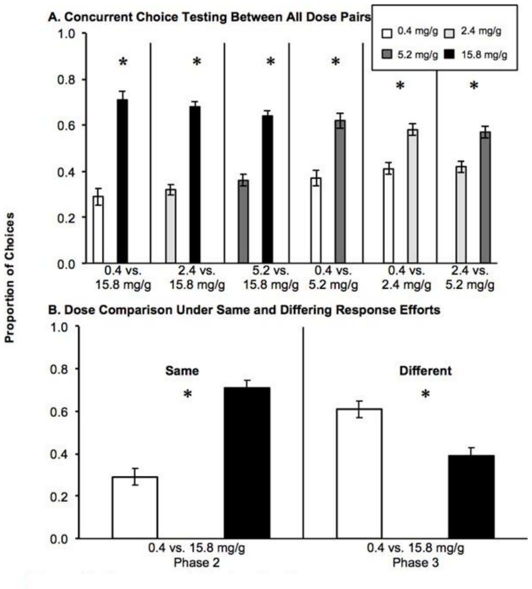

Figure 1. Concurrent Choice Testing

Results were collapsed across populations as similarities outnumbered differences. In concurrent choice testing, participants chose the higher over the lower nicotine content cigarettes across each of the six dose pairs when available at the same low price, providing strong evidence that reducing nicotine content decreases the relative reinforcing effects (i.e., addiction potential) of smoking in a graded dose-dependent manner (Figure 1, upper panel). Importantly in terms of regulatory implications, we also demonstrated that the robust preference for the high 15.8 mg/g control dose over the 0.4 mg/g dose was reversed in all three populations by incrementing the response effort required to obtain the high dose while keeping the low dose available at the same low response requirement (lower panel). This suggests that product preference is conditional on environmental context. Demand for cigarettes on the CPT task also decreased as an orderly function of nicotine content consistent with the differences in relative reinforcing effects seen in the concurrent choice task. Importantly, there was no evidence of compensatory smoking. This study provided a compelling demonstration that RNCCs reduce the addiction potential of cigarettes in populations highly vulnerable to tobacco addiction. It is worth noting, however, that we observed greater overall cigarette demand across doses in smokers with OUD vs. the other vulnerable populations examined in this study.

Our second multi-site study is ongoing and on schedule to be completed as planned. The study uses a three parallel-groups design to investigate the effects of 12-week exposure to RNCCs (15.8, 2.4, and 0.4 mg/g). Preliminary results on total cigarettes per day (CPD) are shown in Figure 2 for the individuals that have completed the study thus far. Presented are results for the 15.8 mg/g control (i.e., usual brand cigarette) dose and the VLNCC 0.4 mg/g dose,

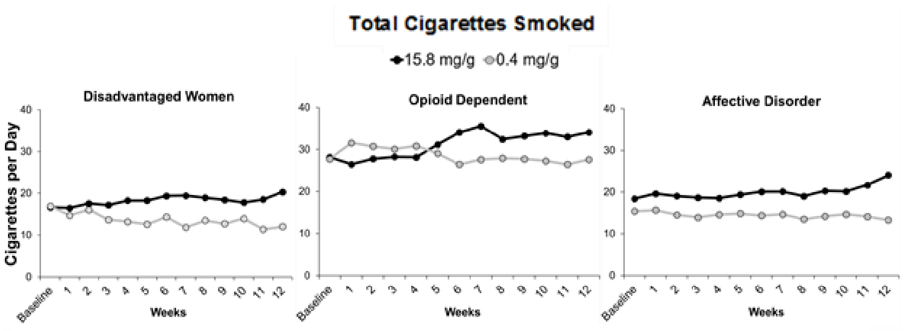

Figure 2. Total cigarettes smoked across 12-week period in three vulnerable populations.

which has the lowest addiction potential. These also represent the two doses that we are proposing to study in this renewal. Thus far, there is no evidence of sustained compensatory smoking in any population receiving VLNCCs. CPD across the two doses separates over time in all three vulnerable smoker groups; however, it does so later and overall consumption is greater among smokers with OUD. Additionally, the current effect size for differences in CPD at 12 weeks among the dis-advantaged women and smokers with AD (Cohen's d=0.70 and 0.81, respectively) are larger than those seen in the opioid dependent population (d=0.38) and consistent with those seen at 6 weeks in the general population of smokers (0.79[51]). Fagerstrom dependence scores at 12 weeks are also significantly decreased among those assigned to VLNCCs vs. the 15.8 mg/g dose in the disadvantaged women and those with AD but not in individuals with OUD. The overall greater cigarette demand and consumption exhibited by smokers with OUD serves as an important reminder of the especially persistent nature of smoking in this population.

3.2 Increasing appeal of alternative non-combusted nicotine sources

These data provide promising initial evidence that smokers with concomitant other socioeconomic, substance use and psychiatric vulnerabilities will respond to RNCCs with subsequent reductions in cigarette demand and other measures of addiction potential. However, there is growing recognition of the critical importance of having appealing alternative non-combusted nicotine sources readily available if the potential of a reduced nicotine standards policy for combusted products is going to be realized. In the words of Benowitz and colleagues, "*The RNCC and the emergence of non-combusted nicotine products like e-cigs should be viewed not as alternatives but as complementary components of regulatory interventions that could virtually end combusted tobacco use.*"[52] Further, the primary conclusion of the 2014 Surgeon General's Report noted that "*the promotion of noncombustible tobacco products is much more likely to provide public health benefits only in an environment where the appeal, accessibility, promotion, and use of cigarettes and other combusted products are being rapidly reduced.*"[1] Our data also lead us to anticipate that smokers with OUD and perhaps other SUDs may persist in smoking even if cigarette nicotine content is reduced to very low levels. This further highlights the critical importance of having non-combusted alternative sources of nicotine readily available at reinforcing doses and in forms that enhance appeal. We know of only one report empirically examining how the presence of non-combusted products influences use of VLNCCs and that was an exploratory study of participants selected from the general smoker population.[44] In that study, when individuals assigned to VLNCCs opted to also use a non-combusted product, they overwhelmingly chose e-cigs over other non-combusted options (i.e., snus, NRT). This is consistent with other research modeling the substitutability of non-combusted tobacco products for combusted cigarettes[53] and serves as the rationale for us focusing on e-cigs in this renewal application.

3.3 E-cig use

E-cigs consist of a cartridge containing an e-liquid nicotine solution, propylene glycol (PG), vegetable glycerin (VG), flavoring, and other additives, which are heated with an atomizer that vaporizes the solution. First generation e-cigs resemble cigarettes and are disposable or rechargeable. Second generation products (and the product used in this study) often resemble pens and have refillable e-liquid reservoirs, while third generation devices have larger batteries, adjustable power delivery and replacement heating coils and wicks.[54] Nicotine delivery from e- cigs can be comparable to those from cigarettes, depending on e-cig characteristics, nicotine content and user topography.[55-58] Toxin and carcinogen levels appear to be lower than those from cigarettes. Hecht et al.[59] reported that former smokers who had switched to e-cigs had 59-99% lower levels of 6 tobacco toxicant and carcinogen metabolites than ongoing smokers, comparable to reductions seen in smokers who had switched to nicotine lozenges.[42] Another study found that NNAL, a metabolite of the tobacco carcinogen NNK, was reduced by 64% in smokers who had switched to e-cigs for two weeks, as was chest tightness.[60] Initial data suggest that e-cigs may be a promising tool for supporting transition away from combusted cigarettes in smokers with OUD.[61-64] In a recent observational study with methadone- and buprenorphine-maintained smokers, 99% were familiar with e-cigs, 73% had ever tried them, and 34% were currently using e-cigs.[65] As in the general

population, smokers with OUD reported that their reasons for e-cig use are primarily to quit or reduce smoking.[65]

3.4 E-cig effects on smoking

E-cig use is associated with smoking reduction[66-68] and possibly quitting. In a trial using second generation e-cigs, cigarette abstinence rates were 34% after 8 weeks and 21% 6 months later, with an overall 60% reduction in CPD.[69] The only published study to our knowledge on the effects of e-cigs in smokers with OUD was an uncontrolled, 6-week pilot study with 12 methadone- maintained smokers. E-cig use was associated with significant reductions in self-reported combusted cigarette use, few adverse effects, and excellent e-cig adherence (89%[70]). While e- cigs can reduce cigarette craving and nicotine withdrawal,[55,69,71-72] these effects are generally determined by the extent to which e-cigs are used by smokers; in turn, determinants of e-cig use include product appeal and reinforcing effects.[73]

3.5 Importance of e-cig flavors

Over two-thirds of adult e-cig users use a flavored e-cig.[5,74] Flavors have been shown to substantially enhance e-cig appeal and relative reinforcing effects[75-76] and have been cited as a key feature affecting e-cig use among adults.[5,77-78] Experimental studies show that flavors increase demand for e-cigs among cigarette smokers.[79-80] Studies of e-cig users also highlight that flavors play an important role in their experience of the product as well as reducing cigarette consumption and craving.[81-84] Importantly, there is a higher prevalence of flavored tobacco use among smokers with SUDs relative to the general population.[5,12,14] A growing literature also suggests that opioid-dependent individuals may be especially sensitive to flavors, as activation of the mu-opioid receptor by opioid administration is associated with increased preference for sweet flavors.[85-90] These data suggest that combining a non-combusted nicotine source with a preferred, likely sweet, flavor may have substantial appeal and thus potential for reducing combusted cigarette use in OUD smokers.

3.6 Products to be tested

<u>Cigarettes to be assessed</u>

The cigarettes to be used in this study were made under an NIH contract with production being overseen by the Research Triangle Institute (referred to as “Spectrum cigarettes”). NIH currently has approximately 10 million of these cigarettes (of varying types) for research purposes. The cigarettes selected for the study span the range of yields likely to produce the hypothesized effects, as described above. Spectrum cigarettes are not currently commercially available, although they are similar in many ways to marketed cigarettes (e.g., similar manufacturing, filter, paper, etc.).

<u>E-cigarettes to be assessed</u>

Both the JUUL and the Vuse Solo will be used and assessed in this study. . While JUUL will be offered to all participants, participants that are unwilling to use JUUL will be offered the Vuse Solo. JUUL is a commercially available closed system containing two components. One component contains a lithium-ion battery (200 mAh), nichrome coil heater, silica wick, and stainless steel vapor path. The other component is the prefilled e-liquid container that also serves as the mouthpiece. Each commercially available cartridge holds approximately 0.7 mL of e-liquid containing approximately 40 mg of nicotine or 5% nicotine by weight (NBW). A lower dose containing approximately 23 mg of nicotine per cartridge or 3% NBW is also marketed but will not be used in this study. All containers contain glycerol, propylene glycol, natural oils, extracts and flavors, nicotine, benzoic acid. We will not alter the e-liquid in any way. The research staff will distribute the e-liquid containers as purchased from the manufacturer. The JUUL apparatus and 5% NBW e-liquids that will be used are legally purchasable and have been as of August 8, 2016. We will not alter them in any way.

Vuse Solo is a commercially available closed system containing two components. The power/heating device includes a 270 mAh battery, silica wick, microchips, and sensor. The other

component is the prefilled e-liquid container. Each commercially available cartridge holds approximately 1 mL of liquid containing 48 mg of nicotine or 4.8% NBW. All containers contain vegetable glycerin, propylene glycol, reverse-osmosis water, glycerin, flavorings, and nicotine. The research staff will distribute the e-liquid containers as purchased from the manufacturer. The Vuse apparatus and e-liquid cartridges that will be used are legally purchasable and have been as of August 8, 2016. We will not alter them in any way.

3.7. Summary

Although smoking rates have declined in the overall US population, there has been little to no decline among people with OUD. A nicotine reduction strategy for combustible tobacco combined with e-cig availability may have complementary effects on smoking reductions and consequent tobacco-related health effects in this vulnerable population. This research is highly significant because it will model how the availability of e-cigs impacts the effectiveness of a reduced-nicotine policy for cigarettes in vulnerable smokers. It is responsive to the goals of the FDA in that the study conditions are designed to model real-world scenarios of possible harm reduction policies in populations that are vulnerable to smoking persistence.

4. Project Study Methods

This study will use a four-condition, parallel-groups research design. After a baseline period in which daily smoking rate and other baseline assessments are completed, participants will be randomly assigned to one of the following four conditions for a 16-week experimental period: (1) normal nicotine content cigarettes (NNCCs, 15.8 mg/g) alone, which serves as the control condition; (2) very low nicotine content cigarettes (VLNCCs, 0.4 mg/g) alone; (3) VLNCCs + tobacco-flavored nicotinized e-cigs (TF e-cig, 4.8 - 5.0% nicotine by weight, NBW, if they choose to use the Vuse or JULL devise, respectively); or (4) VLNCCs + preferred-flavor nicotinized e-cigarette (PF e-cig).

5. Study Screening Procedures

5.1 Participants

Participants will be men and women, ages 21-70, who are currently receiving methadone or buprenorphine maintenance for OUD. They must report smoking ≥5 cigs/day for the past year and provide a breath CO ≥ 8ppm or a positive urine cotinine value. They must be sufficiently literate to complete research tasks, be in good physical health without serious illness or change in health in the past 3 months, must not be daily e-cigarette users, and have the technological capabilities to complete weekly face-to-face video assessments and the compatibility to use ico Smartphone Smokerlyzers for assessing breath carbon monoxide (CO) levels.. As opioid and cocaine use can directly increase smoking rates,[91-96] participants must be maintained on a stable methadone or buprenorphine dose with no regular illicit-drug abuse (<4 positive specimens) for the past month. Consent to confirm dose and drug abstinence with the participant's opioid clinic will be obtained at intake, and we will monitor any changes in dose throughout the study. These practices have been effective for confirming clinical stability in our prior smoking trials with opioid- maintained patients.[50,96-100]

We will exclude those <21 years old as users below that age have legal restrictions on ability to purchase these products that can alter use patterns for reasons that do not apply to adult populations. We will exclude pregnant and nursing women and women not reporting use of contraceptives, anyone currently using nicotine replacement, bupropion or other pharmacotherapies as cessation aids, those who report daily use of e-cigs in the past 30 days as they may not be compliant with the study e-cigs, and anyone intending to quit within 3 months, as participation in this study may not reduce smoking and could increase smoke and/or nicotine exposure. Those who exclusively use roll-your-own cigarettes or used other non-cigarette tobacco products (e.g., cigars, smokeless) ≥10 days in the last 30 are excluded as their use of these products may reduce the validity of our main outcome measure, total CPD (which includes study and non-study cigarettes). The following conditions are excluded as they could affect participants'

abilities to complete the study: unstable medical or medication conditions (significant changes in a serious medical condition in the past 3 months, medication changes in the past 4 weeks; details provided in Protection of Human Subjects section), symptoms of psychosis or dementia, past-month suicidality, current (past-6 months) alcohol or SUDs other than nicotine and the OUD for which they are being treated, positive toxicology screen for illicit drugs (marijuana allowed; one re-screen opportunity), positive breath alcohol level (BAL) at screen (one re-screen opportunity). Participants who meet criteria and provide written informed consent will be enrolled into the trial.

5.2 Recruitment

This study will be conducted at the University of Vermont (UVM) in Burlington, VT (primary site) and Johns Hopkins University (JHU) in Baltimore, MD. This collaboration will permit us to complete this study expeditiously, enhance sample diversity, and facilitate meaningful collaboration across multiple sites. Participants will be recruited through advertisements posted in local opioid treatment clinics, in local newspapers, on community bulletin boards, Craigslist, Facebook and word of mouth. Participants can choose to complete the pre-screening questionnaire online or by phone. At UVM, individuals recruited from online sources will be directed to a UVM-hosted recruitment website where they will have the opportunity to select which research studies interest them. They will then be redirected to a brief online screener to assess eligibility. Those who contact us by phone will be given a description of the study and asked questions to assess eligibility. The RA will read a script briefly explaining the study. Participants will be informed that this is not a smoking cessation program, and that smoking cessation services are available in the community independent of their decision to participate in this study. If interested and eligible, participants will be asked to present for a brief 10-minute in-person medical consent visit where research staff obtain informed consent to contact participant's opioid treatment provider to confirm stability in treatment. Participants will be asked to provide a urine sample for toxicology screening. If interested and eligible following the medical consent visit, they will be invited to participate in the first portion of the screening interview. Research assistants will inform eligible participants that the screening will occur over video chat, and will assist the participant with setting up an appropriately secure video platform.

During this first portion of the screening, the participant will complete questionnaires through REDCap online while the research assistant is present over video chat or phone to deliver instructions and to answer any questions. The participant will then answer interviewer-administered questionnaires over video chat. Participants who did not yet set up their video platforms will do so with the research assistant before beginning any questionnaires. Participants will be instructed to have picture identification (e.g. driver's license) available to show the staff. If participants anticipate not having acceptable ID, staff should consult with the project coordinator or study PI. Initial study eligibility will be determined after data are collected from this visit. Participants who meet initial study eligibility will be scheduled for the second portion of the screening.

Before the second portion of the screening occurs, eligible participants will receive the equipment necessary to use for collecting physiological measurements. Participants will be asked to pick up this equipment via curbside pickup at our clinic (UVM University Health Center, UHC), which will consist of participants calling staff once they arrive at UHC and staff coming out to give participants a bag/box containing the following equipment: a Smokerlyzer; an audio jack adapter for the Smokerlyzer if necessary; a blood pressure cuff; an oximeter; a thermometer; a urinary cotinine dipstick; urine cups with attached temperature test strips; a pregnancy test strip (if applicable) and urine toxicology test strips or a saliva toxicology test. Participants (and staff) will be asked to use cloth face coverings when exchanging product. Participants may be invited to come inside to pick up this equipment if the participant is asked to wait for this exchange. All participants must pass a COVID19 screening before entering the building. If there is any waiting that needs to occur inside the building, the participant will wait inside one of our five highly ventilated smoking chambers. If there happens to be no space in the smoking chambers, the participant will be told that they can not come up to the clinic until space is available. After each use, the all of the surfaces in the smoking chambers will be cleaned with 70% or greater of alcohol solution by staff

wearing a mask and gloves, as well as all of the door handles. If the participant uses the bathroom while they are in the clinic, the bathroom surfaces and handles will be wiped down after use by staff while masks and gloves are worn. Participants who are using the smoking chambers at any point in the study to wait for product or equipment exchange will remain in the chambers until a staff member comes to knock on the door to let them out. In this way, we can avoid people coming into close contact with each other in the larger room that contains the smoking chambers. A minimum of 6 feet of distance will be maintained for all staff and participants at all times. For participants who cannot come to the clinic, a commercial courier will deliver this equipment to them before the second portion of the screening.

If at any point the Smokerlyzers are not available for distribution, we will conduct the CO test curbside before or after participants are invited inside and the courier service will not be available. Research Assistants will bring down the CO monitor to the participant. While maintaining 10 feet of distance and wearing gloves, staff will explain how the CO monitor works. Once the participant is ready, staff will press the button to obtain the measurement and will set the CO monitor down and will back away 10 feet. The participant will then come to pick up the device and will blow into the monitor. After the participant completes the test, they will set down the monitor and back up 10 feet and staff will retrieve the monitor. After every use, staff will wipe down the CO monitor with disinfectant wipes and hydrogen peroxide wipes. When using the monitor, a D-piece (a portable valve filter) must be placed into the monitor and then the single use plastic mouthpiece is placed into the D-piece. The monitor has built in SteriTouch technology to ensure optimum infection control, and the D-pieces filter out 99.9% of airborne bacteria and greater than 97% of viruses for excellent infection control. Each participant will be assigned their own D-piece to use throughout the study, and no D-piece will ever be shared among participants. Participants will gently exhale into the D-piece for the breath carbon monoxide reading. Participants will be instructed only to exhale through the device, not to inhale. D-piece technology also includes a one-way valve that prevents air from being drawn back from the monitor. D-pieces will also be wiped down after each use with disinfectant wipes and hydrogen peroxide wipes and stored in a container at the lab.

Once participants have received the necessary equipment to complete the physiological portion of the screening, the research assistant will initiate a video call with the participant. During this call, the participant will be instructed on how to use the equipment and then will be asked to use the equipment to obtain the following physiological readings: blood pressure, heart rate, oxygen saturation, temperature, and breath CO levels. Participants will also be asked to collect a urine or saliva sample during the visit. If a saliva sample is collected, the participant you will provide the saliva sample over video chat while the staff observes. If a urine sample is collected, staff will ask participant to bring this urine sample to the video screen after collection to perform a urine toxicology test and a pregnancy test (if applicable). These urine cups will have temperature strips affixed to ensure that the sample is valid. Participants who have a carbon monoxide level of less than or equal to 8 will also be asked to use the urinary cotinine dipstick to determine whether they are positive for cotinine. The participant will obtain the physiological readings and perform the tests and then will hold the results of the test up to the camera so that the research assistant can interpret and record the readings on REDCap, UVM participants will be primarily recruited from our university opioid program, The Chittenden Center (CC). Dr. Sigmon is the Director of this clinic, which currently has 1,000 methadone- or buprenorphine-maintained patients. A survey of CC patients indicates that 88% currently smoke, which translates to approximately 880 smokers at our on-site clinic alone. Recruitment will also draw from patients receiving treatment at the office-based buprenorphine practices throughout the Burlington, VT community. JHU participants will be recruited primarily from two clinics located directly on the JHU Bayview Medical Center Campus: Addiction Treatment Services, a methadone clinic with 500 patients, and Comprehensive Care, a primary care clinic with 300 patients receiving buprenorphine. Should these resources be inadequate at any point in time, we will also recruit from two large community-based methadone programs in Baltimore with whom Dr. Stitzer has an on-going relationship as a PI of the NIDA Clinical Trials Network. Taken together, our access to opioid-maintained smokers is more than adequate to fulfill the recruitment needs of this project. Power estimations were based on the 15% dropout rate across populations in our current trials. Using an intent-to-treat approach, we require

75 randomized in each condition (300 total) to test our primary aims.

Participants will be also instructed to have handy a pack of their usual brand cigarettes, all prescription medications they are currently taking and identification (example, driver's license) during this second portion of the screening visit. If participants anticipate not having acceptable ID site staff should consult with the project coordinator or study PI.

A participant must complete his/her two-part screening session within 90 days of completing the pre-screening questionnaire. If the participant is not able to complete the two-part screening visit in that timeframe, he/she will need to complete the pre-screening questionnaire again.

5.3 Informed Consent Process:

Before beginning the informed consent process, potential participants will need to produce identification as described above. The interviewer will confirm the age and identity of the participant. If the participant is not between the ages of 21 and 70, he/she will be dismissed without payment. During the first portion of the screening session, study information will be presented and documentation of the participant's informed consent via electronic signature on REDCap will be required prior to participating in the screening session. In order to ensure adequate informed consent, participants will be asked to read the first several lines aloud (to determine literacy) and will then be given ample time to read the consent document. If the interviewer suspects the participant is not literate, he or she will have them continue reading further to confirm. Inability to read and comprehend written study materials will result in ineligibility and the interviewer will inform the participant that they are not eligible. Only after the participant and the researcher are fully satisfied that the participant understands the purpose of the study, the confidentiality of the data, the procedures, the risks/benefits and his/her rights as a research participant will the consent form be signed and the participant undergo screening procedures.

5.4 Screening Measures

Those who consent will be screened for eligibility using the following measures:

The following physiological measures will be collected and entered directly into REDCap by the interviewer:

1) Expired breath carbon monoxide (CO) levels will be assessed using a Smartphone Monitor (Covita - for remote collection) or a Bedfont CO monitor (for curbside collection), a reliable and valid measure of recent smoking.
    a. Urinary cotinine test strips will be used to asses urinary cotinine levels if a participant's CO reading is less than or equal to 8 ppm.
2) A urine or saliva toxicological screen will be performed to assess the presence of illicit drugs including up to the following drugs: marijuana, cocaine, opiates, oxycodone, benzodiazepines, barbiturates, amphetamines, methadone, buprenorphine, methamphetamines, MDMA and PCP. Participants who fail the drug screen for drugs other than marijuana or their prescribed opioid medication may reschedule the interview but will need to be re-consented to ensure they have received adequate informed consent. They will be excluded if they are positive for drugs (other than marijuana or prescribed medications as determined by PI on a case-by-case basis) the second time.
3) Urine Pregnancy Test (HCG detection) will be performed for all participants.
4) Blood pressure and heart rate will be measured using an automated blood pressure monitor and a finger pulse oximeter to help the licensed medical professional determine final participant eligibility. Participants will be told if their blood pressure is in an abnormal range and advised to see a doctor by research staff. The research staff will also submit a medical event form for the LMP to review along with a Blood Pressure and Heart Rate Symptom Checklist form to ascertain details of the symptomatology for the LMP to review. In severe cases, the LMP may also choose to call the participant to follow-up and/or withdraw the participant from the study if necessary. All of these procedures are documented in our Blood Pressure/Heart Rate Collection: Standard Operating Procedure form which we can submit to the IRB if the Committee deems necessary.

5) Body temperature, respiratory rate and oxygen saturation will be added as physiological measures based on the CDC recommendations and those of Dr. David Kaminsky. https://www.cdc.gov/tobacco/basic_information/e-cigarettes/severe-lung-disease/healthcare-providers/index.html

The following screening assessment will be administered as an interview:

1) The Mini International Neuropsychiatric Interview (MINI) 7.0[70]

The following screening assessments will be administered as an interview and entered directly into REDCap by the interviewer:

1) The MINI Plus Modules
2) The MINI suicide subscale[104] to evaluate suicide risk.
3) MINI Follow-up Questionnaire (if applicable)
4) Tobacco Use History and Exposure Questionnaire, which measures variables such as smoking amount, cigarette brand, age of initiation of smoking, number of quit attempts, duration of quit attempts and duration of smoking.
5) Smoking Cessation Therapy Use Questionnaire
6) Time Since Last Cigarette Questionnaire
7) Maintenance Drug Dose Questionnaire – Screening Version
8) Medical History Questionnaire to assess current diagnoses, symptoms and past health problems.
    a. Medications will be recorded directly onto the Concomitant Medications form in REDCap.
9) Drug Abuse Screening Test (DAST-10), which assesses quantity and frequency of alcohol and drug use (12 month and 1 month version)

The following screening assessments will be completed by the participant directly in REDCap, except where noted otherwise:

1) Demographic History Questionnaire, which will assess age, gender, ethnicity, race, education, income, marital status, and employment history.
2) Alcohol Use Questionnaire---based on the Alcohol Use Disorders Identification Test[105] (12 month and 1 month version)
3) Drug Use Questionnaire---based on the Drug Abuse Screening Test[106] (12 month and 1 month version)
4) Fagerström Test for Nicotine Dependence (FTND)[107];
5) Wisconsin Inventory of Smoking Dependence Motives-Brief Scale (WISDM)[108] will be administered to assess nicotine dependence severity.
6) Penn State Electronic Cigarette Dependence Index[109];
7) Smoking Stages of Change Algorithm[110];
8) Identifying Information Form will include the participant's REDCap Subject Identifier, name, address (including the county of residence), email address, phone number, age, date of birth, and social security number (if applicable).
    a. This form will be entered into the 'Identifying Information Access Database'.
        i. Each site will have a separate 'Identifying Information Access Database'.
        ii. Identifying information will not be shared with other sites. Each site is responsible for maintaining confidentiality of this information.
        iii. Identifying information will be kept in a locked file cabinet (source document) and in a password protected Access Database (electronic version) separate from all other study data.
9) Beck Depression Inventory (BDI-II)[68], to assess depressive symptoms. (This form has been updated on 1.29.20 so as to use the BDI-II. The BDI-II will be used instead of the BDI so that the data collected will be directly comparable to other projects using the BDI-II).
10) Overall Anxiety Severity and Impairment Scale[69](OASIS); to assess frequency and severity of anxiety symptoms.
11) COVID19 Symptom Questionnaire

12) Respiratory Symptom Questionnaire will be administered to assess respiratory health

In the event that the REDCap website is not functioning, the assessments will be administered aloud and participant answers will be recorded securely. The interviewer will enter the data into REDCap when it resumes functioning properly. This information should be recorded in the 'End of Visit Evaluation Form'.

5.5 Suicidality/Mental Health Monitoring

Participants who endorse suicidal intention in the past month or a suicide attempt in the past 6 months as indicated on the BDI (score > 1 on question 9) or MINI suicide subscale (endorse question 4 and/or 5 on the MINI suicide subscale or question 6 on the MINI suicide subscale with suicide attempt in the past 6 months) or answer "yes" to question A3g on the MINI Neuropsychiatric interview and symptoms have occurred in the past two weeks will be assessed by a clinician for eligibility and possible intervention. The research staff member will contact a licensed clinician for evaluation. In the event that no clinician is available, staff will put the participant in contact with the National Suicide Prevention Lifeline at 1-800-273-8255. They will also contact the Study Coordinator and Site PI to inform them of the situation as soon as possible. Additionally, they will contact the Project Coordinator to inform her of the situation. The participant will be paid $25 (+$25 bonus if applicable) and provided with local mental health resources. Post enrollment, any report of suicidal ideation or attempt by a participant will be grounds for immediate withdrawal from the study.

5.6 Inclusion/Exclusion Criteria

Inclusion Criteria:

6) Men and women ages 21-70, who are currently receiving methadone or buprenorphine maintenance treatment for opioid dependence;
7) Report smoking ≥ 5 cigarettes per day for the past year;
8) Provide an intake breath CO sample >8 ppm, (if ≤ 8 ppm, then urinary-cotinine strip must be positive);
9) Be without current (within the past year) serious mental disorder that would interfere with study results of completion as determined by the licensed medical professional or PI,
10) Be sufficiently literate to complete the research-related tasks;
11) Be in good physical health without serious illness or change in health or medication (not including methadone or buprenorphine dose) in the past three months as determined by the licensed medical professional at each site;
12) Participants must be maintained on a stable methadone or buprenorphine dose for the past month, with no evidence of regular illicit-drug abuse (<30% positive specimens in the past 30 days).
    a. Consent to confirm dose and drug abstinence with the participant's opioid clinic will be obtained prior to screening at a medical consent visit and we will monitor any changes in dose throughout the study.
    b. Participants must provide proof of at least three biochemical (e.g. urine, saliva) samples within the last 30 days of the date of the medical consent visit that have no evidence of illicit drug use. If they do not have three, they will be asked to provide additional samples as needed and coordinated by research staff to confirm clinical stability. They may provide up to two samples per week with at least one full day between samples.
13) Have appropriate equipment to complete face-to-face video assessments and use ico Smartphone Smokerlyzer Monitors. For those who do not have a Smartphone, staff will explore potential alternate plans (e.g., project-provided inexpensive Android phone)

Exclusion Criteria:

1) Exclusive use of roll-your-own cigarettes;

2) Planning to quit smoking in the next 30 days;
3) A quit attempt in the past 30 days resulting in greater than 3 days of abstinence;
4) Significant use of other tobacco or nicotine products within the past month (more than 9 days in the past 30).
5) Currently taking anticonvulsant medications including:
    a. Phenytoin [Brand Name: Dilantin]
    b. Carbamazepine [Brand Name: Tegretol, Carbatrol, Equetro, Epitol]
    c. Oxcarbazepine [Brand Name: Trileptal]
    d. Primidone [Brand Name: Mysoline]
    e. Phenobarbital
6) Positive toxicology screen for any of the following drugs: cocaine, opiates, methadone, oxycodone, buprenorphine, benzodiazepines, barbiturates, amphetamines, methamphetamines, MDMA and PCP will be grounds for exclusion.
    a. Marijuana will be tested for but will not be an exclusionary criterion. Participants will be discouraged from smoking marijuana during the study.
    b. Participants with valid prescriptions for opiates, benzodiazepines, barbiturates, or amphetamines will not necessarily be excluded.
    c. Participants failing the toxicology screen will be allowed to re-screen once. These participants will need to be re-consented before being rescreened to ensure they have received adequate informed consent.
7) Not currently enrolled in a treatment program for opioid dependence and/or not currently stable on their methadone or buprenorphine dose,
8) Self-report of binge drinking alcohol (more than 9 days in the past 30 days, 4/5 drinks in a 2 hour period in females/males);
9) Systolic blood pressure < 90 or ≥ 160 mmHg;
    a. Participants failing for blood pressure will be allowed to re-screen once.
10) Diastolic blood pressure < 50 or ≥ 100 mmHg;
    a. Participants failing for blood pressure will be allowed to re-screen once.
11) Breath CO > 80 ppm;
12) Heart rate is greater than or equal to 115 bpm or less than 45 bpm;
    a. Participants failing for heart rate will be allowed to re-screen once.
13) Currently seeking treatment for smoking cessation;
14) Being pregnant or nursing, or not report using an approved form of birth control if applicable determined by the Project Medical Director.
15) Have used nicotine replacement, bupropion or other pharmacotherapies as cessation aids in the past month (bupropion will be allowed for treatment of depression);
16) Current symptoms of psychosis, dementia or mania;
17) Suicidal ideation in the past month (score > 1 on the BDI question 9 or endorse question 4 and/or 5 on the MINI suicide subscale);
18) Reporting a plan or attempt to commit suicide, which is assessed on question A3g of the MINI Neuropsychiatric Interview Major Depressive Episode Module. Thoughts of suicide without an intent or plan is not an exclusion criteria;
19) Suicide attempt in the past 6 months (endorse question 6 on the MINI suicide subscale with suicide attempt in the past 6 months);
20) Participation in another research study in the past 30 days;
21) Daily use of e-cigarettes in the past month (defined as 6 – 7 days per week); or
22) Co-habitation with any research participant who has or is participating in the current study.
23) Oxygen saturation of < 90%
24) Reporting positive symptoms for COVID19

Individuals under age 21 are excluded because they cannot legally buy cigarettes. Those with unstable medical, psychiatric, or medication conditions (as determined by the licensed medical professional) are excluded as these symptoms could affect a participant’s ability to

complete the study. Examples include but are not limited to the following: angina, stroke, heart attack which occurred since phone screening, blood clots in the arms or legs for which the individual is undergoing active medical treatment, cancer requiring active chemotherapy or radiation therapy, severe shortness of breath caused by conditions such as uncontrolled asthma, COPD, or arrhythmia, active untreated infection such as pneumonia, active untreated endocrine disorder such as hyperthyroidism. We will exclude those currently seeking smoking treatment and those who plan to quit in the next 30 days, as participation in this study may not lead to reductions in smoking. We will exclude pregnant or nursing women and women of reproductive potential who are unwilling to use acceptable forms of birth control throughout the study if applicable determined by the Project Medical Director. We will also exclude anyone with current or recent alcohol or drug abuse problems as these factors could independently affect smoking behavior during the study. Individuals with baseline CO readings greater than 80 ppm, those with heart rate or blood pressure readings that are out of range (systolic: 90-159 mmHg; diastolic: 50-99 mmHg; HR: 45- 114 bpm) and anyone who has attempted suicide in the past six months will be excluded from the study for safety concerns. Individuals who smoke ‘roll your own’ cigarettes exclusively will be excluded from the study because we will be unable to standardize their baseline smoking behavior. Individuals who have reported daily use of e-cigarettes in the past 30 days will be excluded as they may not be compliant with experimenter-provided e-cigarettes. Individuals who have recently participated in a research study will be excluded as participation may have changed their smoking patterns, which may preclude a stable smoking baseline. Because participants are required to complete portions of the protocol independently, they will need to be able to independently read and comprehend the study materials.

5.7 Eligibility Determination:

The research assistant will review the entire screening assessment battery for initial eligibility determination, confirming the participant meets the above described inclusion/exclusion criteria. All eligibility criteria that are not related to physiological measurements will be assessed during the first portion of the screening visit, and all criteria related to physiological measurements will be determined during the second portion of the screening. The final eligibility of the participant will be determined by a licensed medical professional (MD, DO, NP, PA, Master’s prepared RN or CRN) at each site after reviewing the Medical History Questionnaire, BDI, Mini Neuropsychiatric Interview, and the MINI suicide subscale. The licensed medical professional may meet with a participant if available and think it necessary for eligibility determination. He/she will sign off on eligibility prior to the first baseline visit. If the licensed medical professional determines the participant is not medically eligible to participate in the study, has current symptomatology that would interfere with interpretation of the data, or is unlikely to complete the study he/she will inform the research assistants who will contact the participant prior to the first baseline visit. The licensed medical professional will not need to review the medical history forms of participants who are ineligible for other, non-medical reasons.

If a participant fails the urine or saliva toxicology screen due to a prescription medication he/she is taking, then he/she will not be automatically excluded. The interviewer will make note of this when he/she submits the forms to the licensed medical professional for final eligibility determination.

Once all the screening procedures have been completed, researchers will pay participants $25 (+$25 bonus if applicable) for their time as long as they pass the drug tests and meet the minimum requirements for carbon monoxide or urinary cotinine levels. Participants will be paid after the completion of the study visit. If participants are deemed ineligible at any point in the screening, the participant will be paid after determined ineligible. Marijuana will be tested for but will not be an exclusionary criterion. If a participant does not pass the drug test but has a current, valid prescription that would explain the failed test he/she will not be automatically excluded and will still receive the visit payment. Participants who meet all other eligibility criteria, sans the medical criteria, will be scheduled for the first baseline visit.

At the end of the screening session, the researcher will complete the End of Visit Evaluation Form. This will allow the researcher to make note of any problems encountered during the visit and to assess the truthfulness of the participant in regards to self-report of tobacco use.

6. Study Baseline Procedures

This study will use a one-week, two-session baseline period to collect baseline individual difference measures and monitor daily usual-brand smoking behavior. At Baseline 1 or within 1 business day of the Baseline 1 visit, participants will be provided their usual brand cigarettes to smoke, equivalent to 150% of their daily smoking rate. Participants will be encouraged to come to the lab to pick up their usual brand cigarettes after they complete the questionnaires and physio for the BL1 visit. Those who cannot come to the lab will receive product via a commercial courier. A time line follow back (TLFB) will be used during the period between Baseline 1 and Baseline 2 to assess the daily cigarette use for the first 7 days the participant has product. The participant must have received their UB cigarettes from the lab before the 7-day assessment period starts, and Baseline 2 must occur at least 7 days after the participant receives their usual brand cigarettes from the lab. If the baseline period extends past seven days and if the participant has run out of product, participants will need to purchase their own usual brand cigarettes. Use of a two session baseline period will ensure stability of daily smoking reports, reduce reactivity to the daily cigarette monitoring, and reduce participant burden. During the two baseline sessions, participants will complete subjective questionnaires. Each visit will last approximately two to four hours. At the end of each baseline session, the researcher will complete the End of Visit Evaluation Form This will allow the researcher to make note of any problems encountered during the visit and to assess the truthfulness of the participant in regards to self-report of tobacco use. Participants will also be supplied with saliva test equipment and urine collection equipment during the Baseline 1 product exchange so that they can collect saliva and first void urine samples during the Baseline 2 visit.

For the Baseline 1 visit and all subsequent visits, the participant will be sent a REDCap link within 15 minutes of the start of the scheduled visit to complete all of the non-interviewer administered questionnaires. The participant will complete these questionnaires on their own but can have the research assistant present on a video call if they desire. Before beginning the physiological assessment portion of the visit over video call, the research assistant must review the participant’s questionnaire responses for that visit. Participants will be compensated after the completion of the study visit and when the participant has received their new product.

At Baseline 2 and all subsequent visits, after the participant has answered the questionnaires and has completed the physiological portion of the visit over video call, participants will be asked to come to the lab for a curbside exchange of product and biological samples. Participants will bring in their used and unused product from the previous visit as well as a first-void urine sample for assessing tobacco-related toxin exposure and a salivary sample for assessing nicotine metabolism rate on applicable visits (BL2, Week 8 and Week 16) using equipment that was provided at the previous visit. Participants will be instructed to call the RA at the office when they get to the clinicto ensure that there is enough space in the smoking chambers to house all participants while abiding by safety guidelines as detailed on page 14 of the protocol. All participants must pass a COVID19 screening before entering the building. When invited into the lab, the participant will be shown to a smoking chamber and will instructed to place their bag of product outside of the chamber. The participant will wait here while the RA processes and returns product through the randomization database. Then the RA will dispense new product and bring the bag back to the participant. When the RA deems it safe for the participant to exit the chamber, the RA will instruct the participant that they can leave. The RA will instruct the participant to observe social distancing measures during this exchange, providing clarification if necessary. If a participant forgets their first-void urine sample at the Baseline 2 visit, staff will ask participant to come back to the clinic with their first void urine sample before exchange of product occurs. If participant is unable to return to the clinic with their first void

sample, staff can arrange to meet the participant off campus to pick up their urine sample and to give participant their study product. Distancing and safety measures as described above must be observed. For participants who cannot make it to UHC, special arrangements will be made to enable use of the randomization database and product return/distribution procedures to the extent possible. Each week, during - or scheduled as nearly as possible to – a virtual visit, a complete accounting of the participant's product inventory will be taken and processed remotely through the randomization database. The participant will separate product based on its type (e-cigarette or combustible inventory) and status (used/unused), and the RA will process return characteristics through the database accordingly. The RA will clarify barcode characteristics with the participant when legibility is compromised. The participant will be instructed to keep unused product in their possession, but to exchange any used product with the courier who will deliver newly dispensed replacement products within 48 hours.

Product that will be given to participants for the Baseline 2 visit cannot be given/sent to participants until 7 days have passed following completion of the Baseline 1 visit. We will need to calculate baseline smoking rate during this 7-day period and so participants cannot have access to any blinded study product or e-cigarettes before this 7-day period has ended.

6.1 Visit scheduling requirements for baseline period:

Participants will be required to schedule the Baseline 1 visit within 30 days of the completion of their screening visit. If a participant still wants to be in the study after 30 days, he/she will need to be re-screened. The participant will need to be re-consented but will maintain the original REDCap Subject Identifier. The ideal target window separating Baseline 1 and Baseline 2 is between 7 and 12 days. The minimum is 7 days and the maximum is 21 days. If the participant does not complete the visit within 21 days, then he/she will not be rescheduled and will be discontinued from the study.

6.2 Measures/Assessments

The following physiological measures will be collected and recorded directly into REDCap by the interviewer:

1) CO
2) Blood Pressure
3) Heart Rate
4) Body temperature
5) Oxygen saturation
6) Respiratory rate
7) Urine or Saliva Toxicology
8) Urine Pregnancy test (if applicable, to be performed every 2 weeks)

The following assessments will be administered as an interview at Baseline 1 and entered into REDCap by the interviewer at the end of the visit:

1) <u>Concomitant Medications Form</u>
2) <u>Health Changes Questionnaire,</u> which will assess any weekly health changes,
3) <u>Time Since Last Cigarette Questionnaire</u>
4) <u>Maintenance Drug Dose Questionnaire</u>

The following Baseline 1 assessments will be completed by the participant directly in REDCap:

1) <u>BDI</u>
2) <u>OASIS</u>
3) <u>Respiratory Symptom Questionnaire</u>
4) <u>COVID19 Symptom Questionnaire</u>
5) <u>Wisconsin Inventory of Smoking Dependence Motives-Brief Scale (WISDM)</u> will be

administered to assess nicotine dependence severity.

6) Perceived Health Risks Rating[112], a measure of the perceived addictive potential and other health risks associated with cigarettes;
7) Perceived Stress Scale (PSS)[112], assessing the degree to which life situations are perceived as stressful;
8) Positive and Negative Affect Scales (PANAS)[113], a measure of changes in positive and negative mood;
9) Respiratory Health Questionnaire, a UVM measure of cough, shortness of breath and other respiratory symptoms;
10) Minnesota Nicotine Withdrawal Scale (MNWS)[114], a measure of nicotine withdrawal;
11) Questionnaire of Smoking Urges-brief scale - Usual Cigarette (QSU)[115], which measures the urge to smoke;
12) Vaping Craving Questionnaire (VCQ)[116] which measures the urge to vape;
13) Cigarette Evaluation Scale – Usual Cigarette (CES)[117], which measures responses to cigarettes (e.g., reward, satisfaction);
14) Vaping Evaluation Scale (VES), which measures responses to vaping (e.g. reward, satisfaction)
15) Cigarette Purchase Task – Usual Brand Version (CPT)[118], a self-report analogue of a progressive-ratio schedule that measures the relative reinforcing efficacy of cigarettes by querying how many of that day's cigarette they would consume in a day at varying prices. This task will indicate whether prolonged VLNC cigarette use reduces cigarette demand and increases sensitivity to increases in cigarette costs;

All participants will also be asked to select their top three flavors of e-cigarette liquid from a list read to them by the RA. This question will be asked in preparation for giving flavored pods to participants who are randomized into the flavored e-cigarette condition. This information will be recorded directly into REDCap. Participants will be asked to rate these top three flavors based on either previous experience with these flavors or to indicate how much they believe that they will like or dislike the flavors.

Physiological measures will be collected at Baseline 2 and entered directly into REDCap by the interviewer:

1) CO
2) Blood Pressure
3) Heart Rate
4) Body temperature
5) Oxygen saturation
6) Respiratory rate
7) Urine or Saliva Toxicology
8) Urine Pregnancy (if applicable, to be performed every 2 weeks)

The following assessments will be administered as an interview at Baseline 2 and entered directly into REDCap by the interviewer:

1) Concomitant Medications Form
2) Health Changes Questionnaire
3) Time Since Last Cigarette Questionnaire
4) Maintenance Drug Dose Questionnaire

The following assessments will be administered at Baseline 2 and completed by the participant directly in REDCap:

1) BDI
2) OASIS

3) COVID19 Symptom Questionnaire
4) Respiratory Symptom Questionnaire
5) MNWS
6) WISDM
7) PANAS
8) QSU (usual brand)
9) Vaping Craving Questionnaire
10) CES (usual brand)
11) Vaping Evaluation Scale
12) CPT (usual brand)
13) E-Cigarette Flavor Rating Questionnaire
    a. Participants will rate only the flavors that they received from staff for this visit

In the event that the REDCap website is not functioning, the assessments will be administered aloud and participant answers will be recorded securely. The interviewer will enter the data into REDCap when it resumes functioning properly. This information should be recorded in the 'End of Visit Evaluation Form'.

6.3 E-cigarette Training Session (Baseline 2):

Participants assigned to an e-cig condition will be told that they will be provided with a JUUL. If a participant indicates an unwillingness to use the JUUL device, the research assistants will offer the participant the Vuse Solo as alternative device. If the participants does not wish to use either device, he or she would be ineligible for the study.

Participants in the e-cigarette conditions will be given e-cigarette pods BEFORE the training session occurs. These pods will be either picked up at the lab (preferred) or delivered to the participant 1 to 2 days before their Baseline 2 visit occurs. Participants randomized to the e-cigarette conditions will be notified before their Baseline 2 visit (but after the 7-day period following Baseline 1) and informed of their e-cigarette condition. Participants randomized to the flavored condition will be given pods of up to three flavors of their choice. Staff will calculate how many total pods participants will be given at this time based on their smoking rate, and participants will be able to choose the proportion of each flavor that they would like to receive.

The e-cigarette training session will occur over video chat after the physiological measurements have been collected. The first 30 minutes of the training session will consist the participant being taught how to use, charge, and replace pods. Participants will first try their JUUL using the tobacco flavor. At this point, participants who are in the tobacco-only flavor condition will conclude their training session.

For all visits following Baseline 2, participants in the preferred flavor condition, participants are permitted to take up to three flavors home per week, but can choose to take less than three flavors if desired. Participants will take home their chosen pods or cartridges of up to three flavors. Participants will be able to change their flavors at only one point in the study if they desire. Participants will only be allowed to take home three flavors at one time. Participants will be given an E-cigarette instructional manual that reviews the e-cigarette training done at this visit. Participants will be encouraged to call with any device issues.

6.4. Interactive Voice Response System:

At the end of the first baseline visit, participants will be trained to use the Interactive Voice Response (IVR) System, which will contact participants each day throughout the study and ask about their smoking behavior as well as withdrawal symptoms the week before and after Baseline 2. We will also review the IVR adherence incentive program, which consists of $1 per call plus a $10 bonus for seven consecutive calls.

The IVR system is operated by TeleSage (https://telesage.com/about/). To be enrolled in the IVR system, research staff will enter the participant's initials, telephone number, subject identifier, and visit dates into the IVR TCORS website. Identifying information (initials and telephone numbers) will not be extracted as part of the data by the bioinformatics group. Please refer to TeleSage's privacy statement and HIPAA compliance form for additional information.

Baseline 2 biological specimens:

1) Urine sample for smoking biomarker assessment**:**
Participants will be asked to provide a urine sample (first void of the day) at the second baseline session and to post-randomization weeks 8 and 16 for biomarker assessment. Biomarker analysis will provide nicotine and carcinogen exposure outcome measures and verify compliance with VLNC cigarettes. Samples will be stored at -80C. Urine samples will be analyzed for total nicotine (cotinine plus its glucuronide conjugate, a useful measure of daily nicotine exposure), the tobacco-specific nitrosamine 4-methylnitrosamineo-1-(3-pyridyl)-1-butanol (NNAL), and metabolites of 4 polycyclic aromatic hydrocarbons (PAHs), which are biomarkers of tobacco smoke carcinogens and decrease upon tobacco cessation or reduction. Anatabine is a minor alkaloid that is reduced in users of VLNC cigarettes and e-cigs. Therefore, anatabine levels in samples from those assigned to the VLNCC, VLNCC+TF e-cig and VLNCC+PF e-cig conditions should be lower than levels from those in the NNCC condition. These analyses will be preformed by the Murphy lab at the University of Minnesota.
2) Saliva Samples for smoking biomarker assessment:
We will collect a saliva sample at the second baseline session and post-randomization weeks 8 and 16 for analysis of nicotine metabolite ratio (NMR; ratio of 3-hydroxycotininine [3 HC] to cotinine [COT]), a phenotypic marker of nicotine metabolic rate. Analyses of these samples will be performed by the Tyndale lab.

Biomarker shipping and storage:

Biomarkers will be shipped quarterly to the University of Vermont Laboratory for Clinical Biochemistry Research (Tracy Lab). The Tracy Lab will serve as a central repository for all biomarker specimens and will be responsible for distributing specimens to the appropriate labs on a quarterly basis. Urine samples will be analyzed and stored at the University of Minnesota Murphy Lab. The saliva samples will be analyzed and stored at the University of Toronto Tyndale Lab.

7. Study Experimental Procedures

7.1. Experimental Period:

Participants will be seen weekly throughout the 16-week experimental period. Weeks 4, 8, 12, 16 and the abstinence visit will take approximately 2-4 hours each. All other sessions will last approximately 2 hours. If a participant has a positive urine or saliva toxicology test or is visibly intoxicated as determined by slurred speech, swaying, or stumbling, the session will be rescheduled until a negative test result is obtained and intoxication is not present. As a part of each experimental visit, participants will be asked to come to UHC for a product exchange. All participants must pass a COVID19 screening before entering the building. Participants will be instructed to contact the RA at the office when they get to the clinic to ensure that there is enough space in the smoking chambers to house all participants while abiding by safety guidelines as detailed on page 14 of the protocol. All participants must pass a COVID19 screening before entering the building. When invited into the lab, the participant will be shown to a smoking chamber and will instructed to place their bag of product outside of the chamber. The participant will wait here while the RA processes and returns product through the randomization database. Then the RA will dispense new product and bring the bag back to the participant's smoking chamber and leave it on the ground in front of the chamber. When the RA deems it safe for the participant to exit the chamber, the RA will instruct the participant that they can leave. The RA will instruct the participant to observe social distancing measures during this exchange, providing clarification if necessary. At the end of each experimental session, the researcher will complete the End of Visit Evaluation Form, which will be filed in the participant's binder. This will allow the researcher to make note of any problems encountered during the visit and to assess the truthfulness of the participant in regards to self-report of tobacco use and compliance to study procedures.

Visit scheduling requirements for experimental period:

The ideal scheduling window between each visit is 7 days based on the date of the Baseline 2 Visit. For additional scheduling requirements, refer to the '*Scheduling Visits SOP*'. If a participant misses a visit and is unable to reschedule during the window (± 3 days), that visit will not be 'made-up' in the future. All measures that were not completed will be considered missing data and will not be collected during future visits. If a visit mistakenly occurs outside of the designated window, this is a protocol deviation. A 'Non-Medical Event Form' will need to be completed. Additionally, each visit should occur at approximately the same time of day ± 1 hour. The interval between the time for the participant's methadone/buprenorphine dose should also be consistent for each visit (± 1 hour).

If a participant is not able to attend his/her Week 16 visit, then it should be rescheduled even if it is outside of the scheduling window. This will be documented as a protocol deviation.

7.2. Experimental Visits Weeks 1, 3, 5, 7, 9, 11, 13, and 15 Procedures

7.2.A. Measures/Assessments

Physiological Measures Collected and entered directly into REDCap by the interviewer:

1) CO
2) Blood Pressure
3) Heart Rate
4) Body temperature
5) Oxygen saturation
6) Respiratory rate
7) Urine or Saliva Toxicology
8) Urine Pregnancy test (if applicable, to be performed every 2 weeks)

The following assessments will be administered as an interview and will be entered directly into REDCap by the interviewer:

1) Concomitant Medications
2) Medical Event Form, if applicable
3) Health Changes Questionnaire
4) Time Since Last Cigarette Questionnaire
5) Maintenance Drug Dose Questionnaire

The following assessments will be completed by the participant directly in REDCap:

1) BDI
2) OASIS
3) COVID19 Symptom Questionnaire
4) Respiratory Symptom Questionnaire
5) MNWS

In the event that the REDCap website is not functioning, the assessments will be administered aloud and participant answers will be recorded securely. The interviewer will enter the data into REDCap when it resumes functioning properly. This information should be recorded in the 'End of Visit Evaluation Form'.

7.3. Experimental Visits Weeks 2, 4, 6, 8, 10, 12, 14, and 16 Procedures:

7.3.A *Measures/Assessments*

Physiological measures collected and entered directly into REDCap by interviewer:

1) CO

2) Blood Pressure
3) Heart Rate
4) Body temperature
5) Oxygen saturation
6) Respiratory rate
7) Urine or Saliva Toxicology
8) Urine Pregnancy test (if applicable)

The following assessments will be administered as an interview and will be entered directly into REDCap by the interviewer:

1) Concomitant Medications
2) Medical Event Form, if applicable
3) Health Changes Questionnaire
4) Time Since Last Cigarette Questionnaire
5) Maintenance Drug Dose Questionnaire

The following assessments will be completed by the participant directly in REDCap:

1) BDI
2) OASIS
3) Respiratory Symptom Questionnaire
4) COVID19 Symptom Questionnaire
5) MNWS
6) QSU (usual brand)
7) QSU (study cigarette)
8) Vaping Craving Questionnaire
9) CES (usual brand)
10) CES (study cigarette)
11) Vaping Evaluation Questionnaire
12) PANAS
13) Cigarette Purchase Task - Usual Brand Cigarette Version (weeks 4, 8, 12 and 16 only)
14) Cigarette Purchase Task – Study Cigarette Version (weeks 4, 8, 12 and 16 only)
15) Cross-price Elasticity Task[120]- e-cigarettes and combustible cigarettes (weeks 4, 8, 12 and 16 only) (for e-cigarette experimental conditions only)
16) Penn State Electronic Cigarette Dependence Index (weeks 8 and 16 only)
17) Respiratory Health Questionnaire (weeks 8 and 16 only)
18) FTND (weeks 8 and 16 only)
19) Perceived Health Risks Questionnaire (weeks 8 and 16 only)
20) Smoking Stages of Change Algorithm and Contemplation Ladder (weeks 8 and 16 only)
21) WISDM – Brief Scale
22) Drug Use Questionnaire – 1 month version (weeks 8 and 16 only)
23) Perceived Stress Scale (weeks 8 and 16 only)
24) Alcohol Use Questionnaire – 1 month version (weeks 8 and 16 only)
25) E-cigarette flavor rating questionnaire (weeks 4, 8, 12 and 16 only)

In the event that the REDCap website is not functioning, the assessments will be administered aloud and participant answers will be recorded securely. interviewer will enter the data into REDCap when it resumes functioning properly. This information should be recorded in the ‘End of Visit Evaluation Form’.

Biological Samples to be collected:

1) First void urine sample (Weeks 8 and 16 only)
2) Saliva sample (Weeks 8 and 16 only)

7.4. Interactive Voice Response System:

Participants will continue to use the IVR system on a daily basis throughout the experimental period to record the number of study cigarettes smoked per day, measurement of e-cig use and use of non-study cigarettes or other tobacco products. Measurement of e-cig use will be collected by asking two questions: how many daily e-cigarette episodes occurred, where one episode consists **of around 10-15 puffs** or up to approximately 10 minutes, and what proportion of pods and/or cartridges were used per day. Participants will also be asked to log how many flavors of e-cigs they used per day. During the first week after Baseline 1, the IVR system will collect information about mood and withdrawal symptoms.

7.5. Variable Incentive Program:

An incentive program has been developed with the goal of improving attendance at scheduled assessment sessions, compliance with using only study-provided tobacco products, and encouraging honest self-reports regarding all nicotine/tobacco use.

Briefly, participants will receive a total of seven tickets for each weekly visit they attend after randomization (Visits 03-18, weeks 1-16). In total, participants could earn 112 valid tickets across the 16 visits. Participants will be instructed that these tickets correspond to attendance (one ticket), honest reporting (one ticket), compliance in bringing back used and unused pods/cartridges (two tickets) and adherence to using only the assigned study product (three tickets). Participants who do not bring back all of their unused study product and used packaging will be told that they may not be eligible to earn the two compliance tickets.

Participants will be further instructed that all of the tickets that they receive “could” be eligible for entry into a monthly drawing for prizes, but that only tickets that are “validated” will be eligible for prizes.

Since it is prohibitively expensive to test urine samples each week for each participant and because it is currently not feasible to detect with reasonable precision non-compliance based on biomarkers in the two higher nicotine group, we plan to only validate the attendance tickets. Hence, each participant who attends their regularly scheduled weekly session will have a total of seven validated tickets entered into the monthly drawing.

To convey the message that we may be validating honest reporting and use of only study-provided products, in a bogus pipeline of sorts, we will tell the participants that a composite assessment of the measures that we collect MAY be used to validate the amount of nicotine and tobacco products that they are using. So there is some minor deception involved, but technically we could conduct urine toxicology testing for both purposes. Hence, if the urine toxicology testing is presented as something that MAY be done for validation purposes, we feel that any deception is relatively minor. For scientific/economic reasons we are just electing to restrict validation to attendance. Nevertheless, we will debrief all participants upon the completion of the trial. We will inform them that the incentive program was based exclusively on attendance due to the relatively high cost of urine toxicology testing and other practical problems with shipping the urines for prompt testing.

Drawings will be conducted on the 1st of each month. Validation will be performed by staff who have no participant interaction and are not blind to condition. Any ticket drawn will be eligible for an incentive as the only true contingency is for attendance. There will be no mention of the basis for earning incentives (i.e., whether the ticket was for attendance, honesty, adherence). Participants will simply be informed that he or she earned an incentive from the drawing.

Each drawing will be independent (without replacement); consequently, some participants will not win a prize and others may win more than one during the study if more than one of their tickets is drawn. After confirming winners, the remaining tickets from each month will be discarded (i.e., tickets will only be entered into one drawing). The monthly prize amounts are detailed below.

We estimate based on the 2 ½ years we think it will take to complete this study, that participants will win an average of approximately $65 in prizes or an additional $5.50 per week per participant.

Grand Prize (1): $500 cash
Second Prize (1): $200 cash
Third Prize (5): $10 cash

7.6. Product and Procedures Compliance Review Sessions:

At each visit, Baseline 2 through Week 16, participants will be counseled about their use of the study cigarettes and assigned e-cigarette (if applicable). Participants will be asked about any concerns or obstacles associated with use of the study cigarettes and assigned e-cigarette (if applicable). The importance of honest self-reporting will be stressed. Participants will be told that they will not be penalized for use of other nicotine or tobacco products and that it is crucial for them to report any use of these products. If difficulties are encountered, participants will be asked why they think they are experiencing difficulties (e.g., taste, withdrawal symptoms) and to problem-solve how to deal with these difficulties in order to meet the protocol requirements. Additionally, participants will be counseled about their IVR completion, visit attendance, task engagement and product accountability. Refer to the '*Product and Procedures Compliance Review Sessions SOP*' for more information.

7.7. Quit Attempts During the Study Protocol:

At each weekly session, we will ask each participant if s/he is currently abstaining from smoking with the intention of quitting and whether s/he is planning to quit smoking prior to his/her next scheduled visit. If a participant is currently abstaining from smoking with the intention to quit, we will encourage the participant to continue abstaining, schedule them for weekly visits, and provide them with NCI's Clearing the Air manual and local smoking cessation resources. We will give them the option of taking study product(s) home but not require that they take them, and if they do take the product(s) home we will suggest that they put the product(s) away at home so as to remove these cues from view. We will ask the participant to contact staff if they lapse and would like to receive study product(s) prior to his/her next visit. If a participant is planning to quit but has not initiated a quit attempt, we will ask if s/he has identified a quit date and if so what the date is, provide them with the Clearing the Air manual and local smoking cessation resources, provide them with the study product(s), and recommend that they put the product(s) away out of view on the quit date.

For those in a condition including e-cigarettes, we will defer to the participant's interests in continuing to use e-cigarettes as part of their quit attempt. Those who indicate that they will continue to use them will be given their same weekly supplies base on their baseline smoking rate. Those who indicate that they are planning to abstain from both combusted and non-combusted tobacco, we will honor that request. As we state above about combusted cigarettes, if participant changes his or her mind about resuming e-cigarette use, they can contact us and obtain their weekly supply.

7.7.A. If a participant is currently abstaining from smoking with the intention to quit:

- Encourage participant to continue abstaining from smoking
- Schedule the participant for normal weekly visits
- Provide the participant with the '*Clearing the Air*' manual and local smoking cessation resources
- Give the participant the option to receive study product rather than require him/her to take the product
- If the participant choses to receive the study product have him/her sign a form acknowledging that cigarette availability could be detrimental to the quit attempt. Recommend that he/she put the product "away" at home as to avoid unwanted cues to smoke.
- If the participant chooses not to receive the study product, have him/her contact the lab if he/she lapses and would like to pick up or be mailed the study product prior to his/her next visit.

7.7.B. If a participant is planning to quit smoking, but has not initiated the quit attempt:

- Ask if he/she has identified a target quit date and, if so, what that target date is
- Provide the participant with the '*Clearing the Air*' manual and local smoking cessation resources
- Provide the participant with the study product as usual. Recommend that on the target date he/she put the product "away" at home as to avoid unwanted cues to smoke.

7.8. Abstinence Assessment Session:

After the week 16 visit, participants will be required to attend one additional visit the following day. During this visit, participants will have been encouraged to abstain from smoking until their next scheduled visit (approximately 24 hours later). The abstinence assessment session should be scheduled no less than 18 hours and no more than 30 hours after the Week 16 visit. Abstinence will be verified by an expired breath carbon monoxide level of less than or equal to 6ppm. This session will allow us to determine whether the experimental cigarettes and e-cigarette use (for the e-cigarette conditions) have reduced the effects of abstinence on these measures relative to the control conditions. If the participant does NOT meet abstinence criteria, he/she will only receive $20 for the visit. Those who do meet abstinence criterion will do concurrent choice session detailed below.

7.8.A Participants Who Meet Criteria for Abstinence

7.8.A.1 Measures/Assessments

Physiological measures collected and entered directly into REDCap by the interviewer:

1) CO
2) Blood Pressure
3) Heart Rate
4) Body temperature
5) Oxygen saturation
6) Respiratory rate
7) Urine or Saliva Toxicology

The following assessments will be administered as an interview and will be entered directly into REDCap by the interviewer:

1) Concomitant Medications
2) Medical Event Form, if applicable
3) Health Changes Questionnaire
4) Time Since Last Cigarette Questionnaire
5) Maintenance Drug Dose Questionnaire

The following assessments will be completed by the participant directly in REDCap:

1) BDI
2) OASIS
3) COVID19 Symptom Questionnaire
4) Respiratory Symptom Questionnaire
5) MNWS
6) PANAS
7) QSU-brief - Usual Cigarette
8) QSU-brief - Study Cigarette
9) Vaping Craving Questionnaire
10) Cigarette Purchase Task - Usual Brand Cigarette Version

11) Cigarette Purchase Task - Study Cigarette Version
12) E-cigarette Purchase Task- E-cigarette Version

In the event that the REDCap website is not functioning, the assessments will be administered aloud and participant answers will be recorded securely. The interviewer will enter the data into REDCap when it resumes functioning properly. This information should be recorded in the 'End of Visit Evaluation Form'.

7.8.B. Participants Who Do Not Meet Criteria for Abstinence

7.8.B.1 Measures/Assessments

Participants who do NOT meet abstinence criteria will be required to complete the following assessments:

1) CO
2) Blood Pressure
3) Heart Rate
4) Body temperature
5) Oxygen saturation
6) Respiratory rate
7) Urine or Saliva Toxicology

The following assessments will be administered as an interview and entered directly into REDCap by the interviewer:

1) Concomitant Medications
2) Health Changes Questionnaire
3) Medical Event Form, if applicable
4) TLFB

The following assessments will be completed by the participant directly in REDCap:

1) BDI
2) OASIS
3) COVID19 Symptom Questionnaire
4) Respiratory Symptom Questionnaire

7.9. Participant Compensation:

Participants will receive $25 for the medical consent visit. Participants will receive $25 plus a $25 bonus for completing each screening visit, plus an additional $25 bonus for completing the visit on time as scheduled. Payment for the first screening session will be made upon its completion. Payment for the second session will be made regardless of enrollment as long as the participant passes the drug test and meets the minimum requirements for carbon monoxide or urinary cotinine levels. Participants who do not pass the drug test or who are visibly intoxicated as determined by slurred speech, swaying, or stumbling will not be able to complete the visit and will be asked to take another test several days after the first positive. If they are negative for the second test, they will be eligible to participate, and if they are positive the for the second text they will be excluded. Participants will receive $100 for each study visit from Baseline 1 to Week 16. Participants will also have a chance to earn an additional $20 bonus for every study visit that is completed on time as scheduled starting at Week 1 and ending at Week 15. Participants can receive up to $120.00 for the abstinence session ($20 if participant does not achieve abstinence, $120 if participant reaches abstinence), $40 for biochemical verification of abstinence, and up to $306 for completing daily IVR reports of study cigarette and other nicotine and tobacco use.. There will also be a $150 bonus distributed at Week 16 for completing the study. Participants who

do not complete the entire study will receive compensation for the sessions that they do complete. Total compensation for completing Study 3, including study visit payments, daily IVR calls and end of study bonus is $2841. As mentioned above, participants will have a chance to earn additional incentives for compliance, honesty and attendance through urine testing. Participants will be given a debit card at the beginning of the study (during the second portion of the screening visit) and compensation for each visit will be automatically transferred to the card after they complete that visit. If debit cards are unavailable, participants will be paid via an alternate method (i.e. cash or check).

7.10. End of Study:

After a participant has completed all study procedures and has been paid for participation the research assistant will read the following script and give the participant the *Clearing the Air Manual.*

"If you've reduced your smoking during this study, we encourage you to continue these reductions or even consider quitting. We would like to provide you with some resources should you decide to try to abstain from smoking (give "Clearing the Air" and hotline information). Please also feel free to consult with your physician and use any medications he/she deems appropriate. We will call you in approximately 30 days to ask about your smoking since leaving the study. There is no right answer and we know how difficult quitting can be. Please just answer honestly. The call will take less than 5 minutes. Thanks again for your participation."

The following assessments will be administered using REDCap:

1) <u>End of Study Questionnaire</u>

7.11. 30-Day Follow up Phone Call:

Participants will receive a follow-up phone call between 25 and 35 days after the abstinence assessment session to assess their smoking patterns. The phone questionnaire will last less than five minutes. The questionnaire will ask if the participant is still smoking, how much and whether he/she has attempted to quit smoking since the end of the study. Participants will receive 5 variable incentive program lottery tickets for completing the call as compensation.

Those who report abstinence will be invited to complete biochemical verification and be compensated $40 for doing so. Abstinence will be achieved by a carbon monoxide reading of 6 parts per million (ppm) or under. A urine sample may also be collected to be sent to the lab for analysis. Additionally, any Medical Event Forms that remain open from the last session will be discussed. If the participant became pregnant during the study, this would have been recorded as a medical event. During this phone call, the research assistant will confirm her due date. This event will remain open until delivery. At that time the licensed medical professional will contact the participant to ask a few questions about the baby's health and will update the Medical Event Form.

Once a participant has completed all study procedures and all open events have been closed, the PI or Project Manager will review the participant's record and sign a form indicating study completion for that participant.

8.0 Study Randomization

8.1 Randomization Process

At the end of Baseline Session 2, participants will be randomized in equal probability to one of the four groups (NNCC, VLNCC, VLNCC + TF e-cig, VLNCC + PF e-cig) for a 16-week period, with stratification by site and menthol cigarette status. Participants will be randomized, using block randomization, in equal number to the dose conditions, with randomization stratified by study site and menthol status. Each site will randomize participants until the total goal of 310 participants across both sites is reached (205 at UVM and 105 at Johns Hopkins), and no effort will be made to recruit a specific number of menthol and non-menthol smokers at each site.

The lead statistician will create a randomization schedule for each of the two sites,

amounting to 150% of expected enrollment at each site. The excess randomization codes will be used in the event that a site will have to enroll extra participants due to unexpectedly slow enrollment at another site. The nicotine doses will be identified by letter code and only Administrative Core personnel with no participant contact will have the link between the statistician's letter code and dose assignments. There will be no blinding of e-cigarette conditions. The Administrative Core will maintain the randomization schedule and the link between the alphabetic code and treatment assignment securely. A second sealed copy will be secured in a separate building to protect against loss related to fire or other unforeseen events.

The University of Vermont will be responsible for removing all identifying information from cigarettes received from the Research Triangle Institute (RTI), labeling each carton with a blind code, assigning product using this blind code based on the randomization schedule being provided by the UVM Biostatistics Core, and shipping cigarettes and e-cigarettes to each site as needed based on recruitment. Each site will be responsible for tracking product received and distributed to participants, collecting unused product from participants, and returning unused cigarettes and e-cigarettes to UVM. The participants, investigators and study staff will not have knowledge of which product is given to a participant or whether different participants received the same or different product.

8.2 Study Product Administration

During the experimental period, participants will be provided with a 14-day supply of research cigarettes equivalent to 150% of their daily smoking rate. Those in the e-cigarette conditions will also be provided with a 14-day supply of e-cigarettes equivalent to their daily smoking rate. This rate will be calculated at Baseline 2 and will be an average daily smoking rate based on the IVR data that reports on the usage for the first seven days following the day of the first baseline visit. This will ensure adequate availability of cigarettes in the numerous locations participants may typically keep a supply (home, work, vehicle, etc.) as well as avoid expending the entire supply if they miss a scheduled visit. Participants will be instructed to use the research cigarettes for 16 weeks, at which point they are to discontinue product use.

If there is prior knowledge a participant will be missing a visit (i.e. planned vacation, laboratory closure, etc.), then the participant will be provided with an adequate supply of cigarettes to make up for the missed visit(s). The participant will be given a 21-day supply if one visit is going to be missed and a 28-day supply if two visits are going to be missed.

8.3 Guidelines for Reporting other Nicotine Product Use

Participants will be asked to refrain from use of other non-study cigarettes during the study period. If participants have to use another nicotine product, they will be told to use a non-combustible product (gum, patch, etc.). Additionally, they will be told there is not a penalty for use of non-study products, and that it is crucial for them to report any use of non-study tobacco products. Throughout the baseline and experimental periods, an Interactive Voice Response (IVR) system will be used on a daily basis to record the number of study cigarettes and non-study cigarettes used the previous day. During the baseline and first experimental week, participants will also answer daily IVR questions about their mood. Participants will be seen weekly for assessments. Brief standardized review sessions focusing on compliance with the study cigarettes and other study procedures will be provided at each visit.

8.4 Product Accountability:

Participants will be required to keep track of all the products provided to them. Therefore, they will be instructed to return all unused products and empty cigarette packs e-liquid pods/cartridges to the laboratory each week. Research staff will complete the 'Product Accountability Log' as they process participants' product. Any discrepancies in the product dispensed versus product returned will be discussed and recorded in the log. Research staff will weigh all opened e-cigarette pods /cartridges that the participant returns at all visits to determine how much e-liquid was used since the participant was last seen. Empty cigarette packs and e-liquid pods/cartridges will not be saved. Unused cigarette packs and e-liquid pods/cartridges will be re-distributed to the participants during Weeks 1-15. During Week 16, remaining unused cigarettes and e-cigarette pods/cartridges returned by the participants will be collected by the research staff.

Participants who report running out of cigarettes or e-liquid pods/cartridges prior to a scheduled weekly visit will be allowed to come in for an unscheduled visit to obtain more research cigarettes. To determine whether a rate change for cigarettes is necessary, we will look at the past two CO levels as compared to the Baseline 2 CO. If the CO trend is consistent with the self-report of smoking all of the allotted cigarettes then a rate increase will be granted. The participant will then receive cigarettes at a rate of 175% of their daily smoking rate. The maximum increase is 200% of their daily smoking rate. To determine whether a rate change for e-cigarettes is necessary, we will monitor the amount of e-cigarette use the participant is reporting and showing through product return along with any unscheduled visits. The investigator may grant an e-cigarette rate increase in increments of 25%. The maximum increase for e-cigarettes will be 200% of their baseline weekly e-cigarette dispensation rate.

If participants lose more than two packs of cigarettes and/or pods/cartridges and require an unscheduled visit to the laboratory to supplement their supply, they will be told the next time they lose more than two packs they will have to wait until their next scheduled appointment to receive more cigarettes.

9. Study 3 Statistical Methods and Sample Size

9.1 Statistical Methods

Continuous outcomes will be summarized by mean, standard deviation, median and range. Categorical outcomes will be summarized by frequencies and percentages. Skewed continuous outcomes will be log- or square-root transformed as appropriate. Variables measured at each baseline visit will be averaged and the average will be used as the baseline measurement.. As we expect conditions to be balanced on important baseline characteristics due to randomization, our primary analysis for all endpoints will not be adjusted for potential confounders. However, a secondary analysis will be completed for all outcomes adjusting for demographic characteristics (e.g., age), that we have found to be important in prior studies. Potential moderators (e.g., SSRI vs. non-SSRI antidepressant, depression vs. anxiety disorders, BMI above or below 30) will be explored by adding that term and the moderator-by-condition term to the model. We will examine age group and gender as potential moderators in a similar fashion.

Participants will be randomized in equal probability to one of the four conditions, with randomization stratified by site and menthol cigarette status. All analyses will follow the intent- to-treat principle (i.e. subjects will be analyzed according to condition assignment, regardless of compliance). The Primary Aim will examine the effects of condition on total CPD (study product and non-study product). CPD will be analyzed by week (mean over all days in a seven-day period) using a mixed model to account for repeated measures from the same individual.

Models will include baseline CPD as a covariate. Using a mixed model also allows us to include the effect of study site as a random effect. Additional analyses conducted using data collected at the end of the study will use orthogonal comparisons to test for a linear trend in the decrease in CPD, such that VLNCC + PF e-cig > VLNCC + TO e-cig > VLNCC > NNCC, with the largest reduction in the VLNCC + PF e-cig condition. As we expect that differences among conditions for some of the outcomes may not follow a linear pattern, we will use related planned comparisons to test for threshold effects, specifically contrasting the NNCC condition to all three VLNCC conditions, and NNCC to the two VLNC + e-cigarette conditions. Analysis of cigarette demand, smoke exposure and tobacco carcinogens (Aim 2) as well as additional outcomes, including subjective effects, will be analyzed in a similar manner. Because Exploratory Aim 3 is based on abstinence-induced effects and will be examined using data collected at a single visit at Week 17, analysis will be based on an analysis of co-variance model. In addition to the effect of condition, we will include important covariates noted above. Exploratory analyses will also be conducted combining data collected from three of the vulnerable populations (disadvantaged women of childbearing age, opioid dependent individuals, individuals with AD) to explore potential differences in effects of study condition across these populations. This will be done with the addition of the effects of population and population-by-condition terms to the models described above. Study staff will make every effort to minimize missing data, and results of our ongoing trial suggest that this

will be minimal. We will examine the missing data pattern, and if it is missing at random, will use all data available, without imputation.

9.2. Sample Size

Sample size was determined using NQuery Advisor based on hypothesis tests related to Aim 1 to detect a significant difference between conditions on total CPD. The primary statistical approach will be repeated measures ANOVAs but required sample sizes were calculated focusing on expected outcomes at Week 16. This estimate is intentionally conservative and calculated based on one outcome at one time point; however, given the repeated measures nature of our data, we will have correlated observations within subjects and thus will achieve the stated power to detect differences of even lesser magnitude. Estimates are based on preliminary results from our current trial and from the Donny et al.[41] study of VLNCCs and NNCCs. The between-group effect size is defined as the difference of study condition means divided by the common standard deviation. A sample size of 75 participants per condition will provide 80% power to detect an effect size of 0.50 for all pair-wise comparisons, with a two-sided type 1 error rate of 0.05. While this is larger than the effect size in our ongoing trial, we assume that the addition of e-cigs to the VLNCCs will decrease CPD from that seen with VLNCCs alone. Thus, while we recognize that we may have insufficient power to detect differences between the VLNCC and NNCC alone conditions, we expect a larger effect size when comparing the NNCC and either of the VLNCC + e-cig conditions. This sample size also provides greater than 95% power to detect a linear dose-response effect across conditions. Because we have relied only on outcomes at Week 16, our proposed sample sizes are somewhat conservative, but we believe this is appropriate given that the effects of VLNCCs in this population, particularly in combination with e-cigs, are unknown. The sample size assumes a 15% loss to follow-up, consistent with our experience in the current study. We will increase our overall sample size to 310 in order to allow pilot testing with 10 participants.

Outcome	Observed between-group ES (15.8 vs. 0.4 mg/g)
CPD	0.38
Craving	0.74
FTND	0.14
Breath CO	0.06

10. Potential Risks of Participation

10.1 Risks of Participation

1) <u>Survey Questionnaires.</u> Surveys include questions about participants' medical and psychiatric histories, drug and alcohol use and history, breath tests for cigarette and alcohol use, urine or salivary tests of illicit drug use and pregnancy, and questionnaires about your mood. Answering these personal questions can make participants uncomfortable. If a participant reports thoughts of killing themselves or other indicators of suicidality, a study clinician will come to talk to the participant. The participant may also request to see a study clinician if he or she is in discomfort and would like help and/or referrals for mental health resources.
2) <u>Breach of Confidentiality</u>: The risk of the interview is loss of privacy if other people find out the results.
3) <u>Undue Influence</u>: Undue influence is a possible risk due to monetary compensation for participating in these studies. The likelihood of this risk is low because the compensation is commensurate with the amount of time and effort required for these studies.
4) <u>Drug Testing</u>: A breach of confidentiality could occur and other people could learn of the participant's drug use.
5) <u>Obtaining Blood Pressure and Heart Rate.</u> The blood pressure cuff may cause minimal discomfort. In obtaining blood pressure we may find a participant to have abnormal blood pressure and/or heart rate. If participant's blood pressure is abnormal, we will inform the participant of this, and participant may be advised to see a doctor, and may also be

contacted by our study doctor. Also, smoking and nicotine can affect the cardiovascular system, which may result in changes in blood pressure and/or heart rate.

6) Smoking Cigarettes: All cigarettes are detrimental to a person's health and can lead to significant medical problems including:

   a. Cardiovascular Diseases: Coronary heart disease, heart attack, stroke, peripheral vascular disease, reduced blood circulation, abdominal aortic aneurysm
   b. Respiratory Diseases: Emphysema, bronchitis, and chronic airway obstruction
   c. Cancers: Cancer of the lung, bladder, cervix, esophagus, kidney, larynx, mouth, pancreas, throat, and stomach; leukemia
   d. Metabolic Diseases: Type 2 Diabetes
   e. Other Health Risks Associated with Smoking: Including but not limited to infertility, lower bone density in postmenopausal women, and hip fracture in women
   f. Death

7) Smoking Study Cigarettes. All cigarettes are harmful to a person's health and can lead to cardiovascular (heart) disease, respiratory (lung) disease, cancer and other health problems. In addition to the above medical problems, participants may experience some minor negative health effects such as headaches. Participants may also experience smoking withdrawal symptoms, which are listed below. In addition, due to the altered nicotine levels, there could be a change in a participant's use of cigarettes including the manner in which he or she inhales the smoke. Smoking the study cigarette does not necessarily provide any less risk than the participant's usual brand of cigarette and could pose increased health risks.

8) Using Study E-cigarettes. E-cigarettes are devices that heat nicotine to produce an aerosol. The health effects of e-cigarettes are still unclear, but appear to be less than that for tobacco cigarettes. Most e-cigarette users have lower nicotine levels than when they smoked regular cigarettes. Some e-cigarette users, especially those who use both e-cigarettes and regular tobacco cigarettes as well as youth and young adults, can have increased nicotine levels. In some rare cases, these use patterns have been associated with seizures. Whether this would occur with the concurrent use of very low nicotine cigarettes is unclear. E-cigarettes users very often maintain addiction to nicotine, but this addiction appears to be somewhat less than that from tobacco cigarettes. Abruptly quitting e-cigarettes could cause withdrawal symptoms similar to those from quitting tobacco cigarettes (see below) but slightly less severe. The most common side effects include dry mouth, irritation of the throat and mouth, and mild cough. The JUUL and Vuse e-cigarettes we will be providing have not been well-studied but appear to be of similar risk to other e-cigarettes. Participants may have heard that e-cigarettes, or "vapes," can explode and seriously injure people. Study staff should instruct participants that although they appear rare, these explosions are dangerous. The exact causes of these incidents are not yet clear, but some evidence suggests that battery-related issues may lead to vape explosions. In order to prevent e-cigarette related injuries, instruct participants to keep their e-cig away from other metal objects, never charge the e-cig with a phone or tablet charger, don't charge the e-cig overnight or leave it charging unattended, and to stop using the e-cig if the batteries get damaged or wet. The participant will be instructed to always keep e-cig liquid out of kids' and pets' reach and sight after use. If the study staff learns about additional risks of e-cigarettes during the study, participants will be informed of these risks.

9) Mood and Psychiatric Symptom Changes. Participants may experience smoking withdrawal symptoms during this study. These symptoms can include anger, anxiousness, craving for a cigarette, depressed mood, difficulty concentrating, frustration, increased appetite, impatience/impulsivity, irritability, restlessness, sleep problems, and weight gain. These feelings can be uncomfortable and can last a couple of weeks, but usually are of minimal risk. In addition, if participants have a past history of anxiety, depression, or alcoholism, it is possible withdrawal could cause substantial increases in depression and anxiety

symptoms, but this appears to be rare. At each visit, study staff will ask participants how they feel. If either study staff or the participant thinks that being in this study is putting the participant's mental health at risk, staff may have the participant meet with an on-site clinician and/or stop participating in the study.

Further, if a participant report thoughts of killing oneself or other indicators of suicidality, a study clinician will come to talk to the participant. Participants may also request to see a study clinician if he or she is in discomfort and would like help and/or referrals for mental health resources.

10) Returning to Regular Smoking: It is possible that if participants return to smoking their usual brand of cigarette at the end of the study they may experience mild and transient nausea, dizziness, and lightheadedness.

11) Risk to Fetus. To avoid risks to a fetus, it is important that participants are not pregnant during this study. Avoiding sexual activity is the only certain method to prevent pregnancy. However, if participants choose to be sexually active, participants should be using approved forms of birth control if applicable determined by the Project Medical Director, including but not limited to prescribed birth control pills, patch, ring, injections, implants or intrauterine device (IUD) or an appropriate "double barrier" method. If you choose to be sexually active during this study, pregnancy could still result even with the use of these birth control methods.

10.2 Avoiding Risks to Fetus:

If participants choose to be sexually active, they should use an appropriate "double barrier" method of birth control (such as female use of a diaphragm, or contraceptive sponge, in addition to male use of a condom) or the female should be using prescribed "birth control" pills, patch, ring, injections, implants or intrauterine device (IUD) if applicable determined by the Project Medical Director. If a participant endorses a "double barrier" method, our medical professional will speak to the participant to confirm which methods will be used during the duration of the study. Participants will be tested for pregnancy every two weeks beginning at screening through the last study visit. If a participant becomes pregnant during the study, she will be withdrawn from the study. Approximately 30 days after being withdrawn or having a positive pregnancy test at the last study visit, the research staff will call the participant to confirm her due date. The licensed medical professional will follow-up with the participant after delivery to ask questions about the baby's health.

10.3 Expected benefits of participation:

There are no benefits from participating in the study. The information obtained from this study may ultimately help the Food and Drug Administration decide how best to regulate nicotine and tobacco products with the goal of improving public health.

11. Protection Against Risk

11.1 Data Collection Protections

Research data without identifiers will be maintained in a locked file cabinet and on password-protected computers in the research staff workplace, with only code numbers identifying subjects. Study consent forms and the linkage between the participants' names and codes will be stored in a locked file cabinet inside a locked office. Interviews with participants will be conducted in private rooms. Urine or saliva samples for drug and pregnancy tests and tobacco exposure biomarkers will be obtained in a private bathroom within the laboratory suite. Subjective measures will be administered electronically. The biostatistics and data-management team will provide consistent data-management practices for all data in the Center. Using REDCap, which is housed on the University of Vermont Medical Center's HIPAA-compliant computing system, will maximize validity and reliability of data. REDCap is a secure, web-based system that accommodates local and remote data collection by each project team, and allows for data entry work-flow monitoring and data quality control monitoring by biometry staff. The RedCap database for this project will be

hosted on the UVMCOM servers. In addition, data will be collected from participants on a daily basis using an interactive voice recognition system (IVR) developed and hosted by TeleSage Inc (www.telesage.com, Chapel Hill, NC). TeleSage is a company with expertise on gathering patient-centered outcomes tracking data for mental health clinical and research institutions. TeleSage has developed and hosted behavioral health-related research software systems using IVR and Web-based technologies and is leader in behavioral health outcomes tracking technologies. For data integrity, data entry windows will follow the structure of paper forms as much as possible to allow for ease of entry, and will use predefined choices to minimize errors when possible. Data quality monitoring will be facilitated with periodic down loads and analysis using a variety of common statistical program format such as SAS, Stata, R, and SPSS. Quality control procedures will be conducted for all data collected, including analysis of missing data and logic checks for out of range and other anomalous values. This secure electronic data gathering and transmission plan, overseen by the experienced biostatistical team, will minimize opportunities for breaches of confidentiality. Biological samples for nicotine and carcinogen biomarker analysis will be marked with participant ID, stored in the locked laboratory suite, and sent to a laboratory for analysis on a quarterly basis.

All information collected as part of this study will be accessible only to research staff. No information will be shared with participants' clinicians unless the participant requests this in writing. All investigators and staff have undergone (and any new staff will undergo) human subjects' ethics training as required by UVM and are fully conversant with relevant ethical principals around confidentiality. Assessments, consenting and study procedures will be closely supervised by the PI.

The sponsors (NIDA/FDA) as well as the Institutional Review Board and regulatory authorities could be granted direct access to original medical and research records for verification of clinical trial procedures and/or data. If this is required, it will be done under conditions that will protect privacy to the fullest extent possible consistent with laws relating to public disclosure of information and the law-enforcement responsibilities of the agency.

11.2 Data Storage:

Data will be stored locally at each site. Long-term storage of all study data, for at least 7 years after study completion, will be at the University of Vermont.

12. Adverse Events

The research assistant will ask about adverse events at each session, using a form that assesses the nature, severity, duration, action taken, and outcome of study-related adverse events. AEs will be captured from the time of first study cigarette. Participants will be given contact cards to inform us of events that occur between study contacts. Any AE that remains open will be reviewed and closed at an interview conducted 30 days after the study completion date (completers) or when the study should have ended had the participant completed the study (dropouts and those withdrawn by investigator).

All procedures will be monitored to ensure that they conform to the approved protocol. In addition, monitoring will be done of all unforeseen circumstances that might arise and affect safety; of all reports of serious adverse events as defined in 38 CFR 46 (death, new or prolonged hospitalization, persistent or significant disability/incapacity); of other significant adverse events (adverse events that lead to drop out by the participant or termination by the investigator); of unexpected adverse events resulting from the study, and of expected adverse events.

Any SAE will be brought to the attention of the site PIs as soon as possible and not longer than 24 hours. Any AE or SAE that is both unexpected and related to study participation will be reported to the IRB within 7 days of the event. The local IRB will make a determination as to whether additional reporting requirements are needed. IRB actions will be reported to the funding agency by the PIs no less than annually and more frequently as recommended by the local IRB. Any SAEs will be summarized in the yearly Progress Reports to the funding agency, including a review of frequency and severity. All SAEs will be followed through ongoing consultation with the physician caring for the patient until they resolve, result in death, or stabilize and are not expected to

improve. The study staff will be in close contact with participants and health care providers throughout the study to monitor for potential unanticipated problems. Any unanticipated problems will be discussed at the weekly research staff meetings and reported as required to the local IRB.

13. Withdrawal or Monitoring of Participants

For the participant's protection, participants will be withdrawn immediately from the study if any of the following occur:

1) Cardiovascular disease (CVD) event: Typically includes MI (heart attack), PTCA (angioplasty/stenting), bypass surgery, stroke, peripheral vascular disease (arterial blockages in arms or legs leading to procedure or surgery). Less common CVD problems would be new cardiac arrhythmias (e.g., new atrial fibrillation) or new valvular disease (e.g., mitral or aortic regurgitation).
2) DVT/PE (deep vein thrombosis/pulmonary embolism, i.e., blood clots in the venous system).
3) Suicide Attempt: A participant will be withdrawn if he/she attempts suicide at any time during participation in the study.
4) Psychiatric Hospitalization: A participant will be withdrawn if he/she is hospitalized for psychiatric reasons at any time during participation in the study.
5) Pregnancy: If participant indicates she is pregnant or has a positive pregnancy test at any time during the study, she will be withdrawn from the study, and this event will remain open until delivery. At that time the licensed medical professional will contact the participant to ask a few questions about the baby's health and will update the open 'Medical Event Form'. A positive pregnancy test at Week 16 will trigger a 'Medical Event Form' to be completed but will not result in withdrawal since she is no longer receiving study product.
6) Expired breath carbon monoxide increase: A participant will be withdrawn from the study if the average of two consecutive CO readings during the same visit is 100 ppm or greater.
7) Marked increase in smoking: A participant will be withdrawn from the study if he/she meets **BOTH** of the following criteria for two consecutive weeks
    a. Cigarette per day increase: The average CPD increases by more than 100% from the average CPD during baseline.
    b. Expired breath carbon monoxide increase: If the average of two consecutive CO measurements in the same visit is
        i. CO is greater than 50 ppm if CO at Baseline 1 is <20 ppm.
        ii. CO is greater than 60 ppm if CO at Baseline 1 is 20 – 34 ppm.
        iii. CO is greater than 70 ppm if CO at Baseline 1 is 35 – 49 ppm.
        iv. CO is greater than 80 ppm if CO at Baseline 1 is 50 – 64 ppm.
        v. CO is greater than 90 ppm if CO at Baseline 1 is 65 – 80 ppm.
    c. Note: If the second consecutive visit is the last study visit, then the participant would not be withdrawn from the study.
8) If a participant is discharged from or discontinues his or her methadone or buprenorphine treatment, they will be discontinued from the study.

The following will be monitored and can lead to the participant being withdrawn by the PI or Licensed Medical Professional:

1) Cigarettes per day increase: Continued participation will be evaluated by the site PI if the average number of cigarettes per day (CPD) increases by more than 100% from the average CPD during baseline as determined by CPD at Baseline 2.
2) Blood pressure (BP) or heart rate (HR) changes: If any of the following occur post-enrollment: 1) BP is at or above 160/100 or below 90/50, or 2) HR is at or above 115 bpm or below 45 bpm a manual blood pressure and heart rate measurement will be taken after 10

minutes have passed. If the manual reading is still out of range, a ‘Blood Pressure and Heart Rate Symptom Checklist’ and ‘Medical Event Form’ will be completed, and the participant will be monitored by the medical professional.

3) Expired breath Carbon Monoxide increase: If the average of two consecutive CO measurements meets the criteria below then the ‘Medical Event Form’ will be completed and the participant will be monitored by the medical professional.
    a. CO is greater than 50 ppm if CO at Baseline 1 is <20 ppm.
    b. CO is greater than 60 ppm if CO at Baseline 1 is 20 – 34 ppm.
    c. CO is greater than 70 ppm if CO at Baseline 1 is 35 – 49 ppm.
    d. CO is greater than 80 ppm if CO at Baseline 1 is 50 – 64 ppm.
    e. CO is greater than 90 ppm if CO at Baseline 1 is 65 – 80 ppm.
4) Any hospitalization or debilitation in which participation in the study could be detrimental to the recovery process. This will be self-reported by the participant and will be reviewed by the site PI and medical professional to determine whether continued participation in the study is appropriate.
5) If a participant is behaving in an inappropriate or threatening manner, admits to lying about eligibility criteria, is participating in other smoking research studies that could affect the primary outcome measures, etc., then the PI can withdraw him/her from the study at the PI’s discretion.
6) If a participant fails to attend regularly scheduled research assessment visits or comply with the research procedures or schedule, then the PI can withdraw him/her from the study at the PI’s discretion.
7) Increase in psychiatric symptoms: Exacerbation in symptoms noted during the study (i.e., change in BDI category from mild to moderate or moderate to severe) will trigger review by the study’s licensed medical professional. The PI will withdraw the participant upon the licensed medical professional’s recommendation.

14. Data Safety Monitoring Board

A Data and Safety Monitoring Board (DSMB) has been established to monitor safety outcomes and will be comprised of four members. The DSMB will be chaired by Kevin Delucchi, PhD., Professor in Residence of Biostatistics in Psychiatry at the University of California San Francisco and Director of the Quantitative Core of the San Francisco Treatment Research Center; Eden Evins, MD, MPH., Cox Family Professor of Psychiatry at Harvard Medical School and Director of Center for Addiction Medicine at Massachusetts General Hospital; Ari Kirshenbaum, PhD, Professor of Psychology at Saint Michael's College who he teaches courses in psychopharmacology and neuroscience, and currently has grants from NIH and NSF for work in human behavioral pharmacology and his grant-funded work focuses on cognitive and behavioral responses to nicotine and cannabinoids; and Elisabeth Johnson, Ph.D., who has over twenty years of clinical experience in women’s health and pediatrics, including caring for women with substance use disorders.

Conflict of Interest

None of the board members will be otherwise affiliated with the center and each member will complete a conflict of interest disclosure form prior to each meeting. Ad hoc specialists may be invited to participate as non-voting members at any time if additional expertise is desired.

Monitoring Activities and Frequency of Meetings

The DSMB will set their own agenda and decisions about monitoring; e.g. how frequently to monitor, what threshold requires changes to protocol or stopping the study, and whether to view raw or analyzed data. The DSMB will be given FDA and EMEA guidelines for DSMBs and recent reviews on DSMBs. A brief report will be generated from each meeting for the study record and forwarded to each of the study site’s Institutional Review Boards (IRB) and NIDA’s Program Officer with the progress report. The DSMB will be available to convene outside of the regular meetings, if necessary. If concerns should arise regarding a particular subject, or any troublesome trends in the

experiences of participants, they will make appropriate recommendations for changes in protocol, as needed. The project investigators will continue to examine safety data, blind to study condition, in case they wish to make study modifications. Before modifications are made, they will inform the DSMB and request their comments.

Communication Plan to IRB, NIDA, and FDA (if applicable)

All IRBs, the FDA and the NIDA's Program Officer will be informed of any significant action taken as a result of the Data and Monitoring Board's findings. Study Participants will be informed of any changes in risk.

Protection of Confidentiality

For DSMB meetings only de-identified data, including blinded study site and condition type, will be provided to the board. All data and discussion during the meeting will be confidential.

15. Investigational Tobacco Product

The University of Vermont Center on Tobacco Regulatory Science will complete an Investigational Tobacco Product (ITP) application with the FDA to cover the experimental cigarettes being used in this study. This application encompasses both Project 2 sites.

16. Certificate of Confidentiality

To help protect the participant's privacy, Dr. Stephen Higgins, PhD, will obtain a Certificate of Confidentiality from the national Institute on Drug Abuse. With this certificate, the researchers cannot be forced to disclose information that may identify the participants, even by a court subpoena, in any federal, state, or local civil, criminal, administrative, legislative, or other proceedings. The researchers will use the Certificate to resist any demands for information that would identify the participants, except as explained below. The Certificate cannot be used to resist a demand for information from personnel of the United States Government that is used for auditing or evaluation of federally funded projects or for information that must be disclosed in order to meet the requirements of the Federal Food and Drug Administration (FDA).

The Certificate of Confidentiality does not prevent the participant or a member of their family from voluntarily releasing information about themselves and their involvement in the research. If an insurer, employer or other person obtains the participant's written consent to receive research information, then the researcher may not use the Certificate to withhold that information.

The Certificate of Confidentiality does not prevent the researchers from disclosing voluntarily, without consent, information that would identify the individual as a participant of the research project in instances such as evidence of child abuse or a participant's threatened violence to self or others.

17. Outcome Variables

Primary Endpoints for Study 3:

1) Cigarette Smoked per Day (CPD)
2) Nicotine Dependence Severity

Secondary Endpoints for Study 3:

1) Measures of adherence: non-study cigarette use, drop-out rate
2) Measures of psychiatric symptoms: BDI, OASIS
3) Measures of discomfort/dysfunction: MNWS, QSU
4) Measures of other health-related behaviors: breath alcohol, urine or salivary drug screen, TLFB-drug use, Alcohol Use Questionnaire, Drug Use Questionnaire, weight
5) Measures of nicotine/tobacco dependence: FTND, WISDM
6) Measures of tobacco exposure: CO, total nicotine equivalents, NNN, NNAL, minor

alkaloids

7) Measures of intention to quit: Stages of Change, Contemplation Ladder
8) Measures of compensatory smoking: puff topography, filter analysis
9) Measures of other tobacco use: TLFB-other tobacco
10) Measures of cigarette characteristics: CES, Cigarette Purchase Task
11) Measures of cardiovascular function: heart rate, blood pressure, urine 11-dehydroTXB2
12) Measures of perceived risk: Perceived Health Risk Questionnaire
13) Safety outcome variables: Adverse Events (AEs), Serious Adverse Events (SAEs)

18. References

1. U.S. Department of Health and Human Services. *The Health Consequences of Smoking—50 Years of Progress: A Report of the Surgeon General*. Centers for Disease Control and Prevention, National Center for Chronic Disease Prevention and Health Promotion (US) Office on Smoking and Health, Atlanta GA; 2014.
2. Lasser K, Boyd JW, Woolhandler S, Himmelstein DU, McCormick D, Bor DH. Smoking and mental illness: A population-based prevalence study. *JAMA*. 2000;284(20):2606-2610.
3. Lawrence D, Mitrou F, Zubrick SR. Smoking and mental illness: results from population surveys in Australia and the United States. *BMC Public Health*. 2009;9:285. doi:10.1186/1471-2458-9-285.
4. Schroeder SA. American health improvement depends upon addressing class disparities. Prev Med. 2016; 92:6-15. Doi: 10.1016/jypmed.2016.02.024.
5. Villanti AC, Johnson AL, Ambrose BK, et al. Flavored Tobacco Product Use in Youth and Adults: Findings From the First Wave of the PATH Study (2013-2014). *Am J Prev Med*. March 2017. doi:10.1016/j.amepre.2017.01.026.
6. Conway KP, Green VR, Kasza KA, et al. Co-occurrence of tobacco product use, substance use, and mental health problems among adults: Findings from Wave 1 (2013-2014) of the Population Assessment of Tobacco and Health (PATH) Study. *Drug Alcohol Depend*. 2017;177:104-111. doi:10.1016/j.drugalcdep.2017.03.032.
7. Engström A, Adamsson C, Allebeck P, Rydberg U. Mortality in patients with substance abuse: a follow-up in Stockholm County, 1973-1984. *Int J Addict*. 1991;26(1):91-106.
8. Grinshpoon A, Barchana M, Lipshitz I, Rosca P, Weizman A, Ponizovsky AM. Methadone maintenance and cancer risk: an Israeli case registry study. *Drug Alcohol Depend*. 2011;119(1-2):88-92. doi:10.1016/j.drugalcdep.2011.05.028.
9. Hser YI, McCarthy WJ, Anglin MD. Tobacco use as a distal predictor of mortality among long-term narcotics addicts. *Prev Med*. 1994;23(1):61-69. doi:10.1006/pmed.1994.1009.
10. Hurt RD, Offord KP, Croghan IT, et al. Mortality following inpatient addictions treatment. Role of tobacco use in a community-based cohort. *JAMA*. 1996;275(14):1097-1103.
11. Jamal A, King BA, Neff LJ, Whitmill J, Babb SD, Graffunder CM. Current cigarette smoking among adults - United States, 2005-2015. *MMWR Morb Mortal Wkly Rep*. 2016;65(44):1205-1211. doi:10.15585/mmwr.mm6544a2.
12. Clemmey P, Brooner R, Chutuape MA, Kidorf M, Stitzer M. Smoking habits and attitudes in a methadone maintenance treatment population. *Drug Alcohol Depend*. 1997;44(2-3):123-132.
13. Guydish J, Passalacqua E, Tajima B, Chan M, Chun J, Bostrom A. Smoking prevalence in addiction treatment: a review. *Nicotine Tob Res*. 2011;13(6):401-411. doi:10.1093/ntr/ntr048.
14. Haas AL, Sorensen JL, Hall SM, et al. Cigarette smoking in opioid-using patients presenting for hospital-based medical services. *Am J Addict*. 2008;17(1):65-69. doi:10.1080/10550490701756112.
15. Mutschler NH, Stephen BJ, Teoh SK, Mendelson JH, Mello NK. An inpatient study of the effects of buprenorphine on cigarette smoking in men concurrently dependent on cocaine and opioids. *Nicotine Tob Res*. 2002;4(2):223-228. doi:10.1080/14622200210124012.
16. Nahvi S, Richter K, Li X, Modali L, Arnsten J. Cigarette smoking and interest in quitting in methadone maintenance patients. *Addict Behav*. 2006;31(11):2127-2134. doi:10.1016/j.addbeh.2006.01.006.
17. Richter KP, Gibson CA, Ahluwalia JS, Schmelzle KH. Tobacco use and quit attempts among methadone maintenance clients. *Am J Public Health*. 2001;91(2):296-299.

18. Parker M, Streck JM, Sigmon SC. Prevalence and severity of nicotine dependence in opioid-dependent vs. general population using national data. In preparation.

19. Clarke JG, Stein MD, McGarry KA, Gogineni A. Interest in smoking cessation among injection drug users. *Am J Addict*. 2001;10(2):159-166.

20. Dunn KE, Sigmon SC, Reimann E, Heil SH, Higgins ST. Effects of smoking cessation on illicit drug use among opioid maintenance patients: A pilot study. *J Drug Issues*. 2009;39(2):313-328.

21. Frosch DL, Shoptaw S, Jarvik ME, Rawson RA, Ling W. Interest in smoking cessation among methadone maintained outpatients. *J Addict Dis*. 1998;17(2):9-19. doi:10.1300/J069v17n02_02.

22. Kozlowski LT, Skinner W, Kent C, Pope MA. Prospects for smoking treatment in individuals seeking treatment for alcohol and other drug problems. *Addict Behav*. 1989;14(3):273-278.

23. Sees KL, Clark HW. When to begin smoking cessation in substance abusers. *J Subst Abuse Treat*. 1993;10(2):189-195.

24. Nahvi S, Ning Y, Segal KS, Richter KP, Arnsten JH. Varenicline efficacy and safety among methadone maintained smokers: a randomized placebo-controlled trial. *Addiction*. 2014;109(9):1554-1563. doi:10.1111/add.12631.

25. Okoli CTC, Khara M, Procyshyn RM, Johnson JL, Barr AM, Greaves L. Smoking cessation interventions among individuals in methadone maintenance: a brief review. *J Subst Abuse Treat*. 2010;38(2):191-199. doi:10.1016/j.jsat.2009.10.001.

26. Poling J, Rounsaville B, Gonsai K, Severino K, Sofuoglu M. The safety and efficacy of varenicline in cocaine using smokers maintained on methadone: a pilot study. *Am J Addict*. 2010;19(5):401-408. doi:10.1111/j.1521-0391.2010.00066.x.

27. Reid MS, Fallon B, Sonne S, et al. Smoking cessation treatment in community- based substance abuse rehabilitation programs. *J Subst Abuse Treat*. 2008;35(1):68-77. doi:10.1016/j.jsat.2007.08.010.

28. Richter KP, McCool RM, Catley D, Hall M, Ahluwalia JS. Dual pharmacotherapy and motivational interviewing for tobacco dependence among drug treatment patients. *J Addict Dis*. 2005;24(4):79-90.

29. Miller ME, Sigmon SC. Are Pharmacotherapies Ineffective in Opioid-Dependent Smokers? Reflections on the Scientific Literature and Future Directions. *Nicotine Tob Res*. 2015;17(8):955-959. doi:10.1093/ntr/ntv030.

30. Substance Abuse and Mental Health Services Administration (SAMHSA). *Results from the 2012 National Survey on Drug Use and Health: Summary of National Findings*, NSDUH Series H-46, HHS Publication No. (SMA) 13-4795. Rockville, MD: Substance Abuse and Mental Health Services Administration; 2013.

31. Substance Abuse and Mental Health Services Administration (SAMHSA). National Survey of Substance Abuse Treatment Services (N-SSATS): 2012. Data on Substance Abuse Treatment Facilities. BHSIS Series S-66, HHS Publication No. (SMA) 14-4809. Rockville, MD: Substance Abuse and Mental Health Services Administration; 2013.

32. Benowitz NL, Henningfield JE. Establishing a nicotine threshold for addiction. The implications for tobacco regulation. *N Engl J Med*. 1994;331(2):123-125. doi:10.1056/NEJM199407143310212.

33. Benowitz NL, Henningfield JE. Reducing the nicotine content to make cigarettes less addictive. *Tob Control*. 2013;22 Suppl 1:i14-i17. doi:10.1136/tobaccocontrol- 2012-050860.\

34. Donny EC, Hatsukami DK, Benowitz NL, Sved AF, Tidey JW, Cassidy RN. Reduced nicotine product standards for combustible tobacco: building an empirical basis for effective regulation.

Prev Med. 2014;68:17-22. doi:10.1016/j.ypmed.2014.06.020.

35. Fagan P, King G, Lawrence D, et al. Eliminating tobacco-related health disparities: directions for future research. *Am J Public Health*. 2004;94(2):211-217.

36. Becker KM, Rose JE, Albino AP. A randomized trial of nicotine replacement therapy in combination with reduced-nicotine cigarettes for smoking cessation. *Nicotine Tob Res*. 2008;10(7):1139-1148. doi:10.1080/14622200802123294.

37. Benowitz NL, Hall SM, Stewart S, Wilson M, Dempsey D, Jacob P. Nicotine and carcinogen exposure with smoking of progressively reduced nicotine content cigarette. *Cancer Epidemiol Biomarkers Prev*. 2007;16(11):2479-2485. doi:10.1158/1055-9965.EPI-07-0393.

38. Benowitz NL, Dains KM, Hall SM, et al. Smoking behavior and exposure to tobacco toxicants during 6 months of smoking progressively reduced nicotine content cigarettes. *Cancer Epidemiol Biomarkers Prev*. 2012;21(5):761-769. doi:10.1158/1055-9965.EPI-11-0644.

39. Donny EC, Jones M. Prolonged exposure to denicotinized cigarettes with or without transdermal nicotine. *Drug Alcohol Depend*. 2009;104(1-2):23-33. doi:10.1016/j.drugalcdep.2009.01.021.

40. Donny EC, Houtsmuller E, Stitzer ML. Smoking in the absence of nicotine: behavioral, subjective and physiological effects over 11 days. *Addiction*. 2007;102(2):324-334. doi:10.1111/j.1360-0443.2006.01670.x.

41. Donny EC, Denlinger RL, Tidey JW, et al. Randomized trial of reduced-nicotine standards for cigarettes. *N Engl J Med*. 2015;373(14):1340-1349. doi:10.1056/NEJMsa1502403.

42. Hatsukami DK, Kotlyar M, Hertsgaard LA, et al. Reduced nicotine content cigarettes: effects on toxicant exposure, dependence and cessation. *Addiction*. 2010;105(2):343-355. doi:10.1111/j.1360-0443.2009.02780.x.

43. Hatsukami DK, Perkins KA, Lesage MG, et al. Nicotine reduction revisited: science and future directions. *Tob Control*. 2010;19(5):e1-e10. doi:10.1136/tc.2009.035584.

44. Hatsukami DK, Luo X, Dick L, et al. Reduced nicotine content cigarettes and use of alternative nicotine products: exploratory trial. *Addiction*. 2017;112(1):156-167. doi:10.1111/add.13603.

45. Walker N, Howe C, Bullen C, et al. The combined effect of very low nicotine content cigarettes, used as an adjunct to usual Quitline care (nicotine replacement therapy and behavioural support), on smoking cessation: a randomized controlled trial. *Addiction*. 2012;107(10):1857-1867. doi:10.1111/j.1360-0443.2012.03906.x.

46. Zeller M, Hatsukami D, Strategic dialogue on tobacco harm reduction group. The strategic dialogue on tobacco harm reduction: a vision and blueprint for action in the US. *Tob Control*. 2009;18(4):324-332. doi:10.1136/tc.2008.027318.

47. Dani JA, Harris RA. Nicotine addiction and comorbidity with alcohol abuse and mental illness. *Nat Neurosci*. 2005;8(11):1465-1470. doi:10.1038/nn1580.

48. Benowitz, N. Compensatory smoking of low-yield cigarettes. In: Shopland, Burns, Benowitz, Amacher, editors. NCI smoking and tobacco control monograph 13: Risks associated with smoking cigarettes with low machine-measured yields of tar and nicotine. Bethesda, MD: US Dept of Health and Human Services, Public Health Service, National Institutes of Health, National Cancer Institute; 2001. p 39-64.

49. Kozlowski LT, O'Connor RJ, Sweeney CT. Cigarette design. In: Shopland, Burns, Benowitz, Amacher, editors. NCI smoking and tobacco control monograph 13: Risks associated with smoking cigarettes with low machine-measured yields of tar and nicotine. Bethesda, MD: US Dept of Health and Human Services, Public Health Service, National Institutes of Health, National Cancer Institute; 2001. p 13-37.

50. Higgins ST, Heil SH, Sigmon SC, et al. Addiction potential of reduced nicotine content

cigarettes in populations with psychiatric disorders and other vulnerabilities to tobacco addiction. *JAMA Psychiatry*. In press.

51. Donny EC, Walker N, Hatsukami D, Bullen C. Reducing the nicotine content of combusted tobacco products sold in New Zealand. *Tob Control*. 2017;26(e1):e37- e42. doi:10.1136/tobaccocontrol-2016-053186.

52. Benowitz NL, Donny EC, Hatsukami DK. Reduced nicotine content cigarettes, e- cigarettes and the cigarette end game. *Addiction*. 2017;112(1):6-7. doi:10.1111/add.13534.

53. Quisenberry AJ, Koffarnus MN, Hatz LE, Epstein LH, Bickel WK. The Experimental Tobacco Marketplace I: Substitutability as a function of the price of conventional cigarettes. *Nicotine Tob Res*. 2016;18(7):1642-1648. doi:10.1093/ntr/ntv230.

54. Dawkins L, Kimber C, Puwanesarasa Y, Soar K. First- versus second-generation electronic cigarettes: predictors of choice and effects on urge to smoke and withdrawal symptoms. *Addiction*. 2015;110(4):669-677. doi:10.1111/add.12807.

55. Farsalinos KE, Spyrou A, Tsimopoulou K, Stefopoulos C, Romagna G, Voudris V. Nicotine absorption from electronic cigarette use: comparison between first and new- generation devices. *Sci Rep*. 2014;4:4133. doi:10.1038/srep04133.

56. Goniewicz ML, Kuma T, Gawron M, Knysak J, Kosmider L. Nicotine levels in electronic cigarettes. *Nicotine Tob Res*. 2013;15(1):158-166. doi:10.1093/ntr/nts103.

57. Talih S, Balhas Z, Eissenberg T, et al. Effects of user puff topography, device voltage, and liquid nicotine concentration on electronic cigarette nicotine yield: measurements and model predictions. *Nicotine Tob Res*. 2015;17(2):150-157. doi:10.1093/ntr/ntu174.

58. Trtchounian A, Williams M, Talbot P. Conventional and electronic cigarettes (e- cigarettes) have different smoking characteristics. *Nicotine Tob Res*. 2010;12(9):905- 912. doi:10.1093/ntr/ntq114.

59. Hecht SS, Carmella SG, Kotandeniya D, et al. Evaluation of toxicant and carcinogen metabolites in the urine of e-cigarette users versus cigarette smokers. *Nicotine Tob Res*. 2015;17(6):704-709. doi:10.1093/ntr/ntu218.

60. Goniewicz ML, Gawron M, Smith DM, Peng M, Jacob P, Benowitz NL. Exposure to nicotine and selected toxicants in cigarette smokers who switched to electronic cigarettes: A longitudinal within-subjects observational study. *Nicotine Tob Res*. 2017;19(2):160-167. doi:10.1093/ntr/ntw160.

61. Hefner K, Rosenheck R, Merrel J, Coffman M, Valentine G, Sofuoglu M. E-cigarette use in veterans seeking mental health and/or substance use services. *J Dual Diagn*. 2016;12(2):109-117. doi:10.1080/15504263.2016.1172895.

62. Hefner K, Valentine G, Sofuoglu M. Electronic cigarettes and mental illness: Reviewing the evidence for help and harm among those with psychiatric and substance use disorders. *Am J Addict*. 2017;26(4):306-315. doi:10.1111/ajad.12504.

63. Peters EN, Harrell PT, Hendricks PS, O'Grady KE, Pickworth WB, Vocci FJ. Electronic cigarettes in adults in outpatient substance use treatment: Awareness, perceptions, use, and reasons for use. *Am J Addict*. 2015;24(3):233-239. doi:10.1111/ajad.12206.

64. Prochaska JJ, Grana RA. E-cigarette use among smokers with serious mental illness. *PLoS ONE*. 2014;9(11):e113013. doi:10.1371/journal.pone.0113013.

65. Stein MD, Caviness CM, Grimone K, Audet D, Borges A, Anderson BJ. E-cigarette knowledge, attitudes, and use in opioid dependent smokers. *J Subst Abuse Treat*. 2015;52:73-77. doi:10.1016/j.jsat.2014.11.002.

66. Bullen C, Howe C, Laugesen M, et al. Electronic cigarettes for smoking cessation: a randomised controlled trial. *Lancet*. 2013;382(9905):1629-1637. doi:10.1016/S0140-

6736(13)61842-5.

67. Caponnetto P, Auditore R, Russo C, Cappello GC, Polosa R. Impact of an electronic cigarette on smoking reduction and cessation in schizophrenic smokers: a prospective 12-month pilot study. *Int J Environ Res Public Health*. 2013;10(2):446-461. doi:10.3390/ijerph10020446.

68. Tseng T-Y, Ostroff JS, Campo A, et al. A randomized trial comparing the effect of nicotine versus placebo electronic cigarettes on smoking reduction among young adult smokers. *Nicotine Tob Res*. 2016;18(10):1937-1943. doi:10.1093/ntr/ntw017.

69. Adriaens K, Van Gucht D, Declerck P, Baeyens F. Effectiveness of the electronic cigarette: An eight-week Flemish study with six-month follow-up on smoking reduction, craving and experienced benefits and complaints. *Int J Environ Res Public Health*. 2014;11(11):11220-11248. doi:10.3390/ijerph111111220.

70. Stein MD, Caviness C, Grimone K, Audet D, Anderson BJ, Bailey GL. An open trial of electronic cigarettes for smoking cessation among methadone-maintained smokers. *Nicotine Tob Res*. 2016;18(5):1157-1162. doi:10.1093/ntr/ntv267.

71. Glasser AM, Collins L, Pearson JL, et al. Overview of electronic nicotine delivery systems: A systematic review. *Am J Prev Med*. 2017;52(2):e33-e66. doi:10.1016/j.amepre.2016.10.036.

72. Lechner WV, Meier E, Wiener JL, et al. The comparative efficacy of first- versus second-generation electronic cigarettes in reducing symptoms of nicotine withdrawal. *Addiction*. 2015;110(5):862-867. doi:10.1111/add.12870.

73. Vansickel AR, Eissenberg T. Electronic cigarettes: effective nicotine delivery after acute administration. *Nicotine Tob Res*. 2013;15(1):267-270. doi:10.1093/ntr/ntr316.

74. Bonhomme MG, Holder-Hayes E, Ambrose BK, et al. Flavoured non-cigarette tobacco product use among US adults: 2013-2014. *Tob Control*. 2016;25(Suppl 2):ii4-ii13. doi:10.1136/tobaccocontrol-2016-053373.

75. Audrain-McGovern J, Strasser AA, Wileyto EP. The impact of flavoring on the rewarding and reinforcing value of e-cigarettes with nicotine among young adult smokers. *Drug Alcohol Depend*. 2016;166:263-267. doi:10.1016/j.drugalcdep.2016.06.030.

76. Goldenson NI, Kirkpatrick MG, Barrington-Trimis JL, et al. Effects of sweet flavorings and nicotine on the appeal and sensory properties of e-cigarettes among young adult vapers: Application of a novel methodology. *Drug Alcohol Depend*. 2016;168:176-180. doi:10.1016/j.drugalcdep.2016.09.014.

77. Berg CJ. Preferred flavors and reasons for e-cigarette use and discontinued use among never, current, and former smokers. *Int J Public Health*. 2016;61(2):225-236. doi:10.1007/s00038-015-0764-x.

78. Kistler CE, Crutchfield TM, Sutfin EL, et al. Consumers' preferences for electronic nicotine delivery system product features: A structured content analysis. *Int J Environ Res Public Health*. 2017;14(6). doi:10.3390/ijerph14060613.

79. Czoli CD, Goniewicz M, Islam T, Kotnowski K, Hammond D. Consumer preferences for electronic cigarettes: results from a discrete choice experiment. *Tob Control*. 2016;25(e1):e30-e36. doi:10.1136/tobaccocontrol-2015-052422.

80. Pesko MF, Kenkel DS, Wang H, Hughes JM. The effect of potential electronic nicotine delivery system regulations on nicotine product selection. *Addiction*. 2016;111(4):734-744. doi:10.1111/add.13257.

81. Etter JF. Electronic cigarettes: a survey of users. *BMC Public Health*. 2010;10:231. doi:10.1186/1471-2458-10-231.

82. Farsalinos KE, Romagna G, Tsiapras D, Kyrzopoulos S, Spyrou A, Voudris V. Impact of flavour variability on electronic cigarette use experience: an internet survey. *Int J Environ Res*

Public Health. 2013;10(12):7272-7282. doi:10.3390/ijerph10127272.

83. Litt MD, Duffy V, Oncken C. Cigarette smoking and electronic cigarette vaping patterns as a function of e-cigarette flavourings. *Tob Control*. 2016;25(Suppl 2):ii67- ii72. doi:10.1136/tobaccocontrol-2016-053223.

84. Yingst JM, Veldheer S, Hrabovsky S, Nichols TT, Wilson SJ, Foulds J. Factors associated with electronic cigarette users' device preferences and transition from first generation to advanced generation devices. *Nicotine Tob Res*. 2015;17(10):1242- 1246. doi:10.1093/ntr/ntv052.

85. Bogucka-Bonikowska A, Baran-Furga H, Chmielewska K, et al. Taste function in methadone-maintained opioid-dependent men. *Drug Alcohol Depend*. 2002;68(1):113-117.

86. Fenn JM, Laurent JS, Sigmon SC. Increases in body mass index following initiation of methadone treatment. *J Subst Abuse Treat*. 2015;51:59-63. doi:10.1016/j.jsat.2014.10.007.

87. Mysels DJ, Sullivan MA. The relationship between opioid and sugar intake: review of evidence and clinical applications. *J Opioid Manag*. 2010;6(6):445-452.

88. Nolan LJ, Scagnelli LM. Preference for sweet foods and higher body mass index in patients being treated in long-term methadone maintenance. *Subst Use Misuse*. 2007;42(10):1555-1566. doi:10.1080/10826080701517727.

89. Olszewski PK, Levine AS. Central opioids and consumption of sweet tastants: when reward outweighs homeostasis. *Physiol Behav*. 2007;91(5):506-512. doi:10.1016/j.physbeh.2007.01.011.

90. Zador D, Lyons Wall PM, Webster I. High sugar intake in a group of women on methadone maintenance in south western Sydney, Australia. *Addiction*. 1996;91(7):1053-1061.

91. Chait LD, Griffiths RR. Effects of methadone on human cigarette smoking and subjective ratings. *J Pharmacol Exp Ther*. 1984;229(3):636-640.

92. Higgins ST, Budney AJ, Hughes JR, Bickel WK, Lynn M, Mortensen A. Influence of cocaine use on cigarette smoking. *JAMA*. 1994;272(22):1724.

93. Mello NK, Mendelson JH, Sellers ML, Kuehnle JC. Effects of heroin self- administration on cigarette smoking. *Psychopharmacology (Berl)*. 1980;67(1):45-52.

94. Mello NK, Lukas SE, Mendelson JH. Buprenorphine effects on cigarette smoking. *Psychopharmacology (Berl)*. 1985;86(4):417-425.

95. Roll JM, Higgins ST, Tidey J. Cocaine use can increase cigarette smoking: evidence from laboratory and naturalistic settings. *Exp Clin Psychopharmacol*. 1997;5(3):263-268.

96. Schmitz JM, Grabowski J, Rhoades H. The effects of high and low doses of methadone on cigarette smoking. *Drug Alcohol Depend*. 1994;34(3):237-242.

97. Dunn KE, Sigmon SC, Thomas CS, Heil SH, Higgins ST. Voucher-based contingent reinforcement of smoking abstinence among methadone-maintained patients: a pilot study. *J Appl Behav Anal*. 2008;41(4):527-538.

98. Dunn KE, Sigmon SC, Reimann EF, Badger GJ, Heil SH, Higgins ST. A contingency-management intervention to promote initial smoking cessation among opioid-maintained patients. *Exp Clin Psychopharmacol*. 2010;18(1):37-50. doi:10.1037/a0018649.

99. Sigmon SC, Miller ME, Meyer AC, et al. Financial incentives to promote extended smoking abstinence in opioid-maintained patients: a randomized trial. *Addiction*. 2016;111(5):903-912. doi:10.1111/add.13264.

100. Higgins ST, Heil SH, Sigmon SC, et al. Response to varying the nicotine content of cigarettes in vulnerable populations: an initial experimental examination of acute effects. *Psychopharmacology (Berl)*. 2017;234(1):89-98. doi:10.1007/s00213-016- 4438-z.

101. Beck AT, Steer RA, Brown GK. Manual for Beck Depression Inventory – II 1996; San Antonio, TX: Psychological Corporation.

102. Norman SB, Cissell SH, Means-Christensen AJ, Stein MB. Development and validation of an Overall Anxiety Severity And Impairment Scale (OASIS). Depress Anxiety. 2006;23(4):245-249.

103. Sheehan DV, Lecrubier Y, Sheehan KH, et al. The Mini-International Neuropsychiatric Interview (M.I.N.I.): the development and validation of a structured diagnostic psychiatric interview for DSM-IV and ICD-10. J Clin Psychiatry. 1998;59 Suppl 20:22-33.

104. Sheehan DV, Lecrubier Y, Sheehan K, Harnett, Janavs J, Weiller E, et al. (1997). The validity of the Mini International Neuropsychiatric Interview (MINI) according to the SCID-P and its reliability. *Eur Psychiat 12*, 232–241.

105. Babor TF, de la Fuente JR, Saunders J, Grant M. AUDIT: The Alcohol Use Disorders Identification Test.Guidelines for use in primary health care. Geneva : World Health Organization, 1992.

106. Skinner HA. The Drug Abuse Screening Test. Addict Behav. 1982; 7(4):363-371.

107. Heatherton TF, Kozlowski LT, Frecker RC, Fagerström KO. The Fagerström Test for Nicotine Dependence: a revision of the Fagerström Tolerance Questionnaire. Br J Addict. 1991;86(9):1119-1127.

108. Piper ME, McCarthy DE, Bolt DM, Smith SS, Lerman C, Benowitz N, et al. Assessing dimensions of nicotine dependence: An evaluation of the Nicotine Dependence Syndrome Scale (NDSS) and the Wisconsin Inventory of Smoking Dependence Motives (WISDM) Nicotine & Tobacco Research. 2008;10:1009–1020.

109. Foulds J, Veldheer S, Yingst J, Wilson SJ, Nichols TT, Eissenberg T. Development of a questionnaire for assessing dependence on electronic cigarettes among a large sample of ex-smoking E-cigarette users. Nicotine Tob Res. 2015; 17(2): 186-92.

110. DiClemente CC, Prochaska JO, Fairhurst SK, Velicer WF, Velasquez MM, Rossi JS. The process of smoking cessation: an analysis of precontemplation, contemplation, and preparation stages of change. J Consult Clin Psychol. 1991;59(2):295-304.

111. McLellan ST, Luborsky L, Cacciola J, Griffith JE, Evans F, Barr HL, O'Brien CP. New data from the Addiction Severity Index: Reliability and validity in three centers. Journal of Nervous and Mental Disease. 1985;173:412-423.

112. Cohen S, Kamarck T, Mermelstein R. A global measure of perceived stress. J Health Soc Behav. 1983;24(4):385-396.

113. Watson D, Clark LA, Tellegen A. Development and validation of brief measures of positive and negative affect: the PANAS scales. J Pers Soc Psychol. 1988;54(6):1063-1070.

114. Hughes JR, Hatsukami D. Signs and symptoms of tobacco withdrawal. Arch Gen Psychiatry. 1986;43(3):289-294.

115. Cox LS, Tiffany ST, Christen AG. Evaluation of the brief questionnaire of smoking urges (QSU-brief) in laboratory and clinical settings. Nicotine Tob Res. 2001;3(1):7- 16.

116. Dowd AN, Motschman CA, Tiffany ST. Development and Validation of the Questionnaire of Vaping Craving. Nicotine Tob Res. 2019;12(1):63-70.

117. Cappelleri JC, Bushmakin AG, Baker CL, Merikle E, Olufade AO, Gilbert DG. Confirmatory factor analyses and reliability of the modified cigarette evaluation questionnaire. Addict Behav. 2007;32(5):912-923.

118. Jacobs EA, Bickel WK. Modeling drug consumption in the clinic using simulation procedures: demand for heroin and cigarettes in opioid-dependent outpatients. Exp Clin Psychopharmacol.

1999;7(4):412-426.

119. Cassidy RN, Tidey JW, Colby SM, Long V, Higgins ST. Initial development of an e- cigarette purchase task: a mixed methods study. Tob Regul Sci. 2017; 3(2): 139-150.

120. Grace RC, Kivel BM, Laugesen M. Estimating cross-price elasticity of e-cigarettes using a simulated demand procedure. Nicotine Tob Res. 2015 May;17(5):592-598.

121. Snaith RP, Hamilton M, Morley S, Humayan A, Hargreaves D, Trigwell P. A scale for the assessment of hedonic tone the Snaith–Hamilton Pleasure Scale. British Journal of Psychiatry. 1995;167:99–103.

STUDY PROTOCOL: SMOKERS WITH SOCIOECONOMIC DISADVANTAGE (WOMEN OF REPRODUCTIVE AGE)

Table of Contents

1. OBJECTIVE 96
2. SIGNIFICANCE 97
   2.1 Special Health Risks of Smoking Among Women 97
   2.2 Empirical Evidence on Reduced Nicotine Content Cigarettes 98
   2.3 Relevance of the Project to the Integrative Theme and Goals of the TCORS 99
   2.4 Relevance to the Scientific Domains and Priorities of the FDA CTP 99
   2.5 How Study Outcomes Will Improve Scientific Knowledge Related to the Manufacture, Distribution, and Marketing of Tobacco Products 100
4. RATIONALE 100
   3.1 Use and Effects of E-Cigarettes 100
   3.2 E-Cigarette Prevalence 100
   3.3 E-Cig Effects on Smoking 100
   3.4 Products to be Tested 101
   3.5 Summary 102
4. PROJECT STUDY METHODS 102
5. STUDY SCREENING PROCEDURES 102
   5.1 Participants 102
   5.2 Recruitment 103
   5.3 Informed Consent Process 104
   5.4 Screening Measures 105
   5.5 Suicidality/Mental Health Monitoring 106
   5.6 Inclusion/Exclusion Criteria 107
   5.7 Eligibility Determination 109
6. STUDY BASELINE PROCEDURES 109
   6.1 Visit Scheduling Requirements for Baseline Period 111
   6.2 Measures/Assessments 111
   6.3 E-Cigarette Training Sessions 112
   6.4 Interactive Voice Response System 113
7. STUDY EXPERIMENTAL PROCEDURES 114
   7.1 Experimental Period 114
   7.2 Experimental Visits Weeks 1, 3, 5, 7, 9, 11, 13, and 15 Procedures 114
      7.2.A. Measures/Assessments 114
   7.3 Experimental Visits Weeks 2, 4, 6, 8, 10, 12, 14, and 16 Procedures 115
      7.3.A. Measures/Assessments 115

7.4 Interactive Voice Response System 116
7.5 Variable Incentive Program 116
7.6 Product and Procedures Compliance Review Sessions 117
7.7 Quit Attempts During the Study Protocol 117
7.7.A. If a participant is currently abstaining from smoking with the intention to quit 118
7.7.B. If a participant is planning to quit smoking, but has not initiated the quit attempt.. 118
7.8 Abstinence Assessment Session 118
7.8.A. Participants Who Meet Criteria for Abstinence 118
7.8.A.1. Measures/Assessments 118
7.8.B. Participants Who Do Not meet Criteria for Abstinence 119
7.8.B.1. Measures/Assessments 119
7.9 Participant Compensation 120
7.10 End of Study 120
7.11 30-day Follow Up Phone Call 120
8. STUDY RANDOMIZATION 121
8.1 Randomization Process 121
8.2 Study Product Administration 121
8.3 Guidelines for Reporting Other Nicotine Product Use 121
8.4 Study Product Accountability 122
9. STUDY 3 STATISTICAL METHODS AND SAMPLE SIZE 122
9.1 Statistical Methods 122
9.2 Sample Size 123
10. POTENTIAL RISKS OF PARTICIPATION 123
10.1 Risks of Participation 123
10.2 Avoiding Risk to Fetus 125
10.3 Expected Benefits of Participation 125
11. PROTECTION AGAINST RISK 125
11.1 Data Collection Protections 126
11.2 Data Storage 126
12. ADVERSE EVENTS 126
13. WITHDRAWAL OR MONITORING OF PARTICIPANTS 127
14. DATA SAFETY MONITORING BOARD 128
15. INVESTIGATIONAL TOBACCO PRODUCT 129
16. CERTIFICATE OF CONFIDENTIALITY 129
17. OUTCOME VARIABLES 130
18. REFERENCES 131

Abbreviations

- 3HC: 3-hydroxycotinine
- BAL: Breath alcohol levels
- BDI: Beck’s Depression Inventory
- BMI: Body Mass Index
- BP: Blood pressure
- BPM: Beats per minute
- BRIEF-A: Behavioral Rating Inventory of Executive Function
- CES: Cigarette Evaluation Scale
- CO: Carbon monoxide
- COT: Cotinine
- CPD: Cigarettes per day
- CPT: Cigarette Purchase Task
- CPT: Continuous Performance Task
- DAST: Drug Abuse Screening Test
- DDT: Delay Discounting Task
- D-KEFS: Delis-Kaplan Executive Function System
- EDC: Electronic Data Capture
- EQ-5D: Euro-Qol
- FSPTCA: Family Smoking Prevention and Tobacco Control Act
- FTND: Fagerström Test for Nicotine Dependence
- GAD: Generalized Anxiety Disorder
- HR: Heart rate
- IVR: Interactive Voice Response
- MDD: Major Depressive Disorder
- MINI: Mini International Neuropsychiatric Interview
- MNWS: Minnesota Nicotine Withdrawal Scale
- NMR: Nicotine metabolite ratio
- NNAL: 4-(methylnitrosamino)-1-(3-pyridyl)-1-butanol
- NNC: Normal nicotine content
- NNN: *N*′-nitrosonornicotine
- OASIS: Overall Anxiety Severity and Impairment Scale
- OUD: Opioid Use Disorder
- PANAS: Positive and Negative Affect Schedule
- PHQ: Patient Health Questionnaire
- PSS: Perceived Stress Scale
- QSU: Questionnaire of Smoking Urges
- RNC: Reduced nicotine content
- SST: Stop Signal Task
- TLFB: Timeline Follow Back
- TPQ: Time Perspectives Questionnaire
- VLNC: Very low nicotine content
- WASI-II: Wechsler Abbreviated Scale of Intelligence-II
- WISDM: Wisconsin Index of Smoking Dependence Motives

Protocol

1. OBJECTIVE

Despite marked reductions in cigarette smoking prevalence in the general U.S. adult population over the past approximately 50 years, smoking rates among women have decreased at a slower rate and have even increased among socioeconomically disadvantaged women where smoking prevalence can be as high as 40%. Smoking among women of reproductive age is a particular concern related to the potential for serious complications should they become pregnant, although smoking also increases risk for other serious adverse impacts on women's reproductive health including cervical cancer. Each of these potential smoking-related adverse health impacts are exacerbated by socioeconomic disadvantage, which is associated with heavier smoking, greater prevalence and severity of nicotine dependence, and a lower likelihood of quitting.

A national policy of reducing the nicotine content of cigarettes has the potential to be an effective method of reducing prevalence of cigarette smoking and associated adverse health outcomes. Controlled trials in the general population of smokers demonstrate that reduced nicotine content cigarettes decrease smoking rates, dependence, and toxin exposure.

Furthermore, our research during the current funding period indicates that reducing the nicotine content of cigarettes decreases the addiction potential of smoking in socioeconomically disadvantaged women, and other groups vulnerable to tobacco addiction. However, tobacco market conditions are likely to exert considerable influence over the extent to which the potential of this promising policy is realized. We hypothesize that the following two conditions will have significant moderating effects on disadvantaged women and other vulnerable populations due to their higher rates of heavy smoking, dependence, and difficulty quitting: (1) having reinforcing alternative sources of non-combusted nicotine readily available and (2) having those alternatives available under conditions that optimize appeal.

The overall goal of the proposed research is to experimentally model whether the availability and appeal of an alternative, non-combusted source of nicotine (i.e., e-cigarettes) will enhance the effectiveness of a reduced nicotine policy for cigarettes in socioeconomically disadvantaged women of reproductive age. Daily female smokers (ages 21-44) will undergo a baseline assessment period and then be randomized to one of the following four conditions: (1) normal nicotine content cigarettes (NNCCs) alone, which will serve as the control condition, (2) very low nicotine content cigarettes (VLNCCs) alone, (3) VLNCC's + nicotinized tobacco-flavored e-cigarettes (TF e-cigs), or (4) VLNCC's + nicotinized preferred flavor e-cigarettes (PF e-cigs). Participants will be asked to use only their assigned products for 16 weeks, and will visit the laboratory weekly to complete measures of smoking topography, cigarette demand using behavioral-economic purchase tasks, withdrawal and craving, and biomarkers of toxicant exposure. In Week 17, participants will be given monetary incentives to abstain from combusted cigarette use for a week and we will assess effects on cigarettes per day (CPD), cigarette demand, and craving and withdrawal.

This research will address the following specific aims:

Aim 1 (Primary): Compare the effects of (1) NNCCs alone, (2) VLNCCs alone, (3) VLNCC's + TF e-cigs, or (4) VLNCC's + PF e-cigs flavoring on total number of CPD. We hypothesize that at Week 16, cigarette smoking rates will have decreased in a linear, graded manner (condition 4 > 3 > 2 > 1) with the largest reduction in smoking rate from control levels observed in the VLNCC's + PF e-cigs condition.

Aim 2 (Secondary): To compare the effects of the four study conditions on cigarette demand, smoke exposure (breath carbon monoxide) and tobacco carcinogen biomarkers (NNAL, PAH metabolites). We hypothesize that at Week 16, cigarette demand and these biomarkers will have decreased in a linear, graded manner with the largest reduction observed in the VLNC's + PF e-

cig condition.

Aim 3 (Exploratory): To explore the effects of the four study conditions on abstinence- induced cigarette demand, craving, and withdrawal in socioeconomically disadvantaged women of reproductive age.

The integrative theme of this TCORS is vulnerable populations. The proposed research is relevant to FDA CTP's scientific domains of Addiction and Behavior by addressing whether reducing the nicotine content of cigarettes reduces cigarette use, dependence, and product appeal, and whether these effects are enhanced by availability of appealing alternative sources of non-combusted nicotine. It addresses Health Effects by assessing tobacco toxicant exposure and other biomarkers. The proposed study is significant and innovative by modeling the impact of a national reduced nicotine standards policy for cigarettes in this understudied population. Finally, it is programmatic as it builds upon and extends the work our team accomplished during the current funding period.

2. SIGNIFICANCE

The 2009 Family Smoking Prevention and Tobacco Control Act (FSPTCA) gives the Food and Drug Administration (FDA) regulatory authority over tobacco products, including nicotine levels in cigarettes. That is an exciting development as it creates the opportunity to examine the Benowitz and Henningfield hypothesis[1] that smoking prevalence, nicotine dependence, and smoking-related morbidity and mortality can be lowered substantially by reducing the nicotine content of cigarettes to non-addictive levels. Computer modeling predicts that reducing nicotine levels in cigarettes would produce substantial improvements in population health.[2] An essential initial step towards the implementation of such a policy is to thoroughly investigate its potential effectiveness, safety, and potential unintended adverse consequences. Indeed, the FDA's Center for Tobacco Products (FDA CTP) seeks to continue programs of multidisciplinary research to assist with the mission of investigating such regulatory matters related to the FSPTCA (see RFA-OD-17-006). The FDA explicitly notes that researching tobacco regulatory questions, including the Scientific Domains of addiction and behavior, in vulnerable populations is a crosscutting agency priority, listing women of reproductive age (21-44) among the vulnerable populations of interest.

Despite marked reductions in cigarette smoking prevalence in the general U.S. adult population over the past approximately 50 years, smoking rates have decreased at a slower rate among women than men, and even increased among economically disadvantaged women.[3,4] We recently examined tobacco and other nicotine delivery product use among women of reproductive age using the first wave of data from the Population Assessment of Tobacco and Health (PATH) dataset.[5,6] Approximately 21% were current smokers, but smoking was strikingly overrepresented among socioeconomically disadvantaged women with nearly twice as many women of reproductive age with a high school degree or less being current smokers compared to women with more education (35% vs. 19%, respectively). These data underscore a robust and pervasive inverse association between educational attainment and smoking among women of childbearing age.[7-10]

2.1 Special Health Risks of Smoking Among Women

In addition to the adverse health consequences of smoking that cross genders, smoking also has adverse consequences specific to women's reproductive health. Women who smoke have an increased risk of cardiovascular disease, with women who smoke and use oral contraceptives – a common combination among women of reproductive age - having a dose-dependent higher risk of heart attacks and strokes.[11,12] Women who smoke also have increased risk of cervical cancer, infertility, and early menopause.[13-16] Rates of unintended pregnancy are also high in this population (61%),[17] while smoking cessation during pregnancy is relatively low (25-35%),[6, 18, 19] introducing additional risk for serious adverse maternal and fetal/neonatal consequences. For example, smoking during pregnancy increases risk for placenta previa, placental abruption, premature rupture of membranes, and preterm delivery. Meta-analyses report increased risks of

20-90% for these complications among smokers, with the highest risks among heavier smokers.[20-22] A robust relationship between smoking and intrauterine growth retardation (IUGR) has also been demonstrated in many studies,[15] with increased risks of more than 50% among smokers.[23,24]

2.2 Empirical Evidence on Reduced Nicotine Content Cigarettes

While results of extended exposure to very low nicotine content cigarettes (VLNCCs) among more socioeconomically stable smokers are quite promising, little is known about specific effects that VLNCCs may have in disadvantaged women and other more vulnerable populations. With these more medically and socially unstable populations, it was agreed that an acute, laboratory model was an appropriate and safe setting to begin examining reduced nicotine content cigarettes. As described in more detail in the Overall Plan (Section 5.C.3), during the current funding period we completed multisite, double-blind, within- subject assessments of acute response to research cigarettes with varying nicotine content; the first was a small pilot study[25] followed by three larger multisite trials.[26] Participants in these studies were socioeconomically disadvantaged women of reproductive age (21-44), women and men with affective disorders, and women and men with opioid dependence, as smoking prevalence in each of these populations is significantly above prevalence for the U.S. adult population (30%, 32% and 92% vs. 21%, respectively) (National Survey on Drug Use and Health, 2016).[27]

Figure 1

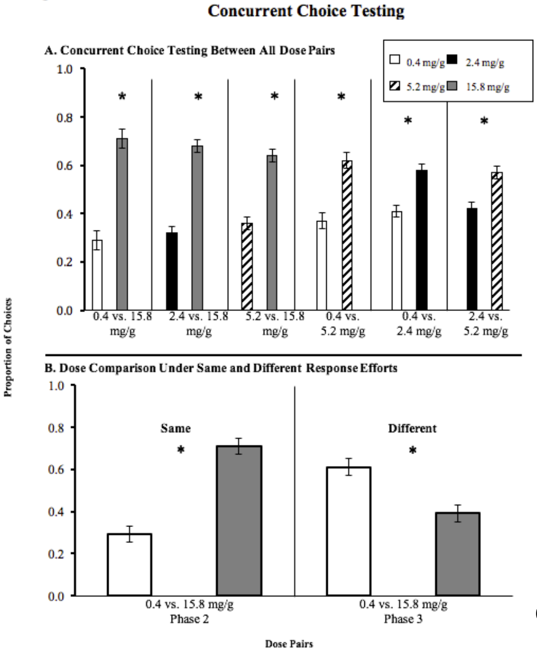

Following brief smoking abstinence, participants were exposed to the varying nicotine dose cigarettes (0.4, 2.3, 5.2, 15.8 mg nicotine/g tobacco) across fourteen 2-4 hour outpatient test sessions. Addiction potential of the cigarettes was assessed using concurrent choice testing and behavioral economic simulation modeling (i.e., Cigarette Purchase Task). Subjective effects were assessed using validated questionnaires, and smoking topography and smoke exposure by a Clinical Research Support System (CReSS) device and expired breath carbon monoxide testing, respectively. In both reports and in all three populations, reducing nicotine content decreased the reinforcing value of smoking. The 0.4 mg/g dose differed significantly from the 15.8 mg/g dose (representative of the nicotine content of commercial cigarettes) across all measures of addiction potential, including preference in concurrent choice testing (Figure 1, Panel A). The only difference between populations in that regard was seen in the 0.4 versus 2.4 mg/g dose comparison, where smokers with affective disorders chose the higher dose more often, while disadvantaged women and those with opioid dependence did not exhibit a significant preference between those two doses. Importantly in terms of regulatory implications, preference for higher over lower nicotine- content cigarettes could be reversed by increasing cost of obtaining the higher dose (Figure 1, Panel B). All doses reduced withdrawal symptoms; compensatory smoking was not observed. The consistency of effects noted across the three vulnerable populations underscores the generality of these results, especially regarding the control that nicotine content exerts over smoker preferences despite considerable individual differences.

These initial studies assessed acute response in a laboratory setting, leaving unanswered whether results can be generalized to vulnerable populations chronically using reduced nicotine content cigarettes in naturalistic settings. That question is being answered in our multisite, double-blind field trials in the same three vulnerable populations which are currently underway. Preliminary results for total CPD (study and non-study cigarettes) from initial study completers in each population (41 economically disadvantaged women of reproductive age (non-pregnant), 35 individuals with opioid dependence, and 49 individuals with affective disorders) are shown in

Figure 2. We show results for the 15.8 mg/g control dose representative of commercial cigarettes and the VLNC 0.4 mg/g dose. Mean CPD was significantly lower among those assigned to the VLNC than control cigarette at the 12-week assessment among disadvantaged women (p=.03, d=0.70) and smokers with affective disorders (p<.006, d=0.81), but not those with opioid dependence (p = .25, d=.38). These preliminary results provide encouraging initial evidence that these vulnerable populations are sensitive to the effects of VLNCs on smoking rate. Fagerstrom dependence total scores at 12 weeks are also decreased significantly among those assigned to VLNCs compared to the 15.8 mg/g dose in the disadvantaged women and those with affective disorders but not the opioid dependent population, although we are not yet seeing any evidence of VLNCs increasing measures of quitting in any of the three populations, which, while still preliminary, could represent an important difference in response to VLNCs between healthier and more vulnerable populations of smokers in whom rates of quitting are well known to be lower.

Our work during the Phase 1 funding period indicates that economically disadvantaged women, like more socioeconomically stable smokers, respond to reductions in the nicotine content of cigarettes with reductions in cigarette demand and other measures of addiction potential. However, tobacco market conditions are likely to exert considerable influence over the extent to which the promising effects of reduced-nicotine cigarettes are realized in the natural environment. This may be particularly true of female smokers, who are more motivated to smoke for nonpharmacological reasons as compared to men (e.g.,[28]). Use of electronic cigarettes (e-cigarettes) is increasing sharply in the US and it is important to consider the potential effects of e-cigarette use on a nicotine reduction policy for cigarettes. We believe that encouraging the use of e-cigarettes, by making them more appealing to smokers, may enhance the efficacy of a reduced-nicotine policy for cigarettes.

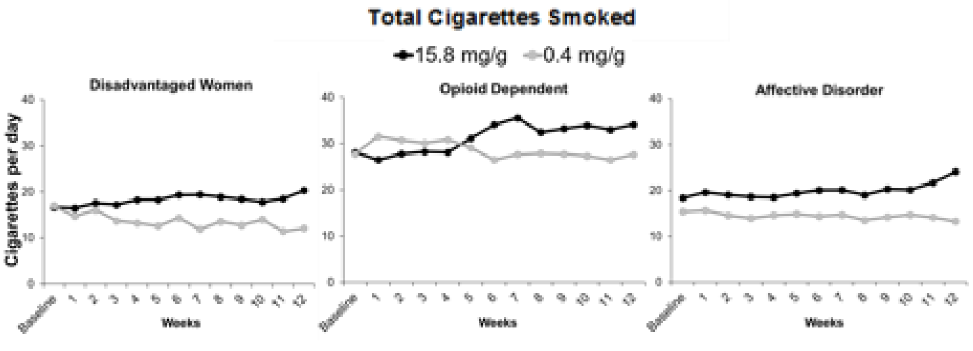

Figure 2. Total cigarettes smoked across 12-week period in three vulnerable populations.

2.3 Relevance of the Project to the Integrative Theme and Goals of the TCORS

The integrative theme of this TCORS is **vulnerable populations**, and its goals are to model the potential effects of tobacco product standards on product use in vulnerable populations, with the goal of reducing the risks of product use, dependence, and product- related adverse health outcomes. For the FDA to effectively execute its tobacco regulatory responsibilities, it must have sound scientific evidence on how product standards impact tobacco use in populations with high rates of tobacco dependence. Our goal is to provide the FDA with that evidence. This project is relevant to that goal because it will examine the effects of a reduced-nicotine standard for cigarettes, alone and combined with another FDA- regulated product, e-cigarettes, on measures of cigarette use, demand, dependence and tobacco toxicant exposure in this vulnerable population.

2.4 Relevance to the Scientific Domains and Priorities of the FDA CTP

This project is highly relevant to CTP's domain of **Addiction** ("understanding the effect of tobacco characteristics on addiction and abuse liability"). By comparing the effects of VLNCCs and NNCCs on smoking rates and cigarette dependence this project addresses the first priority in this domain, which is understanding the impact of changes in tobacco product characteristics on dependence. By examining whether e-cig availability and flavors that increase e-cig appeal increase the effects of VLNCCs on smoking, this project addresses the second priority in this domain, which is understanding differences in dependence and tobacco use patterns with use of low-nicotine content cigarettes in context with other tobacco products. Several other domains and priorities of the FDA CTP will also be addressed. The **Health Effects** domain refers to

understanding the short- and long-term health effects of tobacco products. This project will address this domain by assessing effect of VLNCCs alone and with e-cigs on biomarkers of tobacco-related carcinogen exposure and respiratory disease. The **Behavior** domain refers to the goal of understanding knowledge, attitudes, and behaviors related to tobacco product use and changes in tobacco product characteristics. This project will assess this domain by examining how reducing the nicotine content of cigarettes and e-cig flavors affect measures such as product appeal, health risk perceptions, and cigarette use.

2.5 How Study Outcomes Will Improve Scientific Knowledge Related to the Manufacture, Distribution and Marketing of Tobacco Products.

Study outcomes will directly inform scientific knowledge concerning tobacco product manufacturing by testing whether a reduction in nicotine content of cigarettes to ≤ 0.4 mg nicotine/g tobacco would reduce smoking in this vulnerable population. The outcomes will also indicate whether continuing to allow the sale of flavored e-cigs improves the efficacy of a reduced-nicotine standard for cigarettes on smoking reduction in this population.

3. RATIONALE

3.1 Use and Effects of E-Cigarettes

E-cigarettes consist of a cartridge containing an e-liquid solution of nicotine, propylene glycol (PG), vegetable glycerin (VG), flavorings, and other additives, which is heated with a battery-operated atomizer that vaporizes the solution. The vapor is inhaled and absorbed through the mouth, throat and lungs. E-cigarette subtypes include first generation products that resemble cigarettes (“cigalikes”) and are disposable or rechargeable, second generation products that often resemble pens and have reservoirs that are refilled using e- liquid purchased separately, and third generation devices that have use larger batteries, adjustable power delivery and replacement heating coils and wicks.[29] Nicotine levels from e-cigarettes can be comparable to those observed with cigarettes, depending on e-cigarette characteristics, e-liquid nicotine content and user topography.[30-33] Potential risks of e- cigarette use include exposure to low levels of carcinogens, toxicants and metals in the vapor, and cytotoxic effects of flavors.[34,35] However, toxin and carcinogen levels from e- cigarettes appear to be far lower than those from cigarettes.[36,37] Former smokers who had switched to e-cigarettes had 59-99% lower levels of 6 tobacco toxicant and carcinogen metabolites than ongoing smokers,[36] which were comparable to reductions seen in smokers who had switched from cigarettes to nicotine lozenges.[38] Another study found that NNAL, a metabolite of the tobacco-specific carcinogen NNK, was reduced by 57% in 20 smokers who had switched to e-cigarettes for one week and by 64% in those who had switched for 2 weeks.[39]

3.2 E-cigarette Prevalence

E-cigarette use is increasing sharply in the US and may be particularly so among women, with uptake of e-cigarettes in recent years faster in females than males[40] and initial satisfaction with e-cigarettes higher among women than men.[41] We recently examined tobacco and other nicotine delivery product use among non-pregnant and pregnant women of reproductive age using the first wave of data from PATH and found that 6.2% and 4.9% were current e-cigarette users, respectively, but current cigarette smokers were orders of magnitude more likely (~ 86 times in non-pregnant women) to currently use e-cigarettes compared to never smokers.[5,6] Another recent study we conducted surveyed 800 women of reproductive age using Amazon Mechanical Turk and also found that e-cigarette use was significantly higher among current cigarette smokers and associated with use of nicotine replacement products and other activities consistent with trying to reduce or quit smoking.[42]

3.3 E-cigarette Effects on Smoking

To date, randomized clinical trials of e-cigarette effects on smoking show significant reductions in CPD, although little effect on quitting.[43-45] Modest effects on quitting in these trials may have been due to use of first generation e-cigarettes that provided variable nicotine delivery and are subject to battery failure.[46] A trial using second generation e- cigarette devices found a

cigarette abstinence rates of 34% after 8 weeks of use and 21% 6 months later, with an overall reduction in CPD of 60%.[47] E-cigarettes may reduce cigarette smoking by reducing cigarette craving and nicotine withdrawal symptoms.[48] Although early studies found that e-cigarettes were less effective than cigarettes at reducing cigarette craving and withdrawal,[49-51] second-generation e-cigarettes (those with characteristics most similar to the product proposed for this study) reduce craving and withdrawal under natural *ad lib* puff topography conditions.[30,47,52]

E-cigarette effects on cigarette craving and withdrawal symptoms are determined by the extent to which e-cigarettes are used by smokers. One factor that impacts e-cigarette use is flavors. More than two-thirds of current adult e-cigarette users in 2013-2014 used a flavored e-cigarette.[53,54] Flavors in e-cigarettes have been shown to substantially enhance the subjective appeal and relative reinforcing effects of e-cigarettes[55,56] and have been cited as a reason for e-cigarette use among adults[54,57] and key feature of e-cigarette products.[58] Experimental studies show that flavors increase demand for e-cigarettes among cigarette smokers.[59,60] particularly smokers who are not current e-cigarette users.[60] Studies of e- cigarette users also highlight that flavors play an important role in their experience of the product[61-63] and in reducing cigarette consumption and craving.[62,64]

Few studies have examined the effects of e-cigarettes expressly in women. One recent study reported on gender effects in a study comparing smokers of combusted cigarettes to experienced dual users of combusted cigarettes and e-cigarettes and examined gender effects.[65] All participants engaged in week-long periods of ad lib use interspersed with periods of combusted cigarette reduction. Compared to smokers of combusted cigarettes, dual users smoked the same number of cigarettes during ad lib periods, but quadrupled their use of e-cigarettes during smoking reduction periods and were more successful at achieving smoking reduction. Among women, dual use was associated with higher nicotine levels and greater withdrawal suppression compared to women smoking combusted cigarettes. These results suggest that e-cigarettes may be a meaningful alternative to combusted cigarettes among women. Also encouraging in terms of the aims of the proposed study are data showing that women are more likely than men to use flavored (e.g., menthol, fruit, candy/sweet flavors) tobacco products compared to men.[54,66] Another recent survey of women of reproductive age who have used e-cigarettes reported that 71% of them have used flavored e-cigarettes.[67]

3.4 Products to be Tested

<u>Cigarettes to be assessed</u>

The cigarettes to be used in this study were made under an NIH contract with production being overseen by the Research Triangle Institute (referred to as “Spectrum cigarettes”). NIH currently has approximately 10 million of these cigarettes (of varying types) for research purposes. The cigarettes selected for the study span the range of yields likely to produce the hypothesized effects, as described above. Spectrum cigarettes are not currently commercially available, although they are similar in many ways to marketed cigarettes (e.g., similar manufacturing, filter, paper, etc.).

<u>E-cigarettes to be assessed</u>

Both the JUUL and the Vuse Solo will be used and assessed in this study. While JUUL will be offered to all participants, participants that are unwilling to use JUUL will be offered the Vuse Solo.

JUUL is a commercially available closed system containing two components. One component contains a lithium-ion battery (200 mAh), nichrome coil heater, silica wick, and stainless steel vapor path. The other component is the prefilled e-liquid container that also serves as the mouthpiece. Each commercially available cartridge holds approximately 0.7 mL of e-liquid containing approximately 40 mg of nicotine or 5% nicotine by weight (NBW). A lower dose containing approximately 23 mg of nicotine per cartridge or 3% NBW is also marketed but will not be used in this study. All containers contain glycerol, propylene glycol, natural oils, extracts and flavors, nicotine, benzoic acid. We will not alter the e-liquid in any way. The research staff will distribute the e-liquid containers as purchased from the manufacturer. The JUUL apparatus and

5% NBW e-liquids that will be used are legally purchasable and have been as of August 8, 2016. We will not alter them in any way.

Vuse Solo is a commercially available closed system containing two components. The power/heating device includes a 270 mAh battery, silica wick, microchips, and sensor. The other component is the prefilled e-liquid container. Each commercially available cartridge holds approximately 1 mL of liquid containing 48 mg of nicotine or 4.8% NBW. All containers contain vegetable glycerin, propylene glycol, reverse-osmosis water, glycerin, flavorings, and nicotine. The research staff will distribute the e-liquid containers as purchased from the manufacturer. The Vuse apparatus and e-liquid cartridges that will be used are legally purchasable and have been as of August 8, 2016. We will not alter them in any way.

3.5. Summary

Although cigarette smoking is declining in the overall US population, smoking rates have decreased at a slower rate among women than men, and even increased among economically disadvantaged women. Data from the current funding period indicate that VLNCCs significantly reduce addiction potential of smoking during acute exposure and reduce overall smoking rates during extended exposure in disadvantaged women and it is highly encouraging that a policy of reduced nicotine standards in cigarettes can benefit this vulnerable population. However, considering the modest effects of VLNCCs on quitting thus far in studies in the general population of smokers, and the difficulties that disadvantaged women and other vulnerable populations have with quitting smoking, we believe that other market conditions may need to be in place for the considerable potential of reduced nicotine standards to be realized. One such policy that we deem critical is the ready availability of alternative, non-combusted sources of nicotine. The elevated rates of e-cig uptake among women noted above indicate that they find e-cigs highly reinforcing, which is encouraging. We also believe that the appeal of e-cigs will have to be enhanced. The method for enhancing appeal that we are proposing to investigate is availability of flavors. That market condition also appears to have considerable potential to benefit disadvantaged women in light of the greater preference that women have for flavored tobacco products.

So, while there is considerable reason for optimism, there is also tremendous need for empirical evidence as there has only been a single controlled study reported examining how the effects of VLNCCs are moderated by the availability of non-combusted sources of nicotine, and that was a preliminary study in the general populations of smokers. As such, the proposed study has the potential to contribute significant new knowledge of relevant to the FDA CTP regarding the potential impact of a reduced nicotine standards policy in this vulnerable population of smokers. The research is also programmatic, as it will build on and complement the work that the UVM TCORS team of investigators has accomplished during the current funding period on the effects of reduced nicotine content cigarettes in disadvantaged women and other vulnerable populations.

4. Project Study Methods

This study will use a four-condition, parallel-groups research design. After a baseline period in which daily smoking rate and other baseline assessments are completed, participants will be randomly assigned to one of the following four conditions for a 16-week experimental period: (1) normal nicotine content cigarettes (NNCCs, 15.8 mg/g) alone, which serves as the control condition; (2) very low nicotine content cigarettes (VLNCCs, 0.4 mg/g) alone; (3) VLNCCs + tobacco-flavored nicotinized e-cigs (TF e-cig, 4.8 - 5.0% nicotine by weight, NBW, if they choose to use the Vuse or JULL devise, respectively); or (4) VLNCCs + preferred-flavor nicotinized e-cigarette (PF e-cig).

5. Study Screening Procedures

5.1 Participants

Participants will be women ages 21–44 years who have a high school education or less. For inclusion, participants must smoke ≥ 5 cigarettes per day, have smoked daily for ≥ 1 year and have breath CO levels ≥ 8 ppm or have positive urine cotinine results. Additionally, they must be sufficiently literate to complete the research tasks, be in good physical health without serious illness or change in health in past three months, and have the technological capabilities to complete weekly face-to-face video assessments and the compatibility to use ico Smartphone Smokerlyzers for assessing breath carbon monoxide (CO) levels. *Exclusionary criteria*: We will exclude those under the age of 21 for scientific and legal reasons. We will exclude pregnant and nursing women and women not reporting use of contraceptives, anyone currently using nicotine replacement, bupropion or other pharmacotherapies as cessation aids, those who report daily use of e-cigarettes in the past 30 days as they may not be compliant with the study e-cigarettes, and anyone intending to quit within 3 months, as participation in this study may not reduce smoking and could increase smoke and/or nicotine exposure. Those who exclusively use roll-your-own cigarettes or who use other non-cigarette tobacco products (e.g., cigars, smokeless) ≥ 10 days in the past 30 days are excluded because their use of these products may reduce the validity of our main outcome measure, total cigarettes per day (which includes study and non-study cigarettes). The following conditions are excluded as they could affect participants' abilities to complete the study: unstable medical or medication conditions (significant changes in a serious medical condition in the past 3 months, medication changes in the past 4 weeks; details provided in Protection of Human Subjects section), symptoms of psychosis or dementia, past-month suicidality, suicide attempt in the past 6 months, current (past-6 months) alcohol or substance use disorders other than nicotine, positive toxicology screen for illicit drugs (marijuana allowed; one re-screen opportunity), positive breath alcohol level (BAL) at screen (one re-screen opportunity).

5.2 Recruitment

This study will be conducted at two sites, University of Vermont in Burlington, VT (primary site) and Johns Hopkins University in Baltimore, MD. This collaboration will permit us to complete studies in a timely manner, enroll a more ethnically diverse sample, and facilitate meaningful collaboration across multiple sites, which is one goal of this funding announcement. Power estimations were based on preliminary evidence from trials conducted during Phase 1 UVM TCORS funding which included an approximately 15% dropout rate across populations. For the analyses of primary aims using an intent-to-treat approach, we require 53 randomized in each condition (212 total) to test our primary aims. We estimate 10% attrition prior to randomization and will therefore enroll up to 59 per condition (236 total) to randomize 212. In addition, we will pilot study procedures with 5 participants per e-cigarette condition (10 total).

Participants at both sites will be recruited through Facebook, craigslist, ads in local newspapers and on community bulletin boards, and through word of mouth. At UVM, individuals recruited from online sources will be directed to a UVM-hosted recruitment website where they will have the opportunity to select which research studies interest them. They will then be redirected to a brief online screener to assess eligibility. Those who contact us by phone will be given a description of the study and asked questions to assess eligibility. If a participant appears to be eligible, they will be invited to participate in the first portion of the screening. Research assistants will inform eligible participants that the screening will occur over video chat, and will assist the participant with setting up an appropriately secure video platform.

During this first portion of the screening, the participant will complete questionnaires through REDCap online while the research assistant is present over video chat or phone to deliver instructions and to answer any questions. The participant will then answer interviewer-administered questionnaires over video chat. Participants who did not yet set up their video platforms will do so with the research assistant before beginning any questionnaires. Participants will be instructed to have picture identification (e.g. driver's license) available to show the staff. If participants anticipate not having acceptable ID, staff should consult with the project coordinator or study PI. Initial study eligibility will be determined after data are collected from this visit. Participants who meet initial study eligibility will be scheduled for the second portion of the screening.

Before the second portion of the screening occurs, eligible participants will receive the equipment necessary to use for collecting physiological measurements. Participants will be asked to pick up this equipment via curbside pickup at our clinic (UVM University Health Center, UHC), which will consist of participants calling staff once they arrive at UHC and staff coming out to give participants a bag/box containing the following equipment: a Smokerlyzer; an audio jack adapter for the Smokerlyzer if necessary; a blood pressure cuff; an oximeter; a thermometer; a urinary cotinine dipstick; urine cups with attached temperature test strips; a pregnancy test strip (if applicable) and urine toxicology test strips or a saliva toxicology test. Participants (and staff) will be asked to use cloth face coverings when exchanging product. Participants may be invited to come inside to pick up this equipment if the participant is asked to wait for this exchange. All participants must pass a COVID19 screening before entering the building. If there is any waiting that needs to occur inside the building, the participant will wait inside one of our five highly ventilated smoking chambers. If there happens to be no space in the smoking chambers, the participant will be told that they can not come up to the clinic until space is available. After each use, the all of the surfaces in the smoking chambers will be cleaned with 70% or greater of alcohol solution by staff wearing a mask and gloves, as well as all of the door handles. If the participant uses the bathroom while they are in the clinic, the bathroom surfaces and handles will be wiped down after use by staff while masks and gloves are worn. Participants who are using the smoking chambers at any point in the study to wait for product or equipment exchange will remain in the chambers until a staff member comes to knock on the door to let them out. In this way, we can avoid people coming into close contact with each other in the larger room that contains the smoking chambers. A minimum of 6 feet of distance will be maintained for all staff and participants at all times. For participants who cannot come to the clinic, a commercial courier will deliver this equipment to them before the second portion of the screening.

If at any point the Smokerlyzers are not available for distribution, we will conduct the CO test curbside before or after participants are invited inside and the courier service will not be available. Research Assistants will bring down the CO monitor to the participant. While maintaining 10 feet of distance and wearing gloves, staff will explain how the CO monitor works. Once the participant is ready, staff will press the button to obtain the measurement and will set the CO monitor down and will back away 10 feet. The participant will then come to pick up the device and will blow into the monitor. After the participant completes the test, they will set down the monitor and back up 10 feet and staff will retrieve the monitor. After every use, staff will wipe down the CO monitor with disinfectant wipes and hydrogen peroxide wipes. When using the monitor, a D-piece (a portable valve filter) must be placed into the monitor and then the single use plastic mouthpiece is placed into the D-piece. The monitor has built in SteriTouch technology to ensure optimum infection control, and the D- pieces filter out and remove 99.9% of airborne bacteria and greater than 97% of viruses for excellent infection control. Each participant will be assigned their own D-piece to use throughout the study, and no D-piece will ever be shared among participants. Participants will gently exhale into the D-piece for the breath carbon monoxide reading. Participants will be instructed only to exhale through the device, not to inhale. D-piece technology also includes a one-way valve that prevents air from being drawn back from the monitor. D- pieces will also be wiped down after each use with disinfectant wipes and hydrogen peroxide wipes and stored in a container at the lab.

Once participants have received the necessary equipment to complete the physiological portion of the screening, the research assistant will initiate a video call with the participant. During this call, the participant will be instructed on how to use the equipment and then will be asked to use the equipment to obtain the following physiological readings: blood pressure, heart rate, oxygen saturation, temperature, and breath CO levels. Participants will also be asked to collect a urine or saliva sample during the visit. If a saliva sample is collected, the participant you will provide the saliva sample over video chat while the staff observes. If a urine sample is collected, staff will ask participant to bring this urine sample to the video screen after collection to perform a urine toxicology test and a pregnancy test (if applicable). These urine cups will have temperature strips affixed to ensure that the sample is valid. Participants who have a carbon monoxide level of less than or equal to 8 will also be asked to use the urinary cotinine dipstick to determine whether

they are positive for cotinine. The participant will obtain the physiological readings and perform the tests and then will hold the results of the test up to the camera so that the research assistant can interpret and record the readings on REDCap, Participants will also be instructed to have handy a pack of their usual brand cigarettes, all prescription medications they are currently taking and identification (example, driver's license) during this second portion of the screening visit. If participants anticipate not having acceptable ID site staff should consult with the project coordinator or study PI.

A participant must complete his/her two-part screening session within 90 days of completing the pre-screening questionnaire. If the participant is not able to complete the two-part screening visit in that timeframe, he/she will need to complete the pre-screening questionnaire again.

5.3 Informed Consent Process:

Before beginning the informed consent process, potential participants will need to produce identification as described above. The interviewer will confirm the age and identity of the participant. If the participant is not between the ages of 21 and 44 or has a degree greater than high school, she will be dismissed without payment. During the first portion of the screening session, study information will be presented and documentation of the participant's informed consent via electronic signature on REDCap will be required prior to participating in the screening session. In order to ensure adequate informed consent, participants will be asked to read the first several lines aloud (to determine literacy) and will then be given ample time to read the consent document. If the interviewer suspects the participant is not literate, he or she will have them continue reading further to confirm. Inability to read and comprehend written study materials will result in ineligibility and the interviewer will inform the participant that they are not eligible. Only after the participant and the researcher are fully satisfied that the participant understands the purpose of the study, the confidentiality of the data, the procedures, the risks/benefits and his/her rights as a research participant will the consent form be signed and the participant undergo screening procedures.

5.4 Screening Measures

Those who consent will be screened for eligibility using the following measures:

The following physiological measures will be collected and recorded directly into REDCap by the interviewer:

1. Expired breath carbon monoxide (CO) levels will be assessed using an ico Smokerlyzer Smartphone Monitor (Covita – for remote collection) or a Bedfont CO monitor (for curbside collection), a reliable and valid measure of recent smoking.
    a. Urinary cotinine test strips will be used to asses cotinine levels if a participant's CO reading is less than or equal to 8 ppm.
2. A urine or saliva toxicology screen will be performed to assess the presence of illicit drugs including up to the following drugs: marijuana, cocaine, opiates, oxycodone, benzodiazepines, barbiturates, amphetamines, methadone, buprenorphine, methamphetamines, MDMA and PCP. Participants who fail the drug screen for drugs other than marijuana or their prescribed opioid medication may reschedule the interview but will need to be re-consented to ensure they have received adequate informed consent. They will be excluded if they are positive for drugs (other than marijuana or prescribed medications as determined by PI on a case-by-case basis) the second time.
3. Urine Pregnancy Test (HCG detection) will be performed for all participants.
4. Blood pressure and heart rate will be measured using an automated blood pressure monitor and a finger pulse oximeter to help the licensed medical professional determine final participant eligibility. Participants will be told if their blood pressure is in an abnormal range and advised to see a doctor by research staff. The research staff will also submit a medical event form for the LMP to review along with a Blood Pressure and Heart Rate Symptom Checklist form to ascertain details of the symptomatology for the LMP to review. In severe cases, the LMP may also choose to call the participant to follow-up and/or withdraw the participant from the study if necessary. All of these procedures are documented in our Blood

Pressure/Heart Rate Collection: Standard Operating Procedure form which we can submit to the IRB if the Committee deems necessary.

5. Body temperature, respiratory rate and oxygen saturation will be added as physiological measures based on the CDC recommendations and those of Dr. David Kaminsky. https://www.cdc.gov/tobacco/basic_information/e-cigarettes/severe-lung-disease/healthcare-providers/index.html

The following screening assessment will be administered as an interview:

1) The Mini International Neuropsychiatric Interview (MINI) 7.0[70]

The following screening assessments will be administered as an interview and entered directly into REDCap by the interviewer:

1) The MINI Plus Modules
2) The MINI suicide subscale[71] to evaluate suicide risk.
3) MINI Follow-up Questionnaire (if applicable)
4) Tobacco Use History and Exposure Questionnaire, which measures variables such as smoking amount, cigarette brand, age of initiation of smoking, number of quit attempts, duration of quit attempts and duration of smoking.
5) Smoking Cessation Therapy Use Questionnaire
6) Time Since Last Cigarette Questionnaire
7) Medical History Questionnaire to assess current diagnoses, symptoms and past health problems.
    a. Medications will be recorded directly onto the Concomitant Medications form in REDCap.
8) Drug Abuse Screening Test (DAST-10), which assesses quantity and frequency of alcohol and drug use (12 month and 1 month version)

The following screening assessments will be completed by the participant directly in REDCap, except where noted otherwise:

1) Demographic History Questionnaire, which will assess age, gender, ethnicity, race, education, income, marital status, and employment history.
2) Alcohol Use Questionnaire---based on the Alcohol Use Disorders Identification Test[72] (12 month and 1 month version)
3) Drug Use Questionnaire---based on the Drug Abuse Screening Test[73] (12 month and 1 month version)
4) Fagerström Test for Nicotine Dependence (FTND)[74];
5) Wisconsin Inventory of Smoking Dependence Motives-Brief Scale (WISDM)[75] will be administered to assess nicotine dependence severity.
6) Penn State Electronic Cigarette Dependence Index[76];
7) Smoking Stages of Change Algorithm[77];
8) Identifying Information Form will include the participant's REDCap Subject Identifier, name, address (including the county of residence), email address, phone number, age, date of birth, and social security number (if applicable).
    a. This form will be entered into the 'Identifying Information Access Database'.
        i. Each site will have a separate 'Identifying Information Access Database'.
        ii. Identifying information will not be shared with other sites. Each site is responsible for maintaining confidentiality of this information.
        iii. Identifying information will be kept in a locked file cabinet (source document) and in a password protected Access Database (electronic version) separate from all other study data.

9) Beck Depression Inventory (BDI-II)[68], to assess depressive symptoms.
(This form has been updated on 1.29.20 so as to use the BDI-II. The BDI-II will be used instead of the BDI so that the data collected will be directly comparable to other projects using the BDI-II).

10) Overall Anxiety Severity and Impairment Scale[69](OASIS); to assess frequency and severity of anxiety symptoms.

11) COVID19 Symptom Questionnaire

12) Respiratory Symptom Questionnaire will be administered to assess respiratory health

In the event that the REDCap website is not functioning, the assessments will be administered aloud and participant answers will be recorded securely. The interviewer will enter the data into REDCap when it resumes functioning properly. This information should be recorded in the 'End of Visit Evaluation Form'.

5.5 Suicidality/Mental Health Monitoring

Participants who endorse suicidal intention in the past month or a suicide attempt in the past 6 months as indicated on the BDI (score > 1 on question 9) or MINI suicide subscale (endorse question 4 and/or 5 on the MINI suicide subscale or question 6 on the MINI suicide subscale with suicide attempt in the past 6 months) or answer "yes" to question A3g on the MINI Neuropsychiatric interview and symptoms have occurred in the past two weeks will be assessed by a clinician for eligibility and possible intervention.. The research staff member will contact a licensed clinician for evaluation. In the event that no clinician is available, staff will put the participant in contact with the National Suicide Prevention Lifeline at 1-800-273-8255. They will also contact the Study Coordinator and Site PI to inform them of the situation as soon as possible. Additionally, they will contact the Project Coordinator to inform her of the situation. The participant will be paid $25 (+$25 bonus if applicable) and provided with local mental health resources. Post enrollment, any report of suicidal ideation or attempt by a participant will be grounds for immediate withdrawal from the study.

5.6 Inclusion/Exclusion Criteria

Inclusion Criteria:

1) Women ages 21-44 years who have < an Associates Degree,
2) Report smoking ≥ 5 cigarettes per day for the past year,
3) Provide an intake breath CO sample >8 ppm, (if ≤ 8 ppm, then urinary-cotinine strip must be positive)
4) Be without current (within the past year) serious mental disorder that would interfere with study results or completion as determined by the licensed medical professional or PI,
5) Be without current substance abuse/dependence other than nicotine,
6) Be sufficiently literate to complete the research-related tasks,
7) Be in good physical health without serious illness or change in health or medication in the past three months as determined by the licensed medical professional at each site
8) Have appropriate equipment to complete face-to-face video assessments and use ico Smartphone Smokerlyzer Monitors. For those who do not have a Smartphone, staff will explore potential alternate plans (e.g., project-provided inexpensive Android phone)

Exclusion Criteria:

1) Exclusive use of roll-your-own cigarettes;
2) Planning to quit smoking in the next 30 days;
3) A quit attempt in the past 30 days resulting in greater than 3 days of abstinence;
4) Significant use of other tobacco or nicotine products within the past month (more than 9 days in the past 30).
5) Currently taking anticonvulsant medications including:
    a. Phenytoin [Brand Name: Dilantin]
    b. Carbamazepine [Brand Name: Tegretol, Carbatrol, Equetro, Epitol]
    c. Oxcarbazepine [Brand Name: Trileptal]

   d. Primidone [Brand Name: Mysoline]
   e. Phenobarbital
6) Positive toxicology screen for any of the following drugs: cocaine, opiates, methadone, oxycodone, buprenorphine, benzodiazepines, barbiturates, amphetamines, methamphetamines, MDMA and PCP will be grounds for exclusion.
   a. Marijuana will be tested for but will not be an exclusionary criterion. Participants will be discouraged from smoking marijuana during the study.
   b. Participants with valid prescriptions for opiates, benzodiazepines, barbiturates, or amphetamines will not necessarily be excluded.
   c. Participants failing the toxicology screen will be allowed to re-screen once. These participants will need to be re-consented before being rescreened to ensure they have received adequate informed consent.
7) Self-report of binge drinking alcohol (more than 9 days in the past 30 days, 4/5 drinks in a 2 hour period in females/males);
8) Systolic blood pressure < 90 or ≥ 160 mmHg;
   a. Participants failing for blood pressure will be allowed to re-screen once.
9) Diastolic blood pressure < 50 or ≥ 100 mmHg;
   a. Participants failing for blood pressure will be allowed to re-screen once.
10) Breath CO > 80 ppm;
11) Heart rate is greater than or equal to 115 bpm or less than 45 bpm;
   a. Participants failing for heart rate will be allowed to re-screen once.
12) Currently seeking treatment for smoking cessation;
13) Being pregnant or nursing, or not report using an approved form of birth control if applicable determined by the Project Medical Director.
14) Have used nicotine replacement, bupropion or other pharmacotherapies as cessation aids in the past month (bupropion will be allowed for treatment of depression);
15) Current symptoms of psychosis, dementia or mania;
16) Suicidal ideation in the past month (score > 1 on the BDI question 9 or endorse question 4 and/or 5 on the MINI suicide subscale);
17) Reporting a plan or attempt to commit suicide, which is assessed on question A3g of the MINI Neuropsychiatric Interview Major Depressive Episode Module. Thoughts of suicide without an intent or plan is not an exclusion criteria;
18) Suicide attempt in the past 6 months (endorse question 6 on the MINI suicide subscale with suicide attempt in the past 6 months);
19) Participation in another research study in the past 30 days;
20) Daily use of e-cigarettes in the past month (defined as 6 – 7 days per week); or
21) Co-habitation with any research participant who has or is participating in the current study.
22) Oxygen saturation of < 90%
23) Reporting positive symptoms for COVID19

Women with academic degrees greater than high school are excluded because they have a lower risk of smoking and thus are not of interest in this experiment on smoking among socioeconomically disadvantaged women. Individuals under age 21 are excluded because they cannot legally buy cigarettes. Those with unstable medical, psychiatric, or medication conditions (as determined by the licensed medical professional) are excluded as these symptoms could affect a participant's ability to complete the study. Examples include but are not limited to the following: angina, stroke, heart attack which occurred since phone screening, blood clots in the arms or legs for which the individual is undergoing active medical treatment, cancer requiring active chemotherapy or radiation therapy, severe shortness of breath caused by conditions such as uncontrolled asthma, COPD, or arrhythmia, active untreated infection such as pneumonia, active untreated endocrine disorder such as hyperthyroidism. We will exclude those currently seeking smoking treatment and those who plan to quit in the next 30 days, as participation in this study may not lead to reductions in smoking. We will exclude pregnant or nursing women and

women of reproductive potential who are unwilling to use acceptable forms of birth control throughout the study if applicable determined by the if applicable determined by the Project Medical Director. We will also exclude anyone with current or recent alcohol or drug abuse problems as these factors could independently affect smoking behavior during the study. Individuals with baseline CO readings greater than 80 ppm, those with heart rate or blood pressure readings that are out of range (systolic: 90-159 mmHg; diastolic: 50-99 mmHg; HR: 45-114 bpm) and anyone who has attempted suicide in the past six months will be excluded from the study for safety concerns. Individuals who smoke 'roll your own' cigarettes exclusively will be excluded from the study because we will be unable to standardize their baseline smoking behavior. Individuals who have reported daily use of e-cigarettes in the past 30 days will be excluded as they may not be compliant with experimenter-provided e- cigarettes. Individuals who have recently participated in a research study will be excluded as participation may have changed their smoking patterns, which may preclude a stable smoking baseline. Because participants are required to complete portions of the protocol independently, they will need to be able to independently read and comprehend the study materials.

5.7 Eligibility Determination:

The research assistant will review the entire screening assessment battery for initial eligibility determination, confirming the participant meets the above described inclusion/exclusion criteria. All eligibility criteria that are not related to physiological measurements will be assessed during the first portion of the screening visit, and all criteria related to physiological measurements will be determined during the second portion of the screening. The final eligibility of the participant will be determined by a licensed medical professional (MD, DO, NP, PA, Master's prepared RN or CRN) at each site after reviewing the Medical History Questionnaire, BDI, Mini Neuropsychiatric Interview, and the MINI suicide subscale. The licensed medical professional may meet with a participant if available and think it necessary for eligibility determination. He/she will sign off on eligibility prior to the first baseline visit. If the licensed medical professional determines the participant is not medically eligible to participate in the study, has current symptomatology that would interfere with interpretation of the data, or is unlikely to complete the study he/she will inform the research assistants who will contact the participant prior to the first baseline visit. The licensed medical professional will not need to review the medical history forms of participants who are ineligible for other, non-medical reasons.

If a participant fails the urine or saliva toxicology screen due to a prescription medication he/she is taking, then he/she will not be automatically excluded. The interviewer will make note of this when he/she submits the forms to the licensed medical professional for final eligibility determination.

Once all the screening procedures have been completed, researchers will pay participants $25 (+$25 bonus if applicable) for their time as long as they pass the drug tests and meet the minimum requirements for carbon monoxide or urinary cotinine levels. Participants will be paid after the completion of the study visit. If participants are deemed ineligible at any point in the screening, the participant will be paid after determined ineligible. Marijuana will be tested for but will not be an exclusionary criterion. If a participant does not pass the drug test but has a current, valid prescription that would explain the failed test he/she will not be automatically excluded and will still receive the visit payment. Participants who meet all other eligibility criteria, sans the medical criteria, will be scheduled for the first baseline visit.

At the end of the screening session, the researcher will complete the End of Visit Evaluation Form. This will allow the researcher to make note of any problems encountered during the visit and to assess the truthfulness of the participant in regards to self-report of tobacco use.

6. Study Baseline Procedures

This study will use a one-week, two-session baseline period to collect baseline individual difference measures and monitor daily usual-brand smoking behavior. At Baseline 1 or within 1 business day of the Baseline 1 visit, participants will be provided their usual brand cigarettes to

smoke, equivalent to 150% of their daily smoking rate. Participants will be encouraged to come to the lab to pick up their usual brand cigarettes after they complete the questionnaires and physio for the BL1 visit. Those who cannot come to the lab will receive product via a commercial courier. A timeline follow back (TLFB) will be used during the period between Baseline 1 and Baseline 2 to assess the daily cigarette use for the first 7 days the participant has product. The participant must have received their UB cigarettes from the lab before the 7-day assessment period starts, and Baseline 2 must occur at least 7 days after the participant receives their usual brand cigarettes from the lab. If the baseline period extends past seven days and if the participant has run out of product, participants will need to purchase their own usual brand cigarettes. Use of a two-session baseline period will ensure stability of daily smoking reports, reduce reactivity to the daily cigarette monitoring, and reduce participant burden. During the two baseline sessions, participants will complete subjective questionnaires. Each visit will last approximately two to four hours. At the end of each baseline session, the researcher will complete the End of Visit Evaluation Form. This will allow the researcher to make note of any problems encountered during the visit and to assess the truthfulness of the participant in regards to self-report of tobacco use. Participants will also be supplied with urine collection equipment during the Baseline 1 product exchange so that they can collect first void urine samples during the Baseline 2 visit.

For the Baseline 1 visit and all subsequent visits, the participant will be sent a REDCap link within 15 minutes of the start of the scheduled visit to complete all of the non-interviewer administered questionnaires. The participant will complete these questionnaires on their own but can have the research assistant present on a video call if they desire. Before beginning the physiological assessment portion of the visit over video call, the research assistant must review the participant's questionnaire responses for that visit. Participants will be compensated after the completion of the study visit and when the participant has received their new product.

At Baseline 2 and all subsequent visits, after the participant has answered the questionnaires and has completed the physiological portion of the visit over video call, participants will be asked to come to the lab for exchange of product and biological samples. Participants will bring in their used and unused product from the previous visit as well as a first-void urine sample for assessing tobacco-related toxin exposure for assessing nicotine metabolism rate on applicable visits (BL2, Week 8 and Week 16) using equipment that was provided at the previous visit. Participants will be instructed to call the RA at the office when they get to the clinic to ensure that there is enough space in the smoking chambers to house all participants while abiding by safety guidelines as detailed on page 14 of the protocol. All participants must pass a COVID19 screening before entering the building. When invited into the lab, the participant will be shown to a smoking chamber and will instructed to place their bag of product outside of the chamber. The participant will wait here while the RA processes and returns product through the randomization database. Then the RA will dispense new product and bring the bag back to the participant. When the RA deems it safe for the participant to exit the chamber, the RA will instruct the participant that they can leave. The RA will instruct the participant to observe social distancing measures during this exchange, providing clarification if necessary. If a participant forgets their first-void urine sample at the Baseline 2 visit, staff will ask participant to come back to the clinic with their first void urine sample before exchange of product occurs. If participant is unable to return to the clinic with their first void sample, staff can arrange to meet the participant off campus to pick up their urine sample and to give participant their study product. Distancing and safety measures as described above must be observed. For participants who cannot make it to UHC, special arrangements will be made to enable use of the randomization database and product return/distribution procedures to the extent possible. Each week, during - or scheduled

as nearly as possible to – a virtual visit, a complete accounting of the participant's product inventory will be taken and processed remotely through the randomization database. The participant will separate product based on its type (e-cigarette or combustible inventory) and status (used/unused), and the RA will process return characteristics through the database accordingly. The RA will clarify barcode characteristics with the participant when legibility is compromised. The participant will be instructed to keep unused product in their possession, but to exchange any used product with the courier who will deliver newly dispensed replacement products within 48 hours.

Product that will be given to participants for the Baseline 2 visit cannot be given/sent to participants until 7 days have passed following completion of the Baseline 1 visit. We will need to calculate baseline smoking rate during this 7-day period and so participants cannot have access to any blinded study product or e-cigarettes before this 7-day period has ended.

6.1 Visit scheduling requirements for baseline period:

Participants will be required to schedule the Baseline 1 visit within 30 days of the completion of their screening visit. If a participant still wants to be in the study after 30 days, he/she will need to be re-screened. The participant will need to be re-consented but will maintain the original REDCap Subject Identifier. The ideal target window separating Baseline 1 and Baseline 2 is between 7 and 12 days. The minimum is 7 days and the maximum is 21 days. If the participant does not complete the visit within 21 days, then he/she will not be rescheduled and will be discontinued from the study.

6.2 Measures/Assessments

The following physiological measures will be collected and recorded directly into REDCap by the interviewer:

1) CO
2) Blood Pressure
3) Heart Rate
4) Body temperature
5) Oxygen saturation
6) Respiratory rate
7) Urine or Saliva Toxicology
8) Urine Pregnancy test (if applicable, to be performed every 2 weeks)

The following assessments will be administered as an interview at Baseline 1 and entered directly into REDCap by the interviewer:

1) <u>Concomitant Medications Form</u>
2) <u>Health Changes Questionnaire,</u> which will assess any weekly health changes,
3) <u>Time Since Last Cigarette Questionnaire</u>

The following Baseline 1 assessments will be completed by the participant directly in REDCap:

1) <u>BDI</u>
2) <u>OASIS</u>
3) <u>COVID19 Symptom Questionnaire</u>
4) <u>Respiratory Symptom Questionnaire</u>
5) <u>Wisconsin Inventory of Smoking Dependence Motives-Brief Scale (WISDM)</u> will be

administered to assess nicotine dependence severity.

6) Perceived Health Risks Rating[78], a measure of the perceived addictive potential and other health risks associated with cigarettes;
7) Perceived Stress Scale (PSS)[78], assessing the degree to which life situations are perceived as stressful;
8) Positive and Negative Affect Scales (PANAS)[79], a measure of changes in positive and negative mood;
9) Respiratory Health Questionnaire, a UVM measure of cough, shortness of breath and other respiratory symptoms;
10) Minnesota Nicotine Withdrawal Scale (MNWS)[80], a measure of nicotine withdrawal;
11) Questionnaire of Smoking Urges-brief scale - Usual Cigarette (QSU)81, which measures the urge to smoke;
12) Vaping Craving Questionnaire (VCQ)82, which measures the urge to vape;
13) Cigarette Evaluation Scale – Usual Cigarette (CES)[83], which measures responses to cigarettes (e.g., reward, satisfaction);
14) Vaping Evaluation Scale (VES), which measures responses to vaping (e.g. reward, satisfaction)
15) Cigarette Purchase Task – Usual Brand Version (CPT)[84], a self-report analogue of a progressive-ratio schedule that measures the relative reinforcing efficacy of cigarettes by querying how many of that day's cigarette they would consume in a day at varying prices. This task will indicate whether prolonged VLNC cigarette use reduces cigarette demand and increases sensitivity to increases in cigarette costs;

All participants will also be asked to select their top three flavors of e-cigarette liquid from a list read to them by the RA. This question will be asked in preparation for giving flavored pods to participants who are randomized into the flavored e-cigarette condition. This information will be recorded directly into REDCap. Participants will be asked to rate these top three flavors based on either previous experience with these flavors or to indicate how much they believe that they will like or dislike the flavors.

The following Baseline 2 physiological measures will be collected and recorded directly into REDCap by the interviewer:

1) CO
2) Blood Pressure
3) Heart Rate
4) Body temperature
5) Oxygen saturation
6) Respiratory rate
7) Urine or Saliva Toxicology
8) Urine Pregnancy (if applicable; to be performed every 2 weeks)

The following assessments will be administered as an interview at Baseline 2 and then entered directly into REDCap by the interviewer:

1) Concomitant Medications Form
2) Health Changes Questionnaire
3) Time Since Last Cigarette Questionnaire

The following assessments will be administered at Baseline 2 and completed by the participant directly in REDCap:

1) BDI
2) OASIS
3) COVID19 Symptom Questionnaire
4) Respiratory Symptom Questionnaire
5) MNWS
6) WISDM

7) PANAS
8) QSU (usual brand)
9) Vaping Craving Questionnaire
10) CES (usual brand)
11) Vaping Evaluation Scale
12) CPT (usual brand)
13) E-cigarette flavor rating questionnaire
    a. Participants will rate only the flavors that they received from staff for this visit

In the event that the REDCap website is not functioning, the assessments will be administered aloud and participant answers will be recorded securely. The interviewer will enter the data into REDCap when it resumes functioning properly. This information should be recorded in the ‘End of Visit Evaluation Form’.

6.3 E-cigarette Training Session (Baseline 2):

Participants assigned to an e-cig condition will be told that they will be provided with a JUUL. If a participant indicates an unwillingness to use the JUUL device, the research assistants will offer the participant the Vuse Solo as alternative device. If the participant does not wish to use either device, he or she would be ineligible for the study.

Participants in the e-cigarette conditions will be given e-cigarette pods BEFORE the training session occurs. These pods will be either picked up at the lab (preferred) or delivered to the participant 1 to 2 days before their Baseline 2 visit occurs. Participants randomized to the e-cigarette conditions will be notified before their Baseline 2 visit (but after the 7-day period following Baseline 1) and informed of their e-cigarette condition. Participants randomized to the flavored condition will be given pods of up to three flavors of their choice. Staff will calculate how many total pods participants will be given at this time based on their smoking rate, and participants will be able to choose the proportion of each flavor that they would like to receive.

The e-cigarette training session will occur over video chat after the physiological measurements have been collected. The first 30 minutes of the training session will consist of the participant being taught how to use, charge, and replace pods. Participants will first try their JUUL using the tobacco flavor. At this point, participants who are in the tobacco-only flavor condition will conclude their training session.

For all visits following Baseline 2, participants in the preferred flavor condition are permitted to take up to three flavors home per week but can choose to take less than three flavors if desired. Participants will take home their chosen pods (or cartridges) of up to three flavors. Participants will be able to change their flavors at only one point in the study if they desire. Participants will only be allowed to take home three flavors at one time. Participants will be given an E-cigarette instructional manual that reviews the e-cigarette training done at this visit. Participants will be encouraged to call with any device issues.

6.4 Interactive Voice Response System:

At the end of the first baseline visit, participants will be trained to use the Interactive Voice Response (IVR) System, which will contact participants each day throughout the study and ask about their smoking behavior as well as withdrawal symptoms the week before and after Baseline 2. We will also review the IVR adherence incentive program, which consists of $1 per call plus a $10 bonus for seven consecutive calls.

The IVR system is operated by TeleSage (https://telesage.com/about/). To be enrolled in the IVR system, research staff will enter the participant’s initials, telephone number, subject identifier, and visit dates into the IVR TCORS website. Identifying information (initials and telephone numbers) will not be extracted as part of the data by the bioinformatics group. Please refer to TeleSage’s privacy statement and HIPAA compliance form for additional information.

Baseline 2 biological specimens:

Urine sample for smoking biomarker assessment**:**

Participants will be asked to provide a urine sample (first void of the day) at the second baseline session and to post-randomization weeks 8 and 16 for biomarker assessment. Biomarker analysis will provide nicotine and carcinogen exposure outcome measures and verify compliance with VLNC cigarettes. Samples will be stored at -80C. Urine samples will be analyzed for total nicotine (cotinine plus its glucuronide conjugate, a useful measure of daily nicotine exposure), the tobacco- specific nitrosamine 4-methylnitrosamineo-1-(3-pyridyl)-1-butanol (NNAL), and metabolites of 4 polycyclic aromatic hydrocarbons (PAHs), which are biomarkers of tobacco smoke carcinogens and decrease upon tobacco cessation or reduction. Anatabine is a minor alkaloid that is reduced in users of VLNC cigarettes and e-cigs. Therefore, anatabine levels in samples from those assigned to the VLNCC, VLNCC+TF e-cig and VLNCC+PF e-cig conditions should be lower than levels from those in the NNCC condition. These analyses will be performed by the Murphy lab at the University of Minnesota.

Biomarker shipping and storage:

Biomarkers will be shipped quarterly to the University of Vermont Laboratory for Clinical Biochemistry Research (Tracy Lab). The Tracy Lab will serve as a central repository for all biomarker specimens and will be responsible for distributing specimens to the appropriate labs on a quarterly basis. Urine samples will be analyzed and stored at the University of Minnesota Murphy Lab.

7. Study Experimental Procedures

7.1. Experimental Period:

Participants will be seen weekly throughout the 16-week experimental period. Weeks 4, 8, 12, 16 and the abstinence visit will take approximately 2-4 hours each. All other sessions will last approximately 2 hours. If a participant has a positive urine or saliva toxicology test or is visibly intoxicated as determined by slurred speech, swaying, or stumbling, the session will be rescheduled until a negative test result is obtained and intoxication is not present. As a part of each experimental visit, participants will be asked to come to UHC for a product exchange. All participants must pass a COVID19 screening before entering the building. Participants will be instructed to contact the RA at the office when they get to the clinic to ensure that there is enough space in the smoking chambers to house all participants while abiding by safety guidelines as detailed on page 14 of the protocol. All participants must pass a COVID19 screening before entering the building. When invited into the lab, the participant will be shown to a smoking chamber and will instructed to place their bag of product outside of the chamber. The participant will wait here while the RA processes and returns product through the randomization database. Then the RA will dispense new product and bring the bag back to the participant's smoking chamber and leave it on the ground in front of the chamber. When the RA deems it safe for the participant to exit the chamber, the RA will instruct the participant that they can leave. The RA will instruct the participant to observe social distancing measures during this exchange, providing clarification if necessary. At the end of each experimental session, the researcher will complete the End of Visit Evaluation Form, which will be filed in the participant's binder. This will allow the researcher to make note of any problems encountered during the visit and to assess the truthfulness of the participant in regards to self-report of tobacco use and compliance to study procedures.

Visit scheduling requirements for experimental period:

The ideal scheduling window between each visit is 7 days based on the date of the Baseline 2 Visit. For additional scheduling requirements, refer to the '*Scheduling Visits SOP*'. If a participant misses a visit and is unable to reschedule during the window (± 3 days), that visit will not be 'made-up' in the future. All measures that were not completed will be considered missing data and will not be collected during future visits. If a visit mistakenly occurs outside of the designated window, this is a protocol deviation. A 'Non-Medical Event Form' will need to be completed. Additionally, each visit should occur at approximately the same time of day ± 2 hours.

If a participant is not able to attend his/her Week 16 visit, then it should be rescheduled even if it is outside of the scheduling window. This will be documented as a protocol deviation.

7.2. Experimental Visits Weeks 1, 3, 5, 7, 9, 11, 13, and 15 Procedures

7.2.A. Measures/Assessments

Physiological Measures Collected and entered directly into REDCap by the interviewer:

1) CO
2) Blood Pressure
3) Heart Rate
4) Body temperature
5) Oxygen saturation
6) Respiratory rate
7) Urine or Saliva Toxicology
8) Urine Pregnancy test (if applicable, to be performed every 2 weeks)

The following assessments will be administered as an interview and entered directly into REDCap by the interviewer:

1) Concomitant Medications
2) Medical Event Form, if applicable
3) Health Changes Questionnaire
4) Time Since Last Cigarette Questionnaire

The following assessments will be completed by the participant directly in REDCap:

1) BDI
2) OASIS
3) COVID19 Symptom Questionnaire
4) Respiratory Symptom Questionnaire
5) MNWS

In the event that the REDCap website is not functioning, the assessments will be administered aloud and participant answers will be recorded securely. The interviewer will enter the data into REDCap when it resumes functioning properly. This information should be recorded in the 'End of Visit Evaluation Form'.

7.3. Experimental Visits Weeks 2, 4, 6, 8, 10, 12, 14, and 16 Procedures:

7.3.A Measures/Assessments

Physiological measures collected and entered directly into REDCap by interviewer:

1) CO
2) Blood Pressure
3) Heart Rate
4) Body Temperature
5) Oxygen Saturation
6) Respiratory rate
7) Urine or Saliva Toxicology
8) Urine Pregnancy test (if applicable)

The following assessments will be administered as an interview and will be entered into REDCap by the interviewer at the end of the visit:

1) Concomitant Medications

2) Medical Event Form, if applicable
3) Health Changes Questionnaire
4) Time Since Last Cigarette Questionnaire

The following assessments will be completed by the participant directly in REDCap:

1) BDI
2) OASIS
3) COVID19 Symptom Questionnaire
4) Respiratory Symptom Questionnaire
5) MNWS
6) QSU (usual brand)
7) QSU (study cigarette)
8) Vaping Craving Questionnaire
9) CES (usual brand)
10) CES (study cigarette)
11) Vaping Evaluation Scale
12) PANAS
13) Cigarette Purchase Task - Usual Brand Cigarette Version (weeks 4, 8, 12 and 16 only)
14) Cigarette Purchase Task – Study Cigarette Version (weeks 4, 8, 12 and 16 only)
15) Cross-price Elasticity Task[84]- e-cigarettes and combustible cigarettes (weeks 4, 8, 12 and 16 only) (for e-cigarette experimental conditions only)
16) Penn State Electronic Cigarette Dependence Index (weeks 8 and 16 only)
17) Respiratory Health Questionnaire (weeks 8 and 16 only)
18) FTND (weeks 8 and 16 only)
19) Perceived Health Risks Questionnaire (weeks 8 and 16 only)
20) Smoking Stages of Change Algorithm and Contemplation Ladder (weeks 8 and 16 only)
21) WISDM – Brief Scale
22) Drug Use Questionnaire – 1 month version (weeks 8 and 16 only)
23) Perceived Stress Scale (weeks 8 and 16 only)
24) Alcohol Use Questionnaire – 1 month version (weeks 8 and 16 only)
25) E-cigarette flavor rating questionnaire (weeks 4, 8, 12 and 16 only)
    a. Participants will rate only of the flavors that they have been using over the past week and that have been dispensed by study staff.

In the event that the REDCap website is not functioning, the assessments will be administered aloud and participant answers will be recorded securely. The interviewer will enter the data into REDCap when it resumes functioning properly. This information should be recorded in the ‘End of Visit Evaluation Form’.

Biological Samples to be collected:

1) First void urine sample (Weeks 8 and 16 only)

7.4. Interactive Voice Response System:

Participants will continue to use the IVR system on a daily basis throughout the experimental period to record the number of study cigarettes smoked per day, measurement of e-cig use and use of non-study cigarettes or other tobacco products. Measurement of e- cig use will be collected by asking two questions: how many daily e-cigarette episodes occurred, where one episode consists of around 10-15 puffs or up to approximately 10 minutes, and what proportion of pods and/or cartridges were used per day. Participants will also be asked to log how many flavors of e-cigs they used per day. During the first week after Baseline 1, the IVR system will collect information about mood and withdrawal symptoms.

7.5. Variable Incentive Program:

An incentive program has been developed with the goal of improving attendance at scheduled assessment sessions, compliance with using only study-provided tobacco products, and encouraging honest self-reports regarding all nicotine/tobacco use.

Briefly, participants will receive a total of seven tickets for each weekly visit they attend after randomization (Visits 03-18, week s 1-16). In total, participants could earn 112 valid tickets across the 16 visits. Participants will be instructed that these tickets correspond to attendance (one ticket), honest reporting (one ticket), compliance in bringing back used and unused pods/cartridges (two tickets) and adherence to using only the assigned study product (three tickets). Participants who do not bring back all of their unused study product and used packaging will be told that they may not be eligible to earn the two compliance tickets. Participants will be further instructed that all of the tickets that they receive “could” be eligible for entry into a monthly drawing for prizes, but that only tickets that are “validated” will be eligible for prizes.

Since it is prohibitively expensive to test urine samples each week for each participant and because it is currently not feasible to detect with reasonable precision non- compliance based on biomarkers in the two higher nicotine group, we plan to only validate the attendance tickets. Hence, each participant who attends their regularly scheduled weekly session will have a total of seven validated tickets entered into the monthly drawing.

To convey the message that we may be validating honest reporting and use of only study-provided products, in a bogus pipeline of sorts, we will tell the participants that a composite assessment of the measures that we collect MAY be used to validate the amount of nicotine and tobacco products that they are using. So there is some minor deception involved, but technically we could conduct urine toxicology testing for both purposes. Hence, if the urine toxicology testing is presented as something that MAY be done for validation purposes, we feel that any deception is relatively minor. For scientific/economic reasons we are just electing to restrict validation to attendance. Nevertheless, we will debrief all participants upon the completion of the trial. We will inform them that the incentive program was based exclusively on attendance due to the relatively high cost of urine toxicology testing and other practical problems with shipping the urines for prompt testing.

Drawings will be conducted on the 1st of each month. Validation will be performed by staff who have no participant interaction and are not blind to condition. Any ticket drawn will be eligible for an incentive as the only true contingency is for attendance. There will be no mention of the basis for earning incentives (i.e., whether the ticket was for attendance, honesty, adherence). Participants will simply be informed that he or she earned an incentive from the drawing.

Each drawing will be independent (without replacement); consequently, some participants will not win a prize and others may win more than one during the study if more than one of their tickets is drawn. After confirming winners, the remaining tickets from each month will be discarded (i.e., tickets will only be entered into one drawing). The monthly prize amounts are detailed below.

We estimate based on the 2 ½ years we think it will take to complete this study, that participants will win an average of approximately $65 in prizes or an additional $5.50 per week per participant.

Grand Prize (1): $500 cash
Second Prize (1): $200 cash
Third Prize (5): $10 cash

7.6. Product and Procedures Compliance Review Sessions:

At each visit, Baseline 2 through Week 16, participants will be counseled about their use of the study cigarettes and assigned e-cigarette (if applicable). Participants will be asked about any concerns or obstacles associated with use of the study cigarettes and assigned e- cigarette (if applicable). The importance of honest self-reporting will be stressed. Participants will be told that they will not be penalized for use of other nicotine or tobacco products and that it is crucial for them to report any use of these products. If difficulties are encountered, participants will be

asked why they think they are experiencing difficulties (e.g., taste, withdrawal symptoms) and to problem-solve how to deal with these difficulties in order to meet the protocol requirements. Additionally, participants will be counseled about their IVR completion, visit attendance, task engagement and product accountability. Refer to the '*Product and Procedures Compliance Review Sessions SOP*' for more information.

7.7. Quit Attempts During the Study Protocol:

At each weekly session, we will ask each participant if s/he is currently abstaining from smoking with the intention of quitting and whether s/he is planning to quit smoking prior to his/her next scheduled visit. If a participant is currently abstaining from smoking with the intention to quit, we will encourage the participant to continue abstaining, schedule them for weekly visits , and provide them with NCI's Clearing the Air manual and local smoking cessation resources. We will give them the option of taking study product(s) home but not require that they take them, and if they do take the product(s) home we will suggest that they put the product(s) away at home so as to remove these cues from view. We will ask the participant to contact staff if they lapse and would like to receive study product(s) prior to his/her next visit. If a participant is planning to quit but has not initiated a quit attempt, we will ask if s/he has identified a quit date and if so what the date is, provide them with the Clearing the Air manual and local smoking cessation resources, provide them with the study product(s), and recommend that they put the product(s) away out of view on the quit date.

For those in a condition including e-cigarettes, we will defer to the participant's interests in continuing to use e-cigarettes as part of their quit attempt. Those who indicate that they will continue to use them will be given their same weekly supplies base on their baseline smoking rate. Those who indicate that they are planning to abstain from both combusted and non-combusted tobacco, we will honor that request. As we state above about combusted cigarettes, if participant changes his or her mind about resuming e- cigarette use, they can contact us and obtain their weekly supply.

7.7.A. If a participant is currently abstaining from smoking with the intention to quit:

- Encourage participant to continue abstaining from smoking
- Schedule the participant for normal weekly visits
- Provide the participant with the '*Clearing the Air*' manual and local smoking cessation resources
- Give the participant the option to receive product rather than require him/her to take the product
- If the participant choses to receive the study product have him/her sign a form acknowledging that cigarette availability could be detrimental to the quit attempt. Recommend that he/she put the product "away" at home as to avoid unwanted cues to smoke.
- If the participant chooses not to receive the study product, have him/her contact the lab if he/she lapses and would like to pick up or be mailed the study product prior to his/her next visit.

7.7.B. If a participant is planning to quit smoking, but has not initiated the quit attempt:

- Ask if he/she has identified a target quit date and, if so, what that target date is
- Provide the participant with the '*Clearing the Air*' manual and local smoking cessation resources
- Provide the participant with the study product as usual. Recommend that on the target date he/she put the product "away" at home as to avoid unwanted cues to smoke.

7.8 Abstinence Assessment Session:

After the week 16 visit, participants will be required to attend one additional visit the following day. During this visit, participants will have been encouraged to abstain from smoking until their next scheduled visit (approximately 24 hours later). The abstinence assessment session should be

scheduled no less than 18 hours and no more than 30 hours after the Week 16 visit. Abstinence will be verified by an expired breath carbon monoxide level of 6 parts per million (ppm) or under. This session will allow us to determine whether the experimental cigarettes and e-cigarette use (for the e-cigarette conditions) have reduced the effects of abstinence on these measures relative to the control conditions. If the participant does NOT meet abstinence criteria, he/she will only receive $20 for the visit.

7.8.A Participants Who Meet Criteria for Abstinence

7.8.A.1 Measures/Assessments

Physiological measures collected and entered directly into REDCap by the interviewer:

1) CO
2) Blood Pressure
3) Heart Rate
4) Body temperature
5) Oxygen saturation
6) Respiratory rate
7) Urine or Saliva Toxicology

The following assessments will be administered as an interview and entered directly into REDCap by the interviewer:

1) Concomitant Medications
2) Medical Event Form, if applicable
3) Health Changes Questionnaire
4) Time Since Last Cigarette Questionnaire

The following assessments will be completed by the participant directly in REDCap:

1) BDI
2) OASIS
3) COVID19 Symptom Questionnaire
4) Respiratory Symptom Questionnaire
5) MNWS
6) PANAS
7) QSU-brief - Usual Cigarette
8) QSU-brief - Study Cigarette
9) Vaping Craving Questionnaire
10) Cigarette Purchase Task - Usual Brand Cigarette Version
11) Cigarette Purchase Task - Study Cigarette Version
12) E-cigarette Purchase Task- E-cigarette Version

In the event that the REDCap website is not functioning, the assessments will be administered aloud and participant answers will be recorded securely. The interviewer will enter the data into REDCap when it resumes functioning properly. This information should be recorded in the 'End of Visit Evaluation Form'.

7.8.B. Participants Who Do Not Meet Criteria for Abstinence

7.8.B.1 Measures/Assessments

Participants who do NOT meet abstinence criteria will be required to complete the following assessments:

1) CO
2) Blood Pressure
3) Heart Rate
4) Body temperature
5) Oxygen saturation
6) Respiratory rate
7) Urine or SalivaToxicology

The following assessments will be administered as an interview and entered directly into REDCap by the interviewer:

1) Concomitant Medications
2) Health Changes Questionnaire
3) Medical Event Form, if applicable
4) TLFB

The following assessments will be completed by the participant directly in REDCap:

1) BDI
2) OASIS
3) COVID19 Symptom Questionnaire
4) Respiratory Symptom Questionnaire

7.9 Participant Compensation:

Participants will receive $25 plus a $25 bonus for completing each screening visit on time as scheduled. Payment for the first screening session will be made upon its completion. Payment for the second screening session will be made regardless of enrollment as long as the participant passes the drug test and meets the minimum requirements for carbon monoxide or urinary cotinine levels. Participants who do not pass the drug test or who are visibly intoxicated as determined by slurred speech, swaying, or stumbling will not be able to complete the visit and will be asked to take another test several days after the first positive. If they are negative for the second test, they will be eligible to participate, and if they are positive the for the second text they will be excluded. Participants will receive $100 for each study visit from Baseline 1 to Week 16. Participants will also have a chance to earn an additional $20 bonus for every study visit that is completed on time as scheduled starting at Week 1 and ending at Week 15. Participants can receive up to $120.00 for the abstinence session ($20 if participant does not achieve abstinence, $120 if participant reaches abstinence), $40 for biochemical verification of abstinence at 30 day follow up visit, and up to $306 for completing daily IVR reports of study cigarette and other nicotine and tobacco use. There will also be a $150 bonus distributed at Week 16 for completing the study.

Participants who do not complete the entire study will receive compensation for the sessions that they do complete. Total compensation for completing Study 3, including study visit payments, daily IVR calls and end of study bonus is $2816. As mentioned above, participants will have a chance to earn additional incentives for compliance, honesty and attendance through urine testing. Participants will be given a debit card at the beginning of the study (during the second portion of the screening visit) and compensation for each visit will be automatically transferred to the card after they complete that visit. If debit cards are unavailable, participants will be paid via an alternate method (i.e. cash or check).

7.10 End of Study:

After a participant has completed all study procedures and has been paid for participation the research assistant will read the following script and give the participant the Clearing the Air Manual.

"If you've reduced your smoking during this study, we encourage you to continue these reductions or even consider quitting. We would like to provide you with some resources should you decide to try to abstain from smoking (give "Clearing the Air" and hotline information). Please also feel free to consult with your physician and use any medications he/she deems appropriate. We will call you in approximately 30 days to ask about your smoking since leaving the study. There is no right answer and we know how difficult quitting can be. Please just answer honestly. The call will take less than 5 minutes. Thanks again for your participation."

The following assessments will be administered using REDCap:

1) <u>End of Study Questionnaire</u>

7.11 30 Day Follow up Phone Call:

Participants will receive a follow-up phone call or text between 25 and 35 days after the abstinence assessment session to assess their smoking patterns. The phone questionnaire will last less than five minutes. The questionnaire will ask if the participant is still smoking, how much and whether he/she has attempted to quit smoking since the end of the study.

Participants will receive 5 variable incentive program lottery tickets for completing the call as compensation. Those who report abstinence will be invited to complete biochemical verification and be compensated $40 for doing so. Abstinence will be achieved by a carbon monoxide reading of 6 parts per million (ppm) or under. A urine sample will may also be collected to be sent to the lab for analysis. Additionally, any Medical Event Forms that remain open from the last session will be discussed. If the participant became pregnant during the study, this would have been recorded as a medical event. During this phone call, the research assistant will confirm her due date. This event will remain open until delivery. At that time the licensed medical professional will contact the participant to ask a few questions about the baby's health and will update the Medical Event Form.

Once a participant has completed all study procedures and all open events have been closed, the PI or Project Manager will review the participant's record and sign a form indicating study completion for that participant.

8.0 Study Randomization

8.1 Randomization Process

The lead statistician will create a randomization schedule for each of the two sites, amounting to 150% of expected enrollment at each site. The excess randomization codes will be used in the event that a site will have to enroll extra participants due to unexpectedly slow enrollment at another site. The nicotine doses will be identified by letter code and only Administrative Core personnel with no participant contact will have the link between the statistician's letter code and dose assignments. There will be no blinding of e-cigarette conditions. The Administrative Core will maintain the randomization schedule and the link between the alphabetic code and treatment assignment securely. A second sealed copy will be secured in a separate building to protect against loss related to fire or other unforeseen events.

The University of Vermont will be responsible for removing all identifying information from cigarettes received from the Research Triangle Institute (RTI), labeling each carton with a blind code, assigning product using this blind code based on the randomization schedule being provided by the UVM Biostatistics Core, and shipping cigarettes and e- cigarettes to each site as needed based on recruitment. Each site will be responsible for tracking product received and distributed to participants, collecting unused product from participants, and returning unused cigarettes and e-cigarettes to UVM. The participants, investigators and study staff will not have knowledge of which product is given to a participant or whether different participants received the same or different product.

8.2 Study Product Administration

During the experimental period, participants will be provided with a 14-day supply of research

cigarettes equivalent to 150% of their daily smoking rate. Those in the e-cigarette conditions will also be provided with a 14-day supply of e-cigarettes equivalent to their daily smoking rate. This rate will be calculated at Baseline 2 and will be an average daily smoking rate based on the IVR data that reports on the usage for the first seven days following the day of the first baseline visit. This will ensure adequate availability of cigarettes in the numerous locations participants may typically keep a supply (home, work, vehicle, etc.) as well as avoid expending the entire supply if they miss a scheduled visit. Participants will be instructed to use the research cigarettes for 16 weeks, at which point they are to discontinue product use.

If there is prior knowledge a participant will be missing a visit (i.e. planned vacation, laboratory closure, etc.), then the participant will be provided with an adequate supply of cigarettes to make up for the missed visit(s). The participant will be given a 21-day supply if one visit is going to be missed and a 28-day supply if two visits are going to be missed.

8.3 Guidelines for Reporting other Nicotine Product Use

Participants will be asked to refrain from use of other non-study cigarettes during the study period. If participants have to use another nicotine product, they will be told to use a non-combustible product (gum, patch, etc.). Additionally, they will be told there is not a penalty for use of non-study products, and that it is crucial for them to report any use of non- study tobacco products. Throughout the baseline and experimental periods, an Interactive Voice Response (IVR) system will be used on a daily basis to record the number of study cigarettes and non-study cigarettes used the previous day. During the baseline and first experimental week, participants will also answer daily IVR questions about their mood. Participants will be seen weekly for assessments. Brief standardized review sessions focusing on compliance with the study cigarettes and other study procedures will be provided at each visit.

8.4 Product Accountability:

Participants will be required to keep track of all the products provided to them. Therefore, they will be instructed to return all unused products and empty cigarette packs e- liquid pods/cartridges to the laboratory each week. Research staff will complete the ‘Product Accountability Log’ as they process participants’ product. Any discrepancies in the product dispensed versus product returned will be discussed and recorded in the log. Research staff will weigh all opened e-cigarette pods/cartridges that the participant returns at all visits to determine how much e-liquid was used since the participant was last seen. Empty cigarette packs and e-liquid pods/cartridges will not be saved. Unused cigarette packs and e-liquid pods/cartridges will be re-distributed to the participants during Weeks 1-15. During Week 16, remaining unused cigarettes and e-cigarette pods/cartridges returned by the participants will be collected by the research staff.

Participants who report running out of cigarettes or e-liquid pods/cartridges prior to a scheduled weekly visit will be allowed to come in for an unscheduled visit to obtain more research cigarettes. To determine whether a rate change for cigarettes is necessary, we will look at the past two CO levels as compared to the Baseline 2 CO. If the CO trend is consistent with the self-report of smoking all of the allotted cigarettes then a rate increase will be granted. The participant will then receive cigarettes at a rate of 175% of their daily smoking rate. The maximum increase is 200% of their daily smoking rate. To determine whether a rate change for e-cigarettes is necessary, we will monitor the amount of e- cigarette use the participant is reporting and showing through product return along with any unscheduled visits. The investigator may grant an e-cigarette rate increase in increments of 25%. The maximum increase for e-cigarettes will be 200% of their baseline weekly e- cigarette dispensation rate.

If participants lose more than two packs of cigarettes and/or pods/cartridges and require an unscheduled visit to the laboratory to supplement their supply, they will be told the next time they lose more than two packs they will have to wait until their next scheduled appointment to receive more cigarettes.

9. Study 3 Statistical Methods and Sample Size

9.1 Statistical Methods

Continuous outcomes will be summarized by mean, standard deviation, median and range. Categorical outcomes will be summarized by frequencies and percentages. Skewed continuous outcomes will be log- or square-root transformed as appropriate. Variables measured at each baseline visit will be averaged and the average will be used as the baseline measurement. As we expect conditions to be balanced on important baseline characteristics due to randomization, our primary analysis for all endpoints will not be adjusted for potential confounders. However, a secondary analysis will be completed for all outcomes adjusting for demographic characteristics (e.g., age) that we have found to be important in prior studies. Potential moderators (e.g., SSRI vs. non-SSRI antidepressant, depression vs. anxiety disorders, BMI above or below 30) will be explored by adding that term and the moderator-by-condition term to the model. We will examine age group and gender as potential moderators in a similar fashion.

Participants will be randomized in equal probability to one of the four conditions, with randomization stratified by site and menthol cigarette status. All analyses will follow the intent-to-treat principle (i.e. subjects will be analyzed according to condition assignment, regardless of compliance). The Primary Aim will examine the effects of condition on total CPD (study product and non-study product). CPD will be analyzed by week (mean over all days in a seven-day period) using a mixed model to account for repeated measures from the same individual. Models will include baseline CPD as a covariate. Using a mixed model also allows us to include the effect of study site as a random effect. Additional analyses conducted using data collected at the end of the study will use orthogonal comparisons to test for a linear trend in the decrease in CPD, such that VLNCC + PF e-cig > VLNCC + TO e-cig > VLNCC > NNCC, with the largest reduction in the VLNCC + PF e-cig condition. As we expect that differences among conditions for some of the outcomes may not follow a linear pattern, we will use related planned comparisons to test for threshold effects, specifically contrasting the NNCC condition to all three VLNCC conditions, and NNCC to the two VLNC + e-cigarette conditions. Analysis of cigarette demand, smoke exposure and tobacco carcinogens (Aim 2) as well as additional outcomes, including subjective effects, will be analyzed in a similar manner. Because Exploratory Aim 3 is based on abstinence- induced effects and will be examined using data collected at a single visit at Week 17, analysis will be based on an analysis of co-variance model. In addition to the effect of condition, we will include important covariates noted above. Exploratory analyses will also be conducted combining data collected from three of the vulnerable populations (disadvantaged women of childbearing age, opioid dependent individuals, individuals with AD) to explore potential differences in effects of study condition across these populations.

This will be done with the addition of the effects of population and population-by-condition terms to the models described above. Study staff will make every effort to minimize missing data, and results of our ongoing trial suggest that this will be minimal. We will examine the missing data pattern, and if it is missing at random, will use all data available, without imputation.

9.2 Sample Size

Sample size was determined using NQuery Advisor based on hypothesis tests related to Aim 1, specifically to detect a significant difference between the study conditions (NNCC, VLNCC, VLNCC + TO e-cigs, VLNCC + PF e-cigs) on total CPD. The primary statistical approach will be repeated measures ANOVAs but required sample sizes were calculated focusing on expected outcomes at Week 16. This sample size estimate is intentionally conservative and calculated based on one outcome at one time point; however, given the repeated measures nature of our data, we will have correlated observations within subjects. Thus, with the given sample sizes we will achieve the stated power to detect differences of even lesser magnitude than stated or planned. Our sample size determination is based on preliminary results from our current trial

Table 2. Observed effect sizes

Outcome	Observed between-group ES (15.8 vs 0.4 mg/g)
CPD	0.70
Craving	0.40
FTND	0.12
Breath CO	0.38

and results from the Donny et al. study[98] of VLNC and NNC cigarettes. Note that the between-group effect size is defined as the difference of study condition means divided by the common standard deviation. A sample size of 53 participants per condition will provide 80% power to detect an effect size of 0.60 for all pair-wise comparisons, with a two-sided type 1 error rate of 0.05. This is smaller than that found in our on-going study to date for CPD and smaller than the effect sizes reported by Hatsukami et al. 2010[38] for all measures except breath CO. In addition, this sample size provides greater than 95% power to detect a linear dose-response effect across the four experimental conditions. Because we have relied only on outcomes at Week 16, our proposed sample sizes are somewhat conservative, but we believe this is appropriate for this study given that the effects of VLNC cigarettes in this population, particularly in combination with e-cigarettes, are completely unknown. The sample size above assumes a 15% loss to follow-up, consistent with our experience in the current study.

10. Potential Risks of Participation

10.1 Risks of Participation

1) Survey Questionnaires. Surveys include questions about participants' medical and psychiatric histories, drug and alcohol use and history, breath tests for cigarette and alcohol use, urine or salivary tests of illicit drug use and pregnancy, and questionnaires about mood. Answering these personal questions can make participants uncomfortable. If a participant reports thoughts of killing themselves or other indicators of suicidality, a study clinician will come to talk to the participant. The participant may also request to see a study clinician if he or she is in discomfort and would like help and/or referrals for mental health resources.
2) Breach of Confidentiality: The risk of the interview is loss of privacy if other people find out the results.
3) Undue Influence: Undue influence is a possible risk due to monetary compensation for participating in these studies. The likelihood of this risk is low because the compensation is commensurate with the amount of time and effort required for these studies.
4) Drug Testing: A breach of confidentiality could occur and other people could learn of the participant's drug use.

5) Obtaining Blood Pressure and Heart Rate. The blood pressure cuff may cause minimal discomfort. In obtaining blood pressure we may find a participant to have abnormal blood pressure and/or heart rate. If participant's blood pressure is abnormal, we will inform the participant of this, and participant may be advised to see a doctor, and may also be contacted by our study doctor. Also, smoking and nicotine can affect the cardiovascular system, which may result in changes in blood pressure and/or heart rate.
6) Smoking Cigarettes: All cigarettes are detrimental to a person's health and can lead to significant medical problems including:
    a. Cardiovascular Diseases: Coronary heart disease, heart attack, stroke, peripheral vascular disease, reduced blood circulation, abdominal aortic aneurysm
    b. Respiratory Diseases: Emphysema, bronchitis, and chronic airway obstruction
    c. Cancers: Cancer of the lung, bladder, cervix, esophagus, kidney, larynx, mouth, pancreas, throat, and stomach; leukemia
    d. Metabolic Diseases: Type 2 Diabetes
    e. Other Health Risks Associated with Smoking: Including but not limited to infertility, lower bone density in postmenopausal women, and hip fracture in women
    f. Death
7) Smoking Study Cigarettes. All cigarettes are harmful to a person's health and can lead

to cardiovascular (heart) disease, respiratory (lung) disease, cancer and other health problems. In addition to the above medical problems, participants may experience some minor negative health effects such as headaches. Participants may also experience smoking withdrawal symptoms, which are listed below. In addition, due to the altered nicotine levels, there could be a change in a participant's use of cigarettes including the manner in which he or she inhales the smoke. Smoking the study cigarette does not necessarily provide any less risk than the participant's usual brand of cigarette and could pose increased health risks.

8) Using Study E-cigarettes. E-cigarettes are devices that heat nicotine to produce an aerosol. The health effects of e-cigarettes are still unclear, but appear to be less than that for tobacco cigarettes. Most e-cigarette users have lower nicotine levels than when they smoked regular cigarettes. Some e-cigarette users, especially those who use both e-cigarettes and regular tobacco cigarettes as well as youth and young adults, can have increased nicotine levels. In some rare cases, these use patterns have been associated with seizures. Whether this would occur with the concurrent use of very low nicotine cigarettes is unclear. E-cigarettes users very often maintain addiction to nicotine, but this addiction appears to be somewhat less than that from tobacco cigarettes. Abruptly quitting e-cigarettes could cause withdrawal symptoms similar to those from quitting tobacco cigarettes (see below) but slightly less severe. The most common side effects include dry mouth, irritation of the throat and mouth, and mild cough. The JUUL and Vuse e-cigarettes we will be providing have not been well-studied but appear to be of similar risk to other e-cigarettes. Participants may have heard that e-cigarettes, or "vapes," can explode and seriously injure people. Study staff should instruct participants that although they appear rare, these explosions are dangerous. The exact causes of these incidents are not yet clear, but some evidence suggests that battery-related issues may lead to vape explosions. In order to prevent e-cigarette related injuries, instruct participants to keep their e-cig away from other metal objects, never charge the e-cig with a phone or tablet charger, don't charge the e-cig overnight or leave it charging unattended, and to stop using the e-cig if the batteries get damaged or wet. The participant will be instructed to always keep e-cig liquid out of kids' and pets' reach and sight after use. If we study staff learns about additional risks of e-cigarettes during the study, participants will be informed of these risks.

9) Mood and Psychiatric Symptom Changes. Participants may experience smoking withdrawal symptoms during this study. These symptoms can include anger, anxiousness, craving for a cigarette, depressed mood, difficulty concentrating, frustration, increased appetite, impatience/impulsivity, irritability, restlessness, sleep problems, and weight gain. These feelings can be uncomfortable and can last a couple of weeks, but usually are of minimal risk. In addition, if participants have a past history of anxiety, depression, or alcoholism, it is possible withdrawal could cause substantial increases in depression and anxiety symptoms, but this appears to be rare. At each visit, study staff will ask participants how they feel. If either study staff or the participant thinks that being in this study is putting the participant's mental health at risk, staff may have the participant meet with an on-site clinician and/or stop participating in the study. Further, if a participant report thoughts of killing oneself or other indicators of suicidality, a study clinician will come to talk to the participant. Participants may also request to see a study clinician if he or she is in discomfort and would like help and/or referrals for mental health resources.

10) Returning to Regular Smoking: It is possible that if participants return to smoking their usual brand of cigarette at the end of the study they may experience mild and transient nausea, dizziness, and lightheadedness.

11) Risk to Fetus. To avoid risks to a fetus, it is important that participants are not pregnant during this study. Avoiding sexual activity is the only certain method to prevent pregnancy. However, if participants choose to be sexually active, participants should be using approved forms of birth control if applicable determined by the Project Medical Director. including but not limited to prescribed birth control pills, patch, ring, injections, implants or intrauterine device (IUD) or an appropriate “double barrier” method. If you choose to be sexually active during this study, pregnancy could still result even with the use of these birth control methods.

10.2 Avoiding Risks to Fetus

If participants choose to be sexually active, they should use an appropriate “double barrier” method of birth control (such as female use of a diaphragm, or contraceptive sponge, in addition to male use of a condom) or the female should be using prescribed “birth control” pills, patch, ring, injections, implants or intrauterine device (IUD) if applicable determined by the Project Medical Director. If a participant endorses a “double barrier” method, our medical professional will speak to the participant to confirm which methods will be used during the duration of the study. Participants will be tested for pregnancy every two weeks beginning at screening through the last study visit. If a participant becomes pregnant during the study, she will be withdrawn from the study. Approximately 30 days after being withdrawn or having a positive pregnancy test at the last study visit, the research staff will call the participant to confirm her due date. The licensed medical professional will follow-up with the participant after delivery to ask questions about the baby’s health.

10.3 Expected benefits of participation

There are no benefits from participating in the study. The information obtained from this study may ultimately help the Food and Drug Administration decide how best to regulate nicotine and tobacco products with the goal of improving public health.

11. Protection Against Risk

11.1 Data Collection Protections

Research data without identifiers will be maintained in a locked file cabinet and on password-protected computers in the research staff workplace, with only code numbers identifying subjects. Study consent forms and the linkage between the participants’ names and codes will be stored in a locked file cabinet inside a locked office. Interviews with participants will be conducted in private rooms. Urine or saliva samples for drug and pregnancy tests and tobacco exposure biomarkers will be obtained in a private bathroom within the laboratory suite. Subjective measures will be administered electronically. The biostatistics and data-management team will provide consistent data-management practices for all data in the Center. Using REDCap, which is housed on the University of Vermont Medical Center’s HIPAA-compliant computing system, will maximize validity and reliability of data. REDCap is a secure, web-based system that accommodates local and remote data collection by each project team, and allows for data entry work-flow monitoring and data quality control monitoring by biometry staff. The RedCap database for this project will be hosted on the UVMCOM servers. In addition, data will be collected from participants on a daily basis using an interactive voice recognition system (IVR) developed and hosted by TeleSage Inc (www.telesage.com, Chapel Hill, NC). TeleSage is a company with expertise on gathering patient-centered outcomes tracking data for mental health clinical and research institutions. TeleSage has developed and hosted behavioral health-related research software systems using IVR and Web-based technologies and is leader in behavioral health outcomes tracking technologies. For data integrity, data entry windows will follow the structure of paper forms as much as possible to allow for ease of entry, and will use predefined choices to minimize errors when possible. Data quality monitoring will be facilitated with periodic down loads and analysis using a variety of common statistical program format such as SAS, Stata, R, and SPSS. Quality control

procedures will be conducted for all data collected, including analysis of missing data and logic checks for out of range and other anomalous values. This secure electronic data gathering and transmission plan, overseen by the experienced biostatistical team, will minimize opportunities for breaches of confidentiality. Biological samples for nicotine and carcinogen biomarker analysis will be marked with participant ID, stored in the locked laboratory suite, and sent to a laboratory for analysis on a quarterly basis.

All information collected as part of this study will be accessible only to research staff. No information will be shared with participants' clinicians unless the participant requests this in writing. All investigators and staff have undergone (and any new staff will undergo) human subjects' ethics training as required by UVM and are fully conversant with relevant ethical principals around confidentiality. Assessments, consenting and study procedures will be closely supervised by the PI.

The sponsors (NIDA/FDA) as well as the Institutional Review Board and regulatory authorities could be granted direct access to original medical and research records for verification of clinical trial procedures and/or data. If this is required, it will be done under conditions that will protect privacy to the fullest extent possible consistent with laws relating to public disclosure of information and the law-enforcement responsibilities of the agency.

11.2 Data Storage:

Data will be stored locally at each site. Long-term storage of all study data, for at least 7 years after study completion, will be at the University of Vermont.

12. Adverse Events

The research assistant will ask about adverse events at each session, using a form that assesses the nature, severity, duration, action taken, and outcome of study-related adverse events. AEs will be captured from the time of first study cigarette. Participants will be given contact cards to inform us of events that occur between study contacts. Any AE that remains open will be reviewed and closed at an interview conducted 30 days after the study completion date (completers) or when the study should have ended had the participant completed the study (dropouts and those withdrawn by investigator).

All procedures will be monitored to ensure that they conform to the approved protocol. In addition, monitoring will be done of all unforeseen circumstances that might arise and affect safety; of all reports of serious adverse events as defined in 38 CFR 46 (death, new or prolonged hospitalization, persistent or significant disability/incapacity); of other significant adverse events (adverse events that lead to drop out by the participant or termination by the investigator); of unexpected adverse events resulting from the study, and of expected adverse events.

Any SAE will be brought to the attention of the site PIs as soon as possible and not longer than 24 hours. Any AE or SAE that is both unexpected and related to study participation will be reported to the IRB within 7 days of the event. The local IRB will make a determination as to whether additional reporting requirements are needed. IRB actions will be reported to the funding agency by the PIs no less than annually and more frequently as recommended by the local IRB. Any SAEs will be summarized in the yearly Progress Reports to the funding agency, including a review of frequency and severity. All SAEs will be followed through ongoing consultation with the physician caring for the patient until they resolve, result in death, or stabilize and are not expected to improve. The study staff will be in close contact with participants and health care providers throughout the study to monitor for potential unanticipated problems. Any unanticipated problems will be discussed at the weekly research staff meetings and reported as required to the local IRB.

13. Withdrawal or Monitoring of Participants

For the participant's protection, participants will be withdrawn immediately from the study if any of the following occur:

1) Cardiovascular disease (CVD) event: Typically includes MI (heart attack), PTCA (angioplasty/stenting), bypass surgery, stroke, peripheral vascular disease (arterial blockages in arms or legs leading to procedure or surgery). Less common CVD problems would be new cardiac arrhythmias (e.g., new atrial fibrillation) or new valvular disease (e.g., mitral or aortic regurgitation).

2) DVT/PE (deep vein thrombosis/pulmonary embolism, i.e., blood clots in the venous system).

3) Suicide Attempt: A participant will be withdrawn if he/she attempts suicide at any time during participation in the study.

4) Psychiatric Hospitalization: A participant will be withdrawn if he/she is hospitalized for psychiatric reasons at any time during participation in the study.

5) Pregnancy: If participant indicates she is pregnant or has a positive pregnancy test at any time during the study, she will be withdrawn from the study, and this event will remain open until delivery. At that time the licensed medical professional will contact the participant to ask a few questions about the baby's health and will update the open 'Medical Event Form'. A positive pregnancy test at Week 16 will trigger a 'Medical Event Form' to be completed but will not result in withdrawal since she is no longer receiving study product.

6) Expired breath carbon monoxide increase: A participant will be withdrawn from the study if the average of two consecutive CO readings during the same visit is 100 ppm or greater.

7) Marked increase in smoking: A participant will be withdrawn from the study if he/she meets **BOTH** of the following criteria for two consecutive weeks

    a. Cigarette per day increase: The average CPD increases by more than 100% from the average CPD during baseline.
    b. Expired breath carbon monoxide increase: If the average of two consecutive CO measurements in the same visit is
        i. CO is greater than 50 ppm if CO at Baseline 1 is <20 ppm.
        ii. CO is greater than 60 ppm if CO at Baseline 1 is 20 – 34 ppm.
        iii. CO is greater than 70 ppm if CO at Baseline 1 is 35 – 49 ppm.
        iv. CO is greater than 80 ppm if CO at Baseline 1 is 50 – 64 ppm.
        v. CO is greater than 90 ppm if CO at Baseline 1 is 65 – 80 ppm.
    c. Note: If the second consecutive visit is the last study visit, then the participant would not be withdrawn from the study.

8) If a participant is discharged from or discontinues his or her methadone or buprenorphine treatment, they will be discontinued from the study.

The following will be monitored and can lead to the participant being withdrawn by the PI or Licensed Medical Professional:

1) Cigarettes per day increase: Continued participation will be evaluated by the site PI if the average number of cigarettes per day (CPD) increases by more than 100% from the average CPD during baseline as determined by CPD at Baseline 2.

2) Blood pressure (BP) or heart rate (HR) changes: If any of the following occur post-enrollment: 1) BP is at or above 160/100 or below 90/50, or 2) HR is at or above 115 bpm or below 45 bpm a manual blood pressure and heart rate measurement will be taken after 10 minutes have passed. If the manual reading is still out of range, a 'Blood Pressure and Heart Rate Symptom Checklist' and 'Medical Event Form' will be completed, and the participant will be monitored by the medical professional.

3) Expired breath Carbon Monoxide increase: If the average of two consecutive CO measurements meets the criteria below then the 'Medical Event Form' will be completed and the participant will be monitored by the medical professional.

a. CO is greater than 50 ppm if CO at Baseline 1 is <20 ppm.
b. CO is greater than 60 ppm if CO at Baseline 1 is 20 – 34 ppm.
c. CO is greater than 70 ppm if CO at Baseline 1 is 35 – 49 ppm.
d. CO is greater than 80 ppm if CO at Baseline 1 is 50 – 64 ppm.
e. CO is greater than 90 ppm if CO at Baseline 1 is 65 – 80 ppm.

4) Any hospitalization or debilitation in which participation in the study could be detrimental to the recovery process. This will be self-reported by the participant and will be reviewed by the site PI and medical professional to determine whether continued participation in the study is appropriate.
5) If a participant is behaving in an inappropriate or threatening manner, admits to lying about eligibility criteria, is participating in other smoking research studies that could affect the primary outcome measures, etc., then the PI can withdraw him/her from the study at the PI's discretion.
6) If a participant fails to attend regularly scheduled research assessment visits or comply with the research procedures or schedule, then the PI can withdraw him/her from the study at the PI's discretion.
7) Increase in psychiatric symptoms: Exacerbation in symptoms noted during the study (i.e., change in BDI category from mild to moderate or moderate to severe) will trigger review by the study's licensed medical professional. The PI will withdraw the participant upon the licensed medical professional's recommendation.

14. Data Safety Monitoring Board

A Data and Safety Monitoring Board (DSMB) has been established to monitor safety outcomes and will be comprised of four members. The DSMB will be chaired by Kevin Delucchi, PhD., Professor in Residence of Biostatistics in Psychiatry at the University of California San Francisco and Director of the Quantitative Core of the San Francisco Treatment Research Center; Eden Evins, MD, MPH., Cox Family Professor of Psychiatry at Harvard Medical School and Director of Center for Addiction Medicine at Massachusetts General Hospital; Ari Kirshenbaum, PhD, Professor of Psychology at Saint Michael's College who he teaches courses in psychopharmacology and neuroscience, and currently has grants from NIH and NSF for work in human behavioral pharmacology and his grant-funded work focuses on cognitive and behavioral responses to nicotine and cannabinoids; and Elisabeth Johnson, Ph.D., who has over twenty years of clinical experience in women's health and pediatrics, including caring for women with substance use disorders.

Conflict of Interest

None of the board members will be otherwise affiliated with the center and each member will complete a conflict of interest disclosure form prior to each meeting. Ad hoc specialists may be invited to participate as non-voting members at any time if additional expertise is desired.

Monitoring Activities and Frequency of Meetings

The DSMB will set their own agenda and decisions about monitoring; e.g. how frequently to monitor, what threshold requires changes to protocol or stopping the study, and whether to view raw or analyzed data. The DSMB will be given FDA and EMEA guidelines for DSMBs and recent reviews on DSMBs. A brief report will be generated from each meeting for the study record and forwarded to each of the study site's Institutional Review Boards (IRB) and NIDA's Program Officer with the progress report. The DSMB will be available to convene outside of the regular meetings, if necessary. If concerns should arise regarding a particular subject, or any troublesome trends in the experiences of participants, they will make appropriate recommendations for changes in protocol, as needed. The project investigators will continue to examine safety data, blind to study condition, in case they wish to make study modifications. Before modifications are made, they will inform the DSMB and request their comments.

Communication Plan to IRB, NIDA, and FDA (if applicable)

All IRBs, the FDA and the NIDA's Program Officer will be informed of any significant action taken as a result of the Data and Monitoring Board's findings. Study Participants will be informed of any changes in risk.

Protection of Confidentiality

For DSMB meetings only de-identified data, including blinded study site and condition type, will be provided to the board. All data and discussion during the meeting will be confidential.

15. Investigational Tobacco Product

The University of Vermont Center on Tobacco Regulatory Science will complete an Investigational Tobacco Product (ITP) application with the FDA to cover the experimental cigarettes being used in this study. This application encompasses both Project 1 sites.

16. Certificate of Confidentiality

To help protect the participant's privacy, Dr. Stephen Higgins, PhD, will obtain a Certificate of Confidentiality from the national Institute on Drug Abuse. With this certificate, the researchers cannot be forced to disclose information that may identify the participants, even by a court subpoena, in any federal, state, or local civil, criminal, administrative, legislative, or other proceedings. The researchers will use the Certificate to resist any demands for information that would identify the participants, except as explained below. The Certificate cannot be used to resist a demand for information from personnel of the United States Government that is used for auditing or evaluation of federally funded projects or for information that must be disclosed in order to meet the requirements of the Federal Food and Drug Administration (FDA).

The Certificate of Confidentiality does not prevent the participant or a member of their family from voluntarily releasing information about themselves and their involvement in the research. If an insurer, employer or other person obtains the participant's written consent to receive research information, then the researcher may not use the Certificate to withhold that information.

The Certificate of Confidentiality does not prevent the researchers from disclosing voluntarily, without consent, information that would identify the individual as a participant of the research project in instances such as evidence of child abuse or a participant's threatened violence to self or others.

17. Outcome Variables

Primary Endpoints for Study 3:

1) Cigarette Smoked per Day (CPD)
2) Nicotine Dependence Severity

Secondary Endpoints for Study 3:

1) Measures of adherence: non-study cigarette use, drop-out rate
2) Measures of psychiatric symptoms: BDI, OASIS
3) Measures of discomfort/dysfunction: MNWS, QSU
4) Measures of other health-related behaviors: breath alcohol, urine or salivary drug screen, TLFB-drug use, Alcohol Use Questionnaire, Drug Use Questionnaire, weight
5) Measures of nicotine/tobacco dependence: FTND, WISDM
6) Measures of tobacco exposure: CO, total nicotine equivalents, NNN, NNAL, minor alkaloids
7) Measures of intention to quit: Stages of Change, Contemplation Ladder
8) Measures of compensatory smoking: puff topography, filter analysis
9) Measures of other tobacco use: TLFB-other tobacco

10) Measures of cigarette characteristics: CES, Cigarette Purchase Task
11) Measures of cardiovascular function: heart rate, blood pressure, urine 11-dehydroTXB2
12) Measures of perceived risk: Perceived Health Risk Questionnaire
13) Safety outcome variables: Adverse Events (AEs), Serious Adverse Events (SAEs)

References

1. Benowitz NL, Henningfield JE. Establishing a nicotine threshold for addiction. The implications for tobacco regulation. N Engl J Med. 1994;331(2):123-125.
2. Tengs TO, Ahmad S, Savage JM, Moore R, Gage E. The AMA proposal to mandate nicotine reduction in cigarettes: a simulation of the population health impacts. Prev Med. 2005;40(2):170-180.
3. Higgins ST, Chilcoat HD. Women and smoking: an interdisciplinary examination of socioeconomic influences. Drug Alcohol Depend. 2009;104 Suppl 1:S1-5.
4. Chilcoat HD. An overview of the emergence of disparities in smoking prevalence, cessation, and adverse consequences among women. Drug Alcohol Depend. 2009;104 Suppl 1:S17-23.
5. Kurti AN, Redner R, Lopez AA, et al. Tobacco and nicotine delivery product use in a national sample of pregnant women. Prev Med. 2017. In press.
6. Lopez AA, Redner R, Kurti AN, et al. Tobacco and nicotine delivery product use in a U.S. national sample of women of reproductive age. In preparation.
7. Higgins ST, Heil SH, Badger GJ, Skelly JM, Solomon LJ, Bernstein IM. Educational disadvantage and cigarette smoking during pregnancy. Drug Alcohol Depend. 2009;104 Suppl 1:S100-105.
8. Higgins ST, Solomon LJ. Some recent developments on financial incentives for smoking cessation among pregnant and newly postpartum women. Curr Addict Rep. 2016;3(1):9-18.
9. Kandel DB, Griesler PC, Schaffran C. Educational attainment and smoking among women: risk factors and consequences for offspring. Drug Alcohol Depend. 2009;104 Suppl 1:S24-33.
10. Vurbic D, Harder VS, Redner RR, Lopez AA, Phillips JK, Higgins ST. Co-occurring obesity and smoking among U.S. women of reproductive age: Associations with educational attainment and health biomarkers and outcomes. Prev Med. 2015;80:60-66.
11. WHO. Tobacco or health: A global status report. Geneva, Switzerland: WHO Press, 1997.
12. WHO. Gender, women, and the tobacco epidemic. Geneva, Switzerland: WHO Press, 2010.
13. Hughes EG, Brennan BG. Does cigarette smoking impair natural or assisted fecundity? Fertil Steril. 1996;66(5):679-689.
14. Sun L, Tan L, Yang F, et al. Meta-analysis suggests that smoking is associated with an increased risk of early natural menopause. Menopause. 2012;19(2):126-132.
15. US Department of Health and Human Services. The Health Consequences of Smoking—50 Years of Progress: A Report of the Surgeon General. Atlanta: U.S. Department of Health and Human Services, Centers for Disease Control and Prevention, National Center for Chronic Disease Prevention and Health Promotion, Office on Smoking and Health, 2014.
16. WHO. Tobacco smoke and involuntary smoking. Lyon, France: WHO Press, 2004.
17. Flower A, Shawe J, Stephenson J, Doyle P. Pregnancy planning, smoking behaviour during pregnancy, and neonatal outcome: UK Millennium Cohort Study. BMC Pregnancy Childbirth. 2013;13:238.
18. Solomon L, Quinn V. Spontaneous quitting: self-initiated smoking cessation in early pregnancy. Nicotine Tob Res. 2004;6 Suppl 2:S203-216.
19. Morasco BJ, Dornelas EA, Fischer EH, Oncken C, Lando HA. Spontaneous smoking cessation during pregnancy among ethnic minority women: a preliminary investigation. Addict Behav. 2006;31(2):203-210.
20. Ananth CV, Smulian JC, Vintzileos AM. Incidence of placental abruption in relation to cigarette smoking and hypertensive disorders during pregnancy: a meta-analysis of observational studies. Obstet Gynecol. 1999;93(4):622-628. Castles A, Adams EK, Melvin CL, Kelsch C, Boulton ML. Effects of smoking during pregnancy. Five meta-analyses. Am J Prev Med. 1999;16(3):208-215.
21. Savitz DA, Murnane P. Behavioral influences on preterm birth: a review. Epidemiology. 2010;21(3):291-299.
22. Bakketeig LS, Jacobsen G, Hoffman HJ, et al. Pre-pregnancy risk factors of small-for-

gestational age births among parous women in Scandinavia. Acta Obstet Gynecol Scand. 1993;72(4):273- 279.
23. Spinillo A, Capuzzo E, Nicola SE, Colonna L, Egbe TO, Zara C. Factors potentiating the smoking- related risk of fetal growth retardation. Br J Obstet Gynaecol. 1994;101(11):954-958.
24. Higgins ST, Heil SH, Sigmon SC, et al. Response to varying the nicotine content of cigarettes in vulnerable populations: an initial experimental examination of acute effects. Psychopharmacology (Berl). 2017;234(1):89-98.
25. Higgins ST, Heil SH, Sigmon SC, Tidey JW, Gaalema DE, Stitzer ML, Durand H, Bunn JY, Priest JS, Arger CA, Miller ME, Bergeria CL, Davis DR, Streck JM, Reed DD, Skelly JM, Tursi L. Addiction potential of reduced nicotine content cigarettes in populations with psychiatric disorders and other vulnerabilities to tobacco addiction. JAMA Psych. In press.
26. United States Department of Health and Human Services. Substance Abuse and Mental Health Services Administration. Center for Behavioral Health Statistics and Quality. National Survey on Drug Use and Health, 2016. 34481-v3. Research Triangle Park, NC: RTI International, 2016-04- 02.
27. Perkins KA, Donny E, Caggiula AR. Sex differences in nicotine effects and self-administration: review of human and animal evidence. Nicotine Tob Res. 1999;1(4):301-315.
28. Dawkins L, Kimber C, Puwanesarasa Y, Soar K. First- versus second-generation electronic cigarettes: predictors of choice and effects on urge to smoke and withdrawal symptoms. Addiction. 2015;110(4):669-677.
29. Farsalinos KE, Spyrou A, Tsimopoulou K, Stefopoulos C, Romagna G, Voudris V. Nicotine absorption from electronic cigarette use: comparison between first and new-generation devices. Sci Rep. 2014;4:4133.
30. Goniewicz ML, Kuma T, Gawron M, Knysak J, Kosmider L. Nicotine levels in electronic cigarettes. Nicotine Tob Res. 2013;15(1):158-166.
31. Talih S, Balhas Z, Eissenberg T, et al. Effects of user puff topography, device voltage, and liquid nicotine concentration on electronic cigarette nicotine yield: measurements and model predictions. Nicotine Tob Res. 2015;17(2):150-157.
32. Trtchounian A, Williams M, Talbot P. Conventional and electronic cigarettes (e-cigarettes) have different smoking characteristics. Nicotine Tob Res. 2010;12(9):905-912.
33. Farsalinos KE, Polosa R. Safety evaluation and risk assessment of electronic cigarettes as tobacco cigarette substitutes: a systematic review. Ther Adv Drug Saf. 2014;5(2):67-86.
34. Rom O, Pecorelli A, Valacchi G, Reznick AZ. Are E-cigarettes a safe and good alternative to cigarette smoking? Ann N Y Acad Sci. 2015;1340:65-74.
35. Hecht SS, Carmella SG, Kotandeniya D, et al. Evaluation of toxicant and carcinogen metabolites in the urine of e-cigarette users versus cigarette smokers. Nicotine Tob Res. 2015;17(6):704-709.
36. Goniewicz ML, Knysak J, Gawron M, et al. Levels of selected carcinogens and toxicants in vapour from electronic cigarettes. Tob Control. 2014;23(2):133-139.
37. Hatsukami DK, Kotlyar M, Hertsgaard LA, et al. Reduced nicotine content cigarettes: effects on toxicant exposure, dependence and cessation. Addiction. 2010;105(2):343-355.
38. Goniewicz ML, Gawron M, Smith DM, Peng M, Jacob P, Benowitz NL. Exposure to Nicotine and Selected Toxicants in Cigarette Smokers Who Switched to Electronic Cigarettes: A Longitudinal Within-Subjects Observational Study. Nicotine Tob Res. 2017;19(2):160-167.
39. Strong DR, Myers M, Linke S, et al. Gender differences influence overweight smokers' experimentation with electronic nicotine delivery systems. Addict Behav. 2015;49:20-25.
40. Grace RC, Kivell BM, Laugesen M. Gender differences in satisfaction ratings for nicotine electronic cigarettes by first-time users. Addict Behav. 2015;50:140-143.
41. Chivers LL, Hand DJ, Priest JS, Higgins ST. E-cigarette use among women of reproductive age: Impulsivity, cigarette smoking status, and other risk factors. Prev Med. 2016;92:126-134.
42. Bullen C, Howe C, Laugesen M, et al. Electronic cigarettes for smoking cessation: a randomised controlled trial. Lancet. 2013;382(9905):1629-1637.
43. Caponnetto P, Auditore R, Russo C, Cappello GC, Polosa R. Impact of an electronic cigarette

on smoking reduction and cessation in schizophrenic smokers: a prospective 12-month pilot study. Int J Environ Res Public Health. 2013;10(2):446-461.

44. Tseng T-Y, Ostroff JS, Campo A, et al. A Randomized Trial Comparing the Effect of Nicotine Versus Placebo Electronic Cigarettes on Smoking Reduction Among Young Adult Smokers. Nicotine Tob Res. 2016;18(10):1937-1943.
45. Walton KM, Abrams DB, Bailey WC, et al. NIH electronic cigarette workshop: developing a research agenda. Nicotine Tob Res. 2015;17(2):259-269.
46. Adriaens K, Van Gucht D, Declerck P, Baeyens F. Effectiveness of the electronic cigarette: An eight-week Flemish study with six-month follow-up on smoking reduction, craving and experienced benefits and complaints. Int J Environ Res Public Health. 2014;11(11):11220-11248.
47. Glasser AM, Collins L, Pearson JL, et al. Overview of Electronic Nicotine Delivery Systems: A Systematic Review. Am J Prev Med. 2017;52(2):e33-e66.
48. Bullen C, McRobbie H, Thornley S, Glover M, Lin R, Laugesen M. Effect of an electronic nicotine delivery device (e cigarette) on desire to smoke and withdrawal, user preferences and nicotine delivery: randomised cross-over trial. Tob Control. 2010;19(2):98-103.
49. Dawkins L, Turner J, Hasna S, Soar K. The electronic cigarette: effects on desire to smoke, withdrawal symptoms and cognition. Addict Behav. 2012;37(8):970-3.
50. Vansickel AR, Weaver MF, Eissenberg T. Clinical laboratory assessment of the abuse liability of an electronic cigarette. Addiction. 2012 Aug;107(8):1493-500.
51. Lechner WV, Meier E, Wiener JL, et al. The comparative efficacy of first- versus second-generation electronic cigarettes in reducing symptoms of nicotine withdrawal. Addiction. 2015;110(5):862-867.
52. Bonhomme MG, Holder-Hayes E, Ambrose BK, et al. Flavoured non-cigarette tobacco product use among US adults: 2013-2014. Tob Control. 2016;25(Suppl 2):ii4-ii13.
53. Villanti AC, Johnson AL, Ambrose BK, et al. Flavored tobacco product use in youth and adults: Findings from the first wave of the PATH study (2013-2014). Am J Prev Med. March 2017.
54. Audrain-McGovern J, Strasser AA, Wileyto EP. The impact of flavoring on the rewarding and reinforcing value of e-cigarettes with nicotine among young adult smokers. Drug Alcohol Depend. 2016;166:263-267.
55. Goldenson NI, Kirkpatrick MG, Barrington-Trimis JL, et al. Effects of sweet flavorings and nicotine on the appeal and sensory properties of e-cigarettes among young adult vapers: Application of a novel methodology. Drug Alcohol Depend. 2016;168:176-180.
56. Berg CJ. Preferred flavors and reasons for e-cigarette use and discontinued use among never, current, and former smokers. Int J Public Health. 2016;61(2):225-236.
57. Kistler CE, Crutchfield TM, Sutfin EL, et al. Consumers' Preferences for Electronic Nicotine Delivery System Product Features: A Structured Content Analysis. Int J Environ Res Public Health. 2017;14(6).
58. Czoli CD, Goniewicz M, Islam T, Kotnowski K, Hammond D. Consumer preferences for electronic cigarettes: results from a discrete choice experiment. Tob Control. 2016;25(e1):e30-36.
59. Pesko MF, Kenkel DS, Wang H, Hughes JM. The effect of potential electronic nicotine delivery system regulations on nicotine product selection. Addiction. 2016;111(4):734-744.
60. Etter J-F. Electronic cigarettes: a survey of users. BMC Public Health. 2010;10:231.
61. Farsalinos KE, Romagna G, Tsiapras D, Kyrzopoulos S, Spyrou A, Voudris V. Impact of flavour variability on electronic cigarette use experience: an internet survey. Int J Environ Res Public Health. 2013;10(12):7272-7282.
62. Yingst JM, Veldheer S, Hrabovsky S, Nichols TT, Wilson SJ, Foulds J. Factors Associated With Electronic Cigarette Users' Device Preferences and Transition From First Generation to Advanced Generation Devices. Nicotine Tob Res. 2015;17(10):1242-1246.
63. Litt MD, Duffy V, Oncken C. Cigarette smoking and electronic cigarette vaping patterns as a function of e-cigarette flavourings. Tob Control. 2016;25(Suppl 2):ii67-ii72.
64. Jorenby DE, Smith SS, Fiore MC, Baker TB. Nicotine levels, withdrawal symptoms, and

smoking reduction success in real world use: A comparison of cigarette smokers and dual users of both cigarettes and E-cigarettes. Drug Alcohol Depend. 2017;170:93-101.

65. Giovino GA, Villanti AC, Mowery PD, et al. Differential trends in cigarette smoking in the USA: is menthol slowing progress? Tob Control. 2015;24(1):28-37.
66. Ashford K, Rayens E, Wiggins AT, Rayens MK, Fallin A, Sayre MM. Advertising exposure and use of e-cigarettes among female current and former tobacco users of childbearing age. Public Health Nurs. May 2017.
67. Beck AT, Steer RA, Brown GK. Manual for Beck Depression Inventory – II 1996; San Antonio, TX: Psychological Corporation.
68. Norman SB, Cissell SH, Means-Christensen AJ, Stein MB. Development and validation of an Overall Anxiety Severity And Impairment Scale (OASIS). Depress Anxiety. 2006;23(4):245-249.
69. Sheehan DV, Lecrubier Y, Sheehan KH, et al. The Mini-International Neuropsychiatric Interview (M.I.N.I.): the development and validation of a structured diagnostic psychiatric interview for DSM-IV and ICD-10. J Clin Psychiatry. 1998;59 Suppl 20:22-33.
70. Sheehan DV, Lecrubier Y, Sheehan K, Harnett, Janavs J, Weiller E, et al. (1997). The validity of the Mini International Neuropsychiatric Interview (MINI) according to the SCID-P and its reliability. *Eur Psychiat 12*, 232–241.
71. Babor TF, de la Fuente JR, Saunders J, Grant M. AUDIT: The Alcohol Use Disorders Identification Test. Guidelines for use in primary health care. Geneva : World Health Organization, 1992.
72. Skinner HA. The Drug Abuse Screening Test. Addict Behav. 1982; 7(4):363-371.
73. Heatherton TF, Kozlowski LT, Frecker RC, Fagerström KO. The Fagerström Test for Nicotine Dependence: a revision of the Fagerström Tolerance Questionnaire. Br J Addict. 1991;86(9):1119-1127.
74. Piper ME, McCarthy DE, Bolt DM, Smith SS, Lerman C, Benowitz N, et al. Assessing dimensions of nicotine dependence; An evaluation of the Nicotine Dependence Syndrome Scale (NDSS) and the Wisconsin Inventory of Smoking Dependence Motives (WISDM) Nicotine & Tobacco Research. 2008;10:1009-1020.
75. Foulds J, Veldheer S, Yingst J, Wilson SJ, Nichols TT, Eissenberg T. Development of a questionnaire for assessing dependence on electronic cigarettes among a large sample of ex-smoking E-cigarette users. Nicotine Tob Res. 2015; 17(2): 186-92.
76. DiClemente CC, Prochaska JO, Fairhurst SK, Velicer WF, Velasquez MM, Rossi JS. The process of smoking cessation: an analysis of precontemplation, contemplation, and preparation stages of change. J Consult Clin Psychol. 1991;59(2):295-304.
77. Cohen S, Kamarck T, Mermelstein R. A global measure of perceived stress. J Health Soc Behav. 1983;24(4):385-396.
78. Watson D, Clark LA, Tellegen A. Development and validation of brief measures of positive and negative affect: the PANAS scales. J Pers Soc Psychol. 1988;54(6):1063-1070.
79. Hughes JR, Hatsukami D. Signs and symptoms of tobacco withdrawal. Arch Gen Psychiatry. 1986;43(3):289-294.
80. Cox LS, Tiffany ST, Christen AG. Evaluation of the brief questionnaire of smoking urges (QSU- brief) in laboratory and clinical settings. Nicotine Tob Res. 2001;3(1):7-16.
81. Dowd AN, Motschman CA, Tiffany ST. Development and Validation of the Questionnaire of Vaping Craving. Nicotine Tob Res. 2019;12(1):63-70.
82. Cappelleri JC, Bushmakin AG, Baker CL, Merikle E, Olufade AO, Gilbert DG. Confirmatory factor analyses and reliability of the modified cigarette evaluation questionnaire. Addict Behav. 2007;32(5):912-923.
83. Grace RC, Kivel BM, Laugesen M. Estimating cross-price elasticity of e-cigarettes using a simulated demand procedure. Nicotine Tob Res. 2015 May;17(5):592-598.
