# Supplementary material for: Reduced Nicotine Cigarettes and E-Cigarettes in High-Risk Populations: 3 Randomized Clinical Trials
Source: JAMA Netw Open. 2024 Sep 6;7(9):e2431731. doi: 10.1001/jamanetworkopen.2024.31731 (PMC11380105; doi:10.1001/jamanetworkopen.2024.31731)
Supplement: Supplement 2. — eFigure 1. Number of Total Cigarettes Smoked per Day According to Study Population eFigure 2. Biomarkers of Exposure According to Study Population eFigure 3. Dependence Severity According to Study Population eFigure 4. Craving and Withdrawal eFigure 5. Number of Total Cigarettes Smoked per Day Over Time According to Condition, Across Populations, and According to Population eFigure 6. Number of Total Cigarettes Smoked per Day at Week 16 According to Condition, Across Populations, and According to Study Population eFigure 7. Number of Non-Study Cigarettes Smoked per Day According to Condition eFigure 8. Cigarette Abstinence According to Condition eFigure 9. Nicotine Dependence Severity and Cigarette Craving According to Condition eTable 1. Participant Characteristics eTable 2. e-Cigarette Flavors Selected (VLNC+PF Condition) eTable 3. Total Number of Adverse Events eTable 4. Number of Participants With Any Adverse Event eTable 5. Count of Serious and Severe Adverse Events eTable 6. Description of Serious and Severe Adverse Events [file jamanetwopen-e2431731-s002.pdf]

Supplemental Online Content

Higgins ST, Sigmon SC, Tidey JW, et al. Reduced nicotine cigarettes and e-Cigarettes in high-risk populations: 3 randomized clinical trials. *JAMA Netw Open*. 2024;7(9):e2431731. doi:10.1001/jamanetworkopen.2024.31731

eFigure 1. Number of Total Cigarettes Smoked per Day According to Study Population

eFigure 2. Biomarkers of Exposure According to Study Population

eFigure 3. Dependence Severity According to Study Population

eFigure 4. Craving and Withdrawal

eFigure 5. Number of Total Cigarettes Smoked per Day Over Time According to Condition, Across Populations, and According to Population

eFigure 6. Number of Total Cigarettes Smoked per Day at Week 16 According to Condition, Across Populations, and According to Study Population

eFigure 7. Number of Non-Study Cigarettes Smoked per Day According to Condition

eFigure 8. Cigarette Abstinence According to Condition

eFigure 9. Nicotine Dependence Severity and Cigarette Craving According to Condition

eTable 1. Participant Characteristics

eTable 2. e-Cigarette Flavors Selected (VLNC+PF Condition)

eTable 3. Total Number of Adverse Events

eTable 4. Number of Participants With Any Adverse Event

eTable 5. Count of Serious and Severe Adverse Events

eTable 6. Description of Serious and Severe Adverse Events

This supplemental material has been provided by the authors to give readers additional information about their work.

eFigures

Cigarettes Smoked Per Day

There was a significant main effect of study population on week-16 total CPD (Cohen d=0.35; P=.007; eFigure 1A), with LSmean (±SEM) rates lower in the AD than WSD population (11.45±0.78 versus 16.52±1.63) (t[221]=-3.18, P=.005); AD rates did not differ significantly compared to the OUD population nor did the OUD and WSD populations differ significantly. Similarly, there was also a significant main effect of population in the analysis of total CPD across weeks (Cohen d=0.08; P=0.025; eFigure 1B), with LSmean (±SEM) rates lower in the AD than WSD population (12.25±0.61 versus 15.41±1.16) (t[301]=2.65, P=.023); AD did not differ significantly compared to the OUD population nor did the OUD and WSD populations differ significantly.

eFigure 1. Number of Total Cigarettes Smoked Per Day According to Study Population

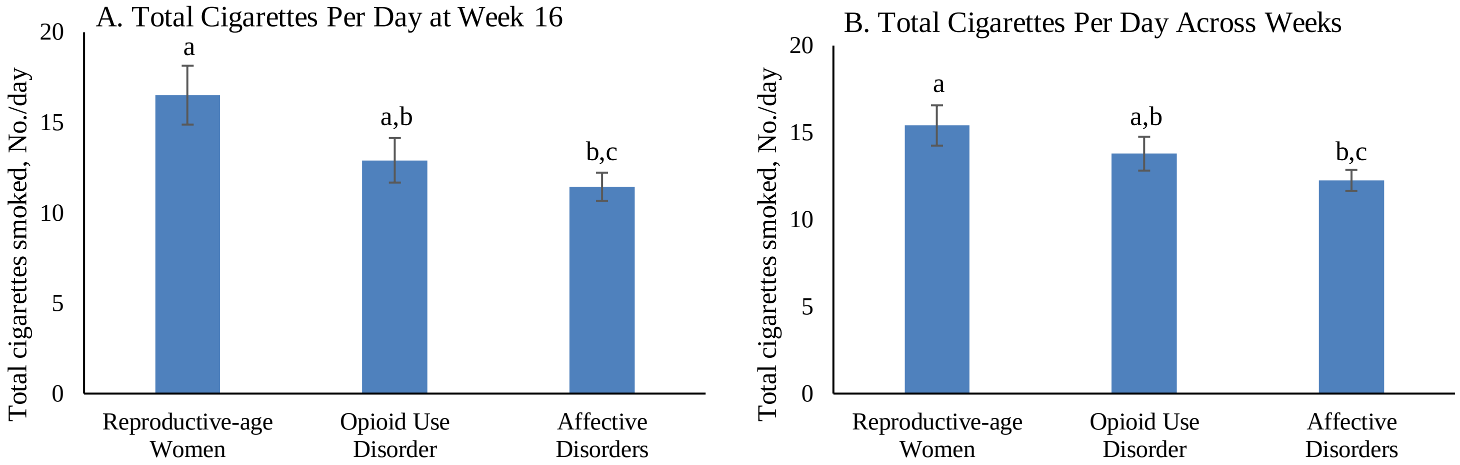

© 2024 Higgins ST et al. *JAMA Network Open*

Biomarkers of Exposure

There was a significant main effect of study population on breath CO in analyses across weeks (Cohen d=0.08; P=.012; eFigure 2A), with LSmean (±SEM) levels in the AD population below OUD (14.41±0.71 versus 17.93±1.15) (t[308]=-2.73, P=.018) but not WSD population levels nor did levels in the OUD and WSD populations differ significantly. There was also a significant main effect of study population on cotinine levels analyzed across weeks 8 and 16 (Cohen d=0.22; P=.023; eFigure 2B) with LSMean levels in the AD population below OUD (2690.62±192.08 versus 3666.27±316.80) (t[510]=2.74, P=.017) but not WSD levels nor did the OUD and WSD levels differ.

eFigure 2. Biomarkers of Exposure According to Study Population

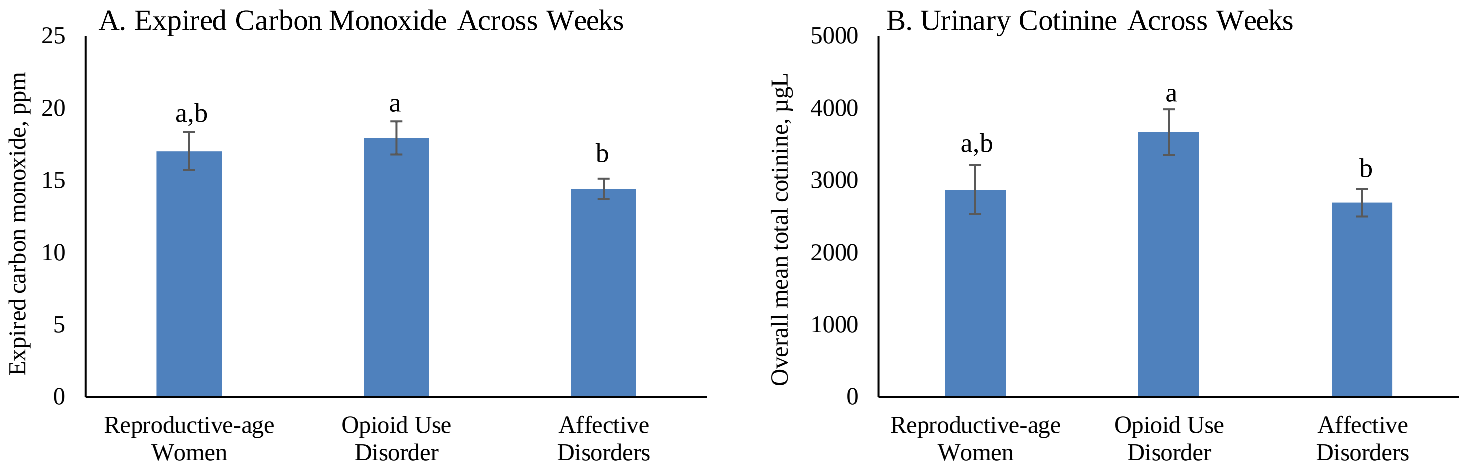

© 2024 Higgins ST et al. *JAMA Network Open*

Dependence Severity

There was a significant main effect of population on week-16 FTND total scores (Cohen d=0.29; P=.035; eFigure 3A), with LSmean (±SEM) scores lower in the AD than OUD population (3.49±0.14 versus 4.07±0.20) (t[251]=2.42, P=.043); AD scores did not differ significantly from WSD scores nor did scores in the WSD and OUD populations differ significantly. In analyses across weeks, again there was a main effect of population (Cohen d=0.26; P=0.004; eFigure 3B) with LSmean (±SEM) scores lower in the AD than OUD population (3.48±0.12 versus 4.12±0.17) (t[269]=3.20, P=.004); AD scores did not differ significantly from the WSD population nor did scores in the WSD and OUD populations differ significantly.

There was a significant week-16 main effect of population in analyses of WISDM primary-motive subscale scores (Cohen d=0.31; P=.024; eFigure 3C), with lower primary-motive-subscale severity scores in the AD than OUD population (13.35±0.42 versus 15.20±0.60) (t[249]=2.56, P=.030); AD scores did not differ significantly from the WSD population nor did scores in the WSD and OUD populations differ significantly.

eFigure 3. Dependence Severity According to Study Population

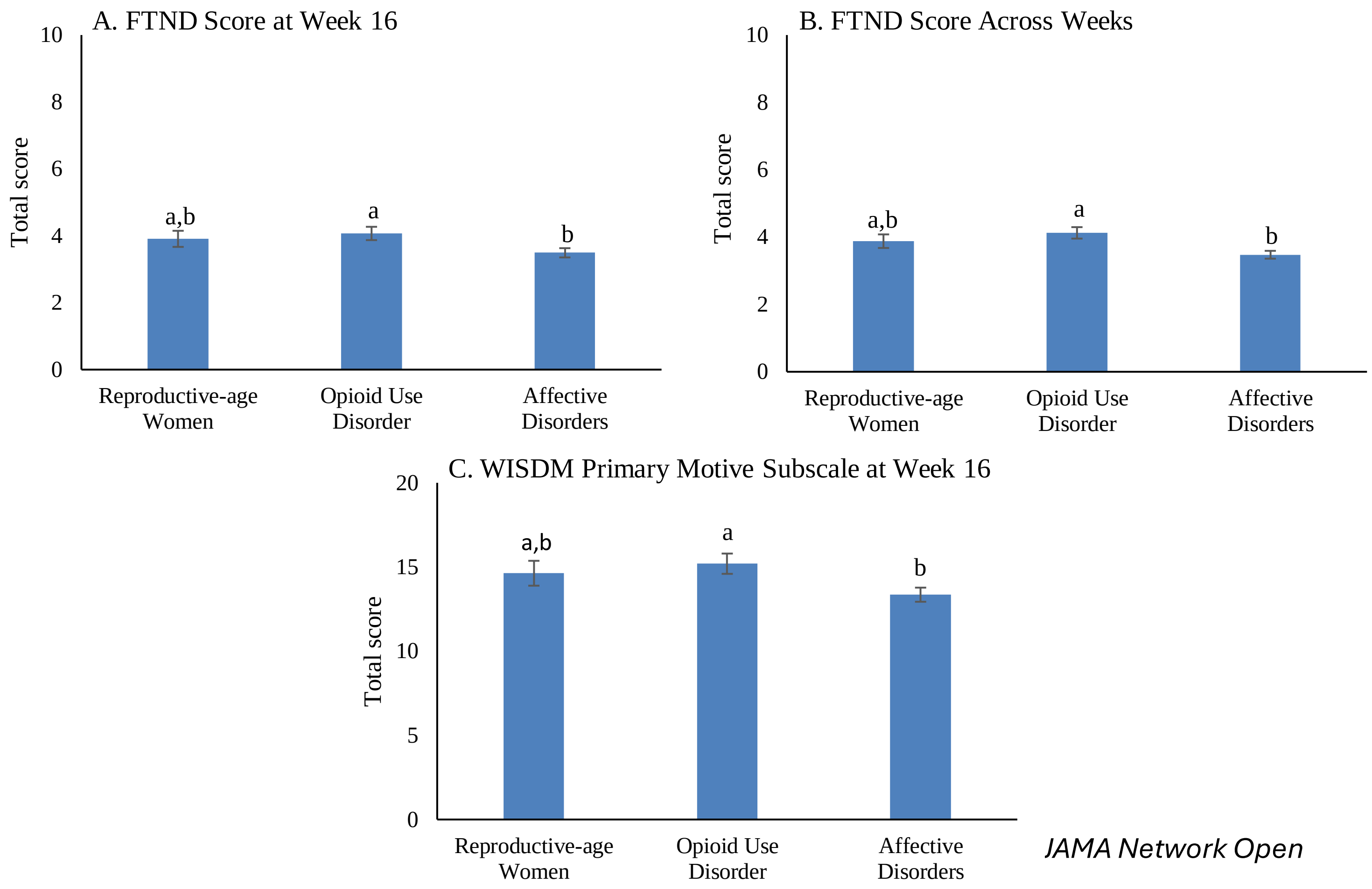

Craving and Withdrawal

In analyses across weeks, there was a significant main effect of population on QSU Factor-1 ratings of study cigarettes (Cohen d=0.12, Ps≤.010; eFigure 4A), with LSmean (±SEM) ratings in the OUD population greater than in the AD (3.38±0.18 versus 2.76±0.12) (t[309]=2.84, P=.013) and WSD (3.38±0.18 versus 2.67±0.22) (t[309]=2.55, P=.030) populations; AD scores did not significantly differ from the WSD population. There were also significant main effects of time for QSU Factor-1 (Cohen d=0.24, P<.001; eFigure 4B) and Factor-2 ratings (Cohen d = 0.17, P<.001; eFigure 4C), with decreases across weeks in Factor-1 (-.040±.006) (t[1874]=6.18, P<.001 and Factor 2 (-.021±.005) (t[1874]=4.45, P<.001).

The only significant effect on MNWS total scores across weeks was an interaction of population and time (Cohen d=0.10, P=.003; eFigure 4D), corresponding to steeper decreases across weeks in the AD (-.036±.003) (t[4062]=10.46, P<.001) compared to the OUD populations (-.015±.005) (t[4062]=2.98, P=.003); the WSD population did not differ significantly from the AD or OUD populations.The only significant effect across weeks on desire-to-smoke was a main effect of time with scores decreasing across weeks (-.042±.004) (t[4064]=10.77, Cohen's d = 0.30, P<.001; eFigure 4E).

© 2024 Higgins ST et al. *JAMA Network Open*

eFigure 4. Craving and Withdrawal

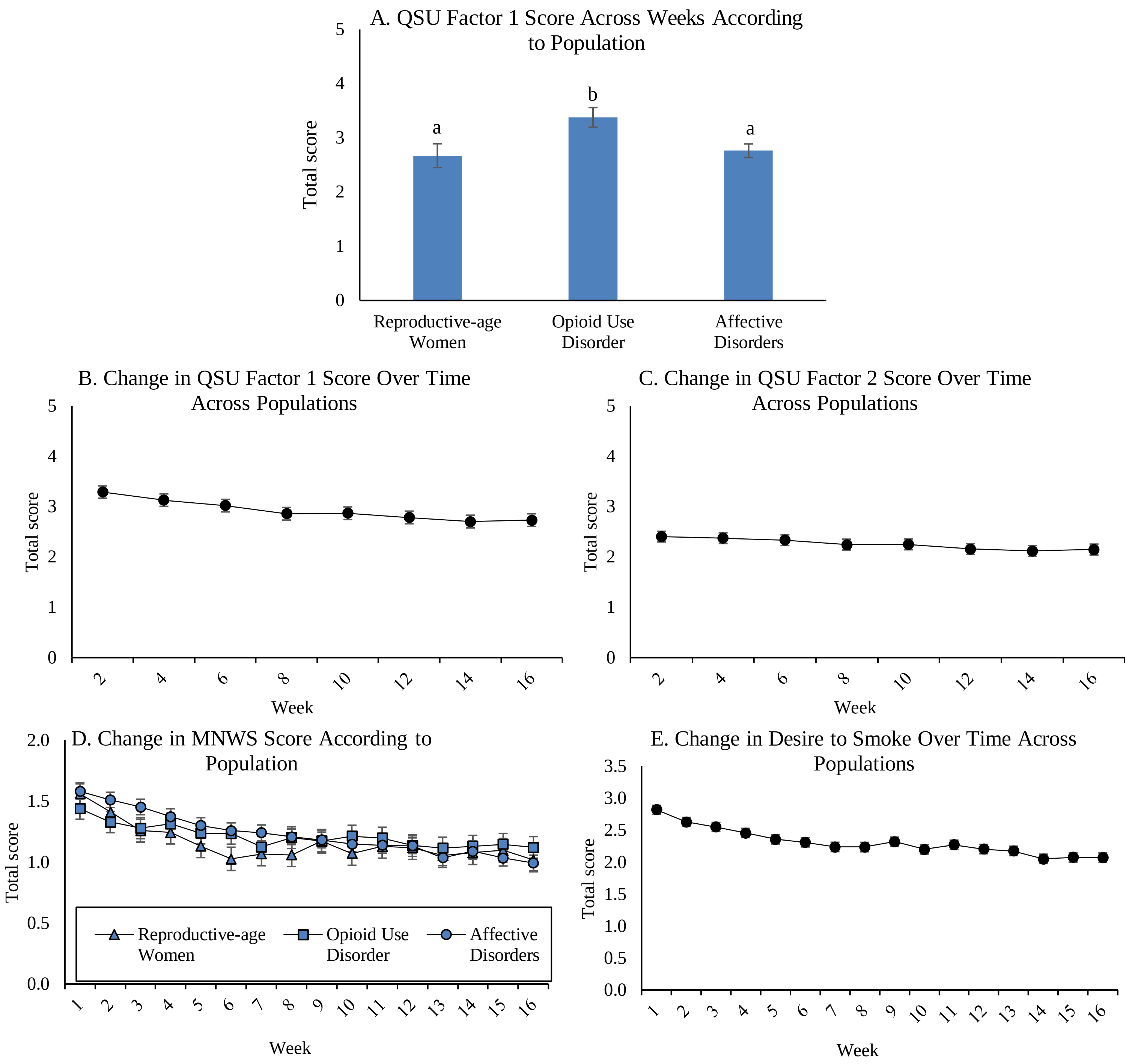

© 2024 Higgins ST et al. *JAMA Network Open*

eFigure 5. Number of Total Cigarettes Smoked Per Day Over Time According to Condition, Across Populations and According to Study Population

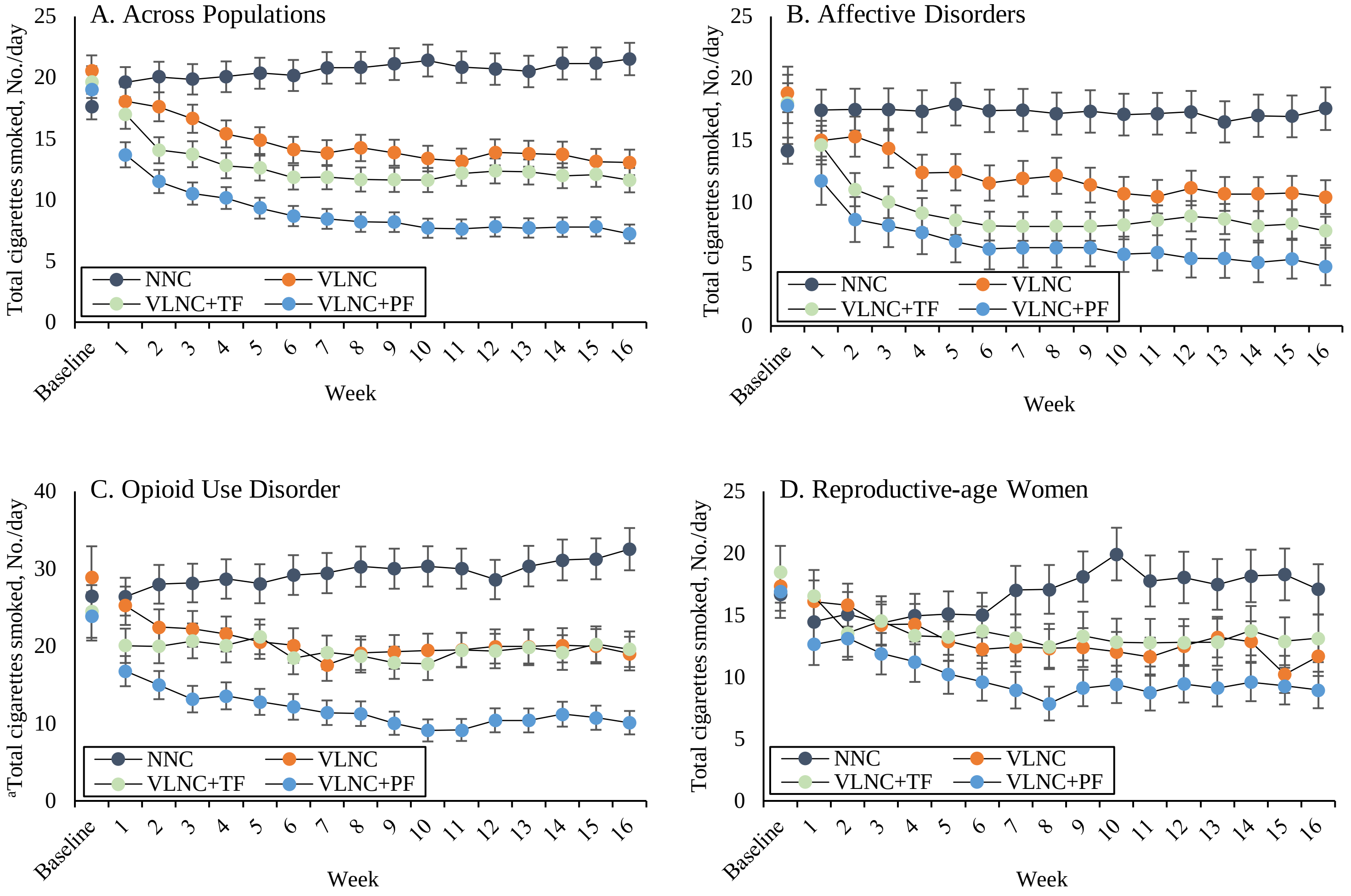

[a]*Note: The scale used on the y-axis for the population with opioid use disorder (Panel C) differs from other populations (Panels A, B, and D).*

© 2024 Higgins ST et al. *JAMA Network Open*

eFigure 6. Number of Total Cigarettes Smoked Per Day at Week 16 According to Condition, Across Populations and According to Study Population

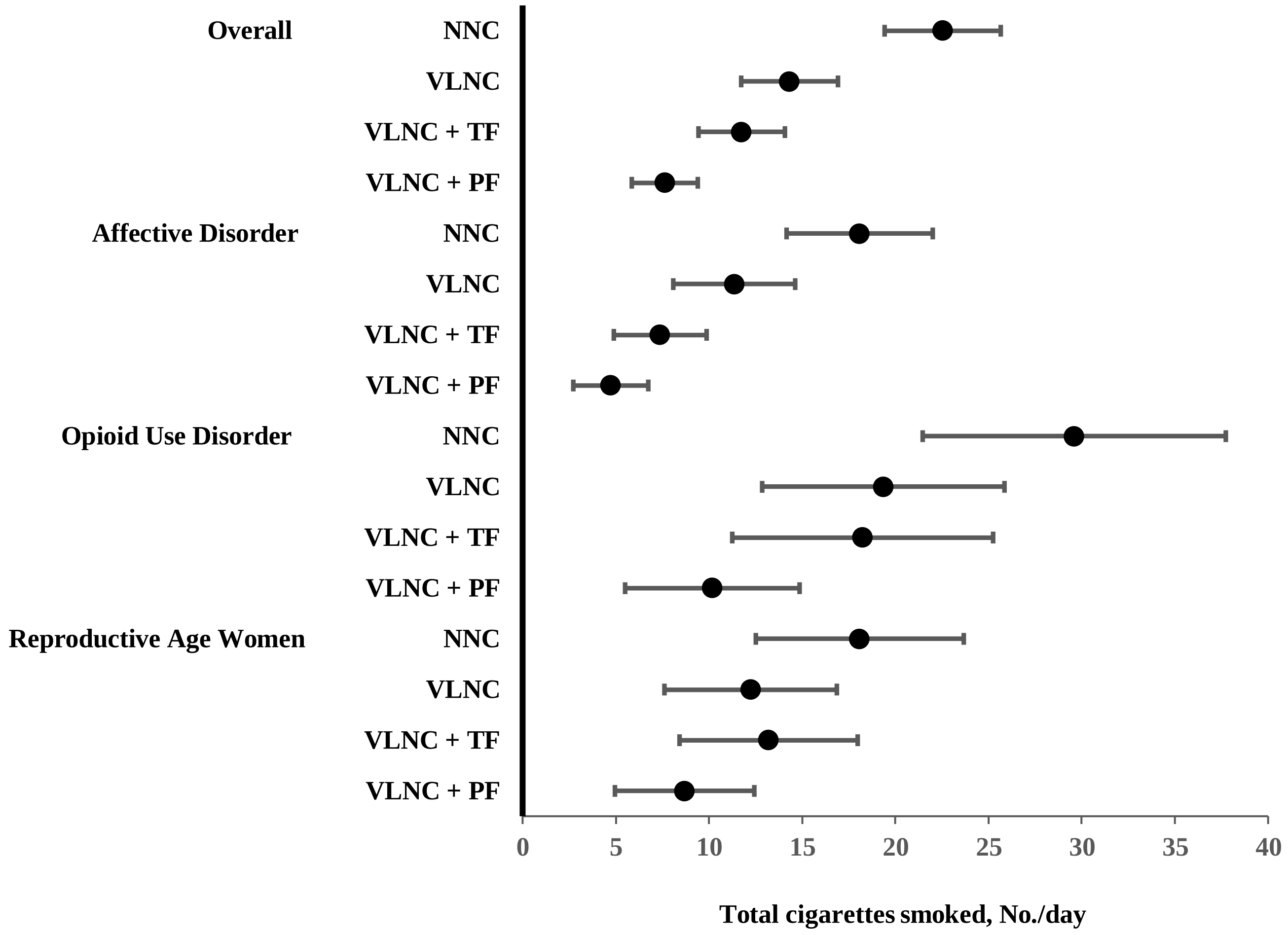

© 2024 Higgins ST et al. *JAMA Network Open*

eFigure 7. Number of Non-study Cigarettes Smoked Per Day According to Condition

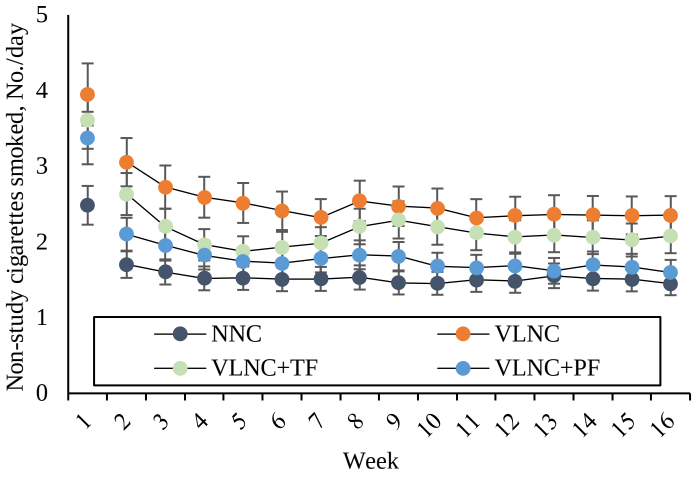

© 2024 Higgins ST et al. *JAMA Network Open*

Smoking Abstinence

There was a significant main effect of condition across populations on LSmean number of trial days without smoking ($X^2(3)=33.11$; P<.001, eFigure 7), with more days in VLNC+PF (23.35±3.93) versus NNC (0.73±1.34) (z=6.10, P<.001), VLNC (2.92±1.12) (z=3.62, P=.002), and VLNC+TF (9.04±2.68) (z=2.59, P=.048). Mean days abstinent in VLNC+TF was greater than NNC (z=3.37, P=.004) but not VLNC; VLNC days abstinent did not differ significantly from NNC. There were no significant differences by condition on outcomes in the post-trial 24-hr abstinence-test nor 30-day smoking-cessation rates.

eFigure 8. Cigarette Abstinence According to Condtion

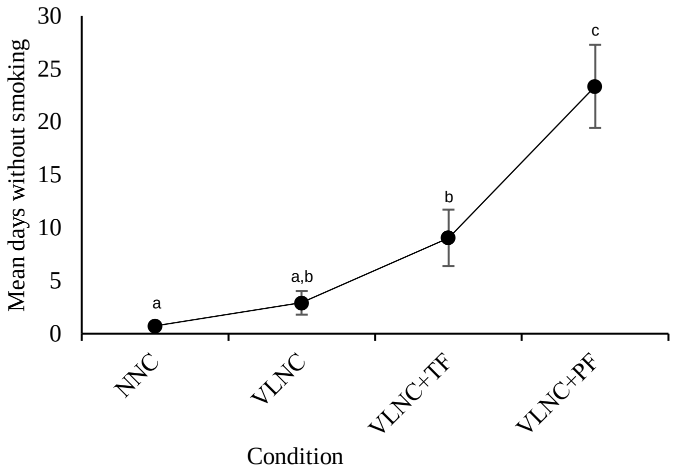

© 2024 Higgins ST et al. *JAMA Network Open*

eFigure 9. Nicotine Dependence Severity and Cigarette Craving According to Condition

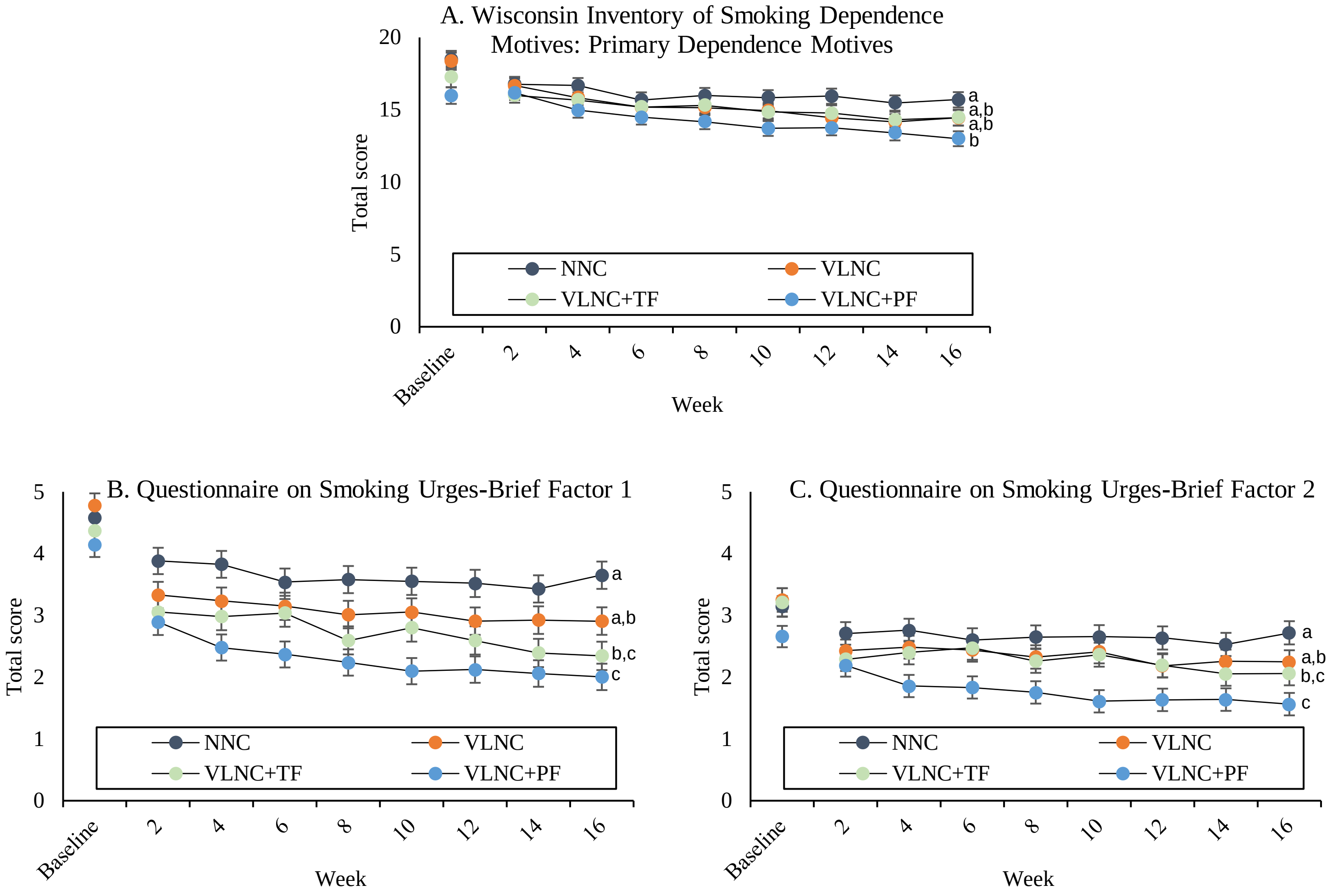

© 2024 Higgins ST et al. *JAMA Network Open*

eTables

Participants

Only one baseline characteristic (FTND total scores) differentiated completers from dropouts (mean [±SD] = 4.97±2.23 versus 5.59±2.07, respectively, P=.044) (eTable 1).

eTable 1: Participant Characteristics

	Overall n = 326	Completers n = 260	Dropouts n = 66	p-value
Condition				0.732
NNC Only	83 (25.46)	67 (25.77)	16 (24.24)	
VLNCC + PF	84 (27.77)	70 (26.92)	14 (21.21)	
VLNCC + TF	74 (22.70)	57 (21.92)	17 (25.76)	
VLNCC Only	85 (26.07)	66 (25.38)	19 (28.79)	
Population				0.074
Disadvantaged Women	80 (24.54)	57 (21.92)	23 (34.85)	
Opioid Dependent	74 (22.70)	63 (24.23)	11 (16.67)	
Affective Disorders	172 (52.76)	140 (53.85)	32 (48.48)	
Age (M ± SD)	40.09 ± 10.79	40.05 ± 10.76	40.21 ± 10.98	0.968
Gender (% Female)	243 (74.54)	193 (74.23)	50 (75.76)	0.799
Race/Ethnicity				0.902
Non-Latino White	262 (80.37)	210 (80.77)	52 (78.79)	
Non-Latino Black	33 (10.12)	26 (10.00)	7 (10.61)	
Latino	12 (3.68)	10 (3.85)	2 (3.03)	
Non-Latino Other or >1 race	19 (5.83)	14 (5.38)	5 (7.58)	
Education				0.916
<High school	30 (9.20)	24 (9.23)	6 (9.09)	
High school graduate or equivalent or some college	201 (61.66)	158 (60.77)	43 (65.15)	
Associate's degree	43 (13.19)	35 (13.46)	8 (12.12)	
>= College graduate	52 (15.95)	43 (16.54)	9 (13.64)	
Marital Status				0.866
Married	51 (15.64)	41 (15.77)	10 (15.15)	
Never married	180 (55.21)	145 (55.77)	35 (53.03)	
Divorced, separated, or widowed	95 (29.14)	74 (28.46)	21 (31.82)	
Primary smoker of mentholated cigarettes	131 (40.18)	107 (41.15)	24 (36.36)	0.478
Cigarettes smoked per day (M ± SD)	17.40 ± 8.87	17.14 ± 8.78	18.42 ± 9.22	0.278
Urine Cotinine Level, ng/ml (M ± SD)	5043.55 ± 3680.12	5141.61 ± 3847.18	4660.19 ± 2931.27	0.973
Breath CO level (M ± SD)	20.46 ± 13.28	20.60 ± 13.42	19.88 ± 12.79	0.675
Age started smoking regularly (M ± SD)	16.39 ± 4.26	16.59 ± 4.28	15.61 ± 4.09	0.050

© 2024 Higgins ST et al. *JAMA Network Open*

Fagerstrom Test for Cigarette Dependence (M ± SD)	5.09 ± 2.21	4.97 ± 2.23	5.59 ± 2.07	**0.044**
Used Other Tobacco Products, Last 30 days	23 (7.06)	17 (6.54)	6 (9.09)	0.470
Used E-cigarettes, Last 30 days	42 (12.88)	31 (11.92)	11 (16.67)	0.304
E-cigarette Flavors, Last 30 days				
Fruit	23 (7.06)	16 (6.15)	7 (10.61)	0.207
Menthol or mint	12 (3.68)	7 (2.69)	5 (7.58)	0.072
Candy or other sweets	4 (1.23)	2 (0.77)	2 (3.03)	0.136
Other	1 (0.31)	0 (0.00)	1 (1.52)	0.203

Note: Unless otherwise indicated, data are expressed as number (percentage)

© 2024 Higgins ST et al. *JAMA Network Open*

eTable 2. E-cigarette flavors selected (VLNC + PF condition).

Week 16	
Flavor	# of choices (%)
Mango	47 (24.87)
Fruit Medley	37 (19.58)
Classic Menthol	23 (12.17)
Cool Mint	20 (10.58)
Classic Tobacco	20 (10.58)
Crème Brûlée	17 (8.99)
Cool Cucumber	16 (8.47)
Virginia Tobacco	9 (4.76)

Across all weeks	
Flavor	# of choices (%)
Mango	811 (23.22)
Fruit Medley	685 (19.62)
Classic Menthol	424 (12.14)
Cool Mint	408 (11.68)
Classic Tobacco	372 (10.65)
Crème Brûlée	323 (9.25)
Cool Cucumber	294 (8.42)
Virginia Tobacco	175 (5.01)

© 2024 Higgins ST et al. *JAMA Network Open*

Adverse Events

Most participants reported an adverse event (284/326, 87.1%) (eTables 2-3). There were no significant differences between experimental conditions in incidence, total number, or number of serious or severe adverse events (eTables 2-5).

eTable 3: Total Number of Adverse Events

Adverse Event Description	Overall	NNC Only	VLNC Only	VLNC + TF	VLNC + PF
Total Number of Events	1225	332	332	261	300
BDI Category Increase	167	52	40	34	41
CPD Increase > 200%	15	7	1	6	1
OASIS Score Increase	177	52	43	42	40
Blood and lymphatic system disorders	1	1	0	0	0
Cardiac disorders	20	6	5	4	5
Ear and labyrinth disorders	3	0	1	0	2
Eye disorders	5	1	1	2	1
Gastrointestinal disorders	84	15	25	21	23
General disorders and administration site conditions	103	26	28	21	28
Immune system disorders	5	3	2	0	0
Infections and infestations	66	17	24	11	14
Injury, poisoning and procedural complications	27	7	7	5	8
Investigations	5	1	2	1	1
Metabolism and nutrition disorders	5	0	1	2	2
Musculoskeletal and connective tissue disorders	49	14	16	7	12
Neoplasms benign, malignant and unspecified	4	3	0	0	1
Nervous system disorders	61	8	23	19	11
Pregnancy, puerperium and perinatal conditions	2	2	0	0	0
Psychiatric disorders	161	54	43	27	37
Renal and urinary disorders	5	2	2	0	1
Reproductive system and breast disorders	5	2	1	1	1
Respiratory, thoracic and mediastinal disorders	160	38	45	41	36
Skin and subcutaneous tissue disorders	7	2	3	1	1
Social circumstances	1	1	0	0	0
Surgical and medical procedures	16	3	4	5	4
Vascular disorders	71	15	15	11	30

© 2024 Higgins ST et al. *JAMA Network Open*

eTable 4: Number of Participants with Any Adverse Event.

Adverse Event Description	Overall		NNC Only		VLNC Only		VLNC + TF		VLNC + PF	
	n	%	n	%	n	%	n	%	n	%
	284	87.1%	74	89.2%	75	88.2%	63	85.1%	72	85.7%
BDI Category Increase	109	33.4%	29	34.9%	29	34.1%	26	35.1%	25	29.8%
CPD Increase > 200%	10	3.1%	4	4.8%	1	1.2%	4	5.4%	1	1.2%
OASIS Score Increase	126	38.7%	33	39.8%	31	36.5%	31	41.9%	31	36.9%
Blood and lymphatic system disorders	1	0.3%	1	1.2%	0	0.0%	0	0.0%	0	0.0%
Cardiac disorders	15	4.6%	4	4.8%	3	3.5%	3	4.1%	5	6.0%
Ear and labyrinth disorders	2	0.6%	0	0.0%	1	1.2%	0	0.0%	1	1.2%
Eye disorders	5	1.5%	1	1.2%	1	1.2%	2	2.7%	1	1.2%
Gastrointestinal disorders	59	18.1%	10	12.0%	17	20.0%	15	20.3%	17	20.2%
General disorders and administration site conditions	83	25.5%	19	22.9%	25	29.4%	17	23.0%	22	26.2%
Immune system disorders	5	1.5%	3	3.6%	2	2.4%	0	0.0%	0	0.0%
Infections and infestations	57	17.5%	16	19.3%	23	27.1%	8	10.8%	10	11.9%
Injury, poisoning and procedural complications	19	5.8%	6	7.2%	4	4.7%	4	5.4%	5	6.0%
Investigations	5	1.5%	1	1.2%	2	2.4%	1	1.4%	1	1.2%
Metabolism and nutrition disorders	5	1.5%	0	0.0%	1	1.2%	2	2.7%	2	2.4%
Musculoskeletal and connective tissue disorders	46	14.1%	11	13.3%	16	18.8%	7	9.5%	12	14.3%
Neoplasms benign, malignant and unspecified	4	1.2%	3	3.6%	0	0.0%	0	0.0%	1	1.2%
Nervous system disorders	50	15.3%	7	8.4%	18	21.2%	15	20.3%	10	11.9%
Pregnancy, puerperium and perinatal conditions	2	0.6%	2	2.4%	0	0.0%	0	0.0%	0	0.0%
Psychiatric disorders	99	30.4%	28	33.7%	28	32.9%	19	25.7%	24	28.6%
Renal and urinary disorders	5	1.5%	2	2.4%	2	2.4%	0	0.0%	1	1.2%
Reproductive system and breast disorders	4	1.2%	1	1.2%	1	1.2%	1	1.4%	1	1.2%
Respiratory, thoracic and mediastinal disorders	89	27.3%	23	27.7%	28	32.9%	19	25.7%	19	22.6%
Skin and subcutaneous tissue disorders	7	2.1%	2	2.4%	3	3.5%	1	1.4%	1	1.2%
Social circumstances	1	0.3%	1	1.2%	0	0.0%	0	0.0%	0	0.0%
Surgical and medical procedures	14	4.3%	2	2.4%	3	3.5%	5	6.8%	4	4.8%
Vascular disorders	48	14.7%	11	13.3%	13	15.3%	8	10.8%	16	19.0%

© 2024 Higgins ST et al. *JAMA Network Open*

eTable 5: Count of Serious and Severe Adverse Events

Description	Overall	NNC Only	VLNC Only	VLNC + TF	VLNC + PF
Number of serious adverse events[a]	19	9	4	4	2
Events related, probably related or possibly related	4	3	0	1	0
Number of severe adverse events	60	12	12	14	22
Events related, probably related or possibly related	24	6	3	6	9

© 2024 Higgins ST et al. *JAMA Network Open*

eTable 6. Description of Serious and Severe Adverse Events

Seriousness	Severity	Treatment Group	Type of Event
Serious	Mild	NNC Only	BDI Category Increase
Serious	Mild	NNC Only	Irritability
Serious	Mild	NNC Only	OASIS Score Increase
Serious	Mild	NNC Only	Pregnancy
Serious	Mild	NNC Only	Hypertension
Serious	Moderate	NNC Only	Tumors on parotid gland
Serious	Moderate	NNC Only	Hysterectomy
Serious	Moderate	VLNC Only	Chest pain
Serious	Moderate	VLNC + TF	Dehydration
Serious	Severe	NNC Only	Esophageal perforation
Serious	Severe	NNC Only	Psychiatric disorders (hospitalization)
Serious	Severe	VLNC Only	Fever
Serious	Severe	VLNC Only	Renal calculi (kidney stones)
Serious	Severe	VLNC Only	Pneumonia
Serious	Severe	VLNC Only	Stent placement surgery for blood clots
Serious	Severe	VLNC + TF	Cocaine overdose
Serious	Severe	VLNC + TF	Hypertension
Serious	Severe	VLNC + PF	Hyperglycemia
Serious	Severe	VLNC + PF	Pneumonia
Serious	Fatal	VLNC + TF	Death
Not Serious	Severe	NNC Only	Atrial fibrillation
Not Serious	Severe	NNC Only	BDI Category Increase
Not Serious	Severe	NNC Only	Hypertension
Not Serious	Severe	NNC Only	Hypertension
Not Serious	Severe	NNC Only	Hypertension
Not Serious	Severe	NNC Only	Hypertension
Not Serious	Severe	NNC Only	Hypertension
Not Serious	Severe	NNC Only	Hypertension
Not Serious	Severe	NNC Only	Toothache
Not Serious	Severe	NNC Only	Trauma related psychiatric event with ER visit
Not Serious	Severe	VLNC Only	BDI Category Increase
Not Serious	Severe	VLNC Only	Bruising
Not Serious	Severe	VLNC Only	Hypertension
Not Serious	Severe	VLNC Only	Hypertension
Not Serious	Severe	VLNC Only	Hypertension
Not Serious	Severe	VLNC Only	Hypertension
Not Serious	Severe	VLNC Only	Hypertension
Not Serious	Severe	VLNC Only	Hypertension
Not Serious	Severe	VLNC Only	Hypertension
Not Serious	Severe	VLNC + TF	BDI Category Increase
Not Serious	Severe	VLNC + TF	Cluster Migraines
Not Serious	Severe	VLNC + TF	Cough
Not Serious	Severe	VLNC + TF	Hypertension
Not Serious	Severe	VLNC + TF	Hypertension
Not Serious	Severe	VLNC + TF	Hypertension
Not Serious	Severe	VLNC + TF	Hypertension
Not Serious	Severe	VLNC + TF	Hypertension
Not Serious	Severe	VLNC + TF	Hypertension
Not Serious	Severe	VLNC + TF	Hypertension
Not Serious	Severe	VLNC + TF	Hypertension
Not Serious	Severe	VLNC + PF	BDI Category Increase
Not Serious	Severe	VLNC + PF	Hypertension
Not Serious	Severe	VLNC + PF	Hypertension
Not Serious	Severe	VLNC + PF	Hypertension

© 2024 Higgins ST et al. *JAMA Network Open*

Not Serious	Severe	VLNC + PF	Hypertension
Not Serious	Severe	VLNC + PF	Hypertension
Not Serious	Severe	VLNC + PF	Hypertension
Not Serious	Severe	VLNC + PF	Hypertension
Not Serious	Severe	VLNC + PF	Hypertension
Not Serious	Severe	VLNC + PF	Hypertension
Not Serious	Severe	VLNC + PF	Hypertension
Not Serious	Severe	VLNC + PF	Hypertension
Not Serious	Severe	VLNC + PF	Hypertension
Not Serious	Severe	VLNC + PF	Hypertension
Not Serious	Severe	VLNC + PF	Hypertension
Not Serious	Severe	VLNC + PF	Hypertension
Not Serious	Severe	VLNC + PF	Hypertension
Not Serious	Severe	VLNC + PF	Hypertension
Not Serious	Severe	VLNC + PF	Hypertension
Not Serious	Severe	VLNC + PF	Nasal fracture

© 2024 Higgins ST et al. *JAMA Network Open*
