# Supplementary material for: Reduced Nicotine Cigarettes and E-Cigarettes in High-Risk Populations: 3 Randomized Clinical Trials
Source: JAMA Netw Open. 2024 Sep 6;7(9):e2431731. doi: 10.1001/jamanetworkopen.2024.31731 (PMC11380105; doi:10.1001/jamanetworkopen.2024.31731)
Supplement: Supplement 3. — Data Sharing Statement [file jamanetwopen-e2431731-s003.pdf]

Data Sharing Statement

Higgins. Reduced Nicotine Cigarettes and E-Cigarettes in High-Risk Populations. *JAMA Netw Open*. Published September 06, 2024. doi:10.1001/jamanetworkopen.2024.31731

Data
Data available: Yes
Data types: Deidentified participant data, Other (please specify)
Additional Information: Study protocols
How to access data: National Institute of Drug Abuse's data sharing site (https://datashare.nida.nih.gov/index.php/)
When available: beginning date: 12-30-2028

Supporting Documents
Document types: Statistical/analytic code, Other (please specify)
Additional Information: study protocols
How to access documents: National Institute of Drug Abuse's data sharing site (https://datashare.nida.nih.gov/index.php/)
When available: beginning date: 12-30-2028

Additional Information
Who can access the data: researchers whose proposed use of the data has been approved
Types of analyses: for any purpose
Mechanisms of data availability: without investigator support
